# Genome and Methylome analysis of a phylogenetic novel *Campylobacter coli* cluster with *C. jejuni* introgression

**DOI:** 10.1099/mgen.0.000679

**Published:** 2021-10-18

**Authors:** Anastasia-Lisa Dieckmann, Thomas Riedel, Boyke Bunk, Cathrin Spröer, Jörg Overmann, Uwe Groß, Oliver Bader, Wolfgang Bohne, Burkhard Morgenstern, Morteza Hosseini, Andreas E. Zautner

**Affiliations:** ^1^​ Institut für Medizinische Mikrobiologie und Virologie, Universitätsmedizin Göttingen, Göttingen, Germany; ^2^​ Leibniz-Institut DSMZ–Deutsche Sammlung von Mikroorganismen und Zellkulturen GmbH, Braunschweig, Germany; ^3^​ Deutsches Zentrum für Infektionsforschung (DZIF) Hannover–Braunschweig, Braunschweig, Germany; ^4^​ Institut für Mikrobiologie und Genetik, Abteilung Bioinformatik, Universität Göttingen, Göttingen, Germany; ^5^​ IEETA/DETI, University of Aveiro, Aveiro, Portugal

**Keywords:** *Campylobacter coli*, *Campylobacter jejuni*, genome, introgression, methylome, PacBio SMRT sequencing

## Abstract

The intriguing recent discovery of *Campylobacter coli* strains, especially of clade 1, that (i) possess mosaic *C. coli */* C. jejuni* alleles, (ii) demonstrate mixed multilocus sequence types (MLSTs) and (iii) have undergone genome-wide introgression has led to the speculation that these two species may be involved in an accelerated rate of horizontal gene transfer that is progressively leading to the merging of both species in a process coined ‘despeciation’. In an MLST-based neighbour-joining tree of a number of *C. coli* and *C. jejuni* isolates of different clades, three prominent *Campylobacter* isolates formed a seemingly separate cluster besides the previously described *C. coli* and *C. jejuni* clades. In the light of the suspected, ongoing genetic introgression between the *C. coli* and *C. jejuni* species, this cluster of *Campylobacter* isolates is proposed to present one of the hybrid clonal complexes in the despeciation process of the genus. Specific DNA methylation as well as restriction modification systems are known to be involved in selective uptake of external DNA and their role in such genetic introgression remains to be further investigated. In this study, the phylogeny and DNA methylation of these putative *C. coli */* C. jejuni* hybrid strains were explored, their genomic mosaic structure caused by *C. jejuni* introgression was demonstrated and basic phenotypic assays were used to characterize these isolates. The genomes of the three hybrid *Campylobacter* strains were sequenced using PacBio SMRT sequencing, followed by methylome analysis by Restriction-Modification Finder and genome analysis by Parsnp, Smash++ and blast. Additionally, the strains were phenotypically characterized with respect to growth behaviour, motility, eukaryotic cell invasion and adhesion, autoagglutination, biofilm formation, and water survival ability. Our analyses show that the three hybrid *Campylobacter* strains are clade 1 *C *. *coli* strains, which have acquired between 8.1 and 9.1 % of their genome from *C. jejuni *. The *C. jejuni* genomic segments acquired are distributed over the entire genome and do not form a coherent cluster. Most of the genes originating from *C. jejuni* are involved in chemotaxis and motility, membrane transport, cell signalling, or the resistance to toxic compounds such as bile acids. Interspecies gene transfer from *C. jejuni* has contributed 8.1–9.1% to the genome of three *C. coli* isolates and initiated the despeciation between *C. jejuni* and *C. coli *. Based on their functional annotation, the genes originating from *C. jejuni* enable the adaptation of the three strains to an intra-intestinal habitat. The transfer of a fused type II restriction-modification system that recognizes the CAYNNNNNCTC/GAGNNNNNRTG motif seems to be the key for the recombination of the *C. jejuni* genetic material with *C. coli* genomes.

## Data Summary

1. Closed genome sequences of all three *C. coli* isolates were submitted to NCBI GenBank (BioProject PRJNA418666).
2. RM-systems and methylation motifs are available via the index of the REBASE genomes database (http://tools.neb.com/genomes/) and under the following reference numbers: 20811 (* C. coli* meC0280), 27867 (* C. coli* meC0281) and 27865 (* C. coli* meC0467).
3. All three bacterial isolates have been deposited in the open collection of the Leibniz Institute DSMZ–German Collection of Microorganisms and Cell Cultures under the following DSM numbers: DSM 101856 (* C. coli* meC0280), DSM 104626 (* C. coli* meC0281) and DSM 104627 (* C. coli* meC0467).

**Impact Statement**

*
Campylobacter jejuni* and *Campylobacter coli* are the most important food-associated bacterial pathogens for acute enteritis. Therefore, there is a need for monitoring along the food production chain from the poultry farm to the diseased individual. A phenomenon has been described for a subgroup of *C. coli* strains where genes are taken up from another bacterial species, *C. jejuni *. This progressive process has been observed to lead to the fusion of the two bacterial species in the course of evolution into a common bacterial species. Therefore, this process is called ‘despeciation’. The mechanisms for this have been poorly described so far. By analysing the DNA sequences and also the DNA methylation of such hybrid strains, we reveal possible mechanisms for the uptake of genes from other bacterial species during despeciation. In particular, the role of restriction-modification systems, which degrade or spare DNA based on its methylation patterns, is discussed. By increasing the awareness of the existence of these hybrid strains, especially in the agricultural niche, it will also be possible to deal with these phenomena in a more structured way in diagnostics. Further studies investigating targeted DNA uptake from the other bacterial species should be performed based on the present data.

## Introduction

The two most common disease-causing *Campylobacter* species are *C. jejuni* and *C. coli *, accounting for about 85 and 15% of all human *Campylobacter* infections, respectively [1]. Leading to abdominal cramps, watery or bloody diarrhoea, and post-infectious complications such as Guillain-Barré syndrome and reactive arthritis [2], these species are thought to cause about 200000 reported cases of campylobacteriosis every year [3].

Despite the fact that *C. coli* and *C. jejuni* share 86.5% identity in their seven multi-locus sequence typing (MLST) housekeeping genes (*aspA*, *glnA*, *gltA*, *glyA*, *pgm*, *tkt* and *uncA*), their identities as phylogenetically discrete species is confirmed by whole genome sequencing [4, 5]. MLST further divides *C. coli* into three clades which, interestingly, also colonize distinct niches and have differing gene exchange behaviours. *C. coli* clade 1 is most frequently isolated from clinical samples as well as farm animals and undergoes high levels of intraclade and interspecies genetic exchange. Isolates from the closely related clades 2 and 3, which are highly distinct from clade 1, are commonly found in water environments and water birds and undergo little genetic exchange (5–10 times less than *C. jejuni *) [6–8].

Ecological separation is thought to be the primary factor for the maintenance of the phylogenetically distinct groups of *C. coli* and *C. jejuni* [5]. For example, *C. coli* is found to dominate in swine, while *C. jejuni* dominates in chicken and cattle [9]. Furthermore, different *C. jejuni* clonal complexes have been found to dominate in distinct wild bird species [7, 10, 11].

Recent observations of *C. coli* and *C. jejuni* isolates that possess mosaic *C. coli */* C. jejuni* alleles [7], that represent mixed *C. coli */* C. jejuni* multilocus sequence types (MLSTs) [5], and that have undergone genome-wide introgression [12], however, suggest that there may be a breakdown in the ecological barriers that have historically kept the two species separate.

In particular, *C. coli* clade 1 isolates have been observed to progressively accumulate *C. jejuni* DNA, with 10 and 23% of its core genome replaced by *C. jejuni* DNA in sequence type (ST)-828 and ST-1150 clonal complexes, respectively [12]. The impact of the increased horizontal gene transfer between the species is also seen in the emergence of multi-host *C. jejuni* lineages that are capable of colonizing both birds and mammals [13]. Thus, the rapid expansion of a single *C. coli* lineage found in both agricultural animals and human disease [6] could be demonstrated. Furthermore, horizontal gene transfer plays a role in the widespread acquisition of antimicrobial resistance [14]. This progressive introgression of the *C. jejuni* and *C. coli* genomes has been hypothesized to eventually lead to a merging of the *C. jejuni* species, particularly with *C. coli* clade 1 isolates in the agricultural niche, in a process termed ‘despeciation’.

In bacteria, DNA methylation plays a role in discriminating between self and extraneous DNA, which is a prerequisite for protecting the host genome from extraneous – sometimes invasive – DNA by restriction-modification (RM) systems [15]. RM systems typically consist of two components: a restriction endonuclease recognizing a specific (methylated) DNA motif and an associated DNA methyltransferase that methylates the same DNA and prevents or prepares its cleavage by the restriction endonuclease [16]. In the context of introgression, it is therefore likely that specific methylated DNA motifs and associated RM systems also play a role. Thus, it will be necessary to determine whether the selective activity of an RM system can facilitate the incorporation of *C. jejuni* DNA fragments into specific *C. coli* strains.

The three isolates meC0280, meC0281 and meC0467 reported here are *Campylobacter* strains that were isolated from turkey meat slaughtered in Berlin-Brandenburg, Germany. Despite being classified as *C. coli* by MALDI-TOF MS analysis [17], MLST-based analysis showed that this cluster is more closely related to *C. jejuni* and may have a possible hybrid species origin. In light of the suspected, ongoing genetic introgression between the *C. coli* and *C. jejuni* species, this study aimed to genotypically and phenotypically characterize these three isolates in order to investigate the potential hybrid origin of these isolates.

## Methods

### Bacterial isolates, culture conditions and eukaryotic cell culture conditions

Two *C. jejuni* reference strains – NCTC 11168 (DSM 27585) and 81116 (DSM 24189) – were obtained from the Leibniz Institute DSMZ–German Collection of Microorganisms and Cell Cultures (Braunschweig, Germany).

*C. jejuni* reference strain 81-176 (ATCC-BAA-2151) and *C. coli* reference strain RM 2228 (ATCC-BAA-1061) were obtained from the American Type Culture Collection (Manassas, VA, USA). *C. coli* BfR-CA-9557 (DSM 100395) and the isolates under investigation – meC0280, meC0281 and meC0467 – isolated from turkey meat slaughtered in Berlin-Brandenburg were kindly provided by Thomas Alter, Free University of Berlin (at that time at the BfR in Berlin, Germany).

The bacterial isolates were cultured on Columbia agar base (Merck) supplemented with 5% sheep blood (ThermoFisher Scientific) and incubated at 42 °C under microaerophilic conditions (5% O_2_, 10% CO_2_, 85% N_2_). The bacteria were passaged every 48 h.

### DNA extraction and MLST

Genomic DNA of all *Campylobacter* isolates was extracted using the QIAamp DNA Mini Kit (Qiagen) according to the manufacturer’s instructions.

The MLS type was established using amplification and sequencing primers reported previously (https://pubmlst.org/campylobacter/info/primers.shtml). The cycling conditions were 94 °C for 1 min, followed by 35 cycles of 94 °C for 2 min, 50 °C for 1 min and 72 °C for 1 min, followed by a final elongation step of 72 °C for 5 min [18]. Amplicons of the seven genes included in the *C. coli */* C. jejuni* MLST scheme were sent for sequencing to Microsynth Seqlab using 10 pmol of the respective sequencing primer. The mega x software for Linux was used for calculation of an MLST-based evolutionary history using the neighbour-joining method [19, 20]. Evolutionary distances were computed using the Maximum Composite Likelihood method [21]. Apart from the three potential hybrid isolates, the remaining sequences of the MLST-STs were taken from the pubMLST database.

### Alignment to *C. coli *- and *C. jejuni *-specific gene markers

From a study by Méric and co-workers, the gene sequences of 21 *C *. *coli *- and 27 *C *. *jejuni *-specific markers were obtained [22]. These gene sequences were aligned to the genomes of meC0280, meC0281 and meC0467 to determine their presence in their genomes and thereby determine the likely origin of these isolates. The alignments were done using the NCBI blast tool optimized for ‘More dissimilar sequences (discontiguous megablast)’ [23].

### Library preparation and genome sequencing

High-molecular-weight DNA from all three isolates (meC0280, meC0281 and meC0467) was extracted using Qiagen Genomic Tip/100 G Kit (Qiagen) according to the manufacturer´s instructions. An SMRTbell template library was prepared according to the instructions from Pacific Biosciences following the Procedure and Checklist – Greater than 10 kb Template Preparation.

Briefly, for preparation of 10 kb libraries, 8 µg genomic DNA was sheared using g-tubes (Covaris) according to the manufacturer’s instructions. DNA was end-repaired and ligated overnight to hairpin adapters applying components from the DNA/Polymerase Binding Kit P6 (Pacific Biosciences). Reactions were carried out according to the manufacturer’s instructions. BluePippin Size-Selection to 4 kb was performed according to the manufacturer’s instructions (Sage Science). Conditions for annealing of sequencing primers and binding of polymerase to purified SMRTbell template were assessed with the calculator in RS Remote (Pacific Biosciences). SMRT sequencing was carried out on the PacBio *RSII* (Pacific Biosciences). In total one SMRT cell per strain was sequenced. In parallel, short-read sequencing was performed from the same DNA on a MiSeq (llumina).

### Genome assembly and bioinformatics analysis

The generated SMRT cell data were assembled using the ‘RS_HGAP_Assembly.3’ protocol included in SMRT Portal version 2.3.0 using default parameters. For PacBio long-read assemblies, 60841 post-filtered reads with an average read length of 13024 bp were used for strain meC0280, 90268 post-filtered reads with an average read length of 12937 bp were used for strain meC0281, and 59866 post-filtered reads with an average read length of 14066 bp were used for strain meC0467. Chromosomal contigs were trimmed, circularized and adjusted to *dnaA* (chromosomal replication initiation protein DnaA) as the first gene. Extrachromosomal elements were trimmed and circularized. The validity of the assembly was checked using the ‘RS_Bridgemapper.1’ protocol included in SMRT Portal version 2.3.0. Finally, each genome was error-corrected against InDel errors by a mapping of generated Illumina reads onto the respective PacBio genome using the Burrows–Wheeler Alignment tool (BWA) [24] with subsequent variant and consensus calling using VarScan 2 [25]. A consensus concordance of QV60 could be confirmed for each of the genomes. Finally, automated genome annotation was performed using Prokka 1.8 [26].

### Phylogenetic analysis

The three genomes generated in this study (meC0280, meC0281 and meC0467), 20 *C *. *coli* clade 1 genomes, one *C. coli* clade 3 genome and four *C. jejuni* genomes were analysed using the Parsnp application. The phylogenetic tree was midpoint-rooted and visualized using Figtree (http://tree.bio.ed.ac.uk/software/figtree/).

### Sequence analysis

For identification of methylated bases and modification motifs, the RS_Modification_and_Motif_Analysis.1 protocol within SMRT Portal version 2.3.0 was used with standard parameters on the basis of the assembled genomes of strains meC0280, meC0281 and meC0467. Putative restriction modification systems have been identified using the Restriction-ModificationFinder-1.0 server (available at https://cge.cbs.dtu.dk/services/) based on the Restriction Enzyme database (REBASE, www.rebase.neb.com) [56]. The consensus sequences were illustrated as sequence logos obtained by the WebLogo 2.82 server (http://weblogo.berkeley.edu/).

### Smash++ analysis

To analyse the composition of the putative hybrid strains meC0280, meC0281 and meC0467, we used the novel software tool Smash++ [27]. To this end, we divided these genomes into non-overlapping segments of 1000 bp each and used Smash++ to match them against the genomes of *C. jejuni* NCTC 11168 and *C. coli* BfR-CA-9557. The aim was to identify 1,000 bp genome segments that are either more likely to be *C. coli *, more likely to be *C. jejuni *, or almost equally likely to be *C. coli* or *C. jejuni *.

For the matching procedure, Smash++ was run with filter size of 100, sampling steps of 180 and compression level of 3. As output, position, size and relative redundancy of similar regions in each 1000 bp genome segment of hybrid strains and *C. coli* or *C. jejuni* homology were obtained. To measure the similarity, we converted relative redundancy (*RR*), which is a dissimilarity measure with values between 0 and 2 [27], to a similarity measure 1−RR/2, with values between 0 and 1, and used it for the calculations explained in the following by an example. Consider a segment x in strain meC0280 — with 1000 bp length — is similar to a segment *y* in the *C. coli* genome — again with 1000 bp length — and the relative redundancy of x rather than *y* is 1.4, i.e. RR*_x:y_=*1.4, and also RR*_x:y_=*1.6. We have Sim*_x:y_* =1−RR*_x:y_*/2=0.3 and Sim*_x:y_* =1−RR*_x:y_*/2=0.2, meaning that 0.3 of *x* is similar to 0.2 of *y*. The total similarity is, then, calculated as

$$\frac{Sim_{x:y}\left|x\right|+Sim_{y:x}\left|y\right|}{\left|x\right|+\left|y\right|}=\frac{0.3*1000+0.2*1000}{1000+1000}=0.25,$$

in which |x| and |y| denote the lengths of x and y , respectively.

The genome segments with a larger *C. jejuni* identity were then blasted against the genome of *C. jejuni* NCTC 11168 using Geneious Prime 2019.3.2 (https://www.geneious.com), in order to identify the genes that potentially integrated into the genome of the hybrid strains.

### Phenotypic assays

In addition to the genomic analyses, the three hybrid strains (meC0280, mec0281 and meC0467) were phenotyped in comparison to *C. coli* (BfR-CA-9557 and RM 2228) and *C. jejuni* (NCTC 11168 and 81116=NCTC 11828). Growth curves, motility, eukaryotic cell adhesion, eukaryotic cell invasion, autoagglutination, biofilm formation and water survival were studied comparatively (see Data S3, available in the online version of this article).

## Results and discussion

### Species confirmation and MLST

**Fig. 1.**
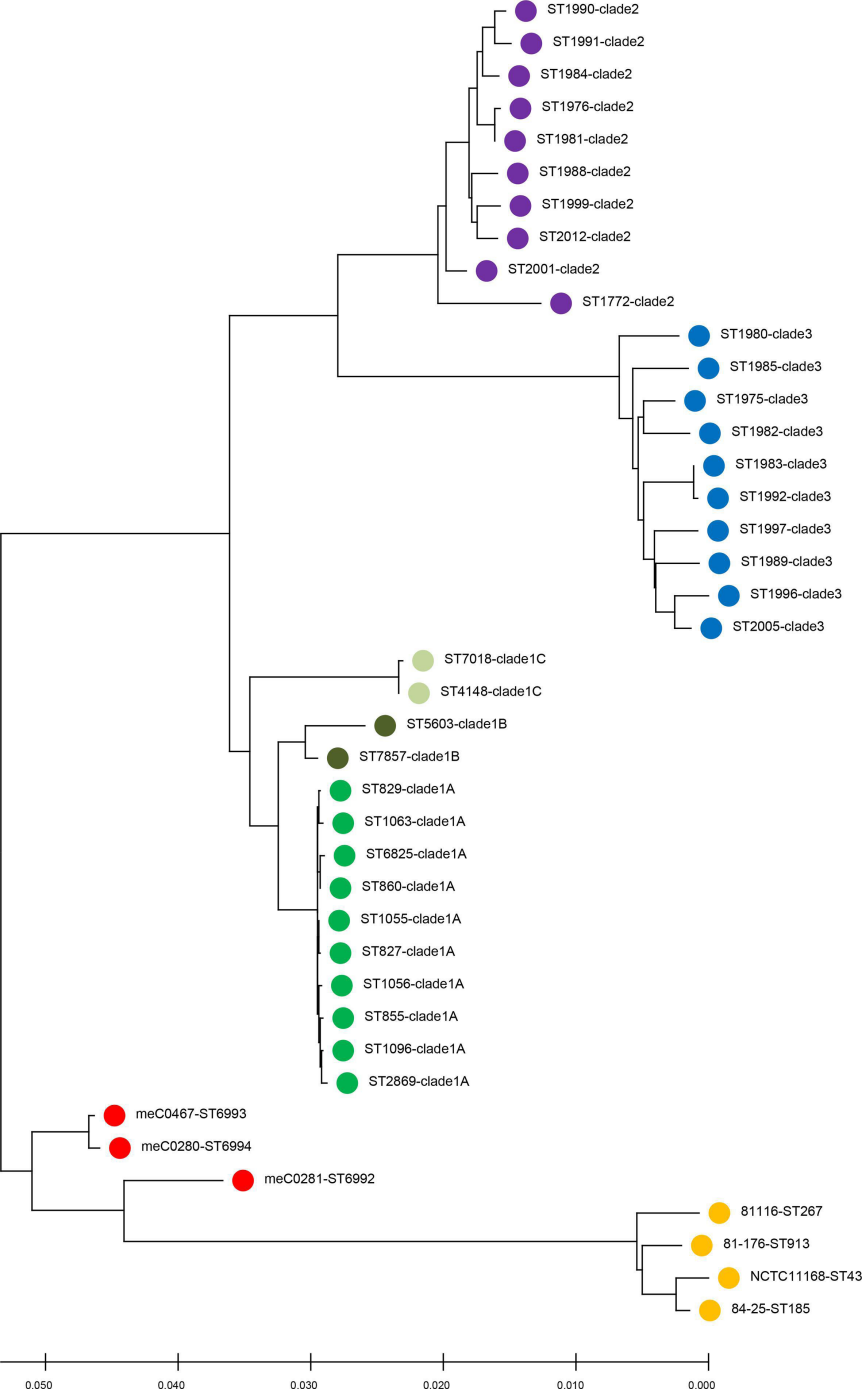
MLST-based phylogenetic tree of *C. coli* and *C. jejuni *. MLST-based dendrogram including sequences of 41 strains places the three hybrid strains (red) between *C. jejuni* (yellow) and *C. coli* (clades 1A green, 1B dark green, 1C pale green, clade 2 purple, clade 3 blue).

Species identification of the three hybrid strains as *C. coli* was performed using the MALDI Biotyper system (Bruker Daltonics). Results with MALDI Biotyper identification score values ≥2.000 were considered correct. Additionally multiplex PCR was used to discriminate between *C. jejuni* and *C. coli* [28]. MLST of the three presumptive *C. coli* isolates meC0280, meC0281 and meC0467 resulted in three, at this time novel, sequence types (ST-6992, 6993 and 6994). However, in the MLST-based dendrogram (neighbour-joining method), the three isolates do not arrange with either of the three *C. coli* clades described previously; instead they branch off at the base of the *C. jejuni* clade (Fig. 1). Due to this unique MLST-based phylogeny, the complete genomes of these three isolates were sequenced.

### SMRT sequencing and annotation

The genome of meC0280 consists of a circular chromosome of 1723814 bp. It contains 1717 predicted coding sequences (CDSs) with a coding density of 0.996 genes per kb and an average gene size of 923 bp. It includes nine rRNAs and 44 tRNAs. The G+C content is 31.37%.

The genome of meC0281 consists of a circular chromosome of 1742400 bp and two extrachromosomal elements (ECEs) of 138548 and 30719 bp. The chromosome contains 1756 CDSs with a coding density of 1.007 genes per kb and an average gene size of 914 bp. It includes nine rRNAs and 44 tRNAs. The G+C content is 31.36%. The larger ECE with episomal character encodes 153 CDSs with a coding density of 1.104 genes per kb and an average gene size of 797 bp. Its G+C content is 26.88%. The smaller ECE with episomal character encodes 39 CDSs with a coding density of 1.269 genes per kb and an average gene size of 718 bp. Its G+C content is 29.25%.

The genome of meC0467 consists of a circular chromosome of 1661120 bp. It contains 1623 predicted CDSs with a coding density of 0.977 genes per kb and an average gene size of 936 bp. It includes nine rRNAs and 44 tRNAs. The G+C content is 31.50%.

### *C. coli-* and *C. jejuni-*specific genes

Méric *et al*. identified 21 genes specific for *C. coli* and 27 genes specific for *C. jejuni* based on analysis of 192 genomes that can be used to differentiate the individual microbial species [22]. blast searches of these 48 genes within the genome sequences of the (at this point of the study ‘potential’) hybrid strains showed that the hybrid strain genomes aligned to all the 21 *C *. *coli *-specific gene markers (100%) with percentage identities averaging 93% (ranging between 84 and 98%) and query coverages averaging 100% (ranging between 98 and 100%). In contrast, the hybrid strain genomes aligned to only eight or nine of the 27 *C *. *jejuni *-specific gene markers (37%) with percentage identities of the aligned gene markers averaging 82% (ranging between 72 and 98%) and query coverages averaging 62% (ranging between 30 and 100%). These results indicate that the hybrid strains are *C. coli* isolates that have genes from *C. jejuni* integrated into their genome.

### Core genome-based dendrogram

**Fig. 2.**
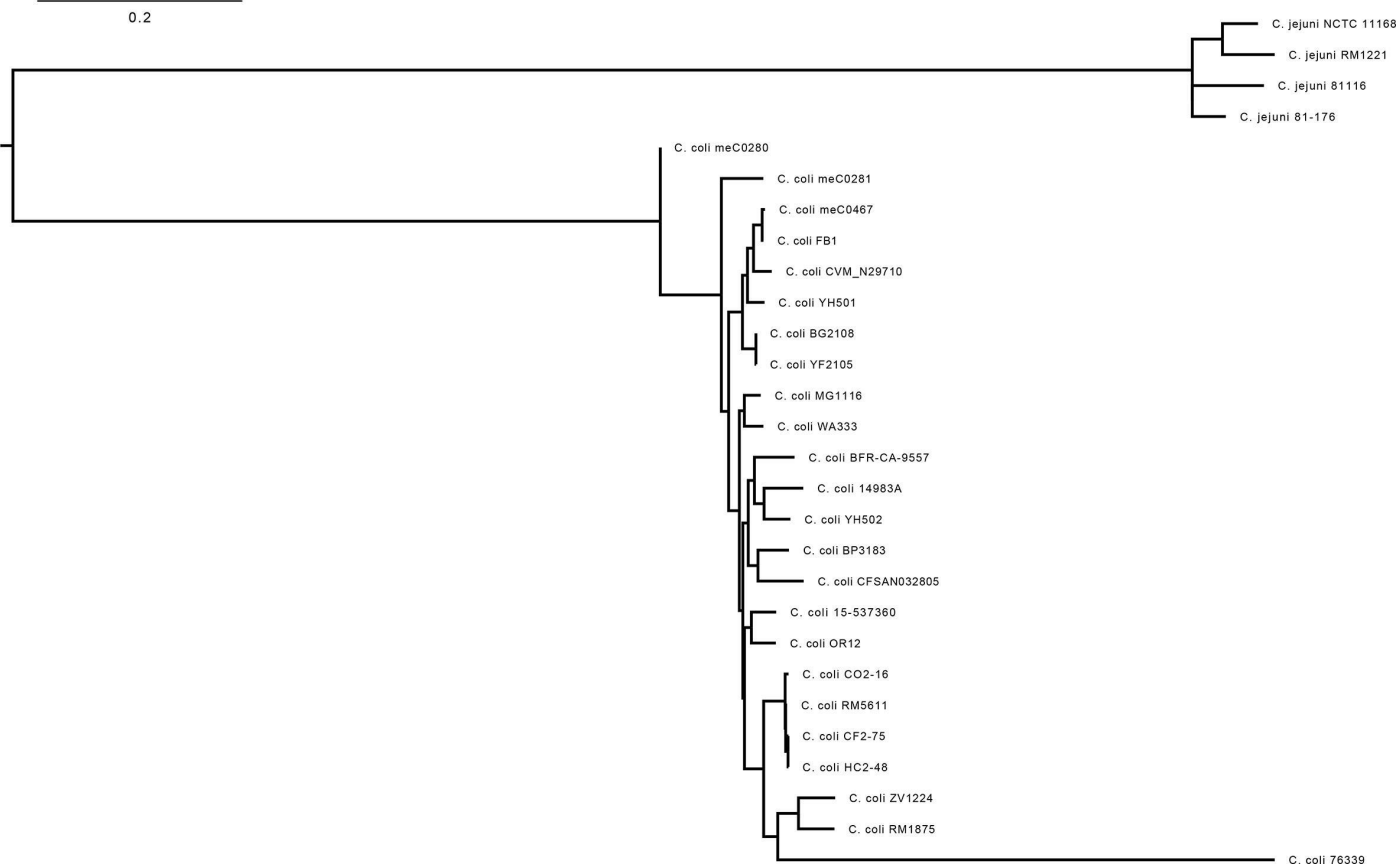
Core genome analysis of representative genomes of *C. coli* and *C. jejuni* by Parsnp. The core genome-based analysis of 20 *C *. *coli* and four *C *. *jejuni* genomes identifies the isolates meC0280, meC0281 and meC0467 as *C. coli* clade 1 representatives.

The core genome-based phylogeny also clearly shows that the isolates meC0280, meC0281 and mec0467 are essentially *C. coli* isolates of clade 1. Nevertheless, the evolutionary distance clearly shows that these three isolates, in particular meC0280, are much more closely related to the *C. jejuni* isolates than, for example, *C. coli* BfR-CA-9557 (Fig. 2).

### Smash++ analysis

**Fig. 3.**
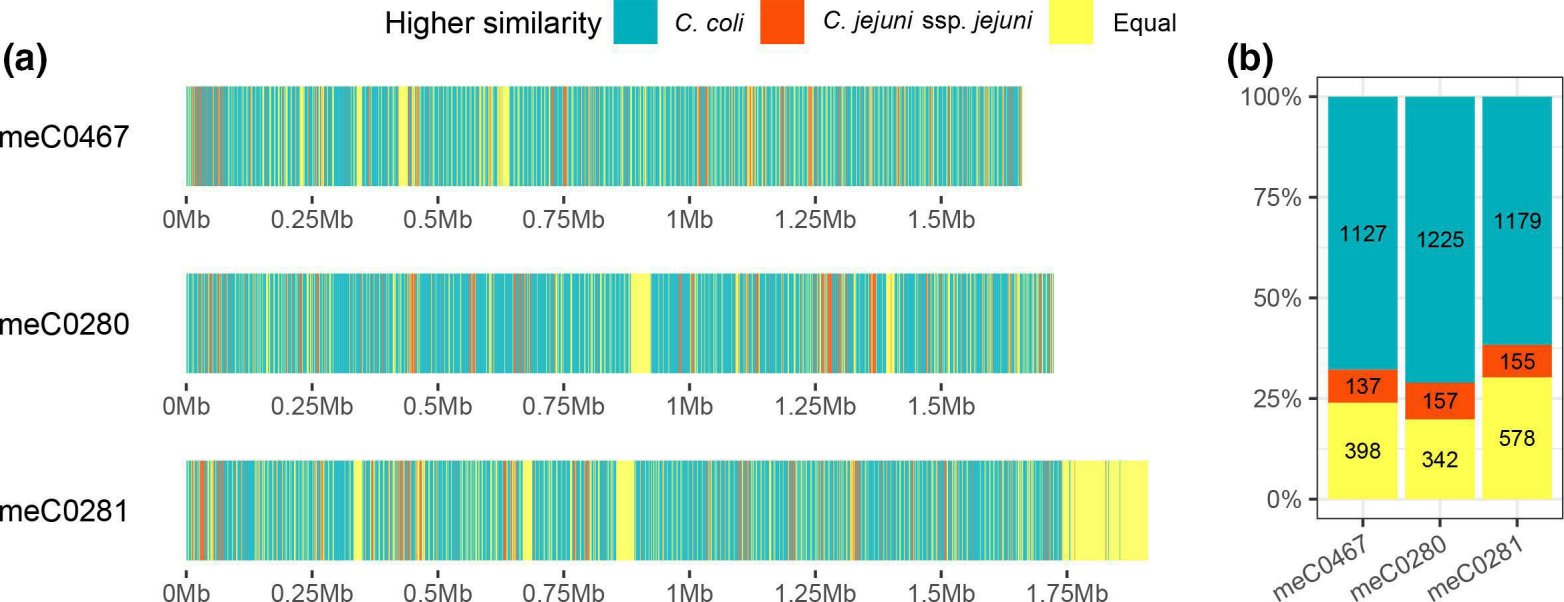
Similarity analysis of hybrid strains to *C. coli* or *C. jejuni* references. Genome segments of 1000 bp (Smash++ algorithm) are matched to the genomes of *C. coli* BfR-CA-9557 and *C. jejuni* NCTC 11168. (a) Segments that are more similar to *C. coli* are shown in turquoise in the track diagram in their position in the genome, segments that are more similar to *C. jejuni* are shown in red, and equal segments in are shown in yellow. (b) Condensed representations of the Smash++ segments according to their similarity to *C. coli* and *C. jejuni* in absolute numbers and in per cent.

**Fig. 4.**
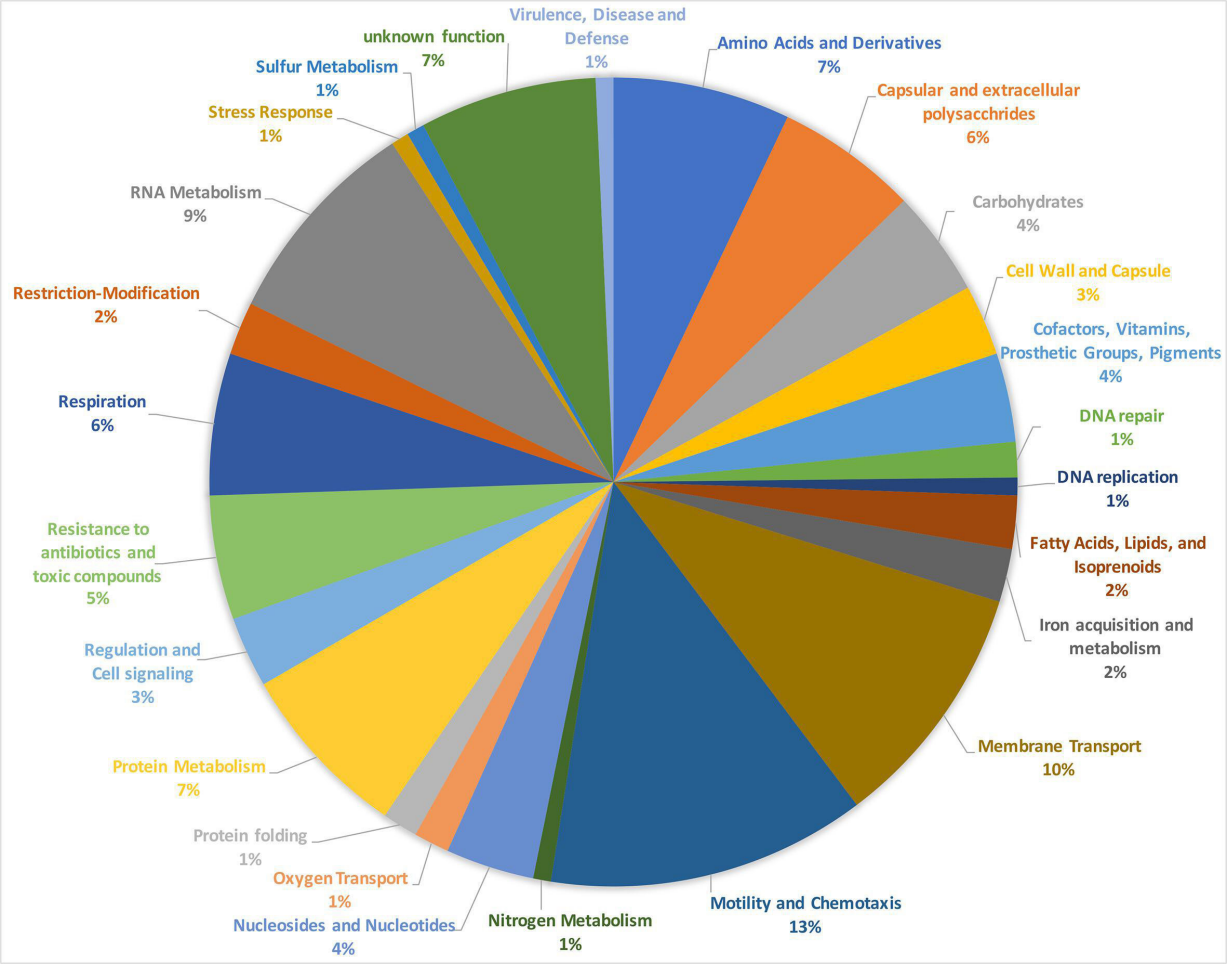
Functional subsystems identified in 141 genes of the hybrid strain meC0280 originating from *C. jejuni *. This pie chart illustrates the functional subsystems of the 141 genes of the hybrid strain meC0280, which presumably originated from *C. jejuni *. In particular, the two categories motility and chemotaxis as well as membrane transport dominate. Isolate meC0280 was chosen as an example because with 141 genes of putative *C. jejuni* origin compared to the other two strains, it contains the most genes of putative *C. jejuni* origin.

The Smash++ algorithm fragmented the genomes of meC0280, meC0281 and meC0467 into 1724, 1912 and 1662 1000 bp fragments. Of these, only 157 (9.11%), 155 (8.11%) and 137 (8.24%), respectively, showed a higher similarity to *C. jejuni *, while 1225 (71.06%), 1179 (61.66%) and 1127 (67.81%) were assigned to *C. coli *. No clear assignment was possible for 342 (19.84%), 578 (30.23%) and 398 (23.95%) fragments (Fig. 3b). Segments with a higher similarity to the *C. jejuni* genome are scattered over the entire genome of the hybrid strains and do not form a coherent cluster (Fig. 3a). The increased number of genome parts of mecC0281, which can equally be assigned to both *C. jejuni* and *C. coli *, is increased due to the presence of two ECEs in this isolate. Again, the hybrid strains are rather *C. coli* isolates of clade 1, containing at least 8.11–9.11% *C *. *jejuni* DNA. blast analysis of these ‘Smash++’-fragments revealed 141, 136 and 124 genes, respectively, which probably originate from *C. jejuni* (see Data S1, Tables 1–3). Note: the Smash++ algorithm analysed 1000 bp segments for their similarity to comparable segments in *C. coli* and *C. jejuni *. Due to the fact that some genes are also significantly larger than 1000 bp, sometimes up to 2000 bp or occasionally 3000 bp, the number of genes is reduced compared to the segments. These genes encode proteins involved in various functions (illustrated in Fig. 4 using the example of meC0280). What stands out is that many of the genes taken up from *C. jejuni* play a role in chemotaxis and motility (e.g. *fliD*), membrane transport, regulation and cell signalling, and resistance to antibiotics and toxic compounds. As compared to the whole genome of the clade 1 *C *. *coli* isolate BfR-CA-9557, the chemotaxis and motility genes taken up from *C. jejuni* in meC0281 account for 13% instead of the expected 5.7%, membrane transport genes account for 10% instead of 3.3%, genes for regulation and cell signalling account for 3% instead of 1.1%, and resistance to antibiotics or toxic compounds genes account for 5 % instead of 1.9%.

**Table 1.** Alignments to 21 *C *. *coli *- and 27 *C *. *jejuni *-specific gene markers

Gene identifier	Species association of gene identifier	meC0280		meC0281		meC0467	
		Identity (%)	Coverage (%)	Identity (%)	Coverage (%)	Identity (%)	Coverage (%)
Cc76339_00005 c	* C. coli *	89	100	89	100	89	100
Cc76339_01340	* C. coli *	92	99	91	99	91	99
Cc76339_01460 c	* C. coli *	96	100	96	100	96	100
Cc76339_01470 c	* C. coli *	98	100	98	100	98	100
Cc76339_01480 c	* C. coli *	96	100	97	100	97	100
Cc76339_01490 c	* C. coli *	94	100	94	100	94	100
Cc76339_01750	* C. coli *	95	100	95	100	95	100
Cc76339_02240	* C. coli *	96	100	96	100	95	100
Cc76339_03250	* C. coli *	98	100	97	100	98	100
Cc76339_04670	* C. coli *	92	100	92	100	92	100
Cc76339_09670	* C. coli *	94	100	94	100	94	100
Cc76339_10710	* C. coli *	97	100	98	100	98	100
Cc76339_10950	* C. coli *	93	100	92	100	92	100
Cc76339_11130	* C. coli *	94	100	96	100	97	100
Cc76339_11470	* C. coli *	85	100	85	100	81	100
Cc76339_11500 c	* C. coli *	84	98	84	98	84	98
Cc76339_12660 c	* C. coli *	94	100	94	100	94	100
Cc76339_12670	* C. coli *	92	100	92	100	92	100
Cc76339_12940	* C. coli *	93	98	93	98	93	98
Cc76339_15800	* C. coli *	96	100	97	100	96	100
Cc76339_15900 c	* C. coli *	94	100	94	100	95	100
11168_Cj0011c	* C. jejuni* ssp. *jejuni*	–	–	–	–	–	–
11168_Cj0090	* C. jejuni* ssp. *jejuni*	–	–	–	–	–	–
11168_Cj0135	* C. jejuni* ssp. *jejuni*	72	97	72	97	72	97
11168_Cj0186c	* C. jejuni* ssp. *jejuni*	67	92	67	92	67	92
11168_Cj0327	* C. jejuni* ssp. *jejuni*	–	–	–	–	–	–
11168_Cj0339	* C. jejuni* ssp. *jejuni*	–	–	–	–	–	–
11168_Cj0340	* C. jejuni* ssp. *jejuni*	–	–	–	–	–	–
11168_Cj0414	* C. jejuni* ssp. *jejuni*	–	–	–	–	–	–
11168_Cj0454c	* C. jejuni* ssp. *jejuni*	70	99	70	100	–	–
11168_Cj0494	* C. jejuni* ssp. *jejuni*	–	–	–	–	–	–
11168_Cj0873c	* C. jejuni* ssp. *jejuni*	–	–	–	–	–	–
11168_Cj0900c	* C. jejuni* ssp. *jejuni*	–	–	–	–	–	–
11168_Cj1021c	* C. jejuni* ssp. *jejuni*	72	57	72	57	72	57
11168_Cj1036c	* C. jejuni* ssp. *jejuni*	88	61	89	61	89	61
11168_Cj1060c	* C. jejuni* ssp. *jejuni*	–	–	77	42	74	42
11168_Cj1162c	* C. jejuni* ssp. *jejuni*	–	–	–	–	–	–
11168_Cj1666c	* C. jejuni* ssp. *jejuni*	–	–	–	–	–	–
11168_Cj1714	* C. jejuni* ssp. *jejuni*	–	–	–	–	–	–
11168_ctsT	* C. jejuni* ssp. *jejuni*	96	60	96	60	98	60
11168_kdpD	* C. jejuni* ssp. *jejuni*	95	48	95	48	95	48
11168_tonB2	* C. jejuni* ssp. *jejuni*	97	30	97	30	97	30
Cj81116_1523	* C. jejuni* ssp. *jejuni*	–	–	–	–	–	–
Cj_81–176_1820 (CJ81176_0363)*	* C. jejuni* ssp. *jejuni*	–	–		–	–	–
Cj_81–176_6530 (CJ81176_1246)*	* C. jejuni* ssp. *jejuni*	–	–	–	–	–	–
Cj_81–176_8530 (FORC46_1556)*	* C. jejuni* ssp. *jejuni*	–	–	–	–	–	–
Cj_81–176_RS08535 (CJ81176_1613)*	* C. jejuni* ssp. *jejuni*	–	–	–	–	–	–
Cjdoylei_26997_0913	* C. jejuni* ssp. *doylei*	–	–	–	–	–	–

Gene identifier in the re-annotated genome.

**Table 2.** Putative *C. coli* meC0280 restriction modification systems

ORF (REBASE)	Strand	Position in genome	Description	Type/subunit	Predicted recognition sequence
2066	+	1511587–1513905	Cco280IP Type I restriction-modification system, restriction subunit R (EC 3.1.21.3)	I/R	CRAANNNNNNNRTAG
2069	+	1516316–1517563	S.Cco280I Type I restriction-modification system, specificity subunit S (EC 3.1.21.3)	I/S	CRAANNNNNNNRTAG
2071	+	1518755–1520242	M.Cco280I Type I restriction-modification system, DNA-methyltransferase subunit M (EC 2.1.1.72)	I/M	CRAANNNNNNNRTAG
50	+	46204–48537	Cco280ORFAAP putative type IIS restriction/modification enzyme	II/RM	
51	+	48507–050015	Cco280ORFABP putative type IIS restriction/modification enzyme	II/RM	
1206	−	889311–890063	M.Cco280ORFBP adenine-specific methyltransferase (EC 2.1.1.72)	II/M	
1377	−	997265–1000351	Cco280IV / Cco280ORFCP adenine-specific DNA methyltransferase	II/RM	GGGTDA
1382	−	1002805–1006899	Cco280III Type II restriction-modification system, DNA-methyltransferase subunit M (EC 2.1.1.72)/Type II restriction-modification system, restriction subunit R (EC 3.1.21.3)	II/RM	GAGNNNNNRTG
2185	+	1599644–1600738	M.Cco280II DNA modification methylase (adenine-specific methyltransferase) (EC 2.1.1.72)	II/M	RAATTY
2238	−	1633326–1634678	Cco280McrCP McrBC restriction endonuclease system, McrB subunit, putative	IV/R	
2239	−	1634665–1636548	Cco280McrBP McrBC restriction endonuclease system, McrB subunit, putative	IV/R	

**Table 3.** Methylation motifs of *C. coli* meC0280

No.	Motif	Modified position	Modification yype	% Motifs detected	No. of motifs detected	No. of motifs in genome	Mean modification QV*	Mean motif coverage	Partner motif
A1	CTAYNNNNNNNTTYG	3	^m6^A	100	284	284	253.97	180.29	CRAANNNNNNNRTAG
A2	CRAANNNNNNNRTAG	4	^m6^A	100	284	284	231.44	177.57	CTAYNNNNNNNTTYG
B1	CAYNNNNNCTC	2	^m6^A	100	797	797	244.40	181.57	GAGNNNNNRTG
B2	GAGNNNNNRTG	2	^m6^A	99.62	794	797	232.87	179.81	CAYNNNNNCTC
C	RAATTY	3	^m6^A	99.74	28 407	28 482	258.96	177.96	RAATTY
D	GGGTDA	6	^m6^A	99.68	1573	1578	251.71	178.39	

The PacBio SMRT Analysis software was used to identify methylation motifs. The sequence consensus of the motif is shown in column 2 whereas the reverse complement of the motif, the partner motif, is shown in column 10. IUPAC ambiguity codes represent non-uniform positions. The position of the modified base within the motif and the type of methylation are indicated in columns 3 and 4. Column 5 denotes the percentage of a motif’s occurrences in the genome (column 7) for which a methylation has been detected (column 6). Column 8 lists the average modification quality (in Phred Q-scores) and column 9 the average coverage of motifs detected as modified.

*QV, quality value

**Fig. 5.**
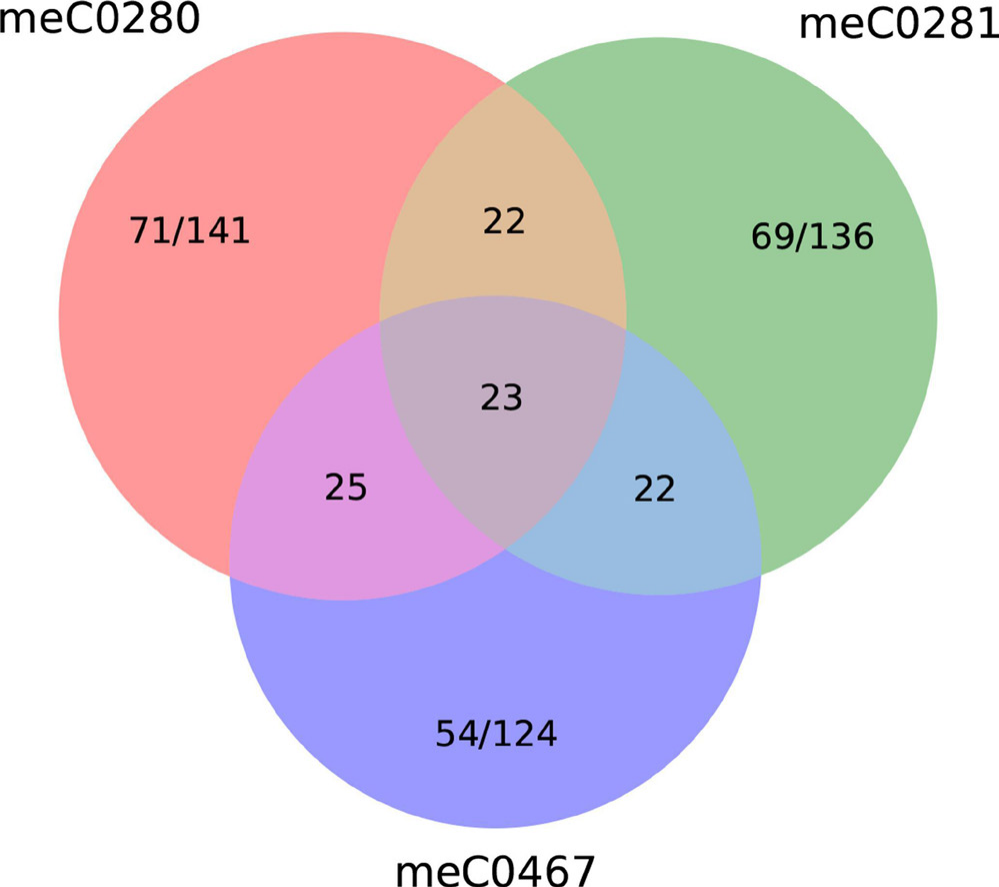
Venn diagram to illustrate the common proportions of genes putatively derived from *C. jejuni* and taken up by the hybrid strains. The three hybrid strains have 141, 136 or 124 genes integrated into their genome, which are presumably derived from *C. jejuni *. These genes are relatively different between the individual hybrid isolates although individual functional groups are clearly overrepresented. Just 23 genes originating from *C. jejuni* are found in all three hybrid genomes.

This suggests that the uptake of genetic material from *C. jejuni* is selective and not merely random and lineages with these genes seem to have acquired selective advantages. Most of these genes do not belong to the core genome and are absent in at least one of the three genomes. Only 23 genes with a probable *C. jejuni* origin are present in all three genomes (Fig. 5, Data S1, Tables S4–S10). These shared genes will be discussed in more detail.

**Fig. 6.**
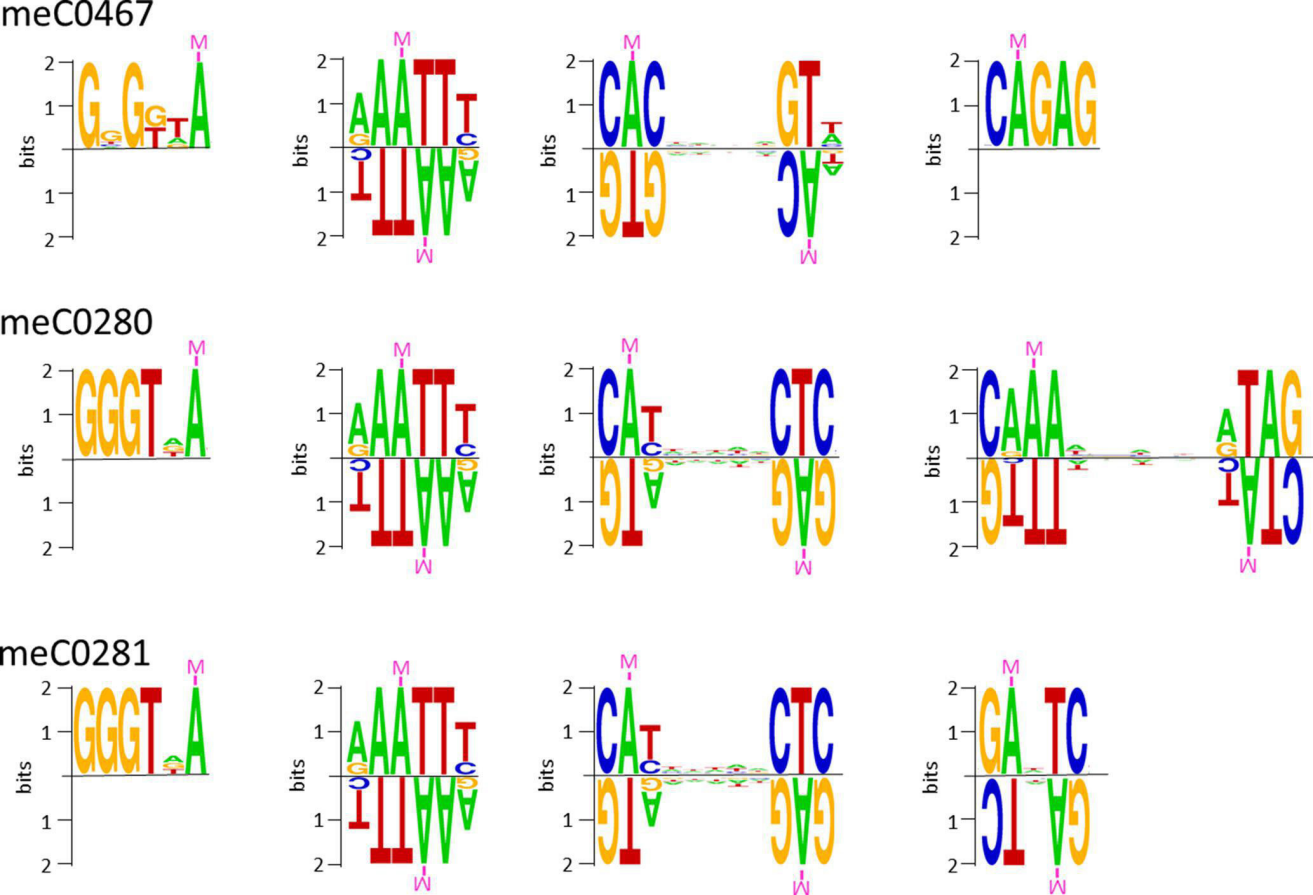
Methylation motifs in the three hybrid strains. Sequence logos of four methylation motifs detected in the hybrid isolate meC0280. The two motifs in A1 and in A2 as well as B1 and B2 are partner motifs that are methylated at both strands. All motifs are recognized by N-6 adenine-specific methyltransferases. The height of each stack indicates the degree of conservation (bits). The height of the letters represents the relative frequency of the base. The asterisk under a particular letter indicates the modified/methylated base.

One of the shared genes encodes a fused type II RM system consisting of a DNA-methyltransferase M and a restriction endonuclease R. It is annotated as Cco280III in meC0280 (annotation according to REBASE; ORF 1382; position in genome 1002805–1006899; locus tag: DSM101856_01047; Table 2) and recognizes the motif CAYNNNNNCTC and the corresponding partner motif GAGNNNNNRTG. (Table 3, Fig. 6). In *C. coli* meC0281 and meC0467 homologous genes encoding a fused type II RM system, Cco104626I and Cco104627ORFBP, are present. The difference between these two is that REBASE predicts the same recognition motif for Cco104626I (meC0281, ORF 1065, position in genome 771536–775630; locus tag: DSM104626_00799) as in meC0280, whereas CACNNNNNGT is the predicted recognition motif of Cco104627ORFBP (meC0467, ORF 977, position in genome 727644–731651; locus tag: DSM104627_00741, Data S2). Canonical type II RM systems are typically composed of two homodimeric R (restriction) subunits and a separate M (methylation) protein. Both act independently and recognize the same methylated palindromic DNA motif [29]. However, in the case of Cco280III and the homologues in the other two hybrid strains, the RM system consists of a single protein, in which the restriction subunit R is fused with the DNA methyltransferase subunit M. This RM system is conserved in the context of this analysis. For the type II RM system encoded by *cj1051c* in *C. jejuni* it could be shown that it reduces the transformation efficiency for plasmids [30]. Additionally, Beauchamp and co-workers identified the so-called *Campylobacter* transformation system methyltransferase (CtsM) [31], which is also present in *C. coli* BfR-CA-9557 (clade 1) [32]. CtsM recognizes the RA^m6^ATTY motif, and DNA originating from a *ctsM* knockout mutant transforms *C. jejuni* significantly less effectively than DNA derived from *ctsM*-expressing bacteria [31]. Thus, Cco280III and homologues in other hybrid strains may bear the function to prevent disadvantageous changes in the chromosome but promote uptake of advantageous DNA, e.g. from *C. jejuni* strains. From this point of view, DNA methylation could be a key factor in the emergence of *C. coli–C. jejuni* hybrid strains.

Another gene present in all three hybrid genomes is *fucP*, which encodes a fucose permease that enables a subgroup of *C. jejuni* isolates to metabolize l-fucose [33]. The presence of *fucP* has been associated with livestock (bovine) habitats of *C. jejuni *, and it has also been deduced that fucose permease is an important prerequisite for residing in the mucosal layer [34].

Among the genes common to all three hybrid strains with putative *C. jejuni* origin are the three multidrug efflux RND transporter permease subunit genes *cmeB*, *cmeE* and *cmeF*. Both multidrug efflux pumps, CmeABC and CmeDEF, comprise a periplasmic fusion protein, CmeA or CmeD, an inner membrane efflux transporter, CmeB or CmeE, and an outer membrane protein, CmeC or CmeF. In particular, the genes of the two periplasmic fusion proteins, CmeA or CmeD, seem to have been taken up from *C. jejuni* and integrated into the genome of the hybrid strains. CmeABC and CmeDEF are involved in bile resistance [35]. Bile acids, especially deoxycholic acid, chenodeoxy-cholic acid and glycocholic acid, have been demonstrated to induce the expression of *cmeA*, *cmeB*, *cmeC* and *cmeE* [36]. The uptake of these genes from *C. jejuni* might be an adaptation to an intestinal bile acid-containing habitat. Bile acids in turn cause oxidative stress in the bacterial cells, so that adaptations to oxidative stress must also be understood in the context of adaptation to an intestinal habitat containing bile acids.

Another common gene is *mutS2*. It encodes the recombination inhibitory protein MutS2. MutS2 has been shown to play an important role in repairing oxidative DNA damage and it has anti-recombination activity in *Helicobacter pylori *. MutS2 maintains the integrity of the genome by suppressing homologous and homoeologous DNA recombination [37, 38].

The gene encoding elongation factor 4 *lepA* is a further common gene in the three hybrid strains. EF4/LepA binds to the post-translocation as well as to the pretranslocation ribosomal complexes and regulates the elongation cycle and thus protein synthesis especially under specific stress conditions [39]. It has been demonstrated that EF4/LepA is retained at the inner cell membrane of *Escherichia coli* and released into the cytoplasm at high intracellular ionic strength or low temperature [40]. EF4/LepA protects cells from moderate stress by allowing stress-paused translation to resume, but at high stress levels it acts in a mechanism that accelerates cell death by accumulation of reactive oxygen species [41]. Other common proteins of *C. jejuni* origin involved in amino acid metabolism and transcription are pantoate–beta-alanine ligase, chorismate-binding protein, cation:dicarboxylase symporter family transporter, and DNA-directed RNA polymerase subunit beta RpoB.

As already observed in the genome of clade 1 isolate BfR-CA-9557, three homologues to the iron transport protein TonB are present in all three hybrid isolates [32]. The TonB-dependent ferri-enterochelin receptor gene *cfrA* (*cj0755*) is also one of the common genes apparently taken up from *C. jejuni *. The outer-membrane receptor protein CfrA binds ferri-enterochelin and initiates the uptake via the inner-membrane ABC transporter system CeuBCDE [42]. The presence of *cfrA* (*cj0755*) and other putative *C. jejuni* iron-uptake systems was associated with better livestock adaptation [34].

The remaining common genes that were apparently taken up from *C. jejuni* can be divided into two categories. The ‘respiration and phosphate metabolism’ category includes the genes for formate dehydrogenase subunit alpha, FAD-binding oxidoreductase, phosphate ABC transporter ATP-binding protein, and esterase-like activity of phytase family protein. The category ‘DNA synthesis and replication’ includes the genes encoding the type I DNA topoisomerase and the ribonucleoside-diphosphate reductase subunit alpha.

### Phenotypic assays

In addition to genome analysis, the hybrid strains were examined for growth behaviour, motility, invasion and adhesion ability, biofilm formation and autoagglutination in comparison to two *C. jejuni* (NCTC 11168 and 81116) and two *C. coli* (BfR-CA-9557 and RM 2228) reference strains. In phenotypic testing, differences of varying significance were observed for some of the parameters both within the hybrid strains and in comparison to the *C. jejuni* and *C. coli* reference strains (see Data S3).

Statistical analysis revealed that strain meC0467 was significantly more motile than strain meC0280 (*P*<0.05). Strain meC0467 demonstrated a significantly (*P*<0.05) lower adhesion to Caco-2 cells compared to the two other hybrid strains. Also, strain meC0281 showed a significantly higher tendency to autoagglutination than the strains meC0280 and meC0467 (*P*<0.05). Finally, it was observed that strain meC0467 formed significantly more biofilm than meC0280 and meC0281 (*P*<0.001). No significant differences were detected between the hybrid strains with respect to water survival and Caco-2 cell invasion.

In the comparison of growth dynamics, meC0280 in particular stands out, which grows about twice as fast as NCTC 11168, 81116, BfR-CA-9557, meC0281 and meC0467. Only *C. coli* RM 2228 also demonstrates an average generation time in the range of 120–140 min. In relation to the reference strains, significant differences between two of the hybrid isolates and the *C. coli* and *C. jejuni* reference strains were also observed in their motility. Strain meC0280 was significantly (*P*<0.05) less motile compared to *C. jejuni* NCTC 11168 as well as 81116, but meC0467 was significantly more motile than *C. coli* BfR-CA-9557. Differences in adhesion to Caco-2 cells in the sense of stronger or weaker host cell adhesion were likewise observed within the reference strains, so that general tendencies were not discernible. No significant differences were observed in Caco-2 cell invasiveness.

Concerning the above-mentioned strong differences in the tendency to autoagglutination within the hybrid strains, which is greater than that between the reference strains, no general statement can be made regarding the autoagglutination comparison with the reference strains. Regarding biofilm formation, strain meC0475 stood out, showing significantly (*P*<0.05) higher biofilm formation in our experiment compared to all other isolates tested except NCTC 11168. The *C. jejuni* strains survived statistically better in water than the *C. coli* strains (*P*<0.05). Here, two of the hybrid strains, meC0281 and meC0467, showed a statistically significant difference to the *C. jejuni* strains (*P*<0.05) and hence a greater similarity to the *C. coli* strains (*P*>0.05), which is in accordance with the higher *C. coli* genome content of the hybrid strains.

### Conclusions

This study on three genome-sequenced *C. coli–C. jejuni* hybrid strains shows that the process of despeciation of *C. coli* and *C. jejuni* continues to progress. *C. coli* isolates from clade 1 were already known to have incorporated genetic material from *C. jejuni *. In some of the isolates described here as hybrid strains, the *C. jejuni* genome proportion is significantly higher (approx. 8–9 %) than in non-hybrid clade 1 *C *. *coli* isolates. Clade 2 and clade 3 *C *. *coli* isolates are found almost exclusively in environmental waters. If one understands this as a reference point, then the uptake of *C. jejuni* genetic information occurs mainly in the framework of adaptation to intestinal habitats in livestock and thus to conditions with bile acids and thus oxidative stress.

A type II restriction-modification system (Cco280III in meC0280) which, according to our analyses, itself originally is derived from *C. jejuni* and that recognizes the motif CAYNNNNNCTC/GAGNNNNNRTG, may play a key role in the uptake of *C. jejuni* genetic material. While appropriately methylated DNA fragments are tolerated and consecutively integrated into the chromosome, DNA sequences are degraded without appropriate methylation.

## Supplementary Data

Supplementary material 1
