# Supplementary material for: Genome and Methylome analysis of a phylogenetic novel Campylobacter coli cluster with C. jejuni introgression
Source: Microb Genom. 2021 Oct 18;7(10):000679. doi: 10.1099/mgen.0.000679 (PMC8627207; doi:10.1099/mgen.0.000679)
Supplement: Supplementary material 1 [file mgen-7-0679-s001.pdf]

Table S1: List of the Smash++ 1,000 bp segments, which presumably originate from *C. jejuni* of the isolate *C. coli* meC0280 with the corresponding locus tags of NCTC 11168.

locus_tag	Sequence Name	Minimum	Maximum	Length	Direction	Min (with gaps)	Max (with gaps)	Length (with gaps)	Min (original sequence)	Max (original sequence)	Description	Sequence Length	% Pairwise Identity	Query Coverage
[locus_tag=DQM41_RS00110]	lcl|NZ_LS483362.1_cds_WP_010891825.1_22	16	717	702	reverse	16	717	702	16	717	[protein=RluA family pseudouridine synthase] [protein_id=WP_010891825.1] [location=complement(27402..28304)] [gbkey=CDS]	903	86.30%	70.20%
[locus_tag=DQM41_RS00120]	lcl|NZ_LS483362.1_cds_WP_002865087.1_24	1	564	564	forward	1	564	564	1	564	[protein=ribonucleoside-diphosphate reductase subunit alpha] [protein_id=WP_002865087.1] [location=29726..32095] [gbkey=CDS]	2370	97.90%	56.40%
[locus_tag=DQM41_RS00140]	lcl|NZ_LS483362.1_cds_WP_002853146.1_28	307	1302	996	forward	307	1302	996	307	1302	[gene=recJ] [protein=single-stranded-DNA-specific exonuclease RecJ] [protein_id=WP_002853146.1] [location=36000..37571] [gbkey=CDS]	1572	90.10%	99.60%
[locus_tag=DQM41_RS00180]	lcl|NZ_LS483362.1_cds_31	764	1762	999	forward	764	1762	999	764	1762	[protein=class I SAM-dependent DNA methyltransferase] [pseudo=true] [location=46420..50152] [gbkey=CDS]	3733	93.40%	99.90%
[locus_tag=DQM41_RS00180]	lcl|NZ_LS483362.1_cds_31	1765	2250	486	forward	1765	2250	486	1765	2250	[protein=class I SAM-dependent DNA methyltransferase] [pseudo=true] [location=46420..50152] [gbkey=CDS]	3733	82.70%	48.60%
[locus_tag=DQM41_RS00190]	lcl|NZ_LS483362.1_cds_WP_002866314.1_33	64	690	627	reverse	64	690	627	64	690	[protein=SIMPL domain-containing protein] [protein_id=WP_002866314.1] [location=complement(51963..52664)] [gbkey=CDS]	702	90.40%	62.70%
[locus_tag=DQM41_RS00230]	lcl|NZ_LS483362.1_cds_WP_002805290.1_41	109	774	666	forward	109	774	666	109	774	[protein=flagellar basal body rod modification protein] [protein_id=WP_002805290.1] [location=61339..62223] [gbkey=CDS]	885	88.10%	66.60%
[locus_tag=DQM41_RS00240]	lcl|NZ_LS483362.1_cds_WP_010891832.1_43	607	1440	834	reverse	607	1440	834	607	1440	[protein=DUF342 domain-containing protein] [protein_id=WP_010891832.1] [location=complement(63868..65739)] [gbkey=CDS]	1872	91.10%	83.40%
[locus_tag=DQM41_RS00240]	lcl|NZ_LS483362.1_cds_WP_010891832.1_43	1	606	606	reverse	1	606	606	1	606	[protein=DUF342 domain-containing protein] [protein_id=WP_010891832.1] [location=complement(63868..65739)] [gbkey=CDS]	1872	100.00%	60.60%
[locus_tag=DQM41_RS00245]	lcl|NZ_LS483362.1_cds_WP_025096273.1_44	214	279	66	reverse	214	279	66	214	279	[protein=bacteriohemerythrin] [protein_id=WP_025096273.1] [location=complement(65696..66463)] [gbkey=CDS]	768	40.90%	6.60%
[locus_tag=DQM41_RS00260]	lcl|NZ_LS483362.1_cds_WP_079254168.1_46	1	180	180	forward	1	180	180	1	180	[protein=anion permease] [protein_id=WP_079254168.1] [location=67420..67671] [gbkey=CDS]	252	95.00%	18.00%
[locus_tag=DQM41_RS00305]	lcl|NZ_LS483362.1_cds_WP_002864362.1_55	1	843	843	forward	1	843	843	1	843	[protein=hypothetical protein] [protein_id=WP_002864362.1] [location=72001..72855] [gbkey=CDS]	855	97.50%	84.30%
[locus_tag=DQM41_RS00320]	lcl|NZ_LS483362.1_cds_WP_002851710.1_58	508	978	471	reverse	508	978	471	508	978	[gene=fliM] [protein=flagellar motor switch protein FliM] [protein_id=WP_002851710.1] [location=complement(74327..75406)] [gbkey=CDS]	1080	93.30%	47.10%
[locus_tag=DQM41_RS00615]	lcl|NZ_LS483362.1_cds_WP_002851926.1_117	574	903	330	forward	574	903	330	574	903	[protein=tRNA-dihydrouridine synthase] [protein_id=WP_002851926.1] [location=complement(125499..126425)] [gbkey=CDS]	927	97.30%	33.00%
[locus_tag=DQM41_RS00680]	lcl|NZ_LS483362.1_cds_WP_002864659.1_130	2023	2616	594	reverse	2023	2616	594	2023	2616	[gene=infB] [protein=translation initiation factor IF-2] [protein_id=WP_002864659.1] [location=136852..139467] [gbkey=CDS]	2616	99.50%	59.40%
[locus_tag=DQM41_RS00680]	lcl|NZ_LS483362.1_cds_WP_002864659.1_130	1024	2019	996	reverse	1024	2019	996	1024	2019	[gene=infB] [protein=translation initiation factor IF-2] [protein_id=WP_002864659.1] [location=136852..139467] [gbkey=CDS]	2616	95.90%	99.60%
[locus_tag=DQM41_RS00700]	lcl|NZ_LS483362.1_cds_WP_010891837.1_134	295	660	366	reverse	295	660	366	295	660	[protein=McrC family protein] [protein_id=WP_010891837.1] [location=142629..143960] [gbkey=CDS]	1332	63.50%	36.60%
[locus_tag=DQM41_RS00890]	lcl|NZ_LS483362.1_cds_WP_002864195.1_172	1	723	723	forward	1	723	723	1	723	[protein=aspartate aminotransferase family protein] [protein_id=WP_002864195.1] [location=complement(174709..175896)] [gbkey=CDS]	1188	92.10%	72.30%
[locus_tag=DQM41_RS00965]	lcl|NZ_LS483362.1_cds_WP_010891845.1_187	205	507	303	forward	205	507	303	205	507	[protein=oligopeptide transporter, OPT family] [protein_id=WP_010891845.1] [location=complement(186279..188276)] [gbkey=CDS]	1998	98.00%	30.30%
[locus_tag=DQM41_RS01005]	lcl|NZ_LS483362.1_cds_WP_002851896.1_195	1	717	717	reverse	1	717	717	1	717	[protein=amidophosphoribosyltransferase] [protein_id=WP_002851896.1] [location=194619..195956] [gbkey=CDS]	1338	94.10%	71.70%
[locus_tag=DQM41_RS01070]	lcl|NZ_LS483362.1_cds_WP_002851723.1_208	397	1338	942	reverse	397	1338	942	397	1338	[protein=HlyC/CorC family transporter] [protein_id=WP_002851723.1] [location=complement(206853..208211)] [gbkey=CDS]	1359	87.30%	94.20%
[locus_tag=DQM41_RS01170]	lcl|NZ_LS483362.1_cds_WP_002851641.1_227	1	486	486	forward	1	486	486	1	486	[protein=hypothetical protein] [protein_id=WP_002851641.1] [location=224294..224791] [gbkey=CDS]	498	82.70%	48.60%
[locus_tag=DQM41_RS01300]	lcl|NZ_LS483362.1_cds_WP_002851928.1_253	148	915	768	reverse	148	915	768	148	915	[gene=ilvE] [protein=branched-chain-amino-acid transaminase] [protein_id=WP_002851928.1] [location=complement(247650..248564)] [gbkey=CDS]	915	100.00%	76.80%
[locus_tag=DQM41_RS01310]	lcl|NZ_LS483362.1_cds_WP_002854561.1_255	1	456	456	forward	1	456	456	1	456	[protein=thioredoxin-dependent thiol peroxidase] [protein_id=WP_002854561.1] [location=248944..249399] [gbkey=CDS]	456	100.00%	45.60%
[locus_tag=DQM41_RS01315]	lcl|NZ_LS483362.1_cds_WP_002858664.1_256	22	1017	996	forward	22	1017	996	22	1017	[protein=epoxyqueuosine reductase QueH] [protein_id=WP_002858664.1] [location=249399..250490] [gbkey=CDS]	1092	91.10%	99.60%
[locus_tag=DQM41_RS01370]	lcl|NZ_LS483362.1_cds_WP_002851649.1_267	1150	2148	999	reverse	1150	2148	999	1150	2148	[protein=response regulator] [protein_id=WP_002851649.1] [location=complement(260898..263207)] [gbkey=CDS]	2310	95.30%	99.90%
[locus_tag=DQM41_RS01400]	lcl|NZ_LS483362.1_cds_273	444	1079	636	reverse	444	1079	636	444	1079	[protein=MFS transporter] [pseudo=true] [location=complement(267510..268878)] [gbkey=CDS]	1369	91.50%	63.60%
[locus_tag=DQM41_RS01415]	lcl|NZ_LS483362.1_cds_WP_002860322.1_275	1	660	660	forward	1	660	660	1	660	[protein=tRNA cyclic N6-threonylcarbamoyladenosine(37) synthase TcdA] [protein_id=WP_002860322.1] [location=269824..270483] [gbkey=CDS]	660	78.50%	66.00%
[locus_tag=DQM41_RS01430]	lcl|NZ_LS483362.1_cds_WP_002778123.1_278	447	848	402	reverse	447	848	402	447	848	[protein=pantoate--beta-alanine ligase] [protein_id=WP_002778123.1] [location=complement(271412..272260)] [gbkey=CDS]	849	99.30%	40.20%
[locus_tag=DQM41_RS01440]	lcl|NZ_LS483362.1_cds_WP_002783228.1_280	1	498	498	forward	1	498	498	1	498	[protein=OXA-61 family class D beta-lactamase OXA-193] [protein_id=WP_002783228.1] [location=273315..274088] [gbkey=CDS]	774	99.40%	49.80%
[locus_tag=DQM41_RS01520]	lcl|NZ_LS483362.1_cds_WP_002858702.1_296	1	645	645	forward	1	645	645	1	645	[protein=HAD-IIA family hydrolase] [protein_id=WP_002858702.1] [location=285487..286254] [gbkey=CDS]	768	99.50%	64.50%
[locus_tag=DQM41_RS01770]	lcl|NZ_LS483362.1_cds_WP_002872052.1_346	1080	2063	984	reverse	1080	2063	984	1080	2063	[gene=cmeB] [protein=multidrug efflux RND transporter permease subunit CmeB] [protein_id=WP_002872052.1] [location=complement(332589..335711)] [gbkey=CDS]	3123	92.90%	98.40%
[locus_tag=DQM41_RS01885]	lcl|NZ_LS483362.1_cds_WP_002858719.1_369	229	960	732	forward	229	960	732	229	960	[gene=trpS] [protein=tryptophan--tRNA ligase] [protein_id=WP_002858719.1] [location=353302..354261] [gbkey=CDS]	960	91.00%	73.20%
[locus_tag=DQM41_RS01890]	lcl|NZ_LS483362.1_cds_WP_002858681.1_370	262	1236	975	forward	262	1236	975	262	1236	[gene=serS] [protein=serine--tRNA ligase] [protein_id=WP_002858681.1] [location=354272..355507] [gbkey=CDS]	1236	92.90%	97.50%
[locus_tag=DQM41_RS01990]	lcl|NZ_LS483362.1_cds_WP_002854193.1_390	193	1191	999	forward	193	1191	999	193	1191	[protein=fumarate reductase flavoprotein subunit] [protein_id=WP_002854193.1] [location=373348..375339] [gbkey=CDS]	1992	98.80%	99.90%
[locus_tag=DQM41_RS02050]	lcl|NZ_LS483362.1_cds_WP_002858716.1_402	490	957	468	reverse	490	957	468	490	957	[protein=membrane protein] [protein_id=WP_002858716.1] [location=complement(385765..386721)] [gbkey=CDS]	957	92.00%	46.80%
[locus_tag=DQM41_RS02380]	lcl|NZ_LS483362.1_cds_WP_010891860.1_458	1810	2808	999	forward	1810	2808	999	1810	2808	[gene=rpoB] [protein=DNA-directed RNA polymerase subunit beta] [protein_id=WP_010891860.1] [location=438986..443122] [gbkey=CDS]	4137	99.70%	99.90%
[locus_tag=DQM41_RS02385]	lcl|NZ_LS483362.1_cds_WP_002858508.1_459	3685	4554	870	forward	3685	4554	870	3685	4554	[gene=rpoC] [protein=DNA-directed RNA polymerase subunit beta'] [protein_id=WP_002858508.1] [location=443115..447668] [gbkey=CDS]	4554	96.60%	87.00%
[locus_tag=DQM41_RS02395]	lcl|NZ_LS483362.1_cds_WP_002858487.1_461	112	909	798	forward	112	909	798	112	909	[protein=dihydrodipicolinate synthase family protein] [protein_id=WP_002858487.1] [location=448691..449599] [gbkey=CDS]	909	94.00%	79.80%
[locus_tag=DQM41_RS02410]	lcl|NZ_LS483362.1_cds_WP_002864169.1_464	1	759	759	forward	1	759	759	1	759	[protein=MFS transporter] [protein_id=WP_002864169.1] [location=451040..452272] [gbkey=CDS]	1233	99.60%	75.90%
[locus_tag=DQM41_RS02410]	lcl|NZ_LS483362.1_cds_WP_002864169.1_464	762	1229	468	forward	762	1229	468	762	1229	[protein=MFS transporter] [protein_id=WP_002864169.1] [location=451040..452272] [gbkey=CDS]	1233	98.70%	46.80%
[locus_tag=DQM41_RS02420]	lcl|NZ_LS483362.1_cds_WP_002864166.1_466	3	452	450	forward	3	452	450	3	452	[gene=fucP] [protein=L-fucose:H+ symporter permease] [protein_id=WP_002864166.1] [location=453113..454369] [gbkey=CDS]	1257	90.40%	45.00%
[locus_tag=DQM41_RS02420]	lcl|NZ_LS483362.1_cds_WP_002864166.1_466	676	1257	582	forward	676	1257	582	676	1257	[gene=fucP] [protein=L-fucose:H+ symporter permease] [protein_id=WP_002864166.1] [location=453113..454369] [gbkey=CDS]	1257	100.00%	58.20%
[locus_tag=DQM41_RS02435]	lcl|NZ_LS483362.1_cds_469	333	1331	999	forward	333	1331	999	333	1331	[gene=aldA] [protein=aldehyde dehydrogenase] [pseudo=true] [location=455455..456893] [gbkey=CDS]	1439	99.10%	99.90%
[locus_tag=DQM41_RS02440]	lcl|NZ_LS483362.1_cds_WP_002782934.1_470	76	387	312	forward	76	387	312	76	387	[protein=30S ribosomal protein S12] [protein_id=WP_002782934.1] [location=457221..457607] [gbkey=CDS]	387	97.10%	31.20%
[locus_tag=DQM41_RS02450]	lcl|NZ_LS483362.1_cds_WP_002779472.1_472	1	627	627	forward	1	627	627	1	627	[gene=fusA] [protein=elongation factor G] [protein_id=WP_002779472.1] [location=458160..460235] [gbkey=CDS]	2076	98.60%	62.70%
[locus_tag=DQM41_RS02610]	lcl|NZ_LS483362.1_cds_WP_002851122.1_503	120	188	69	forward	120	188	69	120	188	[gene=fliE] [protein=flagellar hook-basal body complex protein FliE] [protein_id=WP_002851122.1] [location=complement(489149..489445)] [gbkey=CDS]	297	39.10%	6.90%
[locus_tag=DQM41_RS02615]	lcl|NZ_LS483362.1_cds_WP_002858484.1_504	1	495	495	reverse	1	495	495	1	495	[gene=flgC] [protein=flagellar basal body rod protein FlgC] [protein_id=WP_002858484.1] [location=complement(489452..489946)] [gbkey=CDS]	495	92.40%	49.50%
[locus_tag=DQM41_RS02720]	lcl|NZ_LS483362.1_cds_WP_002864504.1_525	43	690	648	forward	43	690	648	43	690	[gene=fliD] [protein=flagellar filament capping protein FliD] [protein_id=WP_002864504.1] [location=510546..512474] [gbkey=CDS]	1929	93.10%	64.80%
[locus_tag=DQM41_RS02990]	lcl|NZ_LS483362.1_cds_WP_002852205.1_576	832	1320	489	reverse	832	1320	489	832	1320	[protein=MotA/TolQ/ExbB proton channel family protein] [protein_id=WP_002852205.1] [location=555204..556646] [gbkey=CDS]	1443	80.40%	48.90%
[locus_tag=DQM41_RS03065]	lcl|NZ_LS483362.1_cds_WP_002868908.1_591	1	564	564	forward	1	564	564	1	564	[protein=phosphate ABC transporter substrate-binding protein] [protein_id=WP_002868908.1] [location=573215..574210] [gbkey=CDS]	996	87.00%	56.40%
[locus_tag=DQM41_RS03070]	lcl|NZ_LS483362.1_cds_WP_002776292.1_592	3	560	558	forward	3	560	558	3	560	[gene=pstC] [protein=phosphate ABC transporter permease subunit PstC] [protein_id=WP_002776292.1] [location=574220..575134] [gbkey=CDS]	915	99.50%	55.80%
[locus_tag=DQM41_RS03080]	lcl|NZ_LS483362.1_cds_WP_010891867.1_594	1	564	564	forward	1	564	564	1	564	[protein=phosphate ABC transporter ATP-binding protein] [protein_id=WP_010891867.1] [location=576216..576956] [gbkey=CDS]	741	99.50%	56.40%
[locus_tag=DQM41_RS03095]	lcl|NZ_LS483362.1_cds_WP_002858500.1_597	154	648	495	forward	154	648	495	154	648	[protein=M48 family metallopeptidase] [protein_id=WP_002858500.1] [location=579627..580274] [gbkey=CDS]	648	97.60%	49.50%
[locus_tag=DQM41_RS03100]	lcl|NZ_LS483362.1_cds_WP_002826526.1_598	466	1461	996	forward	466	1461	996	466	1461	[protein=hypothetical protein] [protein_id=WP_002826526.1] [location=580316..581836] [gbkey=CDS]	1521	91.50%	99.60%
[locus_tag=DQM41_RS03105]	lcl|NZ_LS483362.1_cds_WP_002826528.1_599	964	1962	999	forward	964	1962	999	964	1962	[gene=hypF] [protein=carbamoyltransferase HypF] [protein_id=WP_002826528.1] [location=581817..584006] [gbkey=CDS]	2190	78.50%	99.90%
[locus_tag=DQM41_RS03110]	lcl|NZ_LS483362.1_cds_WP_002776330.1_600	1	693	693	forward	1	693	693	1	693	[gene=hypB] [protein=hydrogenase nickel incorporation protein HypB] [protein_id=WP_002776330.1] [location=584084..584827] [gbkey=CDS]	744	94.60%	69.30%
[locus_tag=DQM41_RS03125]	lcl|NZ_LS483362.1_cds_WP_002776344.1_603	304	597	294	forward	304	597	294	304	597	[gene=hypE] [protein=hydrogenase expression/formation protein HypE] [protein_id=WP_002776344.1] [location=586180..587154] [gbkey=CDS]	975	96.90%	29.40%
[locus_tag=DQM41_RS03340]	lcl|NZ_LS483362.1_cds_WP_002865516.1_646	42	545	504	forward	42	545	504	42	545	[protein=anaerobic C4-dicarboxylate transporter] [protein_id=WP_002865516.1] [location=625542..626966] [gbkey=CDS]	1425	82.70%	50.40%
[locus_tag=DQM41_RS03630]	lcl|NZ_LS483362.1_cds_WP_002852367.1_704	346	984	639	reverse	346	984	639	346	984	[gene=corA] [protein=magnesium/cobalt transporter CorA] [protein_id=WP_002852367.1] [location=complement(680358..681341)] [gbkey=CDS]	984	89.70%	63.90%
[locus_tag=DQM41_RS03635]	lcl|NZ_LS483362.1_cds_WP_002852095.1_705	1	495	495	forward	1	495	495	1	495	[protein=extracellular solute-binding protein] [protein_id=WP_002852095.1] [location=681458..682504] [gbkey=CDS]	1047	98.80%	49.50%
[locus_tag=DQM41_RS03635]	lcl|NZ_LS483362.1_cds_WP_002852095.1_705	541	1047	507	forward	541	1047	507	541	1047	[protein=extracellular solute-binding protein] [protein_id=WP_002852095.1] [location=681458..682504] [gbkey=CDS]	1047	100.00%	50.70%
[locus_tag=DQM41_RS03640]	lcl|NZ_LS483362.1_cds_WP_002793291.1_706	484	1326	843	forward	484	1326	843	484	1326	[protein=esterase-like activity of phytase family protein] [protein_id=WP_002793291.1] [location=682515..683840] [gbkey=CDS]	1326	98.60%	84.30%
[locus_tag=DQM41_RS03725]	lcl|NZ_LS483362.1_cds_WP_002792114.1_718	2	106	105	forward	2	106	105	2	106	[protein=hypothetical protein] [protein_id=WP_002792114.1] [location=702795..702902] [gbkey=CDS]	108	97.10%	10.50%
[locus_tag=DQM41_RS03750]	lcl|NZ_LS483362.1_cds_WP_010891883.1_723	1021	2019	999	forward	1021	2019	999	1021	2019	[gene=cfrA][protein=TonB-dependent ferric enterobactin receptor CfrA] [protein_id=WP_010891883.1] [location=705435..707525] [gbkey=CDS]	2091	94.90%	99.90%
[locus_tag=DQM41_RS03760]	lcl|NZ_LS483362.1_cds_WP_002780052.1_725	1	531	531	forward	1	531	531	1	531	[gene=grpE] [protein=nucleotide exchange factor GrpE] [protein_id=WP_002780052.1] [location=708616..709146] [gbkey=CDS]	531	90.80%	53.10%
[locus_tag=DQM41_RS03800]	lcl|NZ_LS483362.1_cds_WP_002852541.1_733	151	1146	996	reverse	151	1146	996	151	1146	[protein=histidine--tRNA ligase] [protein_id=WP_002852541.1] [location=complement(716594..717820)] [gbkey=CDS]	1227	90.50%	99.60%
[locus_tag=DQM41_RS03805]	lcl|NZ_LS483362.1_cds_WP_002852526.1_734	1	579	579	reverse	1	579	579	1	579	[protein=dTMP kinase] [protein_id=WP_002852526.1] [location=complement(717817..718395)] [gbkey=CDS]	579	99.50%	57.90%
[locus_tag=DQM41_RS03945]	lcl|NZ_LS483362.1_cds_WP_002864146.1_762	298	423	126	forward	298	423	126	298	423	[protein=hypothetical protein] [protein_id=WP_002864146.1] [location=744480..745760] [gbkey=CDS]	1281	95.20%	12.60%
[locus_tag=DQM41_RS04005]	lcl|NZ_LS483362.1_cds_WP_002852574.1_774	340	897	558	forward	340	897	558	340	897	[protein=4-hydroxy-tetrahydrodipicolinate synthase] [protein_id=WP_002852574.1] [location=758686..759582] [gbkey=CDS]	897	95.70%	55.80%
[locus_tag=DQM41_RS04280]	lcl|NZ_LS483362.1_cds_WP_002864843.1_829	1441	1509	69	forward	1441	1509	69	1441	1509	[protein=chorismate-binding protein] [protein_id=WP_002864843.1] [location=complement(807480..809264)] [gbkey=CDS]	1785	47.80%	6.90%
[locus_tag=DQM41_RS04365]	lcl|NZ_LS483362.1_cds_WP_002852538.1_846	1078	2076	999	forward	1078	2076	999	1078	2076	[gene=flgL] [protein=flagellar hook-associated protein FlgL] [protein_id=WP_002852538.1] [location=complement(823616..825868)] [gbkey=CDS]	2253	98.50%	99.90%
[locus_tag=DQM41_RS05115]	lcl|NZ_LS483362.1_cds_WP_002837927.1_985	235	999	765	reverse	235	999	765	235	999	[gene=lepA] [protein=elongation factor 4] [protein_id=WP_002837927.1] [location=complement(961538..963334)] [gbkey=CDS]	1797	97.30%	76.50%
[locus_tag=DQM41_RS05125]	lcl|NZ_LS483362.1_cds_WP_002858463.1_987	1	522	522	forward	1	522	522	1	522	[gene=cmeE] [protein=multidrug efflux RND transporter periplasmic adaptor subunit CmeE] [protein_id=WP_002858463.1] [location=964732..965472] [gbkey=CDS]	741	92.30%	52.20%
[locus_tag=DQM41_RS05130]	lcl|NZ_LS483362.1_cds_WP_010891901.1_988	781	1779	999	forward	781	1779	999	781	1779	[gene=cmeF] [protein=multidrug efflux RND transporter permease subunit CmeF] [protein_id=WP_010891901.1] [location=965475..968492] [gbkey=CDS]	3018	91.40%	99.90%
[locus_tag=DQM41_RS05130]	lcl|NZ_LS483362.1_cds_WP_010891901.1_988	1780	2778	999	forward	1780	2778	999	1780	2778	[gene=cmeF] [protein=multidrug efflux RND transporter permease subunit CmeF] [protein_id=WP_010891901.1] [location=965475..968492] [gbkey=CDS]	3018	96.30%	99.90%
[locus_tag=DQM41_RS05220]	lcl|NZ_LS483362.1_cds_WP_002864285.1_1006	1903	2628	726	reverse	1903	2628	726	1903	2628	[protein=N-6 DNA methylase] [protein_id=WP_002864285.1] [location=complement(982973..986992)] [gbkey=CDS]	4020	94.20%	72.60%
[locus_tag=DQM41_RS05220]	lcl|NZ_LS483362.1_cds_WP_002864285.1_1006	901	1899	999	reverse	901	1899	999	901	1899	[protein=N-6 DNA methylase] [protein_id=WP_002864285.1] [location=complement(982973..986992)] [gbkey=CDS]	4020	100.00%	99.90%
[locus_tag=DQM41_RS05220]	lcl|NZ_LS483362.1_cds_WP_002864285.1_1006	1	900	900	reverse	1	900	900	1	900	[protein=N-6 DNA methylase] [protein_id=WP_002864285.1] [location=complement(982973..986992)] [gbkey=CDS]	4020	99.00%	90.00%
[locus_tag=DQM41_RS05225]	lcl|NZ_LS483362.1_cds_WP_010891903.1_1007	121	1119	999	reverse	121	1119	999	121	1119	[protein=endonuclease MutS2] [protein_id=WP_010891903.1] [location=complement(987001..989211)] [gbkey=CDS]	2211	99.40%	99.90%
[locus_tag=DQM41_RS05635]	lcl|NZ_LS483362.1_cds_WP_002858148.1_1089	379	1377	999	forward	379	1377	999	379	1377	[protein=glycosyltransferase] [protein_id=WP_002858148.1] [location=1068683..1070230] [gbkey=CDS]	1548	73.30%	99.90%
[locus_tag=DQM41_RS05695]	lcl|NZ_LS483362.1_cds_WP_002858021.1_1101	1	531	531	reverse	1	531	531	1	531	[gene=gmhA] [protein=D-sedoheptulose 7-phosphate isomerase] [protein_id=WP_002858021.1] [location=complement(1081831..1082391)] [gbkey=CDS]	561	97.20%	53.10%
[locus_tag=DQM41_RS05780]	lcl|NZ_LS483362.1_cds_WP_002852826.1_1118	292	777	486	reverse	292	777	486	292	777	[protein=threonine/serine exporter ThrE family protein] [protein_id=WP_002852826.1] [location=complement(1095911..1096687)] [gbkey=CDS]	777	87.70%	48.60%
[locus_tag=DQM41_RS05790]	lcl|NZ_LS483362.1_cds_WP_002864829.1_1120	3	602	600	reverse	3	602	600	3	602	[protein=DedA family protein] [protein_id=WP_002864829.1] [location=complement(1097677..1098279)] [gbkey=CDS]	603	94.60%	60.00%
[locus_tag=DQM41_RS05800]	lcl|NZ_LS483362.1_cds_WP_010891915.1_1122	133	648	516	reverse	133	648	516	133	648	[gene=omp50] [protein=outer membrane tyrosine kinase] [protein_id=WP_010891915.1] [location=complement(1098598..1100019)] [gbkey=CDS]	1422	98.80%	51.60%
[locus_tag=DQM41_RS05875]	lcl|NZ_LS483362.1_cds_WP_002852758.1_1137	184	894	711	reverse	184	894	711	184	894	[protein=cytochrome bc complex cytochrome b subunit] [protein_id=WP_002852758.1] [location=complement(1112094..1113344)] [gbkey=CDS]	1251	96.20%	71.10%
[locus_tag=DQM41_RS05890]	lcl|NZ_LS483362.1_cds_WP_002864546.1_1140	619	1617	999	reverse	619	1617	999	619	1617	[gene=mnmG] [locus_tag=DQM41_RS05890] [protein=tRNA uridine-5-carboxymethylaminomethyl(34) synthesis enzyme MnmG] [protein_id=WP_002864546.1] [location=complement(1115226..1117085)] [gbkey=CDS]	1860	84.20%	99.90%
[locus_tag=DQM41_RS05900]	lcl|NZ_LS483362.1_cds_WP_002790076.1_1142	817	1380	564	reverse	817	1380	564	817	1380	[gene=cetA] [protein=bipartate energy taxis response protein CetA] [protein_id=WP_002790076.1] [location=complement(1117688..1119067)] [gbkey=CDS]	1380	99.50%	56.40%
[locus_tag=DQM41_RS05900]	lcl|NZ_LS483362.1_cds_WP_002790076.1_1142	1	597	597	reverse	1	597	597	1	597	[gene=cetA] [protein=bipartate energy taxis response protein CetA] [protein_id=WP_002790076.1] [location=complement(1117688..1119067)] [gbkey=CDS]	1380	99.50%	59.70%
[locus_tag=DQM41_RS05910]	lcl|NZ_LS483362.1_cds_WP_002852850.1_1144	46	519	474	forward	46	519	474	46	519	[protein=cation:dicarboxylase symporter family transporter] [protein_id=WP_002852850.1] [location=1119951..1121327] [gbkey=CDS]	1377	98.70%	47.40%
[locus_tag=DQM41_RS05995]	lcl|NZ_LS483362.1_cds_WP_002858133.1_1161	706	1554	849	forward	706	1554	849	706	1554	[gene=rny] [protein=ribonuclease Y] [protein_id=WP_002858133.1] [location=1137485..1139038] [gbkey=CDS]	1554	96.80%	84.90%
[locus_tag=DQM41_RS06015]	lcl|NZ_LS483362.1_cds_WP_002852810.1_1165	979	1140	162	reverse	979	1140	162	979	1140	[protein=FAD-binding protein] [protein_id=WP_002852810.1] [location=complement(1141617..1142999)] [gbkey=CDS]	1383	31.50%	16.20%
[locus_tag=DQM41_RS06015]	lcl|NZ_LS483362.1_cds_WP_002852810.1_1165	979	1140	162	reverse	979	1140	162	979	1140	[protein=FAD-binding protein] [protein_id=WP_002852810.1] [location=complement(1141617..1142999)] [gbkey=CDS]	1383	31.50%	16.20%
[locus_tag=DQM41_RS06070]	lcl|NZ_LS483362.1_cds_WP_002853178.1_1176	1	381	381	reverse	1	381	381	1	381	[protein=bacteriohemerythrin] [protein_id=WP_002853178.1] [location=1153134..1153733] [gbkey=CDS]	600	80.30%	38.10%
[locus_tag=DQM41_RS06085]	lcl|NZ_LS483362.1_cds_WP_002853241.1_1178	1	402	402	reverse	1	402	402	1	402	[protein=HAMP domain-containing histidine kinase] [protein_id=WP_002853241.1] [location=complement(1154250..1155497)] [gbkey=CDS]	1248	82.40%	40.20%
[locus_tag=DQM41_RS06385]	lcl|NZ_LS483362.1_cds_WP_002857885.1_1236	1	672	672	reverse	1	672	672	1	672	[protein=hypothetical protein] [protein_id=WP_002857885.1] [location=complement(1217440..1218111)] [gbkey=CDS]	672	91.80%	67.20%
[locus_tag=DQM41_RS06395]	lcl|NZ_LS483362.1_cds_WP_002854063.1_1238	589	1131	543	reverse	589	1131	543	589	1131	[protein=malate dehydrogenase] [protein_id=WP_002854063.1] [location=complement(1218740..1219975)] [gbkey=CDS]	1236	82.60%	54.30%
[locus_tag=DQM41_RS06425]	lcl|NZ_LS483362.1_cds_WP_002858028.1_1244	1	555	555	forward	1	555	555	1	555	[gene=pseB] [protein=UDP-N-acetylglucosamine 4,6-dehydratase (inverting)] [protein_id=WP_002858028.1] [location=1224832..1225836] [gbkey=CDS]	1005	97.80%	55.50%
[locus_tag=DQM41_RS06430]	lcl|NZ_LS483362.1_cds_WP_002856503.1_1245	550	1131	582	forward	550	1131	582	550	1131	[gene=pseC] [protein=UDP-4-amino-4,6-dideoxy-N-acetyl-beta-L-altrosamine transaminase] [protein_id=WP_002856503.1] [location=1225838..1226968] [gbkey=CDS]	1131	97.90%	58.20%
[locus_tag=DQM41_RS06465]	lcl|NZ_LS483362.1_cds_WP_002786080.1_1252	784	1563	780	forward	784	1563	780	784	1563	[protein=HAD-IIIC family phosphatase] [protein_id=WP_002786080.1] [location=1231427..1232989] [gbkey=CDS]	1563	98.50%	78.00%
[locus_tag=DQM41_RS06490]	lcl|NZ_LS483362.1_cds_WP_002858432.1_1257	733	1221	489	reverse	733	1221	489	733	1221	[protein=DUF2920 family protein] [protein_id=WP_002858432.1] [location=complement(1235506..1236732)] [gbkey=CDS]	1227	89.00%	48.90%
[locus_tag=DQM41_RS06495]	lcl|NZ_LS483362.1_cds_WP_002858032.1_1258	1	654	654	forward	1	654	654	1	654	[protein=AMP-binding protein] [protein_id=WP_002858032.1] [location=1236793..1238301] [gbkey=CDS]	1509	98.60%	65.40%
[locus_tag=DQM41_RS06495]	lcl|NZ_LS483362.1_cds_WP_002858032.1_1258	658	1509	852	forward	658	1509	852	658	1509	[protein=AMP-binding protein] [protein_id=WP_002858032.1] [location=1236793..1238301] [gbkey=CDS]	1509	99.30%	85.20%
[locus_tag=DQM41_RS06505]	lcl|NZ_LS483362.1_cds_WP_002856137.1_1260	586	1068	483	reverse	586	1068	483	586	1068	[protein=hypothetical protein] [protein_id=WP_002856137.1] [location=complement(1238565..1239635)] [gbkey=CDS]	1071	100.00%	48.30%
[locus_tag=DQM41_RS06535]	lcl|NZ_LS483362.1_cds_WP_002858306.1_1266	3	746	744	reverse	3	746	744	3	746	[gene=hisF][protein=imidazole glycerol phosphate synthase subunit HisF] [protein_id=WP_002858306.1] [location=complement(1242890..1243636)] [gbkey=CDS]	747	94.00%	74.40%
[locus_tag=DQM41_RS06540]	lcl|NZ_LS483362.1_cds_WP_002856473.1_1267	1	603	603	reverse	1	603	603	1	603	[gene=hisH] [protein=imidazole glycerol phosphate synthase subunit HisH] [protein_id=WP_002856473.1] [location=complement(1243637..1244242)] [gbkey=CDS]	606	96.00%	60.30%
[locus_tag=DQM41_RS06545]	lcl|NZ_LS483362.1_cds_WP_002856493.1_1268	1	735	735	reverse	1	735	735	1	735	[gene=pseA] [protein=pseudaminic acid biosynthesis protein PseA] [protein_id=WP_002856493.1] [location=complement(1244239..1245375)] [gbkey=CDS]	1137	100.00%	73.50%
[locus_tag=DQM41_RS06550]	lcl|NZ_LS483362.1_cds_WP_002870258.1_1269	7	1005	999	forward	7	1005	999	7	1005	[gene=pseI] [protein=pseudaminic acid synthase] [protein_id=WP_002870258.1] [location=1245633..1246664] [gbkey=CDS]	1032	95.80%	99.90%
[locus_tag=DQM41_RS06565]	lcl|NZ_LS483362.1_cds_WP_002786732.1_1272	43	969	927	forward	43	969	927	43	969	[gene=legB] [protein=4,6-dehydratase LegB] [protein_id=WP_002786732.1] [location=1248606..1249577] [gbkey=CDS]	972	99.00%	92.70%
[locus_tag=DQM41_RS06570]	lcl|NZ_LS483362.1_cds_WP_010891923.1_1273	185	1075	891	forward	185	1075	891	185	1075	[gene=legC] [protein=aminotransferase LegC] [protein_id=WP_010891923.1] [location=1249570..1250724] [gbkey=CDS]	1155	95.00%	89.10%
[locus_tag=DQM41_RS06575]	lcl|NZ_LS483362.1_cds_WP_002815677.1_1274	112	537	426	forward	112	537	426	112	537	[protein=transferase] [protein_id=WP_002815677.1] [location=1250834..1251376] [gbkey=CDS]	543	98.60%	42.60%
[locus_tag=DQM41_RS06605]	lcl|NZ_LS483362.1_cds_WP_002864264.1_1280	358	1026	669	forward	358	1026	669	358	1026	[protein=CBS domain-containing protein] [protein_id=WP_002864264.1] [location=1256273..1257298] [gbkey=CDS]	1026	98.20%	66.90%
[locus_tag=DQM41_RS06625]	lcl|NZ_LS483362.1_cds_WP_010891924.1_1284	169	960	792	forward	169	960	792	169	960	[gene=pseD] [protein=flagellin modification protein PseD] [protein_id=WP_010891924.1] [location=1259658..1261619] [gbkey=CDS]	1962	83.30%	79.20%
[locus_tag=DQM41_RS06630]	lcl|NZ_LS483362.1_cds_WP_010891925.1_1285	1428	1859	432	forward	1428	1859	432	1428	1859	[protein=motility associated factor glycosyltransferase family protein] [protein_id=WP_010891925.1] [location=1261616..1263475] [gbkey=CDS]	1860	91.40%	43.20%
[locus_tag=DQM41_RS06640]	lcl|NZ_LS483362.1_cds_WP_041337234.1_1287	1111	1722	612	forward	1111	1722	612	1111	1722	[protein=motility associated factor glycosyltransferase family protein] [protein_id=WP_041337234.1] [location=1263715..1265436] [gbkey=CDS]	1722	85.30%	61.20%
[locus_tag=DQM41_RS06640]	lcl|NZ_LS483362.1_cds_WP_041337234.1_1287	1021	1722	702	forward	1021	1722	702	1021	1722	[protein=motility associated factor glycosyltransferase family protein] [protein_id=WP_041337234.1] [location=1263715..1265436] [gbkey=CDS]	1722	82.20%	70.20%
[locus_tag=DQM41_RS06645]	lcl|NZ_LS483362.1_cds_WP_010891926.1_1288	1438	1887	450	forward	1438	1887	450	1438	1887	[protein=motility associated factor glycosyltransferase family protein] [protein_id=WP_010891926.1] [location=1265433..1267319] [gbkey=CDS]	1887	98.00%	45.00%
[locus_tag=DQM41_RS06650]	lcl|NZ_LS483362.1_cds_WP_002852395.1_1289	436	1203	768	reverse	436	1203	768	436	1203	[protein=flagellin A] [protein_id=WP_002852395.1] [location=complement(1267350..1269068)] [gbkey=CDS]	1719	95.70%	76.80%
[locus_tag=DQM41_RS06665]	lcl|NZ_LS483362.1_cds_WP_010891929.1_1292	37	1032	996	reverse	37	1032	996	37	1032	[protein=motility associated factor glycosyltransferase family protein] [protein_id=WP_010891929.1] [location=complement(1272843..1274666)] [gbkey=CDS]	1824	93.40%	99.60%
[locus_tag=DQM41_RS06695]	lcl|NZ_LS483362.1_cds_WP_002869557.1_1298	274	726	453	reverse	274	726	453	274	726	[protein=phosphatidate cytidylyltransferase] [protein_id=WP_002869557.1] [location=complement(1279900..1280625)] [gbkey=CDS]	726	98.00%	45.30%
[locus_tag=DQM41_RS06715]	lcl|NZ_LS483362.1_cds_WP_002864239.1_1302	205	705	501	forward	205	705	501	205	705	[protein=phospholipase A] [protein_id=WP_002864239.1] [location=1282915..1283904] [gbkey=CDS]	990	90.40%	50.10%
[locus_tag=DQM41_RS06750]	lcl|NZ_LS483362.1_cds_WP_002858075.1_1308	376	1371	996	reverse	376	1371	996	376	1371	[protein=ammonia-forming cytochrome c nitrite reductase subunit c552] [protein_id=WP_002858075.1] [location=complement(1288562..1290394)] [gbkey=CDS]	1833	93.40%	99.60%
[locus_tag=DQM41_RS06910]	lcl|NZ_LS483362.1_cds_1339	906	1439	534	forward	906	1439	534	906	1439	[protein=C4-dicarboxylate ABC transporter] [pseudo=true] [location=1325650..1327089] [gbkey=CDS]	1440	96.10%	53.40%
[locus_tag=DQM41_RS06915]	lcl|NZ_LS483362.1_cds_1340	120	1013	894	forward	120	1013	894	120	1013	[protein=PLP-dependent transferase] [pseudo=true] [location=1327102..1328264] [gbkey=CDS]	1163	98.00%	89.40%
[locus_tag=DQM41_RS06920]	lcl|NZ_LS483362.1_cds_WP_002857989.1_1341	169	840	672	forward	169	840	672	169	840	[protein=adenylosuccinate lyase] [protein_id=WP_002857989.1] [location=1328274..1329641] [gbkey=CDS]	1368	90.90%	67.20%
[locus_tag=DQM41_RS06935]	lcl|NZ_LS483362.1_cds_WP_002865443.1_1344	1	822	822	forward	1	822	822	1	822	[gene=feoB] [protein=ferrous iron transport protein B] [protein_id=WP_002865443.1] [location=1331293..1333134] [gbkey=CDS]	1842	94.00%	82.20%
[locus_tag=DQM41_RS06935]	lcl|NZ_LS483362.1_cds_WP_002865443.1_1344	823	1821	999	forward	823	1821	999	823	1821	[gene=feoB] [protein=ferrous iron transport protein B] [protein_id=WP_002865443.1] [location=1331293..1333134] [gbkey=CDS]	1842	96.20%	99.90%
[locus_tag=DQM41_RS06940]	lcl|NZ_LS483362.1_cds_WP_002858455.1_1345	511	1494	984	reverse	511	1494	984	511	1494	[protein=hydrogenase small subunit] [protein_id=WP_002858455.1] [location=complement(1333131..1334624)] [gbkey=CDS]	1494	95.90%	98.40%
[locus_tag=DQM41_RS06945]	lcl|NZ_LS483362.1_cds_WP_002780456.1_1346	340	825	486	reverse	340	825	486	340	825	[gene=fabI] [protein=enoyl-ACP reductase FabI] [protein_id=WP_002780456.1] [location=complement(1334628..1335452)] [gbkey=CDS]	825	100.00%	48.60%
[locus_tag=DQM41_RS06950]	lcl|NZ_LS483362.1_cds_WP_002855528.1_1347	16	651	636	reverse	16	651	636	16	651	[protein=triose-phosphate isomerase] [protein_id=WP_002855528.1] [location=complement(1335456..1336127)] [gbkey=CDS]	672	91.50%	63.60%
[locus_tag=DQM41_RS06955]	lcl|NZ_LS483362.1_cds_WP_010891932.1_1348	214	1029	816	reverse	214	1029	816	214	1029	[protein=phosphoglycerate kinase] [protein_id=WP_010891932.1] [location=complement(1336124..1337326)] [gbkey=CDS]	1203	91.50%	81.60%
[locus_tag=DQM41_RS06960]	lcl|NZ_LS483362.1_cds_WP_002780453.1_1349	216	995	780	reverse	216	995	780	216	995	[gene=gap] [protein=type I glyceraldehyde-3-phosphate dehydrogenase] [protein_id=WP_002780453.1] [location=complement(1337329..1338327)] [gbkey=CDS]	999	96.50%	78.00%
[locus_tag=DQM41_RS07085]	lcl|NZ_LS483362.1_cds_WP_002864205.1_1374	361	774	414	reverse	361	774	414	361	774	[protein=GDP-L-fucose synthase] [protein_id=WP_002864205.1] [location=complement(1362139..1363179)] [gbkey=CDS]	1041	40.60%	41.40%
[locus_tag=DQM41_RS07230]	lcl|NZ_LS483362.1_cds_WP_002851372.1_1403	373	822	450	reverse	373	822	450	373	822	[protein=thiamine-phosphate kinase] [protein_id=WP_002851372.1] [location=complement(1395354..1396175)] [gbkey=CDS]	822	89.30%	45.00%
[locus_tag=DQM41_RS07235]	lcl|NZ_LS483362.1_cds_WP_002851142.1_1404	100	174	75	reverse	100	174	75	100	174	[protein=DUF5644 domain-containing protein] [protein_id=WP_002851142.1] [location=1396262..1397335] [gbkey=CDS]	1074	44.00%	7.50%
[locus_tag=DQM41_RS07425]	lcl|NZ_LS483362.1_cds_WP_002851458.1_1442	706	1239	534	reverse	706	1239	534	706	1239	[protein=adenylosuccinate synthase] [protein_id=WP_002851458.1] [location=complement(1431075..1432325)] [gbkey=CDS]	1251	97.80%	53.40%
[locus_tag=DQM41_RS07465]	lcl|NZ_LS483362.1_cds_WP_002851362.1_1449	1516	2103	588	reverse	1516	2103	588	1516	2103	[gene=ccaA] [protein=aspartate chemotaxis receptor CcaA] [protein_id=WP_002851362.1] [location=complement(1440866..1442968)] [gbkey=CDS]	2103	94.00%	58.80%
[locus_tag=DQM41_RS07465]	lcl|NZ_LS483362.1_cds_WP_002851362.1_1449	517	1512	996	reverse	517	1512	996	517	1512	[gene=ccaA] [protein=aspartate chemotaxis receptor CcaA] [protein_id=WP_002851362.1] [location=complement(1440866..1442968)] [gbkey=CDS]	2103	97.90%	99.60%
[locus_tag=DQM41_RS07475]	lcl|NZ_LS483362.1_cds_WP_002851178.1_1451	145	783	639	reverse	145	783	639	145	783	[gene=fdhD] [protein=formate dehydrogenase accessory sulfurtransferase FdhD] [protein_id=WP_002851178.1] [location=complement(1443808..1444590)] [gbkey=CDS]	783	98.10%	63.90%
[locus_tag=DQM41_RS07490]	lcl|NZ_LS483362.1_cds_WP_010891941.1_1454	1	717	717	reverse	1	717	717	1	717	[protein=formate dehydrogenase subunit alpha] [transl_except=(pos:541..543,aa:Sec)] [protein_id=WP_010891941.1] [location=complement(1446324..1449128)] [gbkey=CDS]	2805	91.00%	71.70%
[locus_tag=DQM41_RS07595]	lcl|NZ_LS483362.1_cds_WP_002851288.1_1475	502	1497	996	reverse	502	1497	996	502	1497	[gene=acs] [protein=acetate--CoA ligase] [protein_id=WP_002851288.1] [location=complement(1468392..1470365)] [gbkey=CDS]	1974	96.20%	99.60%
[locus_tag=DQM41_RS07610]	lcl|NZ_LS483362.1_cds_WP_002851335.1_1478	1	591	591	forward	1	591	591	1	591	[protein=substrate-binding domain-containing protein] [protein_id=WP_002851335.1] [location=1472265..1473074] [gbkey=CDS]	810	88.60%	59.10%
[locus_tag=DQM41_RS07660]	lcl|NZ_LS483362.1_cds_WP_002851428.1_1488	79	186	108	forward	79	186	108	79	186	[protein=ATP-binding protein] [protein_id=WP_002851428.1] [location=complement(1482471..1484237)] [gbkey=CDS]	1767	33.30%	10.80%
[locus_tag=DQM41_RS07705]	lcl|NZ_LS483362.1_cds_WP_002851141.1_1497	1	420	420	reverse	1	420	420	1	420	[protein=MerR family transcriptional regulator] [protein_id=WP_002851141.1] [location=complement(1491475..1491906)] [gbkey=CDS]	432	97.90%	42.00%
[locus_tag=DQM41_RS07710]	lcl|NZ_LS483362.1_cds_WP_010891944.1_1498	928	1491	564	reverse	928	1491	564	928	1491	[protein=methyl-accepting chemotaxis protein] [protein_id=WP_010891944.1] [location=1491986..1493974] [gbkey=CDS]	1989	70.20%	56.40%
[locus_tag=DQM41_RS07755]	lcl|NZ_LS483362.1_cds_WP_010891945.1_1507	1	735	735	forward	1	735	735	1	735	[protein=NADH-quinone oxidoreductase subunit G] [protein_id=WP_010891945.1] [location=complement(1503514..1505976)] [gbkey=CDS]	2463	98.40%	73.50%
[locus_tag=DQM41_RS07815]	lcl|NZ_LS483362.1_cds_WP_002851210.1_1519	169	1167	999	forward	169	1167	999	169	1167	[protein=FAD-binding oxidoreductase] [protein_id=WP_002851210.1] [location=complement(1514627..1517398)] [gbkey=CDS]	2772	91.30%	99.90%
[locus_tag=DQM41_RS07815]	lcl|NZ_LS483362.1_cds_WP_002851210.1_1519	1168	2166	999	forward	1168	2166	999	1168	2166	[protein=FAD-binding oxidoreductase] [protein_id=WP_002851210.1] [location=complement(1514627..1517398)] [gbkey=CDS]	2772	98.50%	99.90%
[locus_tag=DQM41_RS08085]	lcl|NZ_LS483362.1_cds_WP_002851153.1_1569	1	648	648	forward	1	648	648	1	648	[protein=ribonuclease III] [protein_id=WP_002851153.1] [location=complement(1560794..1561468)] [gbkey=CDS]	675	90.70%	64.80%
[locus_tag=DQM41_RS08185]	lcl|NZ_LS483362.1_cds_WP_002799206.1_1589	1	537	537	forward	1	537	537	1	537	[gene=nhaA] [protein=Na+/H+ antiporter NhaA] [protein_id=WP_002799206.1] [location=complement(1578469..1579617)] [gbkey=CDS]	1149	90.80%	53.70%
[locus_tag=DQM41_RS08350]	lcl|NZ_LS483362.1_cds_WP_002851268.1_1617	598	1596	999	reverse	598	1596	999	598	1596	[protein=type I DNA topoisomerase] [protein_id=WP_002851268.1] [location=complement(1607343..1609445)] [gbkey=CDS]	2103	96.10%	99.90%
[locus_tag=DQM41_RS08395]	lcl|NZ_LS483362.1_cds_WP_002851453.1_1626	1	381	381	reverse	1	381	381	1	381	[gene=rplE] [protein=50S ribosomal protein L5] [protein_id=WP_002851453.1] [location=complement(1614629..1615174)] [gbkey=CDS]	546	96.90%	38.10%
[locus_tag=DQM41_RS08450]	lcl|NZ_LS483362.1_cds_WP_002851146.1_1637	1	615	615	reverse	1	615	615	1	615	[gene=rplD] [protein=50S ribosomal protein L4] [protein_id=WP_002851146.1] [location=complement(1619176..1619790)] [gbkey=CDS]	615	96.30%	61.50%
[locus_tag=DQM41_RS08455]	lcl|NZ_LS483362.1_cds_WP_002864792.1_1638	1	549	549	reverse	1	549	549	1	549	[protein=50S ribosomal protein L3] [protein_id=WP_002864792.1] [location=complement(1619787..1620362)] [gbkey=CDS]	576	99.50%	54.90%
[locus_tag=DQM41_RS08555]	lcl|NZ_LS483362.1_cds_WP_002834281.1_1654	1	384	384	reverse	1	384	384	1	384	[gene=queF] [protein=NADPH-dependent 7-cyano-7-deazaguanine reductase QueF] [protein_id=WP_002834281.1] [location=complement(1634279..1634662)] [gbkey=CDS]	384	100.00%	38.40%
[locus_tag=DQM41_RS08560]	lcl|NZ_LS483362.1_cds_WP_002853070.1_1655	52	600	549	forward	52	600	549	52	600	[protein=NAD(P)/FAD-dependent oxidoreductase] [protein_id=WP_002853070.1] [location=1634822..1635421] [gbkey=CDS]	600	100.00%	54.90%
[locus_tag=DQM41_RS08575]	lcl|NZ_LS483362.1_cds_WP_002882660.1_1658	2020	2598	579	reverse	2020	2598	579	2020	2598	[gene=flgE] [protein=flagellar hook protein FlgE] [protein_id=WP_002882660.1] [location=complement(1638087..1640684)] [gbkey=CDS]	2598	98.40%	57.90%

Table S2: List of the Smash++ 1,000 bp segments, which presumably originate from *C. jejuni* of the isolate *C. coli* meC0281 with the corresponding locus tags of NCTC 11168.

locus_tag	Sequence Name	Minimum	Maximum	Length	Direction	Min (with gaps)	Max (with gaps)	Length (with gaps)	Min (original sequence)	Max (original sequence)	Description	Sequence Length	% Pairwise Identity	Query Coverage
[locus_tag=DQM41_RS00035]	lcl|NZ_LS483362.1_cds_WP_002853155.1_7	1042	2040	999	forward	1042	2040	999	1042	2040	[gene=gltB] [protein=glutamate synthase large subunit] [protein_id=WP_002853155.1] [location=8144..12634] [gbkey=CDS]	4491	97.30%	99.90%
[locus_tag=DQM41_RS00035]	lcl|NZ_LS483362.1_cds_WP_002853155.1_7	2041	3039	999	forward	2041	3039	999	2041	3039	[gene=gltB] [protein=glutamate synthase large subunit] [protein_id=WP_002853155.1] [location=8144..12634] [gbkey=CDS]	4491	98.50%	99.90%
[locus_tag=DQM41_RS00045]	lcl|NZ_LS483362.1_cds_WP_002853084.1_9	541	1446	906	forward	541	1446	906	541	1446	[protein=glutamate synthase subunit beta] [protein_id=WP_002853084.1] [location=14398..15843] [gbkey=CDS]	1446	87.60%	90.60%
[locus_tag=DQM41_RS00080]	lcl|NZ_LS483362.1_cds_WP_002779268.1_16	1	657	657	forward	1	657	657	1	657	[gene=queC] [protein=7-cyano-7-deazaguanine synthase QueC] [protein_id=WP_002779268.1] [location=21159..21833] [gbkey=CDS]	675	99.50%	65.70%
[locus_tag=DQM41_RS00120]	lcl|NZ_LS483362.1_cds_WP_002865087.1_24	904	1902	999	forward	904	1902	999	904	1902	[protein=ribonucleoside-diphosphate reductase subunit alpha] [protein_id=WP_002865087.1] [location=29726..32095] [gbkey=CDS]	2370	97.90%	99.90%
[locus_tag=DQM41_RS00120]	lcl|NZ_LS483362.1_cds_WP_002865087.1_24	1906	2370	465	forward	1906	2370	465	1906	2370	[protein=ribonucleoside-diphosphate reductase subunit alpha] [protein_id=WP_002865087.1] [location=29726..32095] [gbkey=CDS]	2370	98.70%	46.50%
[locus_tag=DQM41_RS00125]	lcl|NZ_LS483362.1_cds_WP_002865086.1_25	3	548	546	reverse	3	548	546	3	548	[protein=L-cystine transporter] [protein_id=WP_002865086.1] [location=complement(32134..33519)] [gbkey=CDS]	1386	96.20%	54.60%
[locus_tag=DQM41_RS00130]	lcl|NZ_LS483362.1_cds_WP_002853111.1_26	1	624	624	reverse	1	624	624	1	624	[protein=FAD-dependent thymidylate synthase] [protein_id=WP_002853111.1] [location=complement(33639..34262)] [gbkey=CDS]	624	100.00%	62.40%
[locus_tag=DQM41_RS00135]	lcl|NZ_LS483362.1_cds_WP_002853102.1_27	250	1245	996	forward	250	1245	996	250	1245	[protein=CTP synthase] [protein_id=WP_002853102.1] [location=34382..36013] [gbkey=CDS]	1632	99.70%	99.60%
[locus_tag=DQM41_RS00140]	lcl|NZ_LS483362.1_cds_WP_002853146.1_28	355	627	273	forward	355	627	273	355	627	[gene=recJ] [protein=single-stranded-DNA-specific exonuclease RecJ] [protein_id=WP_002853146.1] [location=36000..37571] [gbkey=CDS]	1572	100.00%	27.30%
[locus_tag=DQM41_RS00145]	lcl|NZ_LS483362.1_cds_WP_072233179.1_29	6	374	369	forward	6	374	369	6	374	[protein=type II asparaginase] [protein_id=WP_072233179.1] [location=37625..38662] [gbkey=CDS]	1038	96.00%	36.90%
[locus_tag=DQM41_RS00190]	lcl|NZ_LS483362.1_cds_WP_002866314.1_33	64	543	480	reverse	64	543	480	64	543	[protein=SIMPL domain-containing protein] [protein_id=WP_002866314.1] [location=complement(51963..52664)] [gbkey=CDS]	702	93.10%	48.00%
[locus_tag=DQM41_RS00200]	lcl|NZ_LS483362.1_cds_WP_010891829.1_35	553	1146	594	forward	553	1146	594	553	1146	[protein=DUF2130 domain-containing protein] [protein_id=WP_010891829.1] [location=53966..55315] [gbkey=CDS]	1350	96.50%	59.40%
[locus_tag=DQM41_RS00235]	lcl|NZ_LS483362.1_cds_WP_002805291.1_42	1072	1638	567	forward	1072	1638	567	1072	1638	[protein=flagellar hook protein FlgE] [protein_id=WP_002805291.1] [location=62227..63864] [gbkey=CDS]	1638	98.90%	56.70%
[locus_tag=DQM41_RS00245]	lcl|NZ_LS483362.1_cds_WP_025096273.1_44	166	717	552	reverse	166	717	552	166	717	[protein=bacteriohemerythrin] [protein_id=WP_025096273.1] [location=complement(65696..66463)] [gbkey=CDS]	768	92.90%	55.20%
[locus_tag=DQM41_RS00245]	lcl|NZ_LS483362.1_cds_WP_025096273.1_44	1	162	162	reverse	1	162	162	1	162	[protein=bacteriohemerythrin] [protein_id=WP_025096273.1] [location=complement(65696..66463)] [gbkey=CDS]	768	79.60%	16.20%
[locus_tag=DQM41_RS00285]	lcl|NZ_LS483362.1_cds_WP_002851977.1_51	1	456	456	reverse	1	456	456	1	456	[gene=mnmA] [protein=tRNA 2-thiouridine(34) synthase MnmA] [protein_id=WP_002851977.1] [location=complement(68528..69544)] [gbkey=CDS]	1017	99.30%	45.60%
[locus_tag=DQM41_RS00305]	lcl|NZ_LS483362.1_cds_WP_002864362.1_55	91	849	759	forward	91	849	759	91	849	[protein=hypothetical protein] [protein_id=WP_002864362.1] [location=72001..72855] [gbkey=CDS]	855	96.80%	75.90%
[locus_tag=DQM41_RS00315]	lcl|NZ_LS483362.1_cds_WP_002851810.1_57	241	843	603	reverse	241	843	603	241	843	[gene=fliY] [protein=flagellar motor switch protein FliY] [protein_id=WP_002851810.1] [location=complement(73488..74330)] [gbkey=CDS]	843	99.00%	60.30%
[locus_tag=DQM41_RS00320]	lcl|NZ_LS483362.1_cds_WP_002851710.1_58	316	978	663	reverse	316	978	663	316	978	[gene=fliM] [protein=flagellar motor switch protein FliM] [protein_id=WP_002851710.1] [location=complement(74327..75406)] [gbkey=CDS]	1080	95.20%	66.30%
[locus_tag=DQM41_RS00440]	lcl|NZ_LS483362.1_cds_WP_002852014.1_82	130	993	864	forward	130	993	864	130	993	[protein=anaerobic C4-dicarboxylate transporter] [protein_id=WP_002852014.1] [location=97492..98829] [gbkey=CDS]	1338	90.30%	86.40%
[locus_tag=DQM41_RS00830]	lcl|NZ_LS483362.1_cds_WP_002840050.1_160	13	813	801	forward	13	813	801	13	813	[protein=iron-sulfur cluster assembly scaffold protein NifU] [protein_id=WP_002840050.1] [location=164625..165596] [gbkey=CDS]	972	99.60%	80.10%
[locus_tag=DQM41_RS01090]	lcl|NZ_LS483362.1_cds_WP_002857636.1_212	1	762	762	reverse	1	762	762	1	762	[protein=MotA/TolQ/ExbB proton channel family protein] [protein_id=WP_002857636.1] [location=complement(210706..211467)] [gbkey=CDS]	762	91.90%	76.20%
[locus_tag=DQM41_RS01160]	lcl|NZ_LS483362.1_cds_WP_002851982.1_225	1	582	582	reverse	1	582	582	1	582	[gene=miaA] [protein=tRNA (adenosine(37)-N6)-dimethylallyltransferase MiaA] [protein_id=WP_002851982.1] [location=complement(222479..223348)] [gbkey=CDS]	870	83.50%	58.20%
[locus_tag=DQM41_RS01230]	lcl|NZ_LS483362.1_cds_WP_002851994.1_239	1	807	807	forward	1	807	807	1	807	[protein=hypothetical protein] [protein_id=WP_002851994.1] [location=231820..232626] [gbkey=CDS]	807	75.10%	80.70%
[locus_tag=DQM41_RS01295]	lcl|NZ_LS483362.1_cds_WP_002858687.1_252	79	777	699	forward	79	777	699	79	777	[protein=membrane protein] [protein_id=WP_002858687.1] [location=complement(246549..247637)] [gbkey=CDS]	1089	92.00%	69.90%
[locus_tag=DQM41_RS01370]	lcl|NZ_LS483362.1_cds_WP_002851649.1_267	457	1119	663	forward	457	1119	663	457	1119	[protein=response regulator] [protein_id=WP_002851649.1] [location=complement(260898..263207)] [gbkey=CDS]	2310	81.00%	66.30%
[locus_tag=DQM41_RS01410]	lcl|NZ_LS483362.1_cds_WP_002854555.1_274	1	777	777	reverse	1	777	777	1	777	[protein=5'/3'-nucleotidase SurE] [protein_id=WP_002854555.1] [location=269058..269834] [gbkey=CDS]	777	88.70%	77.70%
[locus_tag=DQM41_RS01430]	lcl|NZ_LS483362.1_cds_WP_002778123.1_278	405	848	444	forward	405	848	444	405	848	[protein=pantoate--beta-alanine ligase] [protein_id=WP_002778123.1] [location=complement(271412..272260)] [gbkey=CDS]	849	99.30%	44.40%
[locus_tag=DQM41_RS01440]	lcl|NZ_LS483362.1_cds_WP_002783228.1_280	1	543	543	reverse	1	543	543	1	543	[protein=OXA-61 family class D beta-lactamase OXA-193] [protein_id=WP_002783228.1] [location=273315..274088] [gbkey=CDS]	774	100.00%	54.30%
[locus_tag=DQM41_RS01445]	lcl|NZ_LS483362.1_cds_WP_002816233.1_281	202	885	684	forward	202	885	684	202	885	[protein=sulfate/molybdate ABC transporter ATP-binding protein] [protein_id=WP_002816233.1] [location=complement(274173..275057)] [gbkey=CDS]	885	75.50%	68.40%
[locus_tag=DQM41_RS01495]	lcl|NZ_LS483362.1_cds_WP_002783219.1_291	3	338	336	forward	3	338	336	3	338	[protein=multidrug efflux SMR transporter] [protein_id=WP_002783219.1] [location=complement(281623..281961)] [gbkey=CDS]	339	100.00%	33.60%
[locus_tag=DQM41_RS01525]	lcl|NZ_LS483362.1_cds_WP_002858741.1_297	7	918	912	reverse	7	918	912	7	918	[gene=pheA] [protein=prephenate dehydratase] [protein_id=WP_002858741.1] [location=286242..287315] [gbkey=CDS]	1074	94.70%	91.20%
[locus_tag=DQM41_RS01545]	lcl|NZ_LS483362.1_cds_WP_002858748.1_301	388	447	60	reverse	388	447	60	388	447	[gene=fliH] [protein=flagellar assembly protein FliH] [protein_id=WP_002858748.1] [location=291169..291999] [gbkey=CDS]	831	55.00%	6.00%
[locus_tag=DQM41_RS01615]	lcl|NZ_LS483362.1_cds_WP_002854219.1_315	1	597	597	reverse	1	597	597	1	597	[protein=peroxiredoxin] [protein_id=WP_002854219.1] [location=302377..302973] [gbkey=CDS]	597	97.50%	59.70%
[locus_tag=DQM41_RS01765]	lcl|NZ_LS483362.1_cds_WP_002858696.1_345	1	930	930	forward	1	930	930	1	930	[gene=cmeC] [protein=multidrug efflux transporter outer membrane subunit CmeC] [protein_id=WP_002858696.1] [location=complement(331118..332596)] [gbkey=CDS]	1479	89.40%	93.00%
[locus_tag=DQM41_RS01770]	lcl|NZ_LS483362.1_cds_WP_002872052.1_346	85	1044	960	forward	85	1044	960	85	1044	[gene=cmeB] [protein=multidrug efflux RND transporter permease subunit CmeB] [protein_id=WP_002872052.1] [location=complement(332589..335711)] [gbkey=CDS]	3123	95.20%	96.00%
[locus_tag=DQM41_RS01770]	lcl|NZ_LS483362.1_cds_WP_002872052.1_346	1048	2046	999	forward	1048	2046	999	1048	2046	[gene=cmeB][protein=multidrug efflux RND transporter permease subunit CmeB] [protein_id=WP_002872052.1] [location=complement(332589..335711)] [gbkey=CDS]	3123	86.40%	99.90%
[locus_tag=DQM41_RS01850]	lcl|NZ_LS483362.1_cds_WP_002858700.1_362	160	828	669	forward	160	828	669	160	828	[gene=pyrF] [protein=orotidine-5'-phosphate decarboxylase] [protein_id=WP_002858700.1] [location=complement(347898..348737)] [gbkey=CDS]	840	88.80%	66.90%
[locus_tag=DQM41_RS01955]	lcl|NZ_LS483362.1_cds_WP_002858710.1_383	361	1245	885	reverse	361	1245	885	361	1245	[protein=serine hydroxymethyltransferase] [protein_id=WP_002858710.1] [location=367212..368456] [gbkey=CDS]	1245	88.80%	88.50%
[locus_tag=DQM41_RS02050]	lcl|NZ_LS483362.1_cds_WP_002858716.1_402	490	957	468	forward	490	957	468	490	957	[protein=membrane protein] [protein_id=WP_002858716.1] [location=complement(385765..386721)] [gbkey=CDS]	957	91.40%	46.80%
[locus_tag=DQM41_RS02355]	lcl|NZ_LS483362.1_cds_WP_002851098.1_453	7	519	513	reverse	7	519	513	7	519	[gene=nusG] [protein=transcription termination/antitermination protein NusG] [protein_id=WP_002851098.1] [location=436108..436641] [gbkey=CDS]	534	99.40%	51.30%
[locus_tag=DQM41_RS02380]	lcl|NZ_LS483362.1_cds_WP_010891860.1_458	2131	3129	999	reverse	2131	3129	999	2131	3129	[gene=rpoB] [protein=DNA-directed RNA polymerase subunit beta] [protein_id=WP_010891860.1] [location=438986..443122] [gbkey=CDS]	4137	99.70%	99.90%
[locus_tag=DQM41_RS02380]	lcl|NZ_LS483362.1_cds_WP_010891860.1_458	1132	2127	996	reverse	1132	2127	996	1132	2127	[gene=rpoB] [protein=DNA-directed RNA polymerase subunit beta] [protein_id=WP_010891860.1] [location=438986..443122] [gbkey=CDS]	4137	98.00%	99.60%
[locus_tag=DQM41_RS02385]	lcl|NZ_LS483362.1_cds_WP_002858508.1_459	4006	4554	549	reverse	4006	4554	549	4006	4554	[gene=rpoC] [protein=DNA-directed RNA polymerase subunit beta'] [protein_id=WP_002858508.1] [location=443115..447668] [gbkey=CDS]	4554	96.70%	54.90%
[locus_tag=DQM41_RS02385]	lcl|NZ_LS483362.1_cds_WP_002858508.1_459	2002	3000	999	reverse	2002	3000	999	2002	3000	[gene=rpoC] [protein=DNA-directed RNA polymerase subunit beta'] [protein_id=WP_002858508.1] [location=443115..447668] [gbkey=CDS]	4554	95.80%	99.90%
[locus_tag=DQM41_RS02385]	lcl|NZ_LS483362.1_cds_WP_002858508.1_459	1	999	999	reverse	1	999	999	1	999	[gene=rpoC] [protein=DNA-directed RNA polymerase subunit beta'] [protein_id=WP_002858508.1] [location=443115..447668] [gbkey=CDS]	4554	86.70%	99.90%
[locus_tag=DQM41_RS02395]	lcl|NZ_LS483362.1_cds_WP_002858487.1_461	433	909	477	reverse	433	909	477	433	909	[protein=dihydrodipicolinate synthase family protein] [protein_id=WP_002858487.1] [location=448691..449599] [gbkey=CDS]	909	98.10%	47.70%
[locus_tag=DQM41_RS02405]	lcl|NZ_LS483362.1_cds_WP_025096279.1_463	262	1167	906	reverse	262	1167	906	262	1167	[protein=UxaA family hydrolase] [protein_id=WP_025096279.1] [location=449862..451028] [gbkey=CDS]	1167	96.70%	90.60%
[locus_tag=DQM41_RS02420]	lcl|NZ_LS483362.1_cds_WP_002864166.1_466	1	561	561	reverse	1	561	561	1	561	[gene=fucP] [protein=L-fucose:H+ symporter permease] [protein_id=WP_002864166.1] [location=453113..454369] [gbkey=CDS]	1257	89.10%	56.10%
[locus_tag=DQM41_RS02445]	lcl|NZ_LS483362.1_cds_WP_002779471.1_471	184	471	288	reverse	184	471	288	184	471	[gene=rpsG] [protein=30S ribosomal protein S7] [protein_id=WP_002779471.1] [location=457677..458147] [gbkey=CDS]	471	100.00%	28.80%
[locus_tag=DQM41_RS02485]	lcl|NZ_LS483362.1_cds_WP_002858543.1_478	40	138	99	reverse	40	138	99	40	138	[protein=HIT domain-containing protein] [protein_id=WP_002858543.1] [location=463611..464096] [gbkey=CDS]	486	27.30%	9.90%
[locus_tag=DQM41_RS02500]	lcl|NZ_LS483362.1_cds_WP_002858523.1_481	334	912	579	forward	334	912	579	334	912	[protein=ferrochelatase] [protein_id=WP_002858523.1] [location=complement(466654..467565)] [gbkey=CDS]	912	84.10%	57.90%
[locus_tag=DQM41_RS02540]	lcl|NZ_LS483362.1_cds_WP_002856815.1_489	211	1206	996	reverse	211	1206	996	211	1206	[protein=S41 family peptidase] [protein_id=WP_002856815.1] [location=477749..479083] [gbkey=CDS]	1335	96.10%	99.60%
[locus_tag=DQM41_RS02620]	lcl|NZ_LS483362.1_cds_WP_002858492.1_505	1	432	432	forward	1	432	432	1	432	[gene=flgB] [protein=flagellar basal body rod protein FlgB] [protein_id=WP_002858492.1] [location=complement(489956..490387)] [gbkey=CDS]	432	97.20%	43.20%
[locus_tag=DQM41_RS02710]	lcl|NZ_LS483362.1_cds_WP_002865770.1_523	741	1736	996	reverse	741	1736	996	741	1736	[protein=menaquinone biosynthesis decarboxylase] [protein_id=WP_002865770.1] [location=508218..510020] [gbkey=CDS]	1803	99.10%	99.60%
[locus_tag=DQM41_RS02720]	lcl|NZ_LS483362.1_cds_WP_002864504.1_525	412	1410	999	reverse	412	1410	999	412	1410	[gene=fliD] [protein=flagellar filament capping protein FliD] [protein_id=WP_002864504.1] [location=510546..512474] [gbkey=CDS]	1929	96.10%	99.90%
[locus_tag=DQM41_RS02780]	lcl|NZ_LS483362.1_cds_WP_002856867.1_536	286	936	651	reverse	286	936	651	286	936	[protein=NAD(P)-binding domain-containing protein] [protein_id=WP_002856867.1] [location=521536..522471] [gbkey=CDS]	936	93.50%	65.10%
[locus_tag=DQM41_RS02930]	lcl|NZ_LS483362.1_cds_WP_002852062.1_564	409	1407	999	forward	409	1407	999	409	1407	[gene=ligA] [protein=NAD-dependent DNA ligase LigA] [protein_id=WP_002852062.1] [location=545324..547267] [gbkey=CDS]	1944	88.30%	99.90%
[locus_tag=DQM41_RS02935]	lcl|NZ_LS483362.1_cds_WP_002852264.1_565	524	604	81	reverse	524	604	81	524	604	[protein=membrane protein] [protein_id=WP_002852264.1] [location=547248..548252] [gbkey=CDS]	1005	40.70%	8.10%
[locus_tag=DQM41_RS03075]	lcl|NZ_LS483362.1_cds_WP_002864528.1_593	138	1088	951	reverse	138	1088	951	138	1088	[gene=pstA] [protein=phosphate ABC transporter permease PstA] [protein_id=WP_002864528.1] [location=575131..576219] [gbkey=CDS]	1089	91.90%	95.10%
[locus_tag=DQM41_RS03080]	lcl|NZ_LS483362.1_cds_WP_010891867.1_594	52	741	690	reverse	52	741	690	52	741	[protein=phosphate ABC transporter ATP-binding protein] [protein_id=WP_010891867.1] [location=576216..576956] [gbkey=CDS]	741	99.60%	69.00%
[locus_tag=DQM41_RS03090]	lcl|NZ_LS483362.1_cds_WP_002906848.1_596	276	929	654	reverse	276	929	654	276	929	[protein=MATE family efflux transporter] [protein_id=WP_002906848.1] [location=578335..579651] [gbkey=CDS]	1317	93.10%	65.40%
[locus_tag=DQM41_RS03100]	lcl|NZ_LS483362.1_cds_WP_002826526.1_598	1	948	948	reverse	1	948	948	1	948	[protein=hypothetical protein] [protein_id=WP_002826526.1] [location=580316..581836] [gbkey=CDS]	1521	98.70%	94.80%
[locus_tag=DQM41_RS03110]	lcl|NZ_LS483362.1_cds_WP_002776330.1_600	187	744	558	reverse	187	744	558	187	744	[gene=hypB] [protein=hydrogenase nickel incorporation protein HypB] [protein_id=WP_002776330.1] [location=584084..584827] [gbkey=CDS]	744	99.50%	55.80%
[locus_tag=DQM41_RS03120]	lcl|NZ_LS483362.1_cds_WP_002776343.1_602	178	1092	915	reverse	178	1092	915	178	1092	[gene=hypD] [protein=hydrogenase formation protein HypD] [protein_id=WP_002776343.1] [location=585092..586183] [gbkey=CDS]	1092	99.70%	91.50%
[locus_tag=DQM41_RS03145]	lcl|NZ_LS483362.1_cds_WP_010891871.1_607	232	1230	999	forward	232	1230	999	232	1230	[protein=VacB/RNase II family 3'-5' exoribonuclease] [protein_id=WP_010891871.1] [location=complement(592485..594419)] [gbkey=CDS]	1935	91.30%	99.90%
[locus_tag=DQM41_RS03640]	lcl|NZ_LS483362.1_cds_WP_002793291.1_706	1	948	948	reverse	1	948	948	1	948	[protein=esterase-like activity of phytase family protein] [protein_id=WP_002793291.1] [location=682515..683840] [gbkey=CDS]	1326	99.40%	94.80%
[locus_tag=DQM41_RS03650]	lcl|NZ_LS483362.1_cds_WP_002784190.1_708	1	630	630	reverse	1	630	630	1	630	[protein=ABC transporter permease] [protein_id=WP_002784190.1] [location=684643..685482] [gbkey=CDS]	840	98.60%	63.00%
[locus_tag=DQM41_RS03670]	lcl|NZ_LS483362.1_cds_WP_002858622.1_712	208	756	549	forward	208	756	549	208	756	[protein=basic amino acid ABC transporter substrate-binding protein] [protein_id=WP_002858622.1] [location=complement(687913..688668)] [gbkey=CDS]	756	98.40%	54.90%
[locus_tag=DQM41_RS03750]	lcl|NZ_LS483362.1_cds_WP_010891883.1_723	841	1836	996	reverse	841	1836	996	841	1836	[gene=cfrA] [protein=TonB-dependent ferric enterobactin receptor CfrA] [protein_id=WP_010891883.1] [location=705435..707525] [gbkey=CDS]	2091	99.70%	99.60%
[locus_tag=DQM41_RS03760]	lcl|NZ_LS483362.1_cds_WP_002780052.1_725	1	531	531	reverse	1	531	531	1	531	[gene=grpE] [protein=nucleotide exchange factor GrpE] [protein_id=WP_002780052.1] [location=708616..709146] [gbkey=CDS]	531	91.30%	53.10%
[locus_tag=DQM41_RS03765]	lcl|NZ_LS483362.1_cds_WP_002826316.1_726	106	1104	999	reverse	106	1104	999	106	1104	[gene=dnaK] [protein=molecular chaperone DnaK] [protein_id=WP_002826316.1] [location=709168..711039] [gbkey=CDS]	1872	98.20%	99.90%
[locus_tag=DQM41_RS03775]	lcl|NZ_LS483362.1_cds_WP_002852439.1_728	226	1017	792	reverse	226	1017	792	226	1017	[protein=MBL fold metallo-hydrolase] [protein_id=WP_002852439.1] [location=711299..712321] [gbkey=CDS]	1023	87.90%	79.20%
[locus_tag=DQM41_RS03885]	lcl|NZ_LS483362.1_cds_WP_010891886.1_750	319	771	453	reverse	319	771	453	319	771	[gene=napH] [protein=quinol dehydrogenase ferredoxin subunit NapH] [protein_id=WP_010891886.1] [location=735693..736475] [gbkey=CDS]	783	81.50%	45.30%
[locus_tag=DQM41_RS03945]	lcl|NZ_LS483362.1_cds_WP_002864146.1_762	910	1281	372	reverse	910	1281	372	910	1281	[protein=hypothetical protein] [protein_id=WP_002864146.1] [location=744480..745760] [gbkey=CDS]	1281	80.60%	37.20%
[locus_tag=DQM41_RS03950]	lcl|NZ_LS483362.1_cds_WP_002852462.1_763	388	1386	999	forward	388	1386	999	388	1386	[protein=UDP-N-acetylmuramoyl-tripeptide--D-alanyl-D-alanine ligase] [protein_id=WP_002852462.1] [location=complement(745818..747245)] [gbkey=CDS]	1428	77.10%	99.90%
[locus_tag=DQM41_RS04040]	lcl|NZ_LS483362.1_cds_WP_002852502.1_781	316	720	405	reverse	316	720	405	316	720	[gene=kdsB] [protein=3-deoxy-manno-octulosonate cytidylyltransferase] [protein_id=WP_002852502.1] [location=764464..765183] [gbkey=CDS]	720	80.00%	40.50%
[locus_tag=DQM41_RS04055]	lcl|NZ_LS483362.1_cds_WP_002858779.1_784	78	770	693	reverse	78	770	693	78	770	[protein=transporter substrate-binding domain-containing protein] [protein_id=WP_002858779.1] [location=766676..767446] [gbkey=CDS]	771	100.00%	69.30%
[locus_tag=DQM41_RS04215]	lcl|NZ_LS483362.1_cds_816	1392	1991	600	forward	1392	1991	600	1392	1991	[protein=flagellar hook-length control protein FliK] [pseudo=true] [location=complement(795485..797643)] [gbkey=CDS]	2159	94.00%	60.00%
[locus_tag=DQM41_RS04220]	lcl|NZ_LS483362.1_cds_WP_002878829.1_817	195	278	84	reverse	195	278	84	195	278	[protein=MFS transporter] [protein_id=WP_002878829.1] [location=complement(797636..798823)] [gbkey=CDS]	1188	35.70%	8.40%
[locus_tag=DQM41_RS04280]	lcl|NZ_LS483362.1_cds_WP_002864843.1_829	1441	1509	69	forward	1441	1509	69	1441	1509	[protein=chorismate-binding protein] [protein_id=WP_002864843.1] [location=complement(807480..809264)] [gbkey=CDS]	1785	47.80%	6.90%
[locus_tag=DQM41_RS04280]	lcl|NZ_LS483362.1_cds_WP_002864843.1_829	1441	1509	69	reverse	1441	1509	69	1441	1509	[protein=chorismate-binding protein] [protein_id=WP_002864843.1] [location=complement(807480..809264)] [gbkey=CDS]	1785	47.80%	6.90%
[locus_tag=DQM41_RS04280]	lcl|NZ_LS483362.1_cds_WP_002864843.1_829	1441	1509	69	reverse	1441	1509	69	1441	1509	[protein=chorismate-binding protein] [protein_id=WP_002864843.1] [location=complement(807480..809264)] [gbkey=CDS]	1785	47.80%	6.90%
[locus_tag=DQM41_RS04345]	lcl|NZ_LS483362.1_cds_WP_002852599.1_842	1288	2175	888	reverse	1288	2175	888	1288	2175	[gene=flhA] [protein=flagellar biosynthesis protein FlhA] [protein_id=WP_002852599.1] [location=complement(817562..819736)] [gbkey=CDS]	2175	99.00%	88.80%
[locus_tag=DQM41_RS04545]	lcl|NZ_LS483362.1_cds_WP_002853259.1_880	199	729	531	forward	199	729	531	199	729	[protein=amino acid ABC transporter ATP-binding protein] [protein_id=WP_002853259.1] [location=complement(857882..858610)] [gbkey=CDS]	729	94.40%	53.10%
[locus_tag=DQM41_RS04985]	lcl|NZ_LS483362.1_cds_WP_002852990.1_960	424	1242	819	forward	424	1242	819	424	1242	[protein=ATP-dependent metallopeptidase FtsH/Yme1/Tma family protein] [protein_id=WP_002852990.1] [location=complement(934608..936224)] [gbkey=CDS]	1617	97.10%	81.90%
[locus_tag=DQM41_RS05110]	lcl|NZ_LS483362.1_cds_WP_002852941.1_984	46	645	600	forward	46	645	600	46	645	[protein=hypothetical protein] [protein_id=WP_002852941.1] [location=complement(960817..961461)] [gbkey=CDS]	645	78.00%	60.00%
[locus_tag=DQM41_RS05115]	lcl|NZ_LS483362.1_cds_WP_002837927.1_985	811	1647	837	forward	811	1647	837	811	1647	[gene=lepA][protein=elongation factor 4] [protein_id=WP_002837927.1] [location=complement(961538..963334)] [gbkey=CDS]	1797	95.20%	83.70%
[locus_tag=DQM41_RS05125]	lcl|NZ_LS483362.1_cds_WP_002858463.1_987	1	741	741	reverse	1	741	741	1	741	[gene=cmeE] [protein=multidrug efflux RND transporter periplasmic adaptor subunit CmeE] [protein_id=WP_002858463.1] [location=964732..965472] [gbkey=CDS]	741	97.60%	74.10%
[locus_tag=DQM41_RS05130]	lcl|NZ_LS483362.1_cds_WP_010891901.1_988	2217	2615	399	reverse	2217	2615	399	2217	2615	[gene=cmeF] [protein=multidrug efflux RND transporter permease subunit CmeF] [protein_id=WP_010891901.1] [location=965475..968492] [gbkey=CDS]	3018	100.00%	39.90%
[locus_tag=DQM41_RS05130]	lcl|NZ_LS483362.1_cds_WP_010891901.1_988	1216	2214	999	reverse	1216	2214	999	1216	2214	[gene=cmeF] [protein=multidrug efflux RND transporter permease subunit CmeF] [protein_id=WP_010891901.1] [location=965475..968492] [gbkey=CDS]	3018	98.50%	99.90%
[locus_tag=DQM41_RS05130]	lcl|NZ_LS483362.1_cds_WP_010891901.1_988	217	1173	957	reverse	217	1173	957	217	1173	[gene=cmeF] [protein=multidrug efflux RND transporter permease subunit CmeF] [protein_id=WP_010891901.1] [location=965475..968492] [gbkey=CDS]	3018	98.10%	95.70%
[locus_tag=DQM41_RS05220]	lcl|NZ_LS483362.1_cds_WP_002864285.1_1006	466	1464	999	forward	466	1464	999	466	1464	[protein=N-6 DNA methylase] [protein_id=WP_002864285.1] [location=complement(982973..986992)] [gbkey=CDS]	4020	99.70%	99.90%
[locus_tag=DQM41_RS05220]	lcl|NZ_LS483362.1_cds_WP_002864285.1_1006	1465	2463	999	forward	1465	2463	999	1465	2463	[protein=N-6 DNA methylase] [protein_id=WP_002864285.1] [location=complement(982973..986992)] [gbkey=CDS]	4020	98.20%	99.90%
[locus_tag=DQM41_RS05220]	lcl|NZ_LS483362.1_cds_WP_002864285.1_1006	3391	4020	630	forward	3391	4020	630	3391	4020	[protein=N-6 DNA methylase] [protein_id=WP_002864285.1] [location=complement(982973..986992)] [gbkey=CDS]	4020	93.60%	63.00%
[locus_tag=DQM41_RS05225]	lcl|NZ_LS483362.1_cds_WP_010891903.1_1007	685	1500	816	forward	685	1500	816	685	1500	[protein=endonuclease MutS2] [protein_id=WP_010891903.1] [location=complement(987001..989211)] [gbkey=CDS]	2211	100.00%	81.60%
[locus_tag=DQM41_RS05610]	lcl|NZ_LS483362.1_cds_WP_002858308.1_1084	841	1332	492	forward	841	1332	492	841	1332	[gene=pglK] [protein=ABC-type lipopolysaccharide transporter PglK] [protein_id=WP_002858308.1] [location=complement(1063189..1064883)] [gbkey=CDS]	1695	80.50%	49.20%
[locus_tag=DQM41_RS05780]	lcl|NZ_LS483362.1_cds_WP_002852826.1_1118	1	777	777	forward	1	777	777	1	777	[protein=threonine/serine exporter ThrE family protein] [protein_id=WP_002852826.1] [location=complement(1095911..1096687)] [gbkey=CDS]	777	88.00%	77.70%
[locus_tag=DQM41_RS05785]	lcl|NZ_LS483362.1_cds_WP_002864828.1_1119	1	927	927	reverse	1	927	927	1	927	[protein=L-lactate dehydrogenase] [protein_id=WP_002864828.1] [location=1096754..1097680] [gbkey=CDS]	927	93.20%	92.70%
[locus_tag=DQM41_RS05805]	lcl|NZ_LS483362.1_cds_WP_002869004.1_1123	3	482	480	forward	3	482	480	3	482	[protein=peptidylprolyl isomerase] [protein_id=WP_002869004.1] [location=complement(1100175..1100657)] [gbkey=CDS]	483	93.10%	48.00%
[locus_tag=DQM41_RS05825]	lcl|NZ_LS483362.1_cds_WP_010891916.1_1127	1078	1593	516	forward	1078	1593	516	1078	1593	[protein=arginine--tRNA ligase] [protein_id=WP_010891916.1] [location=complement(1102150..1103742)] [gbkey=CDS]	1593	97.10%	51.60%
[locus_tag=DQM41_RS05845]	lcl|NZ_LS483362.1_cds_WP_002852712.1_1131	322	762	441	forward	322	762	441	322	762	[gene=fliR] [protein=flagellar type III secretion system protein FliR] [protein_id=WP_002852712.1] [location=complement(1106300..1107067)] [gbkey=CDS]	768	79.00%	44.10%
[locus_tag=DQM41_RS05850]	lcl|NZ_LS483362.1_cds_WP_010891917.1_1132	1	636	636	forward	1	636	636	1	636	[protein=ABC transporter ATP-binding protein] [protein_id=WP_010891917.1] [location=complement(1107054..1107689)] [gbkey=CDS]	636	88.70%	63.60%
[locus_tag=DQM41_RS05895]	lcl|NZ_LS483362.1_cds_WP_002777340.1_1141	1	498	498	forward	1	498	498	1	498	[gene=cetB] [protein=bipartate energy taxis response protein CetB] [protein_id=WP_002777340.1] [location=complement(1117173..1117670)] [gbkey=CDS]	498	98.80%	49.80%
[locus_tag=DQM41_RS05900]	lcl|NZ_LS483362.1_cds_WP_002790076.1_1142	817	1296	480	forward	817	1296	480	817	1296	[gene=cetA] [protein=bipartate energy taxis response protein CetA] [protein_id=WP_002790076.1] [location=complement(1117688..1119067)] [gbkey=CDS]	1380	98.80%	48.00%
[locus_tag=DQM41_RS05905]	lcl|NZ_LS483362.1_cds_WP_002855880.1_1143	1	486	486	forward	1	486	486	1	486	[gene=cetC] [protein=energy taxis response protein CetC] [protein_id=WP_002855880.1] [location=complement(1119188..1119682)] [gbkey=CDS]	495	97.50%	48.60%
[locus_tag=DQM41_RS05910]	lcl|NZ_LS483362.1_cds_WP_002852850.1_1144	817	1377	561	reverse	817	1377	561	817	1377	[protein=cation:dicarboxylase symporter family transporter] [protein_id=WP_002852850.1] [location=1119951..1121327] [gbkey=CDS]	1377	100.00%	56.10%
[locus_tag=DQM41_RS05910]	lcl|NZ_LS483362.1_cds_WP_002852850.1_1144	46	813	768	reverse	46	813	768	46	813	[protein=cation:dicarboxylase symporter family transporter] [protein_id=WP_002852850.1] [location=1119951..1121327] [gbkey=CDS]	1377	94.70%	76.80%
[locus_tag=DQM41_RS06130]	lcl|NZ_LS483362.1_cds_WP_002857898.1_1187	52	822	771	reverse	52	822	771	52	822	[protein=M23 family metallopeptidase] [protein_id=WP_002857898.1] [location=1163782..1164603] [gbkey=CDS]	822	87.20%	77.10%
[locus_tag=DQM41_RS06320]	lcl|NZ_LS483362.1_cds_WP_002853464.1_1225	107	187	81	forward	107	187	81	107	187	[protein=DNA-directed RNA polymerase subunit omega] [protein_id=WP_002853464.1] [location=complement(1206758..1206982)] [gbkey=CDS]	225	44.40%	8.10%
[locus_tag=DQM41_RS06420]	lcl|NZ_LS483362.1_cds_WP_002854086.1_1243	61	552	492	reverse	61	552	492	61	552	[protein=dCTP deaminase] [protein_id=WP_002854086.1] [location=1224222..1224782] [gbkey=CDS]	561	98.80%	49.20%
[locus_tag=DQM41_RS06470]	lcl|NZ_LS483362.1_cds_WP_002789842.1_1253	118	1062	945	reverse	118	1062	945	118	1062	[protein=beta-ketoacyl-ACP synthase III] [protein_id=WP_002789842.1] [location=1232979..1234040] [gbkey=CDS]	1062	99.40%	94.50%
[locus_tag=DQM41_RS06490]	lcl|NZ_LS483362.1_cds_WP_002858432.1_1257	1	636	636	forward	1	636	636	1	636	[protein=DUF2920 family protein] [protein_id=WP_002858432.1] [location=complement(1235506..1236732)] [gbkey=CDS]	1227	95.50%	63.60%
[locus_tag=DQM41_RS06490]	lcl|NZ_LS483362.1_cds_WP_002858432.1_1257	637	1227	591	forward	637	1227	591	637	1227	[protein=DUF2920 family protein] [protein_id=WP_002858432.1] [location=complement(1235506..1236732)] [gbkey=CDS]	1227	99.50%	59.10%
[locus_tag=DQM41_RS06490]	lcl|NZ_LS483362.1_cds_WP_002858432.1_1257	733	1221	489	forward	733	1221	489	733	1221	[protein=DUF2920 family protein] [protein_id=WP_002858432.1] [location=complement(1235506..1236732)] [gbkey=CDS]	1227	86.50%	48.90%
[locus_tag=DQM41_RS06505]	lcl|NZ_LS483362.1_cds_WP_002856137.1_1260	280	936	657	forward	280	936	657	280	936	[protein=hypothetical protein] [protein_id=WP_002856137.1] [location=complement(1238565..1239635)] [gbkey=CDS]	1071	99.10%	65.70%
[locus_tag=DQM41_RS06515]	lcl|NZ_LS483362.1_cds_1262	2	580	579	forward	2	580	579	2	580	[protein=DUF2920 family protein] [pseudo=true] [location=complement(1240216..1240870)] [gbkey=CDS]	655	96.90%	57.90%
[locus_tag=DQM41_RS06570]	lcl|NZ_LS483362.1_cds_WP_010891923.1_1273	703	1155	453	reverse	703	1155	453	703	1155	[gene=legC] [protein=aminotransferase LegC] [protein_id=WP_010891923.1] [location=1249570..1250724] [gbkey=CDS]	1155	99.30%	45.30%
[locus_tag=DQM41_RS06590]	lcl|NZ_LS483362.1_cds_WP_002788888.1_1277	1	675	675	reverse	1	675	675	1	675	[protein=methyltransferase domain-containing protein] [protein_id=WP_002788888.1] [location=1253399..1254073] [gbkey=CDS]	675	99.60%	67.50%
[locus_tag=DQM41_RS06600]	lcl|NZ_LS483362.1_cds_WP_002858369.1_1279	193	1146	954	reverse	193	1146	954	193	1146	[gene=legG] [protein=GDP-N,N'-diacetylbacillosamine 2-epimerase (hydrolyzing)] [protein_id=WP_002858369.1] [location=1255110..1256264] [gbkey=CDS]	1155	92.20%	95.40%
[locus_tag=DQM41_RS06605]	lcl|NZ_LS483362.1_cds_WP_002864264.1_1280	1	984	984	reverse	1	984	984	1	984	[protein=CBS domain-containing protein] [protein_id=WP_002864264.1] [location=1256273..1257298] [gbkey=CDS]	1026	95.00%	98.40%
[locus_tag=DQM41_RS06610]	lcl|NZ_LS483362.1_cds_WP_002860798.1_1281	1	906	906	reverse	1	906	906	1	906	[gene=ptmF] [protein=L-glutamine-D-fructose-6-phosphate isomerase subunit] [protein_id=WP_002860798.1] [location=1257295..1258200] [gbkey=CDS]	906	93.40%	90.60%
[locus_tag=DQM41_RS06625]	lcl|NZ_LS483362.1_cds_WP_010891924.1_1284	124	789	666	reverse	124	789	666	124	789	[gene=pseD] [protein=flagellin modification protein PseD] [protein_id=WP_010891924.1] [location=1259658..1261619] [gbkey=CDS]	1962	89.20%	66.60%
[locus_tag=DQM41_RS06650]	lcl|NZ_LS483362.1_cds_WP_002852395.1_1289	1	798	798	forward	1	798	798	1	798	[protein=flagellin A] [protein_id=WP_002852395.1] [location=complement(1267350..1269068)] [gbkey=CDS]	1719	99.20%	79.80%
[locus_tag=DQM41_RS06650]	lcl|NZ_LS483362.1_cds_WP_002852395.1_1289	1282	1719	438	forward	1282	1719	438	1282	1719	[protein=flagellin A] [protein_id=WP_002852395.1] [location=complement(1267350..1269068)] [gbkey=CDS]	1719	97.30%	43.80%
[locus_tag=DQM41_RS06655]	lcl|NZ_LS483362.1_cds_WP_010891927.1_1290	673	1191	519	forward	673	1191	519	673	1191	[protein=flagellin A] [protein_id=WP_010891927.1] [location=complement(1269214..1270932)] [gbkey=CDS]	1719	89.60%	51.90%
[locus_tag=DQM41_RS06660]	lcl|NZ_LS483362.1_cds_WP_010891928.1_1291	586	1584	999	forward	586	1584	999	586	1584	[protein=motility associated factor glycosyltransferase family protein] [protein_id=WP_010891928.1] [location=complement(1271029..1272846)] [gbkey=CDS]	1818	93.90%	99.90%
[locus_tag=DQM41_RS06670]	lcl|NZ_LS483362.1_cds_WP_002864247.1_1293	316	663	348	forward	316	663	348	316	663	[protein=DUF2920 family protein] [protein_id=WP_002864247.1] [location=complement(1274676..1275917)] [gbkey=CDS]	1242	60.30%	34.80%
[locus_tag=DQM41_RS06680]	lcl|NZ_LS483362.1_cds_WP_002864246.1_1295	193	1008	816	forward	193	1008	816	193	1008	[gene=tsaD] [protein=tRNA (adenosine(37)-N6)-threonylcarbamoyltransferase complex transferase subunit TsaD] [protein_id=WP_002864246.1] [location=complement(1276433.	1008	85.70%	81.60%
[locus_tag=DQM41_RS06705]	lcl|NZ_LS483362.1_cds_WP_002864240.1_1300	1039	1308	270	forward	1039	1308	270	1039	1308	[protein=fibronectin-binding domain-containing protein] [protein_id=WP_002864240.1] [location=complement(1280974..1282281)] [gbkey=CDS]	1308	86.70%	27.00%
[locus_tag=DQM41_RS06760]	lcl|NZ_LS483362.1_cds_WP_002860421.1_1310	559	1557	999	reverse	559	1557	999	559	1557	[protein=RNA degradosome polyphosphate kinase] [protein_id=WP_002860421.1] [location=1291113..1293197] [gbkey=CDS]	2085	98.20%	99.90%
[locus_tag=DQM41_RS06770]	lcl|NZ_LS483362.1_cds_WP_002859649.1_1311	34	453	420	forward	34	453	420	34	453	[protein=hypothetical protein] [protein_id=WP_002859649.1] [location=complement(1293641..1294093)] [gbkey=CDS]	453	98.60%	42.00%
[locus_tag=DQM41_RS06910]	lcl|NZ_LS483362.1_cds_1339	136	450	315	reverse	136	450	315	136	450	[protein=C4-dicarboxylate ABC transporter] [pseudo=true] [location=1325650..1327089] [gbkey=CDS]	1440	95.20%	31.50%
[locus_tag=DQM41_RS06915]	lcl|NZ_LS483362.1_cds_1340	633	1163	531	reverse	633	1163	531	633	1163	[protein=PLP-dependent transferase] [pseudo=true] [location=1327102..1328264] [gbkey=CDS]	1163	94.90%	53.10%
[locus_tag=DQM41_RS06915]	lcl|NZ_LS483362.1_cds_1340	120	632	513	reverse	120	632	513	120	632	[protein=PLP-dependent transferase] [pseudo=true] [location=1327102..1328264] [gbkey=CDS]	1163	97.70%	51.30%
[locus_tag=DQM41_RS06980]	lcl|NZ_LS483362.1_cds_WP_010891933.1_1353	577	1371	795	forward	577	1371	795	577	1371	[gene=pgmL] [protein=phosphoglucosamine mutase PgmL] [protein_id=WP_010891933.1] [location=complement(1339742..1341130)] [gbkey=CDS]	1389	89.80%	79.50%
[locus_tag=DQM41_RS07465]	lcl|NZ_LS483362.1_cds_WP_002851362.1_1449	307	642	336	forward	307	642	336	307	642	[gene=ccaA] [protein=aspartate chemotaxis receptor CcaA] [protein_id=WP_002851362.1] [location=complement(1440866..1442968)] [gbkey=CDS]	2103	98.20%	33.60%
[locus_tag=DQM41_RS07465]	lcl|NZ_LS483362.1_cds_WP_002851362.1_1449	1645	2103	459	forward	1645	2103	459	1645	2103	[gene=ccaA] [protein=aspartate chemotaxis receptor CcaA] [protein_id=WP_002851362.1] [location=complement(1440866..1442968)] [gbkey=CDS]	2103	100.00%	45.90%
[locus_tag=DQM41_RS07470]	lcl|NZ_LS483362.1_cds_WP_002822570.1_1450	1	495	495	forward	1	495	495	1	495	[protein=LysR family transcriptional regulator] [protein_id=WP_002822570.1] [location=complement(1443078..1443815)] [gbkey=CDS]	738	100.00%	49.50%
[locus_tag=DQM41_RS07490]	lcl|NZ_LS483362.1_cds_WP_010891941.1_1454	847	1422	576	forward	847	1422	576	847	1422	[protein=formate dehydrogenase subunit alpha] [transl_except=(pos:541..543,aa:Sec)] [protein_id=WP_010891941.1] [location=complement(1446324..1449128)] [gbkey=CDS]	2805	97.40%	57.60%
[locus_tag=DQM41_RS07510]	lcl|NZ_LS483362.1_cds_WP_002858205.1_1458	1	684	684	reverse	1	684	684	1	684	[gene=cueO] [protein=multicopper oxidase CueO] [protein_id=WP_002858205.1] [location=1451583..1453124] [gbkey=CDS]	1542	95.20%	68.40%
[locus_tag=DQM41_RS07550]	lcl|NZ_LS483362.1_cds_1466	654	1172	519	reverse	654	1172	519	654	1172	[protein=TRAP transporter large permease subunit] [pseudo=true] [location=1460273..1461466] [gbkey=CDS]	1194	93.60%	51.90%
[locus_tag=DQM41_RS07595]	lcl|NZ_LS483362.1_cds_WP_002851288.1_1475	1498	1746	249	reverse	1498	1746	249	1498	1746	[gene=acs] [protein=acetate--CoA ligase] [protein_id=WP_002851288.1] [location=complement(1468392..1470365)] [gbkey=CDS]	1974	47.00%	24.90%
[locus_tag=DQM41_RS07815]	lcl|NZ_LS483362.1_cds_WP_002851210.1_1519	325	1323	999	forward	325	1323	999	325	1323	[protein=FAD-binding oxidoreductase] [protein_id=WP_002851210.1] [location=complement(1514627..1517398)] [gbkey=CDS]	2772	95.10%	99.90%
[locus_tag=DQM41_RS07860]	lcl|NZ_LS483362.1_cds_WP_002851551.1_1528	1	558	558	reverse	1	558	558	1	558	[gene=rpsD] [protein=30S ribosomal protein S4] [protein_id=WP_002851551.1] [location=1523241..1523867] [gbkey=CDS]	627	97.80%	55.80%
[locus_tag=DQM41_RS07955]	lcl|NZ_LS483362.1_cds_WP_002851503.1_1547	665	739	75	reverse	665	739	75	665	739	[gene=hugZ] [protein=HugZ family heme oxygenase] [protein_id=WP_002851503.1] [location=complement(1539841..1540596)] [gbkey=CDS]	756	48.00%	7.50%
[locus_tag=DQM41_RS08030]	lcl|NZ_LS483362.1_cds_WP_002851484.1_1558	1	597	597	forward	1	597	597	1	597	[protein=L-serine ammonia-lyase] [protein_id=WP_002851484.1] [location=complement(1551738..1553102)] [gbkey=CDS]	1365	89.20%	59.70%
[locus_tag=DQM41_RS08140]	lcl|NZ_LS483362.1_cds_WP_002851140.1_1580	412	1095	684	reverse	412	1095	684	412	1095	[protein=ABC transporter permease] [protein_id=WP_002851140.1] [location=1571073..1572182] [gbkey=CDS]	1110	89.20%	68.40%
[locus_tag=DQM41_RS08200]	lcl|NZ_LS483362.1_cds_WP_002857880.1_1592	1536	2090	555	reverse	1536	2090	555	1536	2090	[protein=FTR1 family iron permease] [protein_id=WP_002857880.1] [location=1580819..1582909] [gbkey=CDS]	2091	95.10%	55.50%
[locus_tag=DQM41_RS08350]	lcl|NZ_LS483362.1_cds_WP_002851268.1_1617	169	1167	999	reverse	169	1167	999	169	1167	[protein=type I DNA topoisomerase] [protein_id=WP_002851268.1] [location=complement(1607343..1609445)] [gbkey=CDS]	2103	93.90%	99.90%
[locus_tag=DQM41_RS08370]	lcl|NZ_LS483362.1_cds_WP_002779428.1_1621	1	444	444	reverse	1	444	444	1	444	[gene=rpsE] [protein=30S ribosomal protein S5] [protein_id=WP_002779428.1] [location=complement(1612601..1613044)] [gbkey=CDS]	444	100.00%	44.40%
[locus_tag=DQM41_RS08425]	lcl|NZ_LS483362.1_cds_WP_002851138.1_1632	121	672	552	reverse	121	672	552	121	672	[gene=rpsC] [protein=30S ribosomal protein S3] [protein_id=WP_002851138.1] [location=complement(1616640..1617341)] [gbkey=CDS]	702	98.40%	55.20%
[locus_tag=DQM41_RS08470]	lcl|NZ_LS483362.1_cds_WP_002864793.1_1641	457	1455	999	reverse	457	1455	999	457	1455	[protein=ribonuclease J] [protein_id=WP_002864793.1] [location=complement(1621679..1623673)] [gbkey=CDS]	1995	99.70%	99.90%
[locus_tag=DQM41_RS08570]	lcl|NZ_LS483362.1_cds_WP_002853122.1_1657	1	630	630	reverse	1	630	630	1	630	[protein=O-acetylhomoserine aminocarboxypropyltransferase/cysteine synthase] [protein_id=WP_002853122.1] [location=complement(1636390..1637661)] [gbkey=CDS]	1272	96.20%	63.00%

Table S3: List of the Smash++ 1,000 bp segments, which presumably originate from *C. jejuni* of the isolate *C. coli* meC0467 with the corresponding locus tags of NCTC 11168.

locus_tag	Sequence Name	Minimum	Maximum	Length	Direction	Min (with gaps)	Max (with gaps)	Length (with gaps)	Min (original sequence)	Max (original sequence)	Description	Sequence Length	% Pairwise Identity	Query Coverage
[locus_tag=DQM41_RS00035]	lcl|NZ_LS483362.1_cds_WP_002853155.1_7	2683	3678	996	forward	2683	3678	996	2683	3678	[gene=gltB] [protein=glutamate synthase large subunit] [protein_id=WP_002853155.1] [location=8144..12634] [gbkey=CDS]	4491	100.00%	99.90%
[locus_tag=DQM41_RS00035]	lcl|NZ_LS483362.1_cds_WP_002853155.1_7	1681	2679	999	forward	1681	2679	999	1681	2679	[gene=gltB] [protein=glutamate synthase large subunit] [protein_id=WP_002853155.1] [location=8144..12634] [gbkey=CDS]	4491	94.70%	99.60%
[locus_tag=DQM41_RS00060]	lcl|NZ_LS483362.1_cds_WP_002853081.1_12	1	525	525	reverse	1	525	525	1	525	[protein=desulfoferrodoxin FeS4 iron-binding domain-containing protein] [protein_id=WP_002853081.1] [location=complement(16756..17403)] [gbkey=CDS]	648	90.60%	52.50%
[locus_tag=DQM41_RS00075]	lcl|NZ_LS483362.1_cds_WP_002853125.1_15	1	627	627	reverse	1	627	627	1	627	[protein=HD family hydrolase] [protein_id=WP_002853125.1] [location=complement(19867..21093)] [gbkey=CDS]	1227	99.00%	62.70%
[locus_tag=DQM41_RS00085]	lcl|NZ_LS483362.1_cds_WP_002787430.1_17	1033	1527	495	reverse	1033	1527	495	1033	1527	[gene=dsbI] [protein=disulfide bond formation protein DsbI] [protein_id=WP_002787430.1] [location=complement(21854..23380)] [gbkey=CDS]	1527	94.50%	49.50%
[locus_tag=DQM41_RS00110]	lcl|NZ_LS483362.1_cds_WP_010891825.1_22	31	717	687	reverse	31	717	687	31	717	[protein=RluA family pseudouridine synthase] [protein_id=WP_010891825.1] [location=complement(27402..28304)] [gbkey=CDS]	903	86.00%	68.70%
[locus_tag=DQM41_RS00120]	lcl|NZ_LS483362.1_cds_WP_002865087.1_24	1552	2370	819	forward	1552	2370	819	1552	2370	[protein=ribonucleoside-diphosphate reductase subunit alpha] [protein_id=WP_002865087.1] [location=29726..32095] [gbkey=CDS]	2370	97.80%	54.90%
[locus_tag=DQM41_RS00120]	lcl|NZ_LS483362.1_cds_WP_002865087.1_24	1	549	549	forward	1	549	549	1	549	[protein=ribonucleoside-diphosphate reductase subunit alpha] [protein_id=WP_002865087.1] [location=29726..32095] [gbkey=CDS]	2370	94.90%	81.90%
[locus_tag=DQM41_RS00125]	lcl|NZ_LS483362.1_cds_WP_002865086.1_25	403	1236	834	reverse	403	1236	834	403	1236	[protein=L-cystine transporter] [protein_id=WP_002865086.1] [location=complement(32134..33519)] [gbkey=CDS]	1386	84.70%	83.40%
[locus_tag=DQM41_RS00145]	lcl|NZ_LS483362.1_cds_WP_072233179.1_29	625	858	234	forward	625	858	234	625	858	[protein=type II asparaginase] [protein_id=WP_072233179.1] [location=37625..38662] [gbkey=CDS]	1038	82.10%	23.40%
[locus_tag=DQM41_RS00180]	lcl|NZ_LS483362.1_cds_31	746	1744	999	forward	746	1744	999	746	1744	[protein=class I SAM-dependent DNA methyltransferase] [pseudo=true] [location=46420..50152] [gbkey=CDS]	3733	92.00%	99.90%
[locus_tag=DQM41_RS00190]	lcl|NZ_LS483362.1_cds_WP_002866314.1_33	64	702	639	reverse	64	702	639	64	702	[protein=SIMPL domain-containing protein] [protein_id=WP_002866314.1] [location=complement(51963..52664)] [gbkey=CDS]	702	90.60%	63.90%
[locus_tag=DQM41_RS00200]	lcl|NZ_LS483362.1_cds_WP_010891829.1_35	805	1350	546	forward	805	1350	546	805	1350	[protein=DUF2130 domain-containing protein] [protein_id=WP_010891829.1] [location=53966..55315] [gbkey=CDS]	1350	97.30%	54.60%
[locus_tag=DQM41_RS00230]	lcl|NZ_LS483362.1_cds_WP_002805290.1_41	112	591	480	forward	112	591	480	112	591	[protein=flagellar basal body rod modification protein] [protein_id=WP_002805290.1] [location=61339..62223] [gbkey=CDS]	885	90.30%	48.00%
[locus_tag=DQM41_RS00235]	lcl|NZ_LS483362.1_cds_WP_002805291.1_42	727	1638	912	forward	727	1638	912	727	1638	[protein=flagellar hook protein FlgE] [protein_id=WP_002805291.1] [location=62227..63864] [gbkey=CDS]	1638	99.30%	91.20%
[locus_tag=DQM41_RS00240]	lcl|NZ_LS483362.1_cds_WP_010891832.1_43	1	786	786	reverse	1	786	786	1	786	[protein=DUF342 domain-containing protein] [protein_id=WP_010891832.1] [location=complement(63868..65739)] [gbkey=CDS]	1872	98.90%	78.60%
[locus_tag=DQM41_RS00285]	lcl|NZ_LS483362.1_cds_WP_002851977.1_51	805	1017	213	reverse	805	1017	213	805	1017	[gene=mnmA] [protein=tRNA 2-thiouridine(34) synthase MnmA] [protein_id=WP_002851977.1] [location=complement(68528..69544)] [gbkey=CDS]	1017	88.70%	21.30%
[locus_tag=DQM41_RS00285]	lcl|NZ_LS483362.1_cds_WP_002851977.1_51	1	801	801	reverse	1	801	801	1	801	[gene=mnmA] [protein=tRNA 2-thiouridine(34) synthase MnmA] [protein_id=WP_002851977.1] [location=complement(68528..69544)] [gbkey=CDS]	1017	98.90%	80.10%
[locus_tag=DQM41_RS00310]	lcl|NZ_LS483362.1_cds_WP_002851963.1_56	208	570	363	forward	208	570	363	208	570	[protein=peptidase C39] [protein_id=WP_002851963.1] [location=72840..73439] [gbkey=CDS]	600	96.70%	36.30%
[locus_tag=DQM41_RS00325]	lcl|NZ_LS483362.1_cds_WP_002859753.1_59	4	366	363	reverse	4	366	363	4	366	[protein=RNA polymerase sigma factor FliA] [protein_id=WP_002859753.1] [location=complement(75406..76122)] [gbkey=CDS]	717	83.00%	36.30%
[locus_tag=DQM41_RS00610]	lcl|NZ_LS483362.1_cds_WP_002851870.1_116	106	474	369	reverse	106	474	369	106	474	[protein=hypothetical protein] [protein_id=WP_002851870.1] [location=124785..125474] [gbkey=CDS]	690	93.50%	36.90%
[locus_tag=DQM41_RS00615]	lcl|NZ_LS483362.1_cds_WP_002851926.1_117	397	903	507	forward	397	903	507	397	903	[protein=tRNA-dihydrouridine synthase] [protein_id=WP_002851926.1] [location=complement(125499..126425)] [gbkey=CDS]	927	95.30%	50.70%
[locus_tag=DQM41_RS00670]	lcl|NZ_LS483362.1_cds_WP_002888538.1_128	307	858	552	reverse	307	858	552	307	858	[protein=homoserine kinase] [protein_id=WP_002888538.1] [location=135705..136583] [gbkey=CDS]	879	72.30%	55.20%
[locus_tag=DQM41_RS00825]	lcl|NZ_LS483362.1_cds_WP_002858709.1_159	325	1158	834	forward	325	1158	834	325	1158	[gene=nifS] [protein=cysteine desulfurase, NifS family] [protein_id=WP_002858709.1] [location=163434..164615] [gbkey=CDS]	1182	97.80%	83.40%
[locus_tag=DQM41_RS00830]	lcl|NZ_LS483362.1_cds_WP_002840050.1_160	133	972	840	forward	133	972	840	133	972	[protein=iron-sulfur cluster assembly scaffold protein NifU] [protein_id=WP_002840050.1] [location=164625..165596] [gbkey=CDS]	972	95.60%	84.00%
[locus_tag=DQM41_RS01100]	lcl|NZ_LS483362.1_cds_WP_002851704.1_214	1	837	837	reverse	1	837	837	1	837	[protein=ChaN family lipoprotein] [protein_id=WP_002851704.1] [location=complement(213743..214594)] [gbkey=CDS]	852	92.50%	83.70%
[locus_tag=DQM41_RS01160]	lcl|NZ_LS483362.1_cds_WP_002851982.1_225	1	570	570	reverse	1	570	570	1	570	[gene=miaA] [protein=tRNA (adenosine(37)-N6)-dimethylallyltransferase MiaA] [protein_id=WP_002851982.1] [location=complement(222479..223348)] [gbkey=CDS]	870	83.70%	57.00%
[locus_tag=DQM41_RS01275]	lcl|NZ_LS483362.1_cds_WP_010891848.1_248	2038	2517	480	forward	2038	2517	480	2038	2517	[protein=molybdopterin guanine dinucleotide-containing S/N-oxide reductase] [protein_id=WP_010891848.1] [location=complement(241915..244431)] [gbkey=CDS]	2517	91.10%	99.60%
[locus_tag=DQM41_RS01275]	lcl|NZ_LS483362.1_cds_WP_010891848.1_248	1039	2034	996	forward	1039	2034	996	1039	2034	[protein=molybdopterin guanine dinucleotide-containing S/N-oxide reductase] [protein_id=WP_010891848.1] [location=complement(241915..244431)] [gbkey=CDS]	2517	100.00%	48.00%
[locus_tag=DQM41_RS01410]	lcl|NZ_LS483362.1_cds_WP_002854555.1_274	1	474	474	reverse	1	474	474	1	474	[protein=5'/3'-nucleotidase SurE] [protein_id=WP_002854555.1] [location=269058..269834] [gbkey=CDS]	777	87.10%	47.40%
[locus_tag=DQM41_RS01430]	lcl|NZ_LS483362.1_cds_WP_002778123.1_278	3	728	726	forward	3	728	726	3	728	[protein=pantoate--beta-alanine ligase] [protein_id=WP_002778123.1] [location=complement(271412..272260)] [gbkey=CDS]	849	99.20%	72.60%
[locus_tag=DQM41_RS01485]	lcl|NZ_LS483362.1_cds_WP_002816248.1_289	1	606	606	forward	1	606	606	1	606	[protein=dethiobiotin synthase] [protein_id=WP_002816248.1] [location=complement(280690..281295)] [gbkey=CDS]	606	99.00%	60.60%
[locus_tag=DQM41_RS01500]	lcl|NZ_LS483362.1_cds_WP_002791314.1_292	1	498	498	reverse	1	498	498	1	498	[protein=50S ribosomal protein L25/general stress protein Ctc] [protein_id=WP_002791314.1] [location=282095..282631] [gbkey=CDS]	537	100.00%	49.80%
[locus_tag=DQM41_RS01520]	lcl|NZ_LS483362.1_cds_WP_002858702.1_296	115	768	654	reverse	115	768	654	115	768	[protein=HAD-IIA family hydrolase] [protein_id=WP_002858702.1] [location=285487..286254] [gbkey=CDS]	768	93.80%	65.40%
[locus_tag=DQM41_RS01635]	lcl|NZ_LS483362.1_cds_WP_002858644.1_319	1912	2640	729	forward	1912	2640	729	1912	2640	[gene=polA] [protein=DNA polymerase I] [protein_id=WP_002858644.1] [location=complement(305724..308363)] [gbkey=CDS]	2640	89.00%	72.90%
[locus_tag=DQM41_RS01740]	lcl|NZ_LS483362.1_cds_WP_002860288.1_340	967	1338	372	reverse	967	1338	372	967	1338	[protein=phosphoglucosamine mutase] [protein_id=WP_002860288.1] [location=327136..328473] [gbkey=CDS]	1338	92.70%	37.20%
[locus_tag=DQM41_RS01770]	lcl|NZ_LS483362.1_cds_WP_002872052.1_346	321	614	294	forward	321	614	294	321	614	[gene=cmeB] [protein=multidrug efflux RND transporter permease subunit CmeB] [protein_id=WP_002872052.1] [location=complement(332589..335711)] [gbkey=CDS]	3123	91.80%	29.40%
[locus_tag=DQM41_RS01805]	lcl|NZ_LS483362.1_cds_WP_002858689.1_353	4	999	996	reverse	4	999	996	4	999	[protein=glutathionylspermidine synthase family protein] [protein_id=WP_002858689.1] [location=340011..341180] [gbkey=CDS]	1170	88.70%	99.60%
[locus_tag=DQM41_RS01885]	lcl|NZ_LS483362.1_cds_WP_002858719.1_369	1	690	690	reverse	1	690	690	1	690	[gene=trpS] [protein=tryptophan--tRNA ligase] [protein_id=WP_002858719.1] [location=353302..354261] [gbkey=CDS]	960	93.90%	69.00%
[locus_tag=DQM41_RS02045]	lcl|NZ_LS483362.1_cds_WP_002855621.1_401	64	573	510	reverse	64	573	510	64	573	[protein=polyisoprenoid-binding protein] [protein_id=WP_002855621.1] [location=385188..385760] [gbkey=CDS]	573	87.10%	51.00%
[locus_tag=DQM41_RS02380]	lcl|NZ_LS483362.1_cds_WP_010891860.1_458	3616	4137	522	reverse	3616	4137	522	3616	4137	[gene=rpoB] [protein=DNA-directed RNA polymerase subunit beta] [protein_id=WP_010891860.1] [location=438986..443122] [gbkey=CDS]	4137	97.70%	52.20%
[locus_tag=DQM41_RS02380]	lcl|NZ_LS483362.1_cds_WP_010891860.1_458	1615	2613	999	reverse	1615	2613	999	1615	2613	[gene=rpoB] [protein=DNA-directed RNA polymerase subunit beta] [protein_id=WP_010891860.1] [location=438986..443122] [gbkey=CDS]	4137	99.40%	99.90%
[locus_tag=DQM41_RS02405]	lcl|NZ_LS483362.1_cds_WP_025096279.1_463	1	744	744	reverse	1	744	744	1	744	[protein=UxaA family hydrolase] [protein_id=WP_025096279.1] [location=449862..451028] [gbkey=CDS]	1167	96.80%	74.40%
[locus_tag=DQM41_RS02410]	lcl|NZ_LS483362.1_cds_WP_002864169.1_464	570	1229	660	reverse	570	1229	660	570	1229	[protein=MFS transporter] [protein_id=WP_002864169.1] [location=451040..452272] [gbkey=CDS]	1233	80.70%	66.00%
[locus_tag=DQM41_RS02415]	lcl|NZ_LS483362.1_cds_WP_002825115.1_465	286	783	498	reverse	286	783	498	286	783	[protein=SDR family oxidoreductase] [protein_id=WP_002825115.1] [location=452314..453102] [gbkey=CDS]	789	96.40%	49.80%
[locus_tag=DQM41_RS02420]	lcl|NZ_LS483362.1_cds_WP_002864166.1_466	619	1236	618	reverse	619	1236	618	619	1236	[gene=fucP] [protein=L-fucose:H+ symporter permease] [protein_id=WP_002864166.1] [location=453113..454369] [gbkey=CDS]	1257	92.20%	61.80%
[locus_tag=DQM41_RS02435]	lcl|NZ_LS483362.1_cds_469	189	1136	948	reverse	189	1136	948	189	1136	[gene=aldA] [protein=aldehyde dehydrogenase] [pseudo=true] [location=455455..456893] [gbkey=CDS]	1439	96.80%	94.80%
[locus_tag=DQM41_RS02440]	lcl|NZ_LS483362.1_cds_WP_002782934.1_470	76	387	312	reverse	76	387	312	76	387	[protein=30S ribosomal protein S12] [protein_id=WP_002782934.1] [location=457221..457607] [gbkey=CDS]	387	99.00%	31.20%
[locus_tag=DQM41_RS02635]	lcl|NZ_LS483362.1_cds_WP_002858520.1_508	364	1362	999	reverse	364	1362	999	364	1362	[protein=NADP-dependent isocitrate dehydrogenase] [protein_id=WP_002858520.1] [location=494071..496275] [gbkey=CDS]	2205	87.90%	99.90%
[locus_tag=DQM41_RS02670]	lcl|NZ_LS483362.1_cds_WP_002864641.1_515	1	558	558	reverse	1	558	558	1	558	[gene=oorC] [protein=2-oxoacid:acceptor oxidoreductase family protein] [protein_id=WP_002864641.1] [location=501519..502076] [gbkey=CDS]	558	91.10%	55.80%
[locus_tag=DQM41_RS02695]	lcl|NZ_LS483362.1_cds_WP_010891863.1_520	241	1239	999	reverse	241	1239	999	241	1239	[protein=proline--tRNA ligase] [protein_id=WP_010891863.1] [location=505206..506915] [gbkey=CDS]	1710	95.20%	99.90%
[locus_tag=DQM41_RS02710]	lcl|NZ_LS483362.1_cds_WP_002865770.1_523	1230	1802	573	reverse	1230	1802	573	1230	1802	[protein=menaquinone biosynthesis decarboxylase] [protein_id=WP_002865770.1] [location=508218..510020] [gbkey=CDS]	1803	99.50%	57.30%
[locus_tag=DQM41_RS02710]	lcl|NZ_LS483362.1_cds_WP_002865770.1_523	229	1227	999	reverse	229	1227	999	229	1227	[protein=menaquinone biosynthesis decarboxylase] [protein_id=WP_002865770.1] [location=508218..510020] [gbkey=CDS]	1803	99.70%	99.90%
[locus_tag=DQM41_RS02720]	lcl|NZ_LS483362.1_cds_WP_002864504.1_525	43	900	858	reverse	43	900	858	43	900	[gene=fliD] [protein=flagellar filament capping protein FliD] [protein_id=WP_002864504.1] [location=510546..512474] [gbkey=CDS]	1929	99.00%	85.80%
[locus_tag=DQM41_RS02890]	lcl|NZ_LS483362.1_cds_WP_002852171.1_556	85	714	630	reverse	85	714	630	85	714	[gene=tatC] [protein=twin-arginine translocase subunit TatC] [protein_id=WP_002852171.1] [location=complement(538990..539727)] [gbkey=CDS]	738	95.70%	63.00%
[locus_tag=DQM41_RS03070]	lcl|NZ_LS483362.1_cds_WP_002776292.1_592	3	575	573	reverse	3	575	573	3	575	[gene=pstC] [protein=phosphate ABC transporter permease subunit PstC] [protein_id=WP_002776292.1] [location=574220..575134] [gbkey=CDS]	915	99.00%	57.30%
[locus_tag=DQM41_RS03080]	lcl|NZ_LS483362.1_cds_WP_010891867.1_594	1	579	579	reverse	1	579	579	1	579	[protein=phosphate ABC transporter ATP-binding protein] [protein_id=WP_010891867.1] [location=576216..576956] [gbkey=CDS]	741	99.50%	57.90%
[locus_tag=DQM41_RS03090]	lcl|NZ_LS483362.1_cds_WP_002906848.1_596	323	1315	993	reverse	323	1315	993	323	1315	[protein=MATE family efflux transporter] [protein_id=WP_002906848.1] [location=578335..579651] [gbkey=CDS]	1317	91.10%	99.30%
[locus_tag=DQM41_RS03095]	lcl|NZ_LS483362.1_cds_WP_002858500.1_597	31	648	618	reverse	31	648	618	31	648	[protein=M48 family metallopeptidase] [protein_id=WP_002858500.1] [location=579627..580274] [gbkey=CDS]	648	95.60%	61.80%
[locus_tag=DQM41_RS03100]	lcl|NZ_LS483362.1_cds_WP_002826526.1_598	343	1338	996	reverse	343	1338	996	343	1338	[protein=hypothetical protein] [protein_id=WP_002826526.1] [location=580316..581836] [gbkey=CDS]	1521	91.60%	99.60%
[locus_tag=DQM41_RS03120]	lcl|NZ_LS483362.1_cds_WP_002776343.1_602	1	564	564	reverse	1	564	564	1	564	[gene=hypD] [protein=hydrogenase formation protein HypD] [protein_id=WP_002776343.1] [location=585092..586183] [gbkey=CDS]	1092	99.50%	56.40%
[locus_tag=DQM41_RS03125]	lcl|NZ_LS483362.1_cds_WP_002776344.1_603	480	974	495	reverse	480	974	495	480	974	[gene=hypE] [protein=hydrogenase expression/formation protein HypE] [protein_id=WP_002776344.1] [location=586180..587154] [gbkey=CDS]	975	99.40%	49.50%
[locus_tag=DQM41_RS03300]	lcl|NZ_LS483362.1_cds_WP_002852226.1_638	271	1098	828	forward	271	1098	828	271	1098	[gene=hslU] [protein=ATP-dependent protease ATPase subunit HslU] [protein_id=WP_002852226.1] [location=complement(619068..620387)] [gbkey=CDS]	1320	86.80%	82.80%
[locus_tag=DQM41_RS03340]	lcl|NZ_LS483362.1_cds_WP_002865516.1_646	156	566	411	reverse	156	566	411	156	566	[protein=anaerobic C4-dicarboxylate transporter] [protein_id=WP_002865516.1] [location=625542..626966] [gbkey=CDS]	1425	87.00%	41.10%
[locus_tag=DQM41_RS03630]	lcl|NZ_LS483362.1_cds_WP_002852367.1_704	1	486	486	forward	1	486	486	1	486	[gene=corA] [protein=magnesium/cobalt transporter CorA] [protein_id=WP_002852367.1] [location=complement(680358..681341)] [gbkey=CDS]	984	99.40%	48.60%
[locus_tag=DQM41_RS03640]	lcl|NZ_LS483362.1_cds_WP_002793291.1_706	343	1326	984	reverse	343	1326	984	343	1326	[protein=esterase-like activity of phytase family protein] [protein_id=WP_002793291.1] [location=682515..683840] [gbkey=CDS]	1326	99.40%	98.40%
[locus_tag=DQM41_RS03645]	lcl|NZ_LS483362.1_cds_WP_010891882.1_707	22	795	774	reverse	22	795	774	22	795	[protein=alkaline phosphatase family protein] [protein_id=WP_010891882.1] [location=683837..684646] [gbkey=CDS]	810	100.00%	77.40%
[locus_tag=DQM41_RS03650]	lcl|NZ_LS483362.1_cds_WP_002784190.1_708	528	839	312	reverse	528	839	312	528	839	[protein=ABC transporter permease] [protein_id=WP_002784190.1] [location=684643..685482] [gbkey=CDS]	840	98.10%	31.20%
[locus_tag=DQM41_RS03665]	lcl|NZ_LS483362.1_cds_WP_002810914.1_711	1	603	603	reverse	1	603	603	1	603	[protein=HAD-IB family hydrolase] [protein_id=WP_002810914.1] [location=687251..687889] [gbkey=CDS]	639	99.00%	60.30%
[locus_tag=DQM41_RS03735]	lcl|NZ_LS483362.1_cds_WP_002792117.1_720	1	213	213	reverse	1	213	213	1	213	[protein=hypothetical protein] [protein_id=WP_002792117.1] [location=703533..703796] [gbkey=CDS]	264	97.20%	21.30%
[locus_tag=DQM41_RS03740]	lcl|NZ_LS483362.1_cds_WP_072280311.1_721	1	438	438	reverse	1	438	438	1	438	[protein=IS200/IS605 family element transposase accessory protein TnpB] [protein_id=WP_072280311.1] [location=703917..704354] [gbkey=CDS]	438	99.30%	43.80%
[locus_tag=DQM41_RS03745]	lcl|NZ_LS483362.1_cds_WP_002859789.1_722	3	248	246	forward	3	248	246	3	248	[protein=energy transducer TonB] [protein_id=WP_002859789.1] [location=complement(704427..705110)] [gbkey=CDS]	684	86.00%	24.60%
[locus_tag=DQM41_RS03750]	lcl|NZ_LS483362.1_cds_WP_010891883.1_723	268	1266	999	reverse	268	1266	999	268	1266	[gene=cfrA] [protein=TonB-dependent ferric enterobactin receptor CfrA] [protein_id=WP_010891883.1] [location=705435..707525] [gbkey=CDS]	2091	100.00%	99.90%
[locus_tag=DQM41_RS03765]	lcl|NZ_LS483362.1_cds_WP_002826316.1_726	535	1530	996	reverse	535	1530	996	535	1530	[gene=dnaK] [protein=molecular chaperone DnaK] [protein_id=WP_002826316.1] [location=709168..711039] [gbkey=CDS]	1872	94.20%	99.60%
[locus_tag=DQM41_RS04280]	lcl|NZ_LS483362.1_cds_WP_002864843.1_829	1441	1509	69	forward	1441	1509	69	1441	1509	[protein=chorismate-binding protein] [protein_id=WP_002864843.1] [location=complement(807480..809264)] [gbkey=CDS]	1785	47.80%	6.90%
[locus_tag=DQM41_RS04360]	lcl|NZ_LS483362.1_cds_WP_010891893.1_845	619	1617	999	reverse	619	1617	999	619	1617	[protein=DNA translocase FtsK] [protein_id=WP_010891893.1] [location=complement(820666..823506)] [gbkey=CDS]	2841	96.30%	99.90%
[locus_tag=DQM41_RS04360]	lcl|NZ_LS483362.1_cds_WP_010891893.1_845	205	618	414	reverse	205	618	414	205	618	[protein=DNA translocase FtsK] [protein_id=WP_010891893.1] [location=complement(820666..823506)] [gbkey=CDS]	2841	83.80%	41.40%
[locus_tag=DQM41_RS04585]	lcl|NZ_LS483362.1_cds_WP_002858396.1_888	1	627	627	reverse	1	627	627	1	627	[gene=ychF] [protein=redox-regulated ATPase YchF] [protein_id=WP_002858396.1] [location=863643..864746] [gbkey=CDS]	1104	93.80%	62.70%
[locus_tag=DQM41_RS04615]	lcl|NZ_LS483362.1_cds_WP_002853292.1_894	76	330	255	reverse	76	330	255	76	330	[protein=F0F1 ATP synthase subunit C] [protein_id=WP_002853292.1] [location=872498..872836] [gbkey=CDS]	339	54.90%	25.50%
[locus_tag=DQM41_RS04630]	lcl|NZ_LS483362.1_cds_WP_002857980.1_896	2383	3381	999	forward	2383	3381	999	2383	3381	[protein=acyl-[ACP]--phospholipid O-acyltransferase] [protein_id=WP_002857980.1] [location=complement(874072..877584)] [gbkey=CDS]	3513	90.40%	99.90%
[locus_tag=DQM41_RS04875]	lcl|NZ_LS483362.1_cds_WP_010891898.1_938	43	732	690	reverse	43	732	690	43	732	[protein=lipoprotein] [protein_id=WP_010891898.1] [location=916608..917726] [gbkey=CDS]	1119	47.00%	69.00%
[locus_tag=DQM41_RS04935]	lcl|NZ_LS483362.1_cds_WP_002852925.1_950	85	984	900	forward	85	984	900	85	984	[gene=hemB] [protein=porphobilinogen synthase] [protein_id=WP_002852925.1] [location=complement(926151..927134)] [gbkey=CDS]	984	92.30%	90.00%
[locus_tag=DQM41_RS04990]	lcl|NZ_LS483362.1_cds_WP_002858158.1_961	1	474	474	forward	1	474	474	1	474	[gene=mtaB] [protein=tRNA (N(6)-L-threonylcarbamoyladenosine(37)-C(2))-methylthiotransferase MtaB] [protein_id=WP_002858158.1] [location=complement(936228..937478)] [gbkey=CDS]	1251	95.60%	47.40%
[locus_tag=DQM41_RS05110]	lcl|NZ_LS483362.1_cds_WP_002852941.1_984	46	645	600	forward	46	645	600	46	645	[protein=hypothetical protein] [protein_id=WP_002852941.1] [location=complement(960817..961461)] [gbkey=CDS]	645	78.50%	60.00%
[locus_tag=DQM41_RS05115]	lcl|NZ_LS483362.1_cds_WP_002837927.1_985	1	627	627	forward	1	627	627	1	627	[gene=lepA] [protein=elongation factor 4] [protein_id=WP_002837927.1] [location=complement(961538..963334)] [gbkey=CDS]	1797	94.50%	62.70%
[locus_tag=DQM41_RS05120]	lcl|NZ_LS483362.1_cds_WP_002852947.1_986	367	1248	882	reverse	367	1248	882	367	1248	[gene=cmeD] [protein=multidrug efflux RND transporter outer membrane subunit CmeD] [protein_id=WP_002852947.1] [location=963461..964735] [gbkey=CDS]	1275	90.50%	88.20%
[locus_tag=DQM41_RS05125]	lcl|NZ_LS483362.1_cds_WP_002858463.1_987	1	741	741	reverse	1	741	741	1	741	[gene=cmeE] [protein=multidrug efflux RND transporter periplasmic adaptor subunit CmeE] [protein_id=WP_002858463.1] [location=964732..965472] [gbkey=CDS]	741	98.40%	74.10%
[locus_tag=DQM41_RS05130]	lcl|NZ_LS483362.1_cds_WP_010891901.1_988	1237	2232	996	reverse	1237	2232	996	1237	2232	[gene=cmeF] [protein=multidrug efflux RND transporter permease subunit CmeF] [protein_id=WP_010891901.1] [location=965475..968492] [gbkey=CDS]	3018	99.40%	99.60%
[locus_tag=DQM41_RS05130]	lcl|NZ_LS483362.1_cds_WP_010891901.1_988	235	1173	939	reverse	235	1173	939	235	1173	[gene=cmeF] [protein=multidrug efflux RND transporter permease subunit CmeF] [protein_id=WP_010891901.1] [location=965475..968492] [gbkey=CDS]	3018	96.20%	93.90%
[locus_tag=DQM41_RS05220]	lcl|NZ_LS483362.1_cds_WP_002864285.1_1006	2359	3354	996	forward	2359	3354	996	2359	3354	[protein=N-6 DNA methylase] [protein_id=WP_002864285.1] [location=complement(982973..986992)] [gbkey=CDS]	4020	100.00%	99.90%
[locus_tag=DQM41_RS05220]	lcl|NZ_LS483362.1_cds_WP_002864285.1_1006	1357	2355	999	forward	1357	2355	999	1357	2355	[protein=N-6 DNA methylase] [protein_id=WP_002864285.1] [location=complement(982973..986992)] [gbkey=CDS]	4020	98.80%	99.90%
[locus_tag=DQM41_RS05220]	lcl|NZ_LS483362.1_cds_WP_002864285.1_1006	358	1356	999	forward	358	1356	999	358	1356	[protein=N-6 DNA methylase] [protein_id=WP_002864285.1] [location=complement(982973..986992)] [gbkey=CDS]	4020	98.50%	99.60%
[locus_tag=DQM41_RS05225]	lcl|NZ_LS483362.1_cds_WP_010891903.1_1007	577	1500	924	forward	577	1500	924	577	1500	[protein=endonuclease MutS2] [protein_id=WP_010891903.1] [location=complement(987001..989211)] [gbkey=CDS]	2211	99.40%	92.40%
[locus_tag=DQM41_RS05395]	lcl|NZ_LS483362.1_cds_WP_002852904.1_1041	121	180	60	reverse	121	180	60	121	180	[protein=M23 family metallopeptidase] [protein_id=WP_002852904.1] [location=complement(1018879..1019781)] [gbkey=CDS]	903	50.00%	6.00%
[locus_tag=DQM41_RS05690]	lcl|NZ_LS483362.1_cds_WP_002875425.1_1100	1	594	594	reverse	1	594	594	1	594	[gene=waaF] [protein=lipopolysaccharide heptosyltransferase II] [protein_id=WP_002875425.1] [location=1080891..1081850] [gbkey=CDS]	960	80.60%	59.40%
[locus_tag=DQM41_RS05800]	lcl|NZ_LS483362.1_cds_WP_010891915.1_1122	1093	1422	330	forward	1093	1422	330	1093	1422	[gene=omp50] [protein=outer membrane tyrosine kinase] [protein_id=WP_010891915.1] [location=complement(1098598..1100019)] [gbkey=CDS]	1422	100.00%	33.00%
[locus_tag=DQM41_RS05820]	lcl|NZ_LS483362.1_cds_WP_002780599.1_1126	3	308	306	reverse	3	308	306	3	308	[protein=multidrug efflux SMR transporter] [protein_id=WP_002780599.1] [location=1101824..1102132] [gbkey=CDS]	309	89.70%	30.60%
[locus_tag=DQM41_RS05910]	lcl|NZ_LS483362.1_cds_WP_002852850.1_1144	370	1365	996	reverse	370	1365	996	370	1365	[protein=cation:dicarboxylase symporter family transporter] [protein_id=WP_002852850.1] [location=1119951..1121327] [gbkey=CDS]	1377	95.70%	99.60%
[locus_tag=DQM41_RS06015]	lcl|NZ_LS483362.1_cds_WP_002852810.1_1165	979	1140	162	forward	979	1140	162	979	1140	[protein=FAD-binding protein] [protein_id=WP_002852810.1] [location=complement(1141617..1142999)] [gbkey=CDS]	1383	31.50%	16.20%
[locus_tag=DQM41_RS06220]	lcl|NZ_LS483362.1_cds_WP_002853182.1_1205	1093	2049	957	reverse	1093	2049	957	1093	2049	[protein=polyribonucleotide nucleotidyltransferase] [protein_id=WP_002853182.1] [location=1183577..1185736] [gbkey=CDS]	2160	89.00%	95.70%
[locus_tag=DQM41_RS06260]	lcl|NZ_LS483362.1_cds_WP_002853168.1_1213	1	525	525	forward	1	525	525	1	525	[protein=response regulator transcription factor] [protein_id=WP_002853168.1] [location=1191771..1192442] [gbkey=CDS]	672	94.30%	52.50%
[locus_tag=DQM41_RS06285]	lcl|NZ_LS483362.1_cds_WP_002853190.1_1218	1	909	909	reverse	1	909	909	1	909	[protein=nickel-dependent hydrogenase large subunit] [protein_id=WP_002853190.1] [location=complement(1195479..1197194)] [gbkey=CDS]	1716	93.90%	90.90%
[locus_tag=DQM41_RS06445]	lcl|NZ_LS483362.1_cds_WP_002856586.1_1248	90	791	702	reverse	90	791	702	90	791	[gene=legH] [protein=N-acetyltransferase LegH] [protein_id=WP_002856586.1] [location=1229079..1229870] [gbkey=CDS]	792	93.60%	70.20%
[locus_tag=DQM41_RS06465]	lcl|NZ_LS483362.1_cds_WP_002786080.1_1252	742	1563	822	reverse	742	1563	822	742	1563	[protein=HAD-IIIC family phosphatase] [protein_id=WP_002786080.1] [location=1231427..1232989] [gbkey=CDS]	1563	99.60%	82.20%
[locus_tag=DQM41_RS06465]	lcl|NZ_LS483362.1_cds_WP_002786080.1_1252	1	525	525	reverse	1	525	525	1	525	[protein=HAD-IIIC family phosphatase] [protein_id=WP_002786080.1] [location=1231427..1232989] [gbkey=CDS]	1563	99.40%	52.50%
[locus_tag=DQM41_RS06490]	lcl|NZ_LS483362.1_cds_WP_002858432.1_1257	733	1221	489	forward	733	1221	489	733	1221	[protein=DUF2920 family protein] [protein_id=WP_002858432.1] [location=complement(1235506..1236732)] [gbkey=CDS]	1227	87.70%	48.90%
[locus_tag=DQM41_RS06505]	lcl|NZ_LS483362.1_cds_WP_002856137.1_1260	280	849	570	forward	280	849	570	280	849	[protein=hypothetical protein] [protein_id=WP_002856137.1] [location=complement(1238565..1239635)] [gbkey=CDS]	1071	98.90%	57.00%
[locus_tag=DQM41_RS06525]	lcl|NZ_LS483362.1_cds_WP_002830499.1_1264	1	732	732	reverse	1	732	732	1	732	[gene=pseG] [protein=UDP-2,4-diacetamido-2,4,6-trideoxy-beta-L-altropyranose hydrolase] [protein_id=WP_002830499.1] [location=1241631..1242455] [gbkey=CDS]	825	95.70%	73.20%
[locus_tag=DQM41_RS06550]	lcl|NZ_LS483362.1_cds_WP_002870258.1_1269	1	840	840	reverse	1	840	840	1	840	[gene=pseI] [protein=pseudaminic acid synthase] [protein_id=WP_002870258.1] [location=1245633..1246664] [gbkey=CDS]	1032	94.10%	84.00%
[locus_tag=DQM41_RS06565]	lcl|NZ_LS483362.1_cds_WP_002786732.1_1272	1	810	810	reverse	1	810	810	1	810	[gene=legB] [protein=4,6-dehydratase LegB] [protein_id=WP_002786732.1] [location=1248606..1249577] [gbkey=CDS]	972	99.60%	81.00%
[locus_tag=DQM41_RS06670]	lcl|NZ_LS483362.1_cds_WP_002864247.1_1293	563	1108	546	forward	563	1108	546	563	1108	[protein=DUF2920 family protein] [protein_id=WP_002864247.1] [location=complement(1274676..1275917)] [gbkey=CDS]	1242	89.60%	54.60%
[locus_tag=DQM41_RS06770]	lcl|NZ_LS483362.1_cds_WP_002859649.1_1311	1	453	453	forward	1	453	453	1	453	[protein=hypothetical protein] [protein_id=WP_002859649.1] [location=complement(1293641..1294093)] [gbkey=CDS]	453	100.00%	45.30%
[locus_tag=DQM41_RS06935]	lcl|NZ_LS483362.1_cds_WP_002865443.1_1344	658	1161	504	reverse	658	1161	504	658	1161	[gene=feoB] [protein=ferrous iron transport protein B] [protein_id=WP_002865443.1] [location=1331293..1333134] [gbkey=CDS]	1842	90.80%	50.40%
[locus_tag=DQM41_RS06935]	lcl|NZ_LS483362.1_cds_WP_002865443.1_1344	1	654	654	reverse	1	654	654	1	654	[gene=feoB] [protein=ferrous iron transport protein B] [protein_id=WP_002865443.1] [location=1331293..1333134] [gbkey=CDS]	1842	93.80%	65.40%
[locus_tag=DQM41_RS07215]	lcl|NZ_LS483362.1_cds_WP_002851475.1_1400	1	579	579	reverse	1	579	579	1	579	[gene=prfB] [protein=peptide chain release factor 2] [protein_id=WP_002851475.1] [location=1392577..1393674] [gbkey=CDS]	1098	99.00%	57.90%
[locus_tag=DQM41_RS07270]	lcl|NZ_LS483362.1_cds_WP_002851396.1_1411	3	572	570	reverse	3	572	570	3	572	[gene=flgK] [protein=flagellar hook-associated protein FlgK] [protein_id=WP_002851396.1] [location=1400584..1402410] [gbkey=CDS]	1827	93.70%	57.00%
[locus_tag=DQM41_RS07425]	lcl|NZ_LS483362.1_cds_WP_002851458.1_1442	148	1146	999	forward	148	1146	999	148	1146	[protein=adenylosuccinate synthase] [protein_id=WP_002851458.1] [location=complement(1431075..1432325)] [gbkey=CDS]	1251	99.70%	99.90%
[locus_tag=DQM41_RS07460]	lcl|NZ_LS483362.1_cds_WP_002851218.1_1448	1	573	573	forward	1	573	573	1	573	[gene=yedF] [protein=sulfurtransferase-like selenium metabolism protein YedF] [protein_id=WP_002851218.1] [location=complement(1440183..1440755)] [gbkey=CDS]	573	96.30%	57.30%
[locus_tag=DQM41_RS07470]	lcl|NZ_LS483362.1_cds_WP_002822570.1_1450	1	738	738	forward	1	738	738	1	738	[protein=LysR family transcriptional regulator] [protein_id=WP_002822570.1] [location=complement(1443078..1443815)] [gbkey=CDS]	738	100.00%	73.80%
[locus_tag=DQM41_RS07490]	lcl|NZ_LS483362.1_cds_WP_010891941.1_1454	1471	2157	687	forward	1471	2157	687	1471	2157	[protein=formate dehydrogenase subunit alpha] [transl_except=(pos:541..543,aa:Sec)] [protein_id=WP_010891941.1] [location=complement(1446324..1449128)] [gbkey=CDS]	2805	95.20%	68.70%
[locus_tag=DQM41_RS07525]	lcl|NZ_LS483362.1_cds_WP_002858151.1_1461	115	1113	999	reverse	115	1113	999	115	1113	[protein=molybdopterin molybdotransferase MoeA] [protein_id=WP_002858151.1] [location=1453798..1454988] [gbkey=CDS]	1191	97.00%	99.90%
[locus_tag=DQM41_RS07550]	lcl|NZ_LS483362.1_cds_1466	654	1076	423	reverse	654	1076	423	654	1076	[protein=TRAP transporter large permease subunit] [pseudo=true] [location=1460273..1461466] [gbkey=CDS]	1194	88.70%	42.30%
[locus_tag=DQM41_RS07605]	lcl|NZ_LS483362.1_cds_WP_010891942.1_1477	1	720	720	forward	1	720	720	1	720	[protein=ABC transporter permease] [protein_id=WP_010891942.1] [location=complement(1471500..1472219)] [gbkey=CDS]	720	84.40%	72.00%
[locus_tag=DQM41_RS07710]	lcl|NZ_LS483362.1_cds_WP_010891944.1_1498	42	608	567	reverse	42	608	567	42	608	[protein=methyl-accepting chemotaxis protein] [protein_id=WP_010891944.1] [location=1491986..1493974] [gbkey=CDS]	1989	98.90%	56.70%
[locus_tag=DQM41_RS07815]	lcl|NZ_LS483362.1_cds_WP_002851210.1_1519	1	399	399	forward	1	399	399	1	399	[protein=FAD-binding oxidoreductase] [protein_id=WP_002851210.1] [location=complement(1514627..1517398)] [gbkey=CDS]	2772	89.90%	39.90%
[locus_tag=DQM41_RS07975]	lcl|NZ_LS483362.1_cds_WP_002851399.1_1551	1	390	390	reverse	1	390	390	1	390	[protein=ABC transporter substrate-binding protein] [protein_id=WP_002851399.1] [location=1544653..1545459] [gbkey=CDS]	807	81.50%	39.00%
[locus_tag=DQM41_RS08080]	lcl|NZ_LS483362.1_cds_WP_002851463.1_1568	418	954	537	forward	418	954	537	418	954	[gene=aroC] [protein=chorismate synthase] [protein_id=WP_002851463.1] [location=complement(1559706..1560794)] [gbkey=CDS]	1089	84.90%	53.70%
[locus_tag=DQM41_RS08100]	lcl|NZ_LS483362.1_cds_WP_002851181.1_1572	1	903	903	reverse	1	903	903	1	903	[protein=DNA primase] [protein_id=WP_002851181.1] [location=1562938..1564755] [gbkey=CDS]	1818	92.40%	90.30%
[locus_tag=DQM41_RS08205]	lcl|NZ_LS483362.1_cds_WP_002778842.1_1593	99	134	36	reverse	99	134	36	99	134	[protein=iron transporter] [protein_id=WP_002778842.1] [location=1582906..1583445] [gbkey=CDS]	540	75.00%	3.60%
[locus_tag=DQM41_RS08205]	lcl|NZ_LS483362.1_cds_WP_002778842.1_1593	99	134	36	forward	99	134	36	99	134	[protein=iron transporter] [protein_id=WP_002778842.1] [location=1582906..1583445] [gbkey=CDS]	540	75.00%	3.60%
[locus_tag=DQM41_RS08345]	lcl|NZ_LS483362.1_cds_WP_002851400.1_1616	1	807	807	reverse	1	807	807	1	807	[protein=biotin synthase] [protein_id=WP_002851400.1] [location=complement(1606505..1607341)] [gbkey=CDS]	837	93.70%	80.70%
[locus_tag=DQM41_RS08350]	lcl|NZ_LS483362.1_cds_WP_002851268.1_1617	1	912	912	reverse	1	912	912	1	912	[protein=type I DNA topoisomerase] [protein_id=WP_002851268.1] [location=complement(1607343..1609445)] [gbkey=CDS]	2103	97.00%	91.20%
[locus_tag=DQM41_RS08455]	lcl|NZ_LS483362.1_cds_WP_002864792.1_1638	1	576	576	reverse	1	576	576	1	576	[protein=50S ribosomal protein L3] [protein_id=WP_002864792.1] [location=complement(1619787..1620362)] [gbkey=CDS]	576	99.50%	57.60%
[locus_tag=DQM41_RS08470]	lcl|NZ_LS483362.1_cds_WP_002864793.1_1641	190	1182	993	reverse	190	1182	993	190	1182	[protein=ribonuclease J] [protein_id=WP_002864793.1] [location=complement(1621679..1623673)] [gbkey=CDS]	1995	99.10%	99.30%
[locus_tag=DQM41_RS08480]	lcl|NZ_LS483362.1_cds_WP_002853142.1_1643	1	537	537	forward	1	537	537	1	537	[protein=purine-nucleoside phosphorylase] [protein_id=WP_002853142.1] [location=1624524..1625060] [gbkey=CDS]	537	86.00%	53.70%
[locus_tag=DQM41_RS08570]	lcl|NZ_LS483362.1_cds_WP_002853122.1_1657	1	357	357	reverse	1	357	357	1	357	[protein=O-acetylhomoserine aminocarboxypropyltransferase/cysteine synthase] [protein_id=WP_002853122.1] [location=complement(1636390..1637661)] [gbkey=CDS]	1272	96.60%	35.70%
[locus_tag=DQM41_RS08575]	lcl|NZ_LS483362.1_cds_WP_002882660.1_1658	340	804	465	reverse	340	804	465	340	804	[gene=flgE] [protein=flagellar hook protein FlgE] [protein_id=WP_002882660.1] [location=complement(1638087..1640684)] [gbkey=CDS]	2598	96.80%	46.50%

Table S4: Common genes with putative *C. jejuni* origin of meC0280 and meC0281.

genome	locus_tag	Sequence Name	Minimum	Maximum	Length	Direction	Min (with gaps)	Max (with gaps)	Length (with gaps)	Min (original sequence)	Max (original sequence)	Description	Sequence Length	% Pairwise Identity	Query Coverage
meC0280	[locus_tag=DQM41_RS07815]	lcl|NZ_LS483362.1_cds_WP_002851210.1_1519	169	1167	999	forward	169	1167	999	169	1167	[protein=FAD-binding oxidoreductase] [protein_id=WP_002851210.1] [location=complement(1514627..1517398)] [gbkey=CDS]	2772	91.30%	99.90%
meC0280	[locus_tag=DQM41_RS07815]	lcl|NZ_LS483362.1_cds_WP_002851210.1_1519	1168	2166	999	forward	1168	2166	999	1168	2166	[protein=FAD-binding oxidoreductase] [protein_id=WP_002851210.1] [location=complement(1514627..1517398)] [gbkey=CDS]	2772	98.50%	99.90%
meC0281	[locus_tag=DQM41_RS07815]	lcl|NZ_LS483362.1_cds_WP_002851210.1_1519	325	1323	999	forward	325	1323	999	325	1323	[protein=FAD-binding oxidoreductase] [protein_id=WP_002851210.1] [location=complement(1514627..1517398)] [gbkey=CDS]	2772	95.10%	99.90%
meC0280	[locus_tag=DQM41_RS03100]	lcl|NZ_LS483362.1_cds_WP_002826526.1_598	466	1461	996	forward	466	1461	996	466	1461	[protein=hypothetical protein] [protein_id=WP_002826526.1] [location=580316..581836] [gbkey=CDS]	1521	91.50%	99.60%
meC0281	[locus_tag=DQM41_RS03100]	lcl|NZ_LS483362.1_cds_WP_002826526.1_598	1	948	948	reverse	1	948	948	1	948	[protein=hypothetical protein] [protein_id=WP_002826526.1] [location=580316..581836] [gbkey=CDS]	1521	98.70%	94.80%
meC0280	[locus_tag=DQM41_RS00305]	lcl|NZ_LS483362.1_cds_WP_002864362.1_55	1	843	843	forward	1	843	843	1	843	[protein=hypothetical protein] [protein_id=WP_002864362.1] [location=72001..72855] [gbkey=CDS]	855	97.50%	84.30%
meC0281	[locus_tag=DQM41_RS00305]	lcl|NZ_LS483362.1_cds_WP_002864362.1_55	91	849	759	forward	91	849	759	91	849	[protein=hypothetical protein] [protein_id=WP_002864362.1] [location=72001..72855] [gbkey=CDS]	855	96.80%	75.90%
meC0280	[locus_tag=DQM41_RS01430]	lcl|NZ_LS483362.1_cds_WP_002778123.1_278	447	848	402	reverse	447	848	402	447	848	[protein=pantoate--beta-alanine ligase] [protein_id=WP_002778123.1] [location=complement(271412..272260)] [gbkey=CDS]	849	99.30%	40.20%
meC0281	[locus_tag=DQM41_RS01430]	lcl|NZ_LS483362.1_cds_WP_002778123.1_278	405	848	444	forward	405	848	444	405	848	[protein=pantoate--beta-alanine ligase] [protein_id=WP_002778123.1] [location=complement(271412..272260)] [gbkey=CDS]	849	99.30%	44.40%
meC0280	[locus_tag=DQM41_RS04280]	lcl|NZ_LS483362.1_cds_WP_002864843.1_829	1441	1509	69	forward	1441	1509	69	1441	1509	[protein=chorismate-binding protein] [protein_id=WP_002864843.1] [location=complement(807480..809264)] [gbkey=CDS]	1785	47.80%	6.90%
meC0281	[locus_tag=DQM41_RS04280]	lcl|NZ_LS483362.1_cds_WP_002864843.1_829	1441	1509	69	reverse	1441	1509	69	1441	1509	[protein=chorismate-binding protein] [protein_id=WP_002864843.1] [location=complement(807480..809264)] [gbkey=CDS]	1785	47.80%	6.90%
meC0281	[locus_tag=DQM41_RS04280]	lcl|NZ_LS483362.1_cds_WP_002864843.1_829	1441	1509	69	reverse	1441	1509	69	1441	1509	[protein=chorismate-binding protein] [protein_id=WP_002864843.1] [location=complement(807480..809264)] [gbkey=CDS]	1785	47.80%	6.90%
meC0281	[locus_tag=DQM41_RS04280]	lcl|NZ_LS483362.1_cds_WP_002864843.1_829	1441	1509	69	reverse	1441	1509	69	1441	1509	[protein=chorismate-binding protein] [protein_id=WP_002864843.1] [location=complement(807480..809264)] [gbkey=CDS]	1785	47.80%	6.90%
meC0280	[locus_tag=DQM41_RS03640]	lcl|NZ_LS483362.1_cds_WP_002793291.1_706	484	1326	843	forward	484	1326	843	484	1326	[protein=esterase-like activity of phytase family protein] [protein_id=WP_002793291.1] [location=682515..683840] [gbkey=CDS]	1326	98.60%	84.30%
meC0281	[locus_tag=DQM41_RS03640]	lcl|NZ_LS483362.1_cds_WP_002793291.1_706	1	948	948	reverse	1	948	948	1	948	[protein=esterase-like activity of phytase family protein] [protein_id=WP_002793291.1] [location=682515..683840] [gbkey=CDS]	1326	99.40%	94.80%
meC0280	[locus_tag=DQM41_RS05220]	lcl|NZ_LS483362.1_cds_WP_002864285.1_1006	1903	2628	726	reverse	1903	2628	726	1903	2628	[protein=N-6 DNA methylase] [protein_id=WP_002864285.1] [location=complement(982973..986992)] [gbkey=CDS]	4020	94.20%	72.60%
meC0280	[locus_tag=DQM41_RS05220]	lcl|NZ_LS483362.1_cds_WP_002864285.1_1006	901	1899	999	reverse	901	1899	999	901	1899	[protein=N-6 DNA methylase] [protein_id=WP_002864285.1] [location=complement(982973..986992)] [gbkey=CDS]	4020	100.00%	99.90%
meC0280	[locus_tag=DQM41_RS05220]	lcl|NZ_LS483362.1_cds_WP_002864285.1_1006	1	900	900	reverse	1	900	900	1	900	[protein=N-6 DNA methylase] [protein_id=WP_002864285.1] [location=complement(982973..986992)] [gbkey=CDS]	4020	99.00%	90.00%
meC0281	[locus_tag=DQM41_RS05220]	lcl|NZ_LS483362.1_cds_WP_002864285.1_1006	466	1464	999	forward	466	1464	999	466	1464	[protein=N-6 DNA methylase] [protein_id=WP_002864285.1] [location=complement(982973..986992)] [gbkey=CDS]	4020	99.70%	99.90%
meC0281	[locus_tag=DQM41_RS05220]	lcl|NZ_LS483362.1_cds_WP_002864285.1_1006	1465	2463	999	forward	1465	2463	999	1465	2463	[protein=N-6 DNA methylase] [protein_id=WP_002864285.1] [location=complement(982973..986992)] [gbkey=CDS]	4020	98.20%	99.90%
meC0281	[locus_tag=DQM41_RS05220]	lcl|NZ_LS483362.1_cds_WP_002864285.1_1006	3391	4020	630	forward	3391	4020	630	3391	4020	[protein=N-6 DNA methylase] [protein_id=WP_002864285.1] [location=complement(982973..986992)] [gbkey=CDS]	4020	93.60%	63.00%
meC0280	[locus_tag=DQM41_RS05910]	lcl|NZ_LS483362.1_cds_WP_002852850.1_1144	46	519	474	forward	46	519	474	46	519	[protein=cation:dicarboxylase symporter family transporter] [protein_id=WP_002852850.1] [location=complement(1119951..1121327] [gbkey=CDS]	1377	98.70%	47.40%
meC0281	[locus_tag=DQM41_RS05910]	lcl|NZ_LS483362.1_cds_WP_002852850.1_1144	817	1377	561	reverse	817	1377	561	817	1377	[protein=cation:dicarboxylase symporter family transporter] [protein_id=WP_002852850.1] [location=complement(1119951..1121327] [gbkey=CDS]	1377	100.00%	56.10%
meC0281	[locus_tag=DQM41_RS05910]	lcl|NZ_LS483362.1_cds_WP_002852850.1_1144	46	813	768	reverse	46	813	768	46	813	[protein=cation:dicarboxylase symporter family transporter] [protein_id=WP_002852850.1] [location=complement(1119951..1121327] [gbkey=CDS]	1377	94.70%	76.80%
meC0280	[locus_tag=DQM41_RS00245]	lcl|NZ_LS483362.1_cds_WP_025096273.1_44	214	279	66	forward	214	279	66	214	279	[protein=bacteriohemerythrin] [protein_id=WP_025096273.1] [location=complement(65696..66463)] [gbkey=CDS]	768	40.90%	6.60%
meC0281	[locus_tag=DQM41_RS00245]	lcl|NZ_LS483362.1_cds_WP_025096273.1_44	166	717	552	reverse	166	717	552	166	717	[protein=bacteriohemerythrin] [protein_id=WP_025096273.1] [location=complement(65696..66463)] [gbkey=CDS]	768	92.90%	55.20%
meC0281	[locus_tag=DQM41_RS00245]	lcl|NZ_LS483362.1_cds_WP_025096273.1_44	1	162	162	reverse	1	162	162	1	162	[protein=bacteriohemerythrin] [protein_id=WP_025096273.1] [location=complement(65696..66463)] [gbkey=CDS]	768	79.60%	16.20%
meC0280	[locus_tag=DQM41_RS03110]	lcl|NZ_LS483362.1_cds_WP_002776330.1_600	1	693	693	reverse	1	693	693	1	693	[gene=hypB] [protein=hydrogenase nickel incorporation protein HypB] [protein_id=WP_002776330.1] [location=584084..584827] [gbkey=CDS]	744	94.60%	69.30%
meC0281	[locus_tag=DQM41_RS03110]	lcl|NZ_LS483362.1_cds_WP_002776330.1_600	187	744	558	reverse	187	744	558	187	744	[gene=hypB] [protein=hydrogenase nickel incorporation protein HypB] [protein_id=WP_002776330.1] [location=584084..584827] [gbkey=CDS]	744	99.50%	55.80%
meC0280	[locus_tag=DQM41_RS01370]	lcl|NZ_LS483362.1_cds_WP_002851649.1_267	1150	2148	999	reverse	1150	2148	999	1150	2148	[protein=response regulator] [protein_id=WP_002851649.1] [location=complement(260898..263207)] [gbkey=CDS]	2310	95.30%	99.90%
meC0281	[locus_tag=DQM41_RS01370]	lcl|NZ_LS483362.1_cds_WP_002851649.1_267	457	1119	663	forward	457	1119	663	457	1119	[protein=response regulator] [protein_id=WP_002851649.1] [location=complement(260898..263207)] [gbkey=CDS]	2310	81.00%	66.30%
meC0280	[locus_tag=DQM41_RS06625]	lcl|NZ_LS483362.1_cds_WP_010891924.1_1284	169	960	792	forward	169	960	792	169	960	[gene=pseD] [protein=flagellin modification protein PseD] [protein_id=WP_010891924.1] [location=1259658..1261619] [gbkey=CDS]	1962	83.30%	79.20%
meC0281	[locus_tag=DQM41_RS06625]	lcl|NZ_LS483362.1_cds_WP_010891924.1_1284	124	789	666	reverse	124	789	666	124	789	[gene=pseD] [protein=flagellin modification protein PseD] [protein_id=WP_010891924.1] [location=1259658..1261619] [gbkey=CDS]	1962	89.20%	66.60%
meC0280	[locus_tag=DQM41_RS07465]	lcl|NZ_LS483362.1_cds_WP_002851362.1_1449	1516	2103	588	reverse	1516	2103	588	1516	2103	[gene=ccaA] [protein=aspartate chemotaxis receptor CcaA] [protein_id=WP_002851362.1] [location=complement(1440866..1442968)] [gbkey=CDS]	2103	94.00%	58.80%
meC0280	[locus_tag=DQM41_RS07465]	lcl|NZ_LS483362.1_cds_WP_002851362.1_1449	517	1512	996	reverse	517	1512	996	517	1512	[gene=ccaA] [protein=aspartate chemotaxis receptor CcaA] [protein_id=WP_002851362.1] [location=complement(1440866..1442968)] [gbkey=CDS]	2103	97.90%	99.60%
meC0281	[locus_tag=DQM41_RS07465]	lcl|NZ_LS483362.1_cds_WP_002851362.1_1449	307	642	336	forward	307	642	336	307	642	[gene=ccaA] [protein=aspartate chemotaxis receptor CcaA] [protein_id=WP_002851362.1] [location=complement(1440866..1442968)] [gbkey=CDS]	2103	98.20%	33.60%
meC0281	[locus_tag=DQM41_RS07465]	lcl|NZ_LS483362.1_cds_WP_002851362.1_1449	1645	2103	459	forward	1645	2103	459	1645	2103	[gene=ccaA] [protein=aspartate chemotaxis receptor CcaA] [protein_id=WP_002851362.1] [location=complement(1440866..1442968)] [gbkey=CDS]	2103	100.00%	45.90%
meC0280	[locus_tag=DQM41_RS06505]	lcl|NZ_LS483362.1_cds_WP_002856137.1_1260	586	1068	483	reverse	586	1068	483	586	1068	[protein=hypothetical protein] [protein_id=WP_002856137.1] [location=complement(1238565..1239635)] [gbkey=CDS]	1071	100.00%	48.30%
meC0281	[locus_tag=DQM41_RS06505]	lcl|NZ_LS483362.1_cds_WP_002856137.1_1260	280	936	657	forward	280	936	657	280	936	[protein=hypothetical protein] [protein_id=WP_002856137.1] [location=complement(1238565..1239635)] [gbkey=CDS]	1071	99.10%	65.70%
meC0280	[locus_tag=DQM41_RS07595]	lcl|NZ_LS483362.1_cds_WP_002851288.1_1475	502	1497	996	reverse	502	1497	996	502	1497	[gene=acs] [protein=acetate--CoA ligase] [protein_id=WP_002851288.1] [location=complement(1468392..1470365)] [gbkey=CDS]	1974	96.20%	99.60%
meC0281	[locus_tag=DQM41_RS07595]	lcl|NZ_LS483362.1_cds_WP_002851288.1_1475	1498	1746	249	reverse	1498	1746	249	1498	1746	[gene=acs] [protein=acetate--CoA ligase] [protein_id=WP_002851288.1] [location=complement(1468392..1470365)] [gbkey=CDS]	1974	47.00%	24.90%
meC0280	[locus_tag=DQM41_RS00320]	lcl|NZ_LS483362.1_cds_WP_002851710.1_58	508	978	471	reverse	508	978	471	508	978	[gene=fliM] [protein=flagellar motor switch protein FliM] [protein_id=WP_002851710.1] [location=complement(74327..75406)] [gbkey=CDS]	1080	93.30%	47.10%
meC0281	[locus_tag=DQM41_RS00320]	lcl|NZ_LS483362.1_cds_WP_002851710.1_58	316	978	663	reverse	316	978	663	316	978	[gene=fliM] [protein=flagellar motor switch protein FliM] [protein_id=WP_002851710.1] [location=complement(74327..75406)] [gbkey=CDS]	1080	95.20%	66.30%
meC0280	[locus_tag=DQM41_RS07490]	lcl|NZ_LS483362.1_cds_WP_010891941.1_1454	1	717	717	reverse	1	717	717	1	717	[protein=formate dehydrogenase subunit alpha] [transl_except=(pos:541..543,aa:Sec)] [protein_id=WP_010891941.1] [location=complement(1446324..1449128)] [gbkey=CDS]	2805	91.00%	71.70%
meC0281	[locus_tag=DQM41_RS07490]	lcl|NZ_LS483362.1_cds_WP_010891941.1_1454	847	1422	576	forward	847	1422	576	847	1422	[protein=formate dehydrogenase subunit alpha] [transl_except=(pos:541..543,aa:Sec)] [protein_id=WP_010891941.1] [location=complement(1446324..1449128)] [gbkey=CDS]	2805	97.40%	57.60%
meC0280	[locus_tag=DQM41_RS03945]	lcl|NZ_LS483362.1_cds_WP_002864146.1_762	298	423	126	forward	298	423	126	298	423	[protein=hypothetical protein] [protein_id=WP_002864146.1] [location=744480..745760] [gbkey=CDS]	1281	95.20%	12.60%
meC0281	[locus_tag=DQM41_RS03945]	lcl|NZ_LS483362.1_cds_WP_002864146.1_762	910	1281	372	reverse	910	1281	372	910	1281	[protein=hypothetical protein] [protein_id=WP_002864146.1] [location=744480..745760] [gbkey=CDS]	1281	80.60%	37.20%
meC0280	[locus_tag=DQM41_RS02380]	lcl|NZ_LS483362.1_cds_WP_010891860.1_458	1810	2808	999	forward	1810	2808	999	1810	2808	[gene=rpoB] [protein=DNA-directed RNA polymerase subunit beta] [protein_id=WP_010891860.1] [location=438986..443122] [gbkey=CDS]	4137	99.70%	99.90%
meC0281	[locus_tag=DQM41_RS02380]	lcl|NZ_LS483362.1_cds_WP_010891860.1_458	2131	3129	999	reverse	2131	3129	999	2131	3129	[gene=rpoB] [protein=DNA-directed RNA polymerase subunit beta] [protein_id=WP_010891860.1] [location=438986..443122] [gbkey=CDS]	4137	99.70%	99.90%
meC0281	[locus_tag=DQM41_RS02380]	lcl|NZ_LS483362.1_cds_WP_010891860.1_458	1132	2127	996	reverse	1132	2127	996	1132	2127	[gene=rpoB] [protein=DNA-directed RNA polymerase subunit beta] [protein_id=WP_010891860.1] [location=438986..443122] [gbkey=CDS]	4137	98.00%	99.60%
meC0280	[locus_tag=DQM41_RS01440]	lcl|NZ_LS483362.1_cds_WP_002783228.1_280	1	498	498	forward	1	498	498	1	498	[protein=OXA-61 family class D beta-lactamase OXA-193] [protein_id=WP_002783228.1] [location=273315..274088] [gbkey=CDS]	774	99.40%	49.80%
meC0281	[locus_tag=DQM41_RS01440]	lcl|NZ_LS483362.1_cds_WP_002783228.1_280	1	543	543	reverse	1	543	543	1	543	[protein=OXA-61 family class D beta-lactamase OXA-193] [protein_id=WP_002783228.1] [location=273315..274088] [gbkey=CDS]	774	100.00%	54.30%
meC0280	[locus_tag=DQM41_RS01770]	lcl|NZ_LS483362.1_cds_WP_002872052.1_346	1080	2063	984	reverse	1080	2063	984	1080	2063	[gene=cmeB] [protein=multidrug efflux RND transporter permease subunit CmeB] [protein_id=WP_002872052.1] [location=complement(332589..335711)] [gbkey=CDS]	3123	92.90%	98.40%
meC0281	[locus_tag=DQM41_RS01770]	lcl|NZ_LS483362.1_cds_WP_002872052.1_346	85	1044	960	reverse	85	1044	960	85	1044	[gene=cmeB] [protein=multidrug efflux RND transporter permease subunit CmeB] [protein_id=WP_002872052.1] [location=complement(332589..335711)] [gbkey=CDS]	3123	95.20%	96.00%
meC0281	[locus_tag=DQM41_RS01770]	lcl|NZ_LS483362.1_cds_WP_002872052.1_346	1048	2046	999	forward	1048	2046	999	1048	2046	[gene=cmeB][protein=multidrug efflux RND transporter permease subunit CmeB] [protein_id=WP_002872052.1] [location=complement(332589..335711)] [gbkey=CDS]	3123	86.40%	99.90%
meC0280	[locus_tag=DQM41_RS02395]	lcl|NZ_LS483362.1_cds_WP_002858487.1_461	112	909	798	reverse	112	909	798	112	909	[protein=dihydrodipicolinate synthase family protein] [protein_id=WP_002858487.1] [location=448691..449599] [gbkey=CDS]	909	94.00%	79.80%
meC0281	[locus_tag=DQM41_RS02395]	lcl|NZ_LS483362.1_cds_WP_002858487.1_461	433	909	477	reverse	433	909	477	433	909	[protein=dihydrodipicolinate synthase family protein] [protein_id=WP_002858487.1] [location=448691..449599] [gbkey=CDS]	909	98.10%	47.70%
meC0280	[locus_tag=DQM41_RS06605]	lcl|NZ_LS483362.1_cds_WP_002864264.1_1280	358	1026	669	forward	358	1026	669	358	1026	[protein=CBS domain-containing protein] [protein_id=WP_002864264.1] [location=1256273..1257298] [gbkey=CDS]	1026	98.20%	66.90%
meC0281	[locus_tag=DQM41_RS06605]	lcl|NZ_LS483362.1_cds_WP_002864264.1_1280	1	984	984	reverse	1	984	984	1	984	[protein=CBS domain-containing protein] [protein_id=WP_002864264.1] [location=1256273..1257298] [gbkey=CDS]	1026	95.00%	98.40%
meC0280	[locus_tag=DQM41_RS02720]	lcl|NZ_LS483362.1_cds_WP_002864504.1_525	43	690	648	forward	43	690	648	43	690	[gene=fliD] [protein=flagellar filament capping protein FliD] [protein_id=WP_002864504.1] [location=510546..512474] [gbkey=CDS]	1929	93.10%	64.80%
meC0281	[locus_tag=DQM41_RS02720]	lcl|NZ_LS483362.1_cds_WP_002864504.1_525	412	1410	999	reverse	412	1410	999	412	1410	[gene=fliD] [protein=flagellar filament capping protein FliD] [protein_id=WP_002864504.1] [location=510546..512474] [gbkey=CDS]	1929	96.10%	99.90%
meC0280	[locus_tag=DQM41_RS05225]	lcl|NZ_LS483362.1_cds_WP_010891903.1_1007	121	1119	999	reverse	121	1119	999	121	1119	[protein=endonuclease MutS2] [protein_id=WP_010891903.1] [location=complement(987001..989211)] [gbkey=CDS]	2211	99.40%	99.90%
meC0281	[locus_tag=DQM41_RS05225]	lcl|NZ_LS483362.1_cds_WP_010891903.1_1007	685	1500	816	forward	685	1500	816	685	1500	[protein=endonuclease MutS2] [protein_id=WP_010891903.1] [location=complement(987001..989211)] [gbkey=CDS]	2211	100.00%	81.60%
meC0280	[locus_tag=DQM41_RS05130]	lcl|NZ_LS483362.1_cds_WP_010891901.1_988	781	1779	999	forward	781	1779	999	781	1779	[gene=cmeF] [protein=multidrug efflux RND transporter permease subunit CmeF] [protein_id=WP_010891901.1] [location=965475..968492] [gbkey=CDS]	3018	91.40%	99.90%
meC0280	[locus_tag=DQM41_RS05130]	lcl|NZ_LS483362.1_cds_WP_010891901.1_988	1780	2778	999	forward	1780	2778	999	1780	2778	[gene=cmeF] [protein=multidrug efflux RND transporter permease subunit CmeF] [protein_id=WP_010891901.1] [location=965475..968492] [gbkey=CDS]	3018	96.30%	99.90%
meC0280	[locus_tag=DQM41_RS05130]	lcl|NZ_LS483362.1_cds_WP_010891901.1_988	2217	2615	399	reverse	2217	2615	399	2217	2615	[gene=cmeF] [protein=multidrug efflux RND transporter permease subunit CmeF] [protein_id=WP_010891901.1] [location=965475..968492] [gbkey=CDS]	3018	100.00%	39.90%
meC0281	[locus_tag=DQM41_RS05130]	lcl|NZ_LS483362.1_cds_WP_010891901.1_988	1216	2214	999	reverse	1216	2214	999	1216	2214	[gene=cmeF] [protein=multidrug efflux RND transporter permease subunit CmeF] [protein_id=WP_010891901.1] [location=965475..968492] [gbkey=CDS]	3018	98.50%	99.90%
meC0281	[locus_tag=DQM41_RS05130]	lcl|NZ_LS483362.1_cds_WP_010891901.1_988	217	1173	957	reverse	217	1173	957	217	1173	[gene=cmeF] [protein=multidrug efflux RND transporter permease subunit CmeF] [protein_id=WP_010891901.1] [location=965475..968492] [gbkey=CDS]	3018	98.10%	95.70%
meC0280	[locus_tag=DQM41_RS05900]	lcl|NZ_LS483362.1_cds_WP_002790076.1_1142	817	1380	564	reverse	817	1380	564	817	1380	[gene=cetA] [protein=bipartate energy taxis response protein CetA] [protein_id=WP_002790076.1] [location=complement(1117688..1119067)] [gbkey=CDS]	1380	99.50%	56.40%
meC0280	[locus_tag=DQM41_RS05900]	lcl|NZ_LS483362.1_cds_WP_002790076.1_1142	1	597	597	reverse	1	597	597	1	597	[gene=cetA] [protein=bipartate energy taxis response protein CetA] [protein_id=WP_002790076.1] [location=complement(1117688..1119067)] [gbkey=CDS]	1380	99.50%	59.70%
meC0281	[locus_tag=DQM41_RS05900]	lcl|NZ_LS483362.1_cds_WP_002790076.1_1142	817	1296	480	forward	817	1296	480	817	1296	[gene=cetA] [protein=bipartate energy taxis response protein CetA] [protein_id=WP_002790076.1] [location=complement(1117688..1119067)] [gbkey=CDS]	1380	98.80%	48.00%
meC0280	[locus_tag=DQM41_RS06910]	lcl|NZ_LS483362.1_cds_1339	906	1439	534	forward	906	1439	534	906	1439	[protein=C4-dicarboxylate ABC transporter] [pseudo=true] [location=1325650..1327089] [gbkey=CDS]	1440	96.10%	53.40%
meC0281	[locus_tag=DQM41_RS06910]	lcl|NZ_LS483362.1_cds_1339	136	450	315	reverse	136	450	315	136	450	[protein=C4-dicarboxylate ABC transporter] [pseudo=true] [location=1325650..1327089] [gbkey=CDS]	1440	95.20%	31.50%
meC0280	[locus_tag=DQM41_RS06650]	lcl|NZ_LS483362.1_cds_WP_002852395.1_1289	436	1203	768	reverse	436	1203	768	436	1203	[protein=flagellin A] [protein_id=WP_002852395.1] [location=complement(1267350..1269068)] [gbkey=CDS]	1719	95.70%	76.80%
meC0281	[locus_tag=DQM41_RS06650]	lcl|NZ_LS483362.1_cds_WP_002852395.1_1289	1	798	798	forward	1	798	798	1	798	[protein=flagellin A] [protein_id=WP_002852395.1] [location=complement(1267350..1269068)] [gbkey=CDS]	1719	99.20%	79.80%
meC0281	[locus_tag=DQM41_RS06650]	lcl|NZ_LS483362.1_cds_WP_002852395.1_1289	1282	1719	438	forward	1282	1719	438	1282	1719	[protein=flagellin A] [protein_id=WP_002852395.1] [location=complement(1267350..1269068)] [gbkey=CDS]	1719	97.30%	43.80%
meC0280	[locus_tag=DQM41_RS03760]	lcl|NZ_LS483362.1_cds_WP_002780052.1_725	1	531	531	forward	1	531	531	1	531	[gene=grpE] [protein=nucleotide exchange factor GrpE] [protein_id=WP_002780052.1] [location=708616..709146] [gbkey=CDS]	531	90.80%	53.10%
meC0281	[locus_tag=DQM41_RS03760]	lcl|NZ_LS483362.1_cds_WP_002780052.1_725	1	531	531	forward	1	531	531	1	531	[gene=grpE] [protein=nucleotide exchange factor GrpE] [protein_id=WP_002780052.1] [location=708616..709146] [gbkey=CDS]	531	91.30%	53.10%
meC0280	[locus_tag=DQM41_RS02385]	lcl|NZ_LS483362.1_cds_WP_002858508.1_459	3685	4554	870	forward	3685	4554	870	3685	4554	[gene=rpoC] [protein=DNA-directed RNA polymerase subunit beta'] [protein_id=WP_002858508.1] [location=443115..447668] [gbkey=CDS]	4554	96.60%	87.00%
meC0281	[locus_tag=DQM41_RS02385]	lcl|NZ_LS483362.1_cds_WP_002858508.1_459	4006	4554	549	reverse	4006	4554	549	4006	4554	[gene=rpoC] [protein=DNA-directed RNA polymerase subunit beta'] [protein_id=WP_002858508.1] [location=443115..447668] [gbkey=CDS]	4554	96.70%	54.90%
meC0281	[locus_tag=DQM41_RS02385]	lcl|NZ_LS483362.1_cds_WP_002858508.1_459	2002	3000	999	reverse	2002	3000	999	2002	3000	[gene=rpoC] [protein=DNA-directed RNA polymerase subunit beta'] [protein_id=WP_002858508.1] [location=443115..447668] [gbkey=CDS]	4554	95.80%	99.90%
meC0281	[locus_tag=DQM41_RS02385]	lcl|NZ_LS483362.1_cds_WP_002858508.1_459	1	999	999	reverse	1	999	999	1	999	[gene=rpoC] [protein=DNA-directed RNA polymerase subunit beta'] [protein_id=WP_002858508.1] [location=443115..447668] [gbkey=CDS]	4554	86.70%	99.90%
meC0280	[locus_tag=DQM41_RS05125]	lcl|NZ_LS483362.1_cds_WP_002858463.1_987	1	522	522	forward	1	522	522	1	522	[gene=cmeE] [protein=multidrug efflux RND transporter periplasmic adaptor subunit CmeE] [protein_id=WP_002858463.1] [location=964732..965472] [gbkey=CDS]	741	92.30%	52.20%
meC0281	[locus_tag=DQM41_RS05125]	lcl|NZ_LS483362.1_cds_WP_002858463.1_987	1	741	741	reverse	1	741	741	1	741	[gene=cmeE] [protein=multidrug efflux RND transporter periplasmic adaptor subunit CmeE] [protein_id=WP_002858463.1] [location=964732..965472] [gbkey=CDS]	741	97.60%	74.10%
meC0280	[locus_tag=DQM41_RS05115]	lcl|NZ_LS483362.1_cds_WP_002837927.1_985	235	999	765	reverse	235	999	765	235	999	[gene=lepA] [protein=elongation factor 4] [protein_id=WP_002837927.1] [location=complement(961538..963334)] [gbkey=CDS]	1797	97.30%	76.50%
meC0281	[locus_tag=DQM41_RS05115]	lcl|NZ_LS483362.1_cds_WP_002837927.1_985	811	1647	837	forward	811	1647	837	811	1647	[gene=lepA][protein=elongation factor 4] [protein_id=WP_002837927.1] [location=complement(961538..963334)] [gbkey=CDS]	1797	95.20%	83.70%
meC0280	[locus_tag=DQM41_RS00120]	lcl|NZ_LS483362.1_cds_WP_002865087.1_24	1	564	564	forward	1	564	564	1	564	[protein=ribonucleoside-diphosphate reductase subunit alpha] [protein_id=WP_002865087.1] [location=complement(29726..32095)] [gbkey=CDS]	2370	97.90%	56.40%
meC0281	[locus_tag=DQM41_RS00120]	lcl|NZ_LS483362.1_cds_WP_002865087.1_24	904	1902	999	forward	904	1902	999	904	1902	[protein=ribonucleoside-diphosphate reductase subunit alpha] [protein_id=WP_002865087.1] [location=complement(29726..32095)] [gbkey=CDS]	2370	97.90%	99.90%
meC0281	[locus_tag=DQM41_RS00120]	lcl|NZ_LS483362.1_cds_WP_002865087.1_24	1906	2370	465	forward	1906	2370	465	1906	2370	[protein=ribonucleoside-diphosphate reductase subunit alpha] [protein_id=WP_002865087.1] [location=complement(29726..32095)] [gbkey=CDS]	2370	98.70%	46.50%
meC0280	[locus_tag=DQM41_RS00140]	lcl|NZ_LS483362.1_cds_WP_002853146.1_28	307	1302	996	forward	307	1302	996	307	1302	[gene=recJ] [protein=single-stranded-DNA-specific exonuclease RecJ] [protein_id=WP_002853146.1] [location=36000..37571] [gbkey=CDS]	1572	90.10%	99.60%
meC0281	[locus_tag=DQM41_RS00140]	lcl|NZ_LS483362.1_cds_WP_002853146.1_28	355	627	273	forward	355	627	273	355	627	[gene=recJ] [protein=single-stranded-DNA-specific exonuclease RecJ] [protein_id=WP_002853146.1] [location=36000..37571] [gbkey=CDS]	1572	100.00%	27.30%
meC0280	[locus_tag=DQM41_RS03080]	lcl|NZ_LS483362.1_cds_WP_010891867.1_594	1	564	564	forward	1	564	564	1	564	[protein=phosphate ABC transporter ATP-binding protein] [protein_id=WP_010891867.1] [location=576216..576956] [gbkey=CDS]	741	99.50%	56.40%
meC0281	[locus_tag=DQM41_RS03080]	lcl|NZ_LS483362.1_cds_WP_010891867.1_594	52	741	690	reverse	52	741	690	52	741	[protein=phosphate ABC transporter ATP-binding protein] [protein_id=WP_010891867.1] [location=576216..576956] [gbkey=CDS]	741	99.60%	69.00%
meC0280	[locus_tag=DQM41_RS02420]	lcl|NZ_LS483362.1_cds_WP_002864166.1_466	3	452	450	reverse	3	452	450	3	452	[gene=fucP] [protein=L-fucose:H+ symporter permease] [protein_id=WP_002864166.1] [location=453113..454369] [gbkey=CDS]	1257	90.40%	45.00%
meC0280	[locus_tag=DQM41_RS02420]	lcl|NZ_LS483362.1_cds_WP_002864166.1_466	676	1257	582	reverse	676	1257	582	676	1257	[gene=fucP] [protein=L-fucose:H+ symporter permease] [protein_id=WP_002864166.1] [location=453113..454369] [gbkey=CDS]	1257	100.00%	58.20%
meC0281	[locus_tag=DQM41_RS02420]	lcl|NZ_LS483362.1_cds_WP_002864166.1_466	1	561	561	reverse	1	561	561	1	561	[gene=fucP] [protein=L-fucose:H+ symporter permease] [protein_id=WP_002864166.1] [location=453113..454369] [gbkey=CDS]	1257	89.10%	56.10%
meC0280	[locus_tag=DQM41_RS02050]	lcl|NZ_LS483362.1_cds_WP_002858716.1_402	490	957	468	reverse	490	957	468	490	957	[protein=membrane protein] [protein_id=WP_002858716.1] [location=complement(385765..386721)] [gbkey=CDS]	957	92.00%	46.80%
meC0281	[locus_tag=DQM41_RS02050]	lcl|NZ_LS483362.1_cds_WP_002858716.1_402	490	957	468	forward	490	957	468	490	957	[protein=membrane protein] [protein_id=WP_002858716.1] [location=complement(385765..386721)] [gbkey=CDS]	957	91.40%	46.80%
meC0280	[locus_tag=DQM41_RS03750]	lcl|NZ_LS483362.1_cds_WP_010891883.1_723	1021	2019	999	forward	1021	2019	999	1021	2019	[gene=cfrA][protein=TonB-dependent ferric enterobactin receptor CfrA] [protein_id=WP_010891883.1] [location=705435..707525] [gbkey=CDS]	2091	94.90%	99.90%
meC0281	[locus_tag=DQM41_RS03750]	lcl|NZ_LS483362.1_cds_WP_010891883.1_723	841	1836	996	reverse	841	1836	996	841	1836	[gene=cfrA] [protein=TonB-dependent ferric enterobactin receptor CfrA] [protein_id=WP_010891883.1] [location=705435..707525] [gbkey=CDS]	2091	99.70%	99.60%
meC0280	[locus_tag=DQM41_RS06490]	lcl|NZ_LS483362.1_cds_WP_002858432.1_1257	733	1221	489	reverse	733	1221	489	733	1221	[protein=DUF2920 family protein] [protein_id=WP_002858432.1] [location=complement(1235506..1236732)] [gbkey=CDS]	1227	89.00%	48.90%
meC0280	[locus_tag=DQM41_RS06490]	lcl|NZ_LS483362.1_cds_WP_002858432.1_1257	1	636	636	reverse	1	636	636	1	636	[protein=DUF2920 family protein] [protein_id=WP_002858432.1] [location=complement(1235506..1236732)] [gbkey=CDS]	1227	95.50%	63.60%
meC0281	[locus_tag=DQM41_RS06490]	lcl|NZ_LS483362.1_cds_WP_002858432.1_1257	637	1227	591	forward	637	1227	591	637	1227	[protein=DUF2920 family protein] [protein_id=WP_002858432.1] [location=complement(1235506..1236732)] [gbkey=CDS]	1227	99.50%	59.10%
meC0281	[locus_tag=DQM41_RS06490]	lcl|NZ_LS483362.1_cds_WP_002858432.1_1257	733	1221	489	forward	733	1221	489	733	1221	[protein=DUF2920 family protein] [protein_id=WP_002858432.1] [location=complement(1235506..1236732)] [gbkey=CDS]	1227	86.50%	48.90%
meC0280	[locus_tag=DQM41_RS08350]	lcl|NZ_LS483362.1_cds_WP_002851268.1_1617	598	1596	999	reverse	598	1596	999	598	1596	[protein=type I DNA topoisomerase] [protein_id=WP_002851268.1] [location=complement(1607343..1609445)] [gbkey=CDS]	2103	96.10%	99.90%
meC0281	[locus_tag=DQM41_RS08350]	lcl|NZ_LS483362.1_cds_WP_002851268.1_1617	169	1167	999	reverse	169	1167	999	169	1167	[protein=type I DNA topoisomerase] [protein_id=WP_002851268.1] [location=complement(1607343..1609445)] [gbkey=CDS]	2103	93.90%	99.90%
meC0280	[locus_tag=DQM41_RS05780]	lcl|NZ_LS483362.1_cds_WP_002852826.1_1118	292	777	486	reverse	292	777	486	292	777	[protein=threonine/serine exporter ThrE family protein] [protein_id=WP_002852826.1] [location=complement(1095911..1096687)] [gbkey=CDS]	777	87.70%	48.60%
meC0281	[locus_tag=DQM41_RS05780]	lcl|NZ_LS483362.1_cds_WP_002852826.1_1118	1	777	777	forward	1	777	777	1	777	[protein=threonine/serine exporter ThrE family protein] [protein_id=WP_002852826.1] [location=complement(1095911..1096687)] [gbkey=CDS]	777	88.00%	77.70%
meC0280	[locus_tag=DQM41_RS00190]	lcl|NZ_LS483362.1_cds_WP_002866314.1_33	64	690	627	forward	64	690	627	64	690	[protein=SIMPL domain-containing protein] [protein_id=WP_002866314.1] [location=complement(51963..52664)] [gbkey=CDS]	702	90.40%	62.70%
meC0281	[locus_tag=DQM41_RS00190]	lcl|NZ_LS483362.1_cds_WP_002866314.1_33	64	543	480	reverse	64	543	480	64	543	[protein=SIMPL domain-containing protein] [protein_id=WP_002866314.1] [location=complement(51963..52664)] [gbkey=CDS]	702	93.10%	48.00%
meC0280	[locus_tag=DQM41_RS06915]	lcl|NZ_LS483362.1_cds_1340	120	1013	894	forward	120	1013	894	120	1013	[protein=PLP-dependent transferase] [pseudo=true] [location=1327102..1328264] [gbkey=CDS]	1163	98.00%	89.40%
meC0281	[locus_tag=DQM41_RS06915]	lcl|NZ_LS483362.1_cds_1340	633	1163	531	forward	633	1163	531	633	1163	[protein=PLP-dependent transferase] [pseudo=true] [location=1327102..1328264] [gbkey=CDS]	1163	94.90%	53.10%
meC0281	[locus_tag=DQM41_RS06915]	lcl|NZ_LS483362.1_cds_1340	120	632	513	reverse	120	632	513	120	632	[protein=PLP-dependent transferase] [pseudo=true] [location=1327102..1328264] [gbkey=CDS]	1163	97.70%	51.30%
meC0280	[locus_tag=DQM41_RS06570]	lcl|NZ_LS483362.1_cds_WP_010891923.1_1273	185	1075	891	forward	185	1075	891	185	1075	[gene=legC] [protein=aminotransferase LegC] [protein_id=WP_010891923.1] [location=1249570..1250724] [gbkey=CDS]	1155	95.00%	89.10%
meC0281	[locus_tag=DQM41_RS06570]	lcl|NZ_LS483362.1_cds_WP_010891923.1_1273	703	1155	453	reverse	703	1155	453	703	1155	[gene=legC] [protein=aminotransferase LegC] [protein_id=WP_010891923.1] [location=1249570..1250724] [gbkey=CDS]	1155	99.30%	45.30%

Table S5: Common genes with putative *C. jejuni* origin of meC0280 and meC0467.

genome	locus_tag	Sequence Name	Minimum	Maximum	Length	Direction	Min (with gaps)	Max (with gaps)	Length (with gaps)	Min (original sequence)	Max (original sequence)	Description	Sequence Length	% Pairwise Identity	Query Coverage
meC0280	[locus_tag=DQM41_RS01520]	lcl|NZ_LS483362.1_cds_WP_002858702.1_296	1	645	645	forward	1	645	645	1	645	[protein=HAD-IIA family hydrolase] [protein_id=WP_002858702.1] [location=285487..286254] [gbkey=CDS]	768	99.50%	64.50%
meC0467	[locus_tag=DQM41_RS01520]	lcl|NZ_LS483362.1_cds_WP_002858702.1_296	115	768	654	reverse	115	768	654	115	768	[protein=HAD-IIA family hydrolase] [protein_id=WP_002858702.1] [location=285487..286254] [gbkey=CDS]	768	93.80%	65.40%
meC0280	[locus_tag=DQM41_RS07815]	lcl|NZ_LS483362.1_cds_WP_002851210.1_1519	169	1167	999	reverse	169	1167	999	169	1167	[protein=FAD-binding oxidoreductase] [protein_id=WP_002851210.1] [location=complement(1514627..1517398)] [gbkey=CDS]	2772	91.30%	99.90%
meC0280	[locus_tag=DQM41_RS07815]	lcl|NZ_LS483362.1_cds_WP_002851210.1_1519	1168	2166	999	reverse	1168	2166	999	1168	2166	[protein=FAD-binding oxidoreductase] [protein_id=WP_002851210.1] [location=complement(1514627..1517398)] [gbkey=CDS]	2772	98.50%	99.90%
meC0467	[locus_tag=DQM41_RS07815]	lcl|NZ_LS483362.1_cds_WP_002851210.1_1519	1	399	399	forward	1	399	399	1	399	[protein=FAD-binding oxidoreductase] [protein_id=WP_002851210.1] [location=complement(1514627..1517398)] [gbkey=CDS]	2772	89.90%	39.90%
meC0280	[locus_tag=DQM41_RS03100]	lcl|NZ_LS483362.1_cds_WP_002826526.1_598	466	1461	996	forward	466	1461	996	466	1461	[protein=hypothetical protein] [protein_id=WP_002826526.1] [location=580316..581836] [gbkey=CDS]	1521	91.50%	99.60%
meC0467	[locus_tag=DQM41_RS03100]	lcl|NZ_LS483362.1_cds_WP_002826526.1_598	343	1338	996	reverse	343	1338	996	343	1338	[protein=hypothetical protein] [protein_id=WP_002826526.1] [location=580316..581836] [gbkey=CDS]	1521	91.60%	99.60%
meC0280	[locus_tag=DQM41_RS03630]	lcl|NZ_LS483362.1_cds_WP_002852367.1_704	346	984	639	reverse	346	984	639	346	984	[gene=corA] [protein=magnesium/cobalt transporter CorA] [protein_id=WP_002852367.1] [location=complement(680358..681341)] [gbkey=CDS]	984	89.70%	63.90%
meC0467	[locus_tag=DQM41_RS03630]	lcl|NZ_LS483362.1_cds_WP_002852367.1_704	1	486	486	forward	1	486	486	1	486	[gene=corA] [protein=magnesium/cobalt transporter CorA] [protein_id=WP_002852367.1] [location=complement(680358..681341)] [gbkey=CDS]	984	99.40%	48.60%
meC0280	[locus_tag=DQM41_RS06935]	lcl|NZ_LS483362.1_cds_WP_002865443.1_1344	1	822	822	forward	1	822	822	1	822	[gene=feoB] [protein=ferrous iron transport protein B] [protein_id=WP_002865443.1] [location=1331293..1333134] [gbkey=CDS]	1842	94.00%	82.20%
meC0280	[locus_tag=DQM41_RS06935]	lcl|NZ_LS483362.1_cds_WP_002865443.1_1344	823	1821	999	forward	823	1821	999	823	1821	[gene=feoB] [protein=ferrous iron transport protein B] [protein_id=WP_002865443.1] [location=1331293..1333134] [gbkey=CDS]	1842	96.20%	99.90%
meC0467	[locus_tag=DQM41_RS06935]	lcl|NZ_LS483362.1_cds_WP_002865443.1_1344	658	1161	504	reverse	658	1161	504	658	1161	[gene=feoB] [protein=ferrous iron transport protein B] [protein_id=WP_002865443.1] [location=1331293..1333134] [gbkey=CDS]	1842	90.80%	50.40%
meC0467	[locus_tag=DQM41_RS06935]	lcl|NZ_LS483362.1_cds_WP_002865443.1_1344	1	654	654	reverse	1	654	654	1	654	[gene=feoB] [protein=ferrous iron transport protein B] [protein_id=WP_002865443.1] [location=1331293..1333134] [gbkey=CDS]	1842	93.80%	65.40%
meC0280	[locus_tag=DQM41_RS06465]	lcl|NZ_LS483362.1_cds_WP_002786080.1_1252	784	1563	780	forward	784	1563	780	784	1563	[protein=HAD-IIIC family phosphatase] [protein_id=WP_002786080.1] [location=1231427..1232989] [gbkey=CDS]	1563	98.50%	78.00%
meC0467	[locus_tag=DQM41_RS06465]	lcl|NZ_LS483362.1_cds_WP_002786080.1_1252	742	1563	822	reverse	742	1563	822	742	1563	[protein=HAD-IIIC family phosphatase] [protein_id=WP_002786080.1] [location=1231427..1232989] [gbkey=CDS]	1563	99.60%	82.20%
meC0467	[locus_tag=DQM41_RS06465]	lcl|NZ_LS483362.1_cds_WP_002786080.1_1252	1	525	525	reverse	1	525	525	1	525	[protein=HAD-IIIC family phosphatase] [protein_id=WP_002786080.1] [location=1231427..1232989] [gbkey=CDS]	1563	99.40%	52.50%
meC0280	[locus_tag=DQM41_RS00240]	lcl|NZ_LS483362.1_cds_WP_010891832.1_43	607	1440	834	reverse	607	1440	834	607	1440	[protein=DUF342 domain-containing protein] [protein_id=WP_010891832.1] [location=complement(63868..65739)] [gbkey=CDS]	1872	91.10%	83.40%
meC0280	[locus_tag=DQM41_RS00240]	lcl|NZ_LS483362.1_cds_WP_010891832.1_43	1	606	606	reverse	1	606	606	1	606	[protein=DUF342 domain-containing protein] [protein_id=WP_010891832.1] [location=complement(63868..65739)] [gbkey=CDS]	1872	100.00%	60.60%
meC0467	[locus_tag=DQM41_RS00240]	lcl|NZ_LS483362.1_cds_WP_010891832.1_43	1	786	786	reverse	1	786	786	1	786	[protein=DUF342 domain-containing protein] [protein_id=WP_010891832.1] [location=complement(63868..65739)] [gbkey=CDS]	1872	98.90%	78.60%
meC0280	[locus_tag=DQM41_RS01430]	lcl|NZ_LS483362.1_cds_WP_002778123.1_278	447	848	402	reverse	447	848	402	447	848	[protein=pantoate--beta-alanine ligase] [protein_id=WP_002778123.1] [location=complement(271412..272260)] [gbkey=CDS]	849	99.30%	40.20%
meC0467	[locus_tag=DQM41_RS01430]	lcl|NZ_LS483362.1_cds_WP_002778123.1_278	3	728	726	forward	3	728	726	3	728	[protein=pantoate--beta-alanine ligase] [protein_id=WP_002778123.1] [location=complement(271412..272260)] [gbkey=CDS]	849	99.20%	72.60%
meC0280	[locus_tag=DQM41_RS04280]	lcl|NZ_LS483362.1_cds_WP_002864843.1_829	1441	1509	69	reverse	1441	1509	69	1441	1509	[protein=chorismate-binding protein] [protein_id=WP_002864843.1] [location=complement(807480..809264)] [gbkey=CDS]	1785	47.80%	6.90%
meC0467	[locus_tag=DQM41_RS04280]	lcl|NZ_LS483362.1_cds_WP_002864843.1_829	1441	1509	69	forward	1441	1509	69	1441	1509	[protein=chorismate-binding protein] [protein_id=WP_002864843.1] [location=complement(807480..809264)] [gbkey=CDS]	1785	47.80%	6.90%
meC0280	[locus_tag=DQM41_RS03095]	lcl|NZ_LS483362.1_cds_WP_002858500.1_597	154	648	495	forward	154	648	495	154	648	[protein=M48 family metallopeptidase] [protein_id=WP_002858500.1] [location=579627..580274] [gbkey=CDS]	648	97.60%	49.50%
meC0467	[locus_tag=DQM41_RS03095]	lcl|NZ_LS483362.1_cds_WP_002858500.1_597	31	648	618	reverse	31	648	618	31	648	[protein=M48 family metallopeptidase] [protein_id=WP_002858500.1] [location=579627..580274] [gbkey=CDS]	648	95.60%	61.80%
meC0280	[locus_tag=DQM41_RS03640]	lcl|NZ_LS483362.1_cds_WP_002793291.1_706	484	1326	843	forward	484	1326	843	484	1326	[protein=esterase-like activity of phytase family protein] [protein_id=WP_002793291.1] [location=682515..683840] [gbkey=CDS]	1326	98.60%	84.30%
meC0467	[locus_tag=DQM41_RS03640]	lcl|NZ_LS483362.1_cds_WP_002793291.1_706	343	1326	984	reverse	343	1326	984	343	1326	[protein=esterase-like activity of phytase family protein] [protein_id=WP_002793291.1] [location=682515..683840] [gbkey=CDS]	1326	99.40%	98.40%
meC0280	[locus_tag=DQM41_RS05220]	lcl|NZ_LS483362.1_cds_WP_002864285.1_1006	1903	2628	726	reverse	1903	2628	726	1903	2628	[protein=N-6 DNA methylase] [protein_id=WP_002864285.1] [location=complement(982973..986992)] [gbkey=CDS]	4020	94.20%	72.60%
meC0280	[locus_tag=DQM41_RS05220]	lcl|NZ_LS483362.1_cds_WP_002864285.1_1006	901	1899	999	reverse	901	1899	999	901	1899	[protein=N-6 DNA methylase] [protein_id=WP_002864285.1] [location=complement(982973..986992)] [gbkey=CDS]	4020	100.00%	99.90%
meC0280	[locus_tag=DQM41_RS05220]	lcl|NZ_LS483362.1_cds_WP_002864285.1_1006	1	900	900	reverse	1	900	900	1	900	[protein=N-6 DNA methylase] [protein_id=WP_002864285.1] [location=complement(982973..986992)] [gbkey=CDS]	4020	99.00%	90.00%
meC0467	[locus_tag=DQM41_RS05220]	lcl|NZ_LS483362.1_cds_WP_002864285.1_1006	2359	3354	996	reverse	2359	3354	996	2359	3354	[protein=N-6 DNA methylase] [protein_id=WP_002864285.1] [location=complement(982973..986992)] [gbkey=CDS]	4020	100.00%	99.90%
meC0467	[locus_tag=DQM41_RS05220]	lcl|NZ_LS483362.1_cds_WP_002864285.1_1006	1357	2355	999	reverse	1357	2355	999	1357	2355	[protein=N-6 DNA methylase] [protein_id=WP_002864285.1] [location=complement(982973..986992)] [gbkey=CDS]	4020	98.80%	99.90%
meC0467	[locus_tag=DQM41_RS05220]	lcl|NZ_LS483362.1_cds_WP_002864285.1_1006	358	1356	999	reverse	358	1356	999	358	1356	[protein=N-6 DNA methylase] [protein_id=WP_002864285.1] [location=complement(982973..986992)] [gbkey=CDS]	4020	98.50%	99.60%
meC0280	[locus_tag=DQM41_RS05910]	lcl|NZ_LS483362.1_cds_WP_002852850.1_1144	46	519	474	forward	46	519	474	46	519	[protein=cation:dicarboxylase symporter family transporter] [protein_id=WP_002852850.1] [location=1119951..1121327] [gbkey=CDS]	1377	98.70%	47.40%
meC0467	[locus_tag=DQM41_RS05910]	lcl|NZ_LS483362.1_cds_WP_002852850.1_1144	370	1365	996	reverse	370	1365	996	370	1365	[protein=cation:dicarboxylase symporter family transporter] [protein_id=WP_002852850.1] [location=1119951..1121327] [gbkey=CDS]	1377	95.70%	99.60%
meC0280	[locus_tag=DQM41_RS06505]	lcl|NZ_LS483362.1_cds_WP_002856137.1_1260	586	1068	483	reverse	586	1068	483	586	1068	[protein=hypothetical protein] [protein_id=WP_002856137.1] [location=complement(1238565..1239635)] [gbkey=CDS]	1071	100.00%	48.30%
meC0467	[locus_tag=DQM41_RS06505]	lcl|NZ_LS483362.1_cds_WP_002856137.1_1260	280	849	570	forward	280	849	570	280	849	[protein=hypothetical protein] [protein_id=WP_002856137.1] [location=complement(1238565..1239635)] [gbkey=CDS]	1071	98.90%	57.00%
meC0280	[locus_tag=DQM41_RS08575]	lcl|NZ_LS483362.1_cds_WP_002882660.1_1658	2020	2598	579	reverse	2020	2598	579	2020	2598	[gene=flgE] [protein=flagellar hook protein FlgE] [protein_id=WP_002882660.1] [location=complement(1638087..1640684)] [gbkey=CDS]	2598	98.40%	57.90%
meC0467	[locus_tag=DQM41_RS08575]	lcl|NZ_LS483362.1_cds_WP_002882660.1_1658	340	804	465	reverse	340	804	465	340	804	[gene=flgE] [protein=flagellar hook protein FlgE] [protein_id=WP_002882660.1] [location=complement(1638087..1640684)] [gbkey=CDS]	2598	96.80%	46.50%
meC0280	[locus_tag=DQM41_RS01885]	lcl|NZ_LS483362.1_cds_WP_002858719.1_369	229	960	732	forward	229	960	732	229	960	[gene=trpS] [protein=tryptophan--tRNA ligase] [protein_id=WP_002858719.1] [location=353302..354261] [gbkey=CDS]	960	91.00%	73.20%
meC0467	[locus_tag=DQM41_RS01885]	lcl|NZ_LS483362.1_cds_WP_002858719.1_369	1	690	690	reverse	1	690	690	1	690	[gene=trpS] [protein=tryptophan--tRNA ligase] [protein_id=WP_002858719.1] [location=353302..354261] [gbkey=CDS]	960	93.90%	69.00%
meC0280	[locus_tag=DQM41_RS02410]	lcl|NZ_LS483362.1_cds_WP_002864169.1_464	1	759	759	forward	1	759	759	1	759	[protein=MFS transporter] [protein_id=WP_002864169.1] [location=451040..452272] [gbkey=CDS]	1233	99.60%	75.90%
meC0280	[locus_tag=DQM41_RS02410]	lcl|NZ_LS483362.1_cds_WP_002864169.1_464	762	1229	468	forward	762	1229	468	762	1229	[protein=MFS transporter] [protein_id=WP_002864169.1] [location=451040..452272] [gbkey=CDS]	1233	98.70%	46.80%
meC0467	[locus_tag=DQM41_RS02410]	lcl|NZ_LS483362.1_cds_WP_002864169.1_464	570	1229	660	reverse	570	1229	660	570	1229	[protein=MFS transporter] [protein_id=WP_002864169.1] [location=451040..452272] [gbkey=CDS]	1233	80.70%	66.00%
meC0280	[locus_tag=DQM41_RS03070]	lcl|NZ_LS483362.1_cds_WP_002776292.1_592	3	560	558	forward	3	560	558	3	560	[gene=pstC] [protein=phosphate ABC transporter permease subunit PstC] [protein_id=WP_002776292.1] [location=574220..575134] [gbkey=CDS]	915	99.50%	55.80%
meC0467	[locus_tag=DQM41_RS03070]	lcl|NZ_LS483362.1_cds_WP_002776292.1_592	3	575	573	reverse	3	575	573	3	575	[gene=pstC] [protein=phosphate ABC transporter permease subunit PstC] [protein_id=WP_002776292.1] [location=574220..575134] [gbkey=CDS]	915	99.00%	57.30%
meC0280	[locus_tag=DQM41_RS06565]	lcl|NZ_LS483362.1_cds_WP_002786732.1_1272	43	969	927	forward	43	969	927	43	969	[gene=legB] [protein=4,6-dehydratase LegB] [protein_id=WP_002786732.1] [location=1248606..1249577] [gbkey=CDS]	972	99.00%	92.70%
meC0467	[locus_tag=DQM41_RS06565]	lcl|NZ_LS483362.1_cds_WP_002786732.1_1272	1	810	810	reverse	1	810	810	1	810	[gene=legB] [protein=4,6-dehydratase LegB] [protein_id=WP_002786732.1] [location=1248606..1249577] [gbkey=CDS]	972	99.60%	81.00%
meC0280	[locus_tag=DQM41_RS05800]	lcl|NZ_LS483362.1_cds_WP_010891915.1_1122	133	648	516	reverse	133	648	516	133	648	[gene=omp50] [protein=outer membrane tyrosine kinase] [protein_id=WP_010891915.1] [location=complement(1098598..1100019)] [gbkey=CDS]	1422	98.80%	51.60%
meC0467	[locus_tag=DQM41_RS05800]	lcl|NZ_LS483362.1_cds_WP_010891915.1_1122	1093	1422	330	forward	1093	1422	330	1093	1422	[gene=omp50] [protein=outer membrane tyrosine kinase] [protein_id=WP_010891915.1] [location=complement(1098598..1100019)] [gbkey=CDS]	1422	100.00%	33.00%
meC0280	[locus_tag=DQM41_RS07490]	lcl|NZ_LS483362.1_cds_WP_010891941.1_1454	1	717	717	reverse	1	717	717	1	717	[protein=formate dehydrogenase subunit alpha] [transl_except=(pos:541..543,aa:Sec)] [protein_id=WP_010891941.1] [location=complement(1446324..1449128)] [gbkey=CDS]	2805	91.00%	71.70%
meC0467	[locus_tag=DQM41_RS07490]	lcl|NZ_LS483362.1_cds_WP_010891941.1_1454	1471	2157	687	forward	1471	2157	687	1471	2157	[protein=formate dehydrogenase subunit alpha] [transl_except=(pos:541..543,aa:Sec)] [protein_id=WP_010891941.1] [location=complement(1446324..1449128)] [gbkey=CDS]	2805	95.20%	68.70%
meC0280	[locus_tag=DQM41_RS07710]	lcl|NZ_LS483362.1_cds_WP_010891944.1_1498	928	1491	564	reverse	928	1491	564	928	1491	[protein=methyl-accepting chemotaxis protein] [protein_id=WP_010891944.1] [location=1491986..1493974] [gbkey=CDS]	1989	70.20%	56.40%
meC0467	[locus_tag=DQM41_RS07710]	lcl|NZ_LS483362.1_cds_WP_010891944.1_1498	42	608	567	reverse	42	608	567	42	608	[protein=methyl-accepting chemotaxis protein] [protein_id=WP_010891944.1] [location=1491986..1493974] [gbkey=CDS]	1989	98.90%	56.70%
meC0280	[locus_tag=DQM41_RS02380]	lcl|NZ_LS483362.1_cds_WP_010891860.1_458	1810	2808	999	forward	1810	2808	999	1810	2808	[gene=rpoB] [protein=DNA-directed RNA polymerase subunit beta] [protein_id=WP_010891860.1] [location=438986..443122] [gbkey=CDS]	4137	99.70%	99.90%
meC0467	[locus_tag=DQM41_RS02380]	lcl|NZ_LS483362.1_cds_WP_010891860.1_458	3616	4137	522	reverse	3616	4137	522	3616	4137	[gene=rpoB] [protein=DNA-directed RNA polymerase subunit beta] [protein_id=WP_010891860.1] [location=438986..443122] [gbkey=CDS]	4137	97.70%	52.20%
meC0467	[locus_tag=DQM41_RS02380]	lcl|NZ_LS483362.1_cds_WP_010891860.1_458	1615	2613	999	reverse	1615	2613	999	1615	2613	[gene=rpoB] [protein=DNA-directed RNA polymerase subunit beta] [protein_id=WP_010891860.1] [location=438986..443122] [gbkey=CDS]	4137	99.40%	99.90%
meC0280	[locus_tag=DQM41_RS01770]	lcl|NZ_LS483362.1_cds_WP_002872052.1_346	1080	2063	984	forward	1080	2063	984	1080	2063	[gene=cmeB] [protein=multidrug efflux RND transporter permease subunit CmeB] [protein_id=WP_002872052.1] [location=complement(332589..335711)] [gbkey=CDS]	3123	92.90%	98.40%
meC0467	[locus_tag=DQM41_RS01770]	lcl|NZ_LS483362.1_cds_WP_002872052.1_346	321	614	294	forward	321	614	294	321	614	[gene=cmeB] [protein=multidrug efflux RND transporter permease subunit CmeB] [protein_id=WP_002872052.1] [location=complement(332589..335711)] [gbkey=CDS]	3123	91.80%	29.40%
meC0280	[locus_tag=DQM41_RS02440]	lcl|NZ_LS483362.1_cds_WP_002782934.1_470	76	387	312	forward	76	387	312	76	387	[protein=30S ribosomal protein S12] [protein_id=WP_002782934.1] [location=457221..457607] [gbkey=CDS]	387	97.10%	31.20%
meC0467	[locus_tag=DQM41_RS02440]	lcl|NZ_LS483362.1_cds_WP_002782934.1_470	76	387	312	reverse	76	387	312	76	387	[protein=30S ribosomal protein S12] [protein_id=WP_002782934.1] [location=457221..457607] [gbkey=CDS]	387	99.00%	31.20%
meC0280	[locus_tag=DQM41_RS02435]	lcl|NZ_LS483362.1_cds_469	333	1331	999	forward	333	1331	999	333	1331	[gene=aldA] [protein=aldehyde dehydrogenase] [pseudo=true] [location=455455..456893] [gbkey=CDS]	1439	99.10%	99.90%
meC0467	[locus_tag=DQM41_RS02435]	lcl|NZ_LS483362.1_cds_469	189	1136	948	reverse	189	1136	948	189	1136	[gene=aldA] [protein=aldehyde dehydrogenase] [pseudo=true] [location=455455..456893] [gbkey=CDS]	1439	96.80%	94.80%
meC0280	[locus_tag=DQM41_RS08455]	lcl|NZ_LS483362.1_cds_WP_002864792.1_1638	1	549	549	forward	1	549	549	1	549	[protein=50S ribosomal protein L3] [protein_id=WP_002864792.1] [location=complement(1619787..1620362)] [gbkey=CDS]	576	99.50%	54.90%
meC0467	[locus_tag=DQM41_RS08455]	lcl|NZ_LS483362.1_cds_WP_002864792.1_1638	1	576	576	reverse	1	576	576	1	576	[protein=50S ribosomal protein L3] [protein_id=WP_002864792.1] [location=complement(1619787..1620362)] [gbkey=CDS]	576	99.50%	57.60%
meC0280	[locus_tag=DQM41_RS02720]	lcl|NZ_LS483362.1_cds_WP_002864504.1_525	43	690	648	forward	43	690	648	43	690	[gene=fliD] [protein=flagellar filament capping protein FliD] [protein_id=WP_002864504.1] [location=510546..512474] [gbkey=CDS]	1929	93.10%	64.80%
meC0467	[locus_tag=DQM41_RS02720]	lcl|NZ_LS483362.1_cds_WP_002864504.1_525	43	900	858	reverse	43	900	858	43	900	[gene=fliD] [protein=flagellar filament capping protein FliD] [protein_id=WP_002864504.1] [location=510546..512474] [gbkey=CDS]	1929	99.00%	85.80%
meC0280	[locus_tag=DQM41_RS00110]	lcl|NZ_LS483362.1_cds_WP_010891825.1_22	16	717	702	reverse	16	717	702	16	717	[protein=RluA family pseudouridine synthase] [protein_id=WP_010891825.1] [location=complement(27402..28304)] [gbkey=CDS]	903	86.30%	70.20%
meC0467	[locus_tag=DQM41_RS00110]	lcl|NZ_LS483362.1_cds_WP_010891825.1_22	31	717	687	forward	31	717	687	31	717	[protein=RluA family pseudouridine synthase] [protein_id=WP_010891825.1] [location=complement(27402..28304)] [gbkey=CDS]	903	86.00%	68.70%
meC0280	[locus_tag=DQM41_RS05225]	lcl|NZ_LS483362.1_cds_WP_010891903.1_1007	121	1119	999	reverse	121	1119	999	121	1119	[protein=endonuclease MutS2] [protein_id=WP_010891903.1] [location=complement(987001..989211)] [gbkey=CDS]	2211	99.40%	99.90%
meC0467	[locus_tag=DQM41_RS05225]	lcl|NZ_LS483362.1_cds_WP_010891903.1_1007	577	1500	924	forward	577	1500	924	577	1500	[protein=endonuclease MutS2] [protein_id=WP_010891903.1] [location=complement(987001..989211)] [gbkey=CDS]	2211	99.40%	92.40%
meC0280	[locus_tag=DQM41_RS05130]	lcl|NZ_LS483362.1_cds_WP_010891901.1_988	781	1779	999	reverse	781	1779	999	781	1779	[gene=cmeF] [protein=multidrug efflux RND transporter permease subunit CmeF] [protein_id=WP_010891901.1] [location=965475..968492] [gbkey=CDS]	3018	91.40%	99.90%
meC0280	[locus_tag=DQM41_RS05130]	lcl|NZ_LS483362.1_cds_WP_010891901.1_988	1780	2778	999	reverse	1780	2778	999	1780	2778	[gene=cmeF] [protein=multidrug efflux RND transporter permease subunit CmeF] [protein_id=WP_010891901.1] [location=965475..968492] [gbkey=CDS]	3018	96.30%	99.90%
meC0467	[locus_tag=DQM41_RS05130]	lcl|NZ_LS483362.1_cds_WP_010891901.1_988	1237	2232	996	reverse	1237	2232	996	1237	2232	[gene=cmeF] [protein=multidrug efflux RND transporter permease subunit CmeF] [protein_id=WP_010891901.1] [location=965475..968492] [gbkey=CDS]	3018	99.40%	99.60%
meC0467	[locus_tag=DQM41_RS05130]	lcl|NZ_LS483362.1_cds_WP_010891901.1_988	235	1173	939	reverse	235	1173	939	235	1173	[gene=cmeF] [protein=multidrug efflux RND transporter permease subunit CmeF] [protein_id=WP_010891901.1] [location=965475..968492] [gbkey=CDS]	3018	96.20%	93.90%
meC0280	[locus_tag=DQM41_RS05125]	lcl|NZ_LS483362.1_cds_WP_002858463.1_987	1	522	522	forward	1	522	522	1	522	[gene=cmeE] [protein=multidrug efflux RND transporter periplasmic adaptor subunit CmeE] [protein_id=WP_002858463.1] [location=964732..965472] [gbkey=CDS]	741	92.30%	52.20%
meC0467	[locus_tag=DQM41_RS05125]	lcl|NZ_LS483362.1_cds_WP_002858463.1_987	1	741	741	forward	1	741	741	1	741	[gene=cmeE] [protein=multidrug efflux RND transporter periplasmic adaptor subunit CmeE] [protein_id=WP_002858463.1] [location=964732..965472] [gbkey=CDS]	741	98.40%	74.10%
meC0280	[locus_tag=DQM41_RS07425]	lcl|NZ_LS483362.1_cds_WP_002851458.1_1442	706	1239	534	reverse	706	1239	534	706	1239	[protein=adenylosuccinate synthase] [protein_id=WP_002851458.1] [location=complement(1431075..1432325)] [gbkey=CDS]	1251	97.80%	53.40%
meC0467	[locus_tag=DQM41_RS07425]	lcl|NZ_LS483362.1_cds_WP_002851458.1_1442	148	1146	999	forward	148	1146	999	148	1146	[protein=adenylosuccinate synthase] [protein_id=WP_002851458.1] [location=complement(1431075..1432325)] [gbkey=CDS]	1251	99.70%	99.90%
meC0280	[locus_tag=DQM41_RS05115]	lcl|NZ_LS483362.1_cds_WP_002837927.1_985	235	999	765	reverse	235	999	765	235	999	[gene=lepA] [protein=elongation factor 4] [protein_id=WP_002837927.1] [location=complement(961538..963334)] [gbkey=CDS]	1797	97.30%	76.50%
meC0467	[locus_tag=DQM41_RS05115]	lcl|NZ_LS483362.1_cds_WP_002837927.1_985	1	627	627	forward	1	627	627	1	627	[gene=lepA] [protein=elongation factor 4] [protein_id=WP_002837927.1] [location=complement(961538..963334)] [gbkey=CDS]	1797	94.50%	62.70%
meC0280	[locus_tag=DQM41_RS00120]	lcl|NZ_LS483362.1_cds_WP_002865087.1_24	1	564	564	forward	1	564	564	1	564	[protein=ribonucleoside-diphosphate reductase subunit alpha] [protein_id=WP_002865087.1] [location=complement(29726..32095)] [gbkey=CDS]	2370	97.90%	56.40%
meC0467	[locus_tag=DQM41_RS00120]	lcl|NZ_LS483362.1_cds_WP_002865087.1_24	1552	2370	819	forward	1552	2370	819	1552	2370	[protein=ribonucleoside-diphosphate reductase subunit alpha] [protein_id=WP_002865087.1] [location=complement(29726..32095)] [gbkey=CDS]	2370	97.80%	54.90%
meC0467	[locus_tag=DQM41_RS00120]	lcl|NZ_LS483362.1_cds_WP_002865087.1_24	1	549	549	forward	1	549	549	1	549	[protein=ribonucleoside-diphosphate reductase subunit alpha] [protein_id=WP_002865087.1] [location=complement(29726..32095)] [gbkey=CDS]	2370	94.90%	81.90%
meC0280	[locus_tag=DQM41_RS03080]	lcl|NZ_LS483362.1_cds_WP_010891867.1_594	1	564	564	forward	1	564	564	1	564	[protein=phosphate ABC transporter ATP-binding protein] [protein_id=WP_010891867.1] [location=576216..576956] [gbkey=CDS]	741	99.50%	56.40%
meC0467	[locus_tag=DQM41_RS03080]	lcl|NZ_LS483362.1_cds_WP_010891867.1_594	1	579	579	forward	1	579	579	1	579	[protein=phosphate ABC transporter ATP-binding protein] [protein_id=WP_010891867.1] [location=576216..576956] [gbkey=CDS]	741	99.50%	57.90%
meC0280	[locus_tag=DQM41_RS03340]	lcl|NZ_LS483362.1_cds_WP_002865516.1_646	42	545	504	forward	42	545	504	42	545	[protein=anaerobic C4-dicarboxylate transporter] [protein_id=WP_002865516.1] [location=625542..626966] [gbkey=CDS]	1425	82.70%	50.40%
meC0467	[locus_tag=DQM41_RS03340]	lcl|NZ_LS483362.1_cds_WP_002865516.1_646	156	566	411	reverse	156	566	411	156	566	[protein=anaerobic C4-dicarboxylate transporter] [protein_id=WP_002865516.1] [location=625542..626966] [gbkey=CDS]	1425	87.00%	41.10%
meC0280	[locus_tag=DQM41_RS06550]	lcl|NZ_LS483362.1_cds_WP_002870258.1_1269	7	1005	999	forward	7	1005	999	7	1005	[gene=pseI] [protein=pseudaminic acid synthase] [protein_id=WP_002870258.1] [location=1245633..1246664] [gbkey=CDS]	1032	95.80%	99.90%
meC0467	[locus_tag=DQM41_RS06550]	lcl|NZ_LS483362.1_cds_WP_002870258.1_1269	1	840	840	reverse	1	840	840	1	840	[gene=pseI] [protein=pseudaminic acid synthase] [protein_id=WP_002870258.1] [location=1245633..1246664] [gbkey=CDS]	1032	94.10%	84.00%
meC0280	[locus_tag=DQM41_RS03125]	lcl|NZ_LS483362.1_cds_WP_002776344.1_603	304	597	294	forward	304	597	294	304	597	[gene=hypE] [protein=hydrogenase expression/formation protein HypE] [protein_id=WP_002776344.1] [location=586180..587154] [gbkey=CDS]	975	96.90%	29.40%
meC0467	[locus_tag=DQM41_RS03125]	lcl|NZ_LS483362.1_cds_WP_002776344.1_603	480	974	495	forward	480	974	495	480	974	[gene=hypE] [protein=hydrogenase expression/formation protein HypE] [protein_id=WP_002776344.1] [location=586180..587154] [gbkey=CDS]	975	99.20%	49.50%
meC0280	[locus_tag=DQM41_RS02420]	lcl|NZ_LS483362.1_cds_WP_002864166.1_466	3	452	450	forward	3	452	450	3	452	[gene=fucP] [protein=L-fucose:H+ symporter permease] [protein_id=WP_002864166.1] [location=453113..454369] [gbkey=CDS]	1257	90.40%	45.00%
meC0280	[locus_tag=DQM41_RS02420]	lcl|NZ_LS483362.1_cds_WP_002864166.1_466	676	1257	582	forward	676	1257	582	676	1257	[gene=fucP] [protein=L-fucose:H+ symporter permease] [protein_id=WP_002864166.1] [location=453113..454369] [gbkey=CDS]	1257	100.00%	58.20%
meC0467	[locus_tag=DQM41_RS02420]	lcl|NZ_LS483362.1_cds_WP_002864166.1_466	619	1236	618	reverse	619	1236	618	619	1236	[gene=fucP] [protein=L-fucose:H+ symporter permease] [protein_id=WP_002864166.1] [location=453113..454369] [gbkey=CDS]	1257	92.20%	61.80%
meC0280	[locus_tag=DQM41_RS00230]	lcl|NZ_LS483362.1_cds_WP_002805290.1_41	109	774	666	forward	109	774	666	109	774	[protein=flagellar basal body rod modification protein] [protein_id=WP_002805290.1] [location=61339..62223] [gbkey=CDS]	885	88.10%	66.60%
meC0467	[locus_tag=DQM41_RS00230]	lcl|NZ_LS483362.1_cds_WP_002805290.1_41	112	591	480	forward	112	591	480	112	591	[protein=flagellar basal body rod modification protein] [protein_id=WP_002805290.1] [location=61339..62223] [gbkey=CDS]	885	90.30%	48.00%
meC0280	[locus_tag=DQM41_RS03750]	lcl|NZ_LS483362.1_cds_WP_010891883.1_723	1021	2019	999	forward	1021	2019	999	1021	2019	[gene=cfrA][protein=TonB-dependent ferric enterobactin receptor CfrA] [protein_id=WP_010891883.1] [location=705435..707525] [gbkey=CDS]	2091	94.90%	99.90%
meC0467	[locus_tag=DQM41_RS03750]	lcl|NZ_LS483362.1_cds_WP_010891883.1_723	268	1266	999	reverse	268	1266	999	268	1266	[gene=cfrA] [protein=TonB-dependent ferric enterobactin receptor CfrA] [protein_id=WP_010891883.1] [location=705435..707525] [gbkey=CDS]	2091	100.00%	99.90%
meC0280	[locus_tag=DQM41_RS06490]	lcl|NZ_LS483362.1_cds_WP_002858432.1_1257	733	1221	489	reverse	733	1221	489	733	1221	[protein=DUF2920 family protein] [protein_id=WP_002858432.1] [location=complement(1235506..1236732)] [gbkey=CDS]	1227	89.00%	48.90%
meC0467	[locus_tag=DQM41_RS06490]	lcl|NZ_LS483362.1_cds_WP_002858432.1_1257	733	1221	489	reverse	733	1221	489	733	1221	[protein=DUF2920 family protein] [protein_id=WP_002858432.1] [location=complement(1235506..1236732)] [gbkey=CDS]	1227	87.70%	48.90%
meC0280	[locus_tag=DQM41_RS08350]	lcl|NZ_LS483362.1_cds_WP_002851268.1_1617	598	1596	999	reverse	598	1596	999	598	1596	[protein=type I DNA topoisomerase] [protein_id=WP_002851268.1] [location=complement(1607343..1609445)] [gbkey=CDS]	2103	96.10%	99.90%
meC0467	[locus_tag=DQM41_RS08350]	lcl|NZ_LS483362.1_cds_WP_002851268.1_1617	1	912	912	forward	1	912	912	1	912	[protein=type I DNA topoisomerase] [protein_id=WP_002851268.1] [location=complement(1607343..1609445)] [gbkey=CDS]	2103	97.00%	91.20%
meC0280	[locus_tag=DQM41_RS00180]	lcl|NZ_LS483362.1_cds_31	764	1762	999	forward	764	1762	999	764	1762	[protein=class I SAM-dependent DNA methyltransferase] [pseudo=true] [location=46420..50152] [gbkey=CDS]	3733	93.40%	99.90%
meC0280	[locus_tag=DQM41_RS00180]	lcl|NZ_LS483362.1_cds_31	1765	2250	486	forward	1765	2250	486	1765	2250	[protein=class I SAM-dependent DNA methyltransferase] [pseudo=true] [location=46420..50152] [gbkey=CDS]	3733	82.70%	48.60%
meC0467	[locus_tag=DQM41_RS00180]	lcl|NZ_LS483362.1_cds_31	746	1744	999	forward	746	1744	999	746	1744	[protein=class I SAM-dependent DNA methyltransferase] [pseudo=true] [location=46420..50152] [gbkey=CDS]	3733	92.00%	99.90%
meC0280	[locus_tag=DQM41_RS06015]	lcl|NZ_LS483362.1_cds_WP_002852810.1_1165	979	1140	162	reverse	979	1140	162	979	1140	[protein=FAD-binding protein] [protein_id=WP_002852810.1] [location=complement(1141617..1142999)] [gbkey=CDS]	1383	31.50%	16.20%
meC0280	[locus_tag=DQM41_RS06015]	lcl|NZ_LS483362.1_cds_WP_002852810.1_1165	979	1140	162	reverse	979	1140	162	979	1140	[protein=FAD-binding protein] [protein_id=WP_002852810.1] [location=complement(1141617..1142999)] [gbkey=CDS]	1383	31.50%	16.20%
meC0467	[locus_tag=DQM41_RS06015]	lcl|NZ_LS483362.1_cds_WP_002852810.1_1165	979	1140	162	forward	979	1140	162	979	1140	[protein=FAD-binding protein] [protein_id=WP_002852810.1] [location=complement(1141617..1142999)] [gbkey=CDS]	1383	31.50%	16.20%
meC0280	[locus_tag=DQM41_RS00190]	lcl|NZ_LS483362.1_cds_WP_002866314.1_33	64	690	627	reverse	64	690	627	64	690	[protein=SIMPL domain-containing protein] [protein_id=WP_002866314.1] [location=complement(51963..52664)] [gbkey=CDS]	702	90.40%	62.70%
meC0467	[locus_tag=DQM41_RS00190]	lcl|NZ_LS483362.1_cds_WP_002866314.1_33	64	702	639	reverse	64	702	639	64	702	[protein=SIMPL domain-containing protein] [protein_id=WP_002866314.1] [location=complement(51963..52664)] [gbkey=CDS]	702	90.60%	63.90%
meC0280	[locus_tag=DQM41_RS00615]	lcl|NZ_LS483362.1_cds_WP_002851926.1_117	574	903	330	reverse	574	903	330	574	903	[protein=tRNA-dihydrouridine synthase] [protein_id=WP_002851926.1] [location=complement(125499..126425)] [gbkey=CDS]	927	97.30%	33.00%
meC0467	[locus_tag=DQM41_RS00615]	lcl|NZ_LS483362.1_cds_WP_002851926.1_117	397	903	507	reverse	397	903	507	397	903	[protein=tRNA-dihydrouridine synthase] [protein_id=WP_002851926.1] [location=complement(125499..126425)] [gbkey=CDS]	927	95.30%	50.70%

Table S6: Common genes with putative *C. jejuni* origin of meC0281 and meC0467.

genome	locus_tag	Sequence Name	Minimum	Maximum	Length	Direction	Min (with gaps)	Max (with gaps)	Length (with gaps)	Min (original sequence)	Max (original sequence)	Description	Sequence Length	% Pairwise Identity	Query Coverage
meC0281	[locus_tag=DQM41_RS08470]	lcl|NZ_LS483362.1_cds_WP_002864793.1_1641	457	1455	999	reverse	457	1455	999	457	1455	[protein=ribonuclease J] [protein_id=WP_002864793.1] [location=complement(1621679..1623673)] [gbkey=CDS]	1995	99.70%	99.90%
meC0467	[locus_tag=DQM41_RS08470]	lcl|NZ_LS483362.1_cds_WP_002864793.1_1641	190	1182	993	reverse	190	1182	993	190	1182	[protein=ribonuclease J] [protein_id=WP_002864793.1] [location=complement(1621679..1623673)] [gbkey=CDS]	1995	99.10%	99.30%
meC0281	[locus_tag=DQM41_RS07815]	lcl|NZ_LS483362.1_cds_WP_002851210.1_1519	325	1323	999	forward	325	1323	999	325	1323	[protein=FAD-binding oxidoreductase] [protein_id=WP_002851210.1] [location=complement(1514627..1517398)] [gbkey=CDS]	2772	95.10%	99.90%
meC0467	[locus_tag=DQM41_RS07815]	lcl|NZ_LS483362.1_cds_WP_002851210.1_1519	1	399	399	forward	1	399	399	1	399	[protein=FAD-binding oxidoreductase] [protein_id=WP_002851210.1] [location=complement(1514627..1517398)] [gbkey=CDS]	2772	89.90%	39.90%
meC0281	[locus_tag=DQM41_RS01160]	lcl|NZ_LS483362.1_cds_WP_002851982.1_225	1	582	582	reverse	1	582	582	1	582	[gene=miaA] [protein=tRNA (adenosine(37)-N6)-dimethylallyltransferase MiaA] [protein_id=WP_002851982.1] [location=complement(222479..223348)] [gbkey=CDS]	870	83.50%	58.20%
meC0467	[locus_tag=DQM41_RS01160]	lcl|NZ_LS483362.1_cds_WP_002851982.1_225	1	570	570	reverse	1	570	570	1	570	[gene=miaA] [protein=tRNA (adenosine(37)-N6)-dimethylallyltransferase MiaA] [protein_id=WP_002851982.1] [location=complement(222479..223348)] [gbkey=CDS]	870	83.70%	57.00%
meC0281	[locus_tag=DQM41_RS03100]	lcl|NZ_LS483362.1_cds_WP_002826526.1_598	1	948	948	reverse	1	948	948	1	948	[protein=hypothetical protein] [protein_id=WP_002826526.1] [location=580316..581836] [gbkey=CDS]	1521	98.70%	94.80%
meC0467	[locus_tag=DQM41_RS03100]	lcl|NZ_LS483362.1_cds_WP_002826526.1_598	343	1338	996	reverse	343	1338	996	343	1338	[protein=hypothetical protein] [protein_id=WP_002826526.1] [location=580316..581836] [gbkey=CDS]	1521	91.60%	99.60%
meC0281	[locus_tag=DQM41_RS00235]	lcl|NZ_LS483362.1_cds_WP_002805291.1_42	1072	1638	567	forward	1072	1638	567	1072	1638	[protein=flagellar hook protein FlgE] [protein_id=WP_002805291.1] [location=62227..63864] [gbkey=CDS]	1638	98.90%	56.70%
meC0467	[locus_tag=DQM41_RS00235]	lcl|NZ_LS483362.1_cds_WP_002805291.1_42	727	1638	912	forward	727	1638	912	727	1638	[protein=flagellar hook protein FlgE] [protein_id=WP_002805291.1] [location=62227..63864] [gbkey=CDS]	1638	99.30%	91.20%
meC0281	[locus_tag=DQM41_RS05110]	lcl|NZ_LS483362.1_cds_WP_002852941.1_984	46	645	600	forward	46	645	600	46	645	[protein=hypothetical protein] [protein_id=WP_002852941.1] [location=complement(960817..961461)] [gbkey=CDS]	645	78.00%	60.00%
meC0467	[locus_tag=DQM41_RS05110]	lcl|NZ_LS483362.1_cds_WP_002852941.1_984	46	645	600	forward	46	645	600	46	645	[protein=hypothetical protein] [protein_id=WP_002852941.1] [location=complement(960817..961461)] [gbkey=CDS]	645	78.50%	60.00%
meC0281	[locus_tag=DQM41_RS01430]	lcl|NZ_LS483362.1_cds_WP_002778123.1_278	405	848	444	forward	405	848	444	405	848	[protein=pantoate--beta-alanine ligase] [protein_id=WP_002778123.1] [location=complement(271412..272260)] [gbkey=CDS]	849	99.30%	44.40%
meC0467	[locus_tag=DQM41_RS01430]	lcl|NZ_LS483362.1_cds_WP_002778123.1_278	3	728	726	forward	3	728	726	3	728	[protein=pantoate--beta-alanine ligase] [protein_id=WP_002778123.1] [location=complement(271412..272260)] [gbkey=CDS]	849	99.20%	72.60%
meC0281	[locus_tag=DQM41_RS04280]	lcl|NZ_LS483362.1_cds_WP_002864843.1_829	1441	1509	69	forward	1441	1509	69	1441	1509	[protein=chorismate-binding protein] [protein_id=WP_002864843.1] [location=complement(807480..809264)] [gbkey=CDS]	1785	47.80%	6.90%
meC0281	[locus_tag=DQM41_RS04280]	lcl|NZ_LS483362.1_cds_WP_002864843.1_829	1441	1509	69	reverse	1441	1509	69	1441	1509	[protein=chorismate-binding protein] [protein_id=WP_002864843.1] [location=complement(807480..809264)] [gbkey=CDS]	1785	47.80%	6.90%
meC0281	[locus_tag=DQM41_RS04280]	lcl|NZ_LS483362.1_cds_WP_002864843.1_829	1441	1509	69	reverse	1441	1509	69	1441	1509	[protein=chorismate-binding protein] [protein_id=WP_002864843.1] [location=complement(807480..809264)] [gbkey=CDS]	1785	47.80%	6.90%
meC0467	[locus_tag=DQM41_RS04280]	lcl|NZ_LS483362.1_cds_WP_002864843.1_829	1441	1509	69	forward	1441	1509	69	1441	1509	[protein=chorismate-binding protein] [protein_id=WP_002864843.1] [location=complement(807480..809264)] [gbkey=CDS]	1785	47.80%	6.90%
meC0281	[locus_tag=DQM41_RS03640]	lcl|NZ_LS483362.1_cds_WP_002793291.1_706	1	948	948	reverse	1	948	948	1	948	[protein=esterase-like activity of phytase family protein] [protein_id=WP_002793291.1] [location=682515..683840] [gbkey=CDS]	1326	99.40%	94.80%
meC0467	[locus_tag=DQM41_RS03640]	lcl|NZ_LS483362.1_cds_WP_002793291.1_706	343	1326	984	reverse	343	1326	984	343	1326	[protein=esterase-like activity of phytase family protein] [protein_id=WP_002793291.1] [location=682515..683840] [gbkey=CDS]	1326	99.40%	98.40%
meC0281	[locus_tag=DQM41_RS05220]	lcl|NZ_LS483362.1_cds_WP_002864285.1_1006	466	1464	999	forward	466	1464	999	466	1464	[protein=N-6 DNA methylase] [protein_id=WP_002864285.1] [location=complement(982973..986992)] [gbkey=CDS]	4020	99.70%	99.90%
meC0281	[locus_tag=DQM41_RS05220]	lcl|NZ_LS483362.1_cds_WP_002864285.1_1006	1465	2463	999	forward	1465	2463	999	1465	2463	[protein=N-6 DNA methylase] [protein_id=WP_002864285.1] [location=complement(982973..986992)] [gbkey=CDS]	4020	98.20%	99.90%
meC0281	[locus_tag=DQM41_RS05220]	lcl|NZ_LS483362.1_cds_WP_002864285.1_1006	3391	4020	630	forward	3391	4020	630	3391	4020	[protein=N-6 DNA methylase] [protein_id=WP_002864285.1] [location=complement(982973..986992)] [gbkey=CDS]	4020	93.60%	63.00%
meC0467	[locus_tag=DQM41_RS05220]	lcl|NZ_LS483362.1_cds_WP_002864285.1_1006	2359	3354	996	forward	2359	3354	996	2359	3354	[protein=N-6 DNA methylase] [protein_id=WP_002864285.1] [location=complement(982973..986992)] [gbkey=CDS]	4020	100.00%	99.90%
meC0467	[locus_tag=DQM41_RS05220]	lcl|NZ_LS483362.1_cds_WP_002864285.1_1006	1357	2355	999	forward	1357	2355	999	1357	2355	[protein=N-6 DNA methylase] [protein_id=WP_002864285.1] [location=complement(982973..986992)] [gbkey=CDS]	4020	98.80%	99.90%
meC0467	[locus_tag=DQM41_RS05220]	lcl|NZ_LS483362.1_cds_WP_002864285.1_1006	358	1356	999	forward	358	1356	999	358	1356	[protein=N-6 DNA methylase] [protein_id=WP_002864285.1] [location=complement(982973..986992)] [gbkey=CDS]	4020	98.50%	99.60%
meC0281	[locus_tag=DQM41_RS07470]	lcl|NZ_LS483362.1_cds_WP_002822570.1_1450	1	495	495	forward	1	495	495	1	495	[protein=LysR family transcriptional regulator] [protein_id=WP_002822570.1] [location=complement(1443078..1443815)] [gbkey=CDS]	738	100.00%	49.50%
meC0467	[locus_tag=DQM41_RS07470]	lcl|NZ_LS483362.1_cds_WP_002822570.1_1450	1	738	738	forward	1	738	738	1	738	[protein=LysR family transcriptional regulator] [protein_id=WP_002822570.1] [location=complement(1443078..1443815)] [gbkey=CDS]	738	100.00%	73.80%
meC0281	[locus_tag=DQM41_RS05910]	lcl|NZ_LS483362.1_cds_WP_002852850.1_1144	817	1377	561	reverse	817	1377	561	817	1377	[protein=cation:dicarboxylase symporter family transporter] [protein_id=WP_002852850.1] [location=1119951..1121327] [gbkey=CDS]	1377	100.00%	56.10%
meC0281	[locus_tag=DQM41_RS05910]	lcl|NZ_LS483362.1_cds_WP_002852850.1_1144	46	813	768	reverse	46	813	768	46	813	[protein=cation:dicarboxylase symporter family transporter] [protein_id=WP_002852850.1] [location=1119951..1121327] [gbkey=CDS]	1377	94.70%	76.80%
meC0467	[locus_tag=DQM41_RS05910]	lcl|NZ_LS483362.1_cds_WP_002852850.1_1144	370	1365	996	reverse	370	1365	996	370	1365	[protein=cation:dicarboxylase symporter family transporter] [protein_id=WP_002852850.1] [location=1119951..1121327] [gbkey=CDS]	1377	95.70%	99.60%
meC0281	[locus_tag=DQM41_RS02710]	lcl|NZ_LS483362.1_cds_WP_002865770.1_523	741	1736	996	reverse	741	1736	996	741	1736	[protein=menaquinone biosynthesis decarboxylase] [protein_id=WP_002865770.1] [location=508218..510020] [gbkey=CDS]	1803	99.10%	99.60%
meC0467	[locus_tag=DQM41_RS02710]	lcl|NZ_LS483362.1_cds_WP_002865770.1_523	1230	1802	573	reverse	1230	1802	573	1230	1802	[protein=menaquinone biosynthesis decarboxylase] [protein_id=WP_002865770.1] [location=508218..510020] [gbkey=CDS]	1803	99.50%	57.30%
meC0467	[locus_tag=DQM41_RS02710]	lcl|NZ_LS483362.1_cds_WP_002865770.1_523	229	1227	999	reverse	229	1227	999	229	1227	[protein=menaquinone biosynthesis decarboxylase] [protein_id=WP_002865770.1] [location=508218..510020] [gbkey=CDS]	1803	99.70%	99.90%
meC0281	[locus_tag=DQM41_RS02405]	lcl|NZ_LS483362.1_cds_WP_025096279.1_463	262	1167	906	reverse	262	1167	906	262	1167	[protein=UxaA family hydrolase] [protein_id=WP_025096279.1] [location=449862..451028] [gbkey=CDS]	1167	96.70%	90.60%
meC0467	[locus_tag=DQM41_RS02405]	lcl|NZ_LS483362.1_cds_WP_025096279.1_463	1	744	744	reverse	1	744	744	1	744	[protein=UxaA family hydrolase] [protein_id=WP_025096279.1] [location=449862..451028] [gbkey=CDS]	1167	96.80%	74.40%
meC0281	[locus_tag=DQM41_RS07550]	lcl|NZ_LS483362.1_cds_1466	654	1172	519	reverse	654	1172	519	654	1172	[protein=TRAP transporter large permease subunit] [pseudo=true] [location=1460273..1461466] [gbkey=CDS]	1194	93.60%	51.90%
meC0467	[locus_tag=DQM41_RS07550]	lcl|NZ_LS483362.1_cds_1466	654	1076	423	reverse	654	1076	423	654	1076	[protein=TRAP transporter large permease subunit] [pseudo=true] [location=1460273..1461466] [gbkey=CDS]	1194	88.70%	42.30%
meC0281	[locus_tag=DQM41_RS06505]	lcl|NZ_LS483362.1_cds_WP_002856137.1_1260	280	936	657	forward	280	936	657	280	936	[protein=hypothetical protein] [protein_id=WP_002856137.1] [location=complement(1238565..1239635)] [gbkey=CDS]	1071	99.10%	65.70%
meC0467	[locus_tag=DQM41_RS06505]	lcl|NZ_LS483362.1_cds_WP_002856137.1_1260	280	849	570	forward	280	849	570	280	849	[protein=hypothetical protein] [protein_id=WP_002856137.1] [location=complement(1238565..1239635)] [gbkey=CDS]	1071	98.90%	57.00%
meC0281	[locus_tag=DQM41_RS07490]	lcl|NZ_LS483362.1_cds_WP_010891941.1_1454	847	1422	576	forward	847	1422	576	847	1422	[protein=formate dehydrogenase subunit alpha] [transl_except=(pos:541..543,aa:Sec)] [protein_id=WP_010891941.1] [location=complement(1446324..1449128)] [gbkey=CDS]	2805	97.40%	57.60%
meC0467	[locus_tag=DQM41_RS07490]	lcl|NZ_LS483362.1_cds_WP_010891941.1_1454	1471	2157	687	forward	1471	2157	687	1471	2157	[protein=formate dehydrogenase subunit alpha] [transl_except=(pos:541..543,aa:Sec)] [protein_id=WP_010891941.1] [location=complement(1446324..1449128)] [gbkey=CDS]	2805	95.20%	68.70%
meC0281	[locus_tag=DQM41_RS02380]	lcl|NZ_LS483362.1_cds_WP_010891860.1_458	2131	3129	999	reverse	2131	3129	999	2131	3129	[gene=rpoB] [protein=DNA-directed RNA polymerase subunit beta] [protein_id=WP_010891860.1] [location=438986..443122] [gbkey=CDS]	4137	99.70%	99.90%
meC0281	[locus_tag=DQM41_RS02380]	lcl|NZ_LS483362.1_cds_WP_010891860.1_458	1132	2127	996	reverse	1132	2127	996	1132	2127	[gene=rpoB] [protein=DNA-directed RNA polymerase subunit beta] [protein_id=WP_010891860.1] [location=438986..443122] [gbkey=CDS]	4137	98.00%	99.60%
meC0467	[locus_tag=DQM41_RS02380]	lcl|NZ_LS483362.1_cds_WP_010891860.1_458	3616	4137	522	reverse	3616	4137	522	3616	4137	[gene=rpoB] [protein=DNA-directed RNA polymerase subunit beta] [protein_id=WP_010891860.1] [location=438986..443122] [gbkey=CDS]	4137	97.70%	52.20%
meC0467	[locus_tag=DQM41_RS02380]	lcl|NZ_LS483362.1_cds_WP_010891860.1_458	1615	2613	999	reverse	1615	2613	999	1615	2613	[gene=rpoB] [protein=DNA-directed RNA polymerase subunit beta] [protein_id=WP_010891860.1] [location=438986..443122] [gbkey=CDS]	4137	99.40%	99.90%
meC0281	[locus_tag=DQM41_RS01410]	lcl|NZ_LS483362.1_cds_WP_002854555.1_274	1	777	777	reverse	1	777	777	1	777	[protein=5'/3'-nucleotidase SurE] [protein_id=WP_002854555.1] [location=269058..269834] [gbkey=CDS]	777	88.70%	77.70%
meC0467	[locus_tag=DQM41_RS01410]	lcl|NZ_LS483362.1_cds_WP_002854555.1_274	1	474	474	reverse	1	474	474	1	474	[protein=5'/3'-nucleotidase SurE] [protein_id=WP_002854555.1] [location=269058..269834] [gbkey=CDS]	777	87.10%	47.40%
meC0281	[locus_tag=DQM41_RS01770]	lcl|NZ_LS483362.1_cds_WP_002872052.1_346	85	1044	960	forward	85	1044	960	85	1044	[gene=cmeB] [protein=multidrug efflux RND transporter permease subunit CmeB] [protein_id=WP_002872052.1] [location=complement(332589..335711)] [gbkey=CDS]	3123	95.20%	96.00%
meC0281	[locus_tag=DQM41_RS01770]	lcl|NZ_LS483362.1_cds_WP_002872052.1_346	1048	2046	999	forward	1048	2046	999	1048	2046	[gene=cmeB][protein=multidrug efflux RND transporter permease subunit CmeB] [protein_id=WP_002872052.1] [location=complement(332589..335711)] [gbkey=CDS]	3123	86.40%	99.90%
meC0467	[locus_tag=DQM41_RS01770]	lcl|NZ_LS483362.1_cds_WP_002872052.1_346	321	614	294	forward	321	614	294	321	614	[gene=cmeB] [protein=multidrug efflux RND transporter permease subunit CmeB] [protein_id=WP_002872052.1] [location=complement(332589..335711)] [gbkey=CDS]	3123	91.80%	29.40%
meC0281	[locus_tag=DQM41_RS00145]	lcl|NZ_LS483362.1_cds_WP_072233179.1_29	6	374	369	forward	6	374	369	6	374	[protein=type II asparaginase] [protein_id=WP_072233179.1] [location=37625..38662] [gbkey=CDS]	1038	96.00%	36.90%
meC0467	[locus_tag=DQM41_RS00145]	lcl|NZ_LS483362.1_cds_WP_072233179.1_29	625	858	234	forward	625	858	234	625	858	[protein=type II asparaginase] [protein_id=WP_072233179.1] [location=37625..38662] [gbkey=CDS]	1038	82.10%	23.40%
meC0281	[locus_tag=DQM41_RS00125]	lcl|NZ_LS483362.1_cds_WP_002865086.1_25	3	548	546	reverse	3	548	546	3	548	[protein=L-cystine transporter] [protein_id=WP_002865086.1] [location=complement(32134..33519)] [gbkey=CDS]	1386	96.20%	54.60%
meC0467	[locus_tag=DQM41_RS00125]	lcl|NZ_LS483362.1_cds_WP_002865086.1_25	403	1236	834	reverse	403	1236	834	403	1236	[protein=L-cystine transporter] [protein_id=WP_002865086.1] [location=complement(32134..33519)] [gbkey=CDS]	1386	84.70%	83.40%
meC0281	[locus_tag=DQM41_RS02720]	lcl|NZ_LS483362.1_cds_WP_002864504.1_525	412	1410	999	reverse	412	1410	999	412	1410	[gene=fliD] [protein=flagellar filament capping protein FliD] [protein_id=WP_002864504.1] [location=510546..512474] [gbkey=CDS]	1929	96.10%	99.90%
meC0467	[locus_tag=DQM41_RS02720]	lcl|NZ_LS483362.1_cds_WP_002864504.1_525	43	900	858	reverse	43	900	858	43	900	[gene=fliD] [protein=flagellar filament capping protein FliD] [protein_id=WP_002864504.1] [location=510546..512474] [gbkey=CDS]	1929	99.00%	85.80%
meC0281	[locus_tag=DQM41_RS03650]	lcl|NZ_LS483362.1_cds_WP_002784190.1_708	1	630	630	reverse	1	630	630	1	630	[protein=ABC transporter permease] [protein_id=WP_002784190.1] [location=684643..685482] [gbkey=CDS]	840	98.60%	63.00%
meC0467	[locus_tag=DQM41_RS03650]	lcl|NZ_LS483362.1_cds_WP_002784190.1_708	528	839	312	reverse	528	839	312	528	839	[protein=ABC transporter permease] [protein_id=WP_002784190.1] [location=684643..685482] [gbkey=CDS]	840	98.10%	31.20%
meC0281	[locus_tag=DQM41_RS05225]	lcl|NZ_LS483362.1_cds_WP_010891903.1_1007	685	1500	816	forward	685	1500	816	685	1500	[protein=endonuclease MutS2] [protein_id=WP_010891903.1] [location=complement(987001..989211)] [gbkey=CDS]	2211	100.00%	81.60%
meC0467	[locus_tag=DQM41_RS05225]	lcl|NZ_LS483362.1_cds_WP_010891903.1_1007	577	1500	924	forward	577	1500	924	577	1500	[protein=endonuclease MutS2] [protein_id=WP_010891903.1] [location=complement(987001..989211)] [gbkey=CDS]	2211	99.40%	92.40%
meC0281	[locus_tag=DQM41_RS03090]	lcl|NZ_LS483362.1_cds_WP_002906848.1_596	276	929	654	reverse	276	929	654	276	929	[protein=MATE family efflux transporter] [protein_id=WP_002906848.1] [location=578335..579651] [gbkey=CDS]	1317	93.10%	65.40%
meC0467	[locus_tag=DQM41_RS03090]	lcl|NZ_LS483362.1_cds_WP_002906848.1_596	323	1315	993	reverse	323	1315	993	323	1315	[protein=MATE family efflux transporter] [protein_id=WP_002906848.1] [location=578335..579651] [gbkey=CDS]	1317	91.10%	99.30%
meC0281	[locus_tag=DQM41_RS05130]	lcl|NZ_LS483362.1_cds_WP_010891901.1_988	2217	2615	399	reverse	2217	2615	399	2217	2615	[gene=cmeF] [protein=multidrug efflux RND transporter permease subunit CmeF] [protein_id=WP_010891901.1] [location=965475..968492] [gbkey=CDS]	3018	100.00%	39.90%
meC0281	[locus_tag=DQM41_RS05130]	lcl|NZ_LS483362.1_cds_WP_010891901.1_988	1216	2214	999	reverse	1216	2214	999	1216	2214	[gene=cmeF] [protein=multidrug efflux RND transporter permease subunit CmeF] [protein_id=WP_010891901.1] [location=965475..968492] [gbkey=CDS]	3018	98.50%	99.90%
meC0281	[locus_tag=DQM41_RS05130]	lcl|NZ_LS483362.1_cds_WP_010891901.1_988	217	1173	957	reverse	217	1173	957	217	1173	[gene=cmeF] [protein=multidrug efflux RND transporter permease subunit CmeF] [protein_id=WP_010891901.1] [location=965475..968492] [gbkey=CDS]	3018	98.10%	95.70%
meC0467	[locus_tag=DQM41_RS05130]	lcl|NZ_LS483362.1_cds_WP_010891901.1_988	1237	2232	996	reverse	1237	2232	996	1237	2232	[gene=cmeF] [protein=multidrug efflux RND transporter permease subunit CmeF] [protein_id=WP_010891901.1] [location=965475..968492] [gbkey=CDS]	3018	99.40%	99.60%
meC0467	[locus_tag=DQM41_RS05130]	lcl|NZ_LS483362.1_cds_WP_010891901.1_988	235	1173	939	reverse	235	1173	939	235	1173	[gene=cmeF] [protein=multidrug efflux RND transporter permease subunit CmeF] [protein_id=WP_010891901.1] [location=965475..968492] [gbkey=CDS]	3018	96.20%	93.90%
meC0281	[locus_tag=DQM41_RS05125]	lcl|NZ_LS483362.1_cds_WP_002858463.1_987	1	741	741	reverse	1	741	741	1	741	[gene=cmeE] [protein=multidrug efflux RND transporter periplasmic adaptor subunit CmeE] [protein_id=WP_002858463.1] [location=964732..965472] [gbkey=CDS]	741	97.60%	74.10%
meC0467	[locus_tag=DQM41_RS05125]	lcl|NZ_LS483362.1_cds_WP_002858463.1_987	1	741	741	reverse	1	741	741	1	741	[gene=cmeE] [protein=multidrug efflux RND transporter periplasmic adaptor subunit CmeE] [protein_id=WP_002858463.1] [location=964732..965472] [gbkey=CDS]	741	98.40%	74.10%
meC0281	[locus_tag=DQM41_RS00035]	lcl|NZ_LS483362.1_cds_WP_002853155.1_7	1042	2040	999	forward	1042	2040	999	1042	2040	[gene=gltB] [protein=glutamate synthase large subunit] [protein_id=WP_002853155.1] [location=8144..12634] [gbkey=CDS]	4491	97.30%	99.90%
meC0281	[locus_tag=DQM41_RS00035]	lcl|NZ_LS483362.1_cds_WP_002853155.1_7	2041	3039	999	forward	2041	3039	999	2041	3039	[gene=gltB] [protein=glutamate synthase large subunit] [protein_id=WP_002853155.1] [location=8144..12634] [gbkey=CDS]	4491	98.50%	99.90%
meC0467	[locus_tag=DQM41_RS00035]	lcl|NZ_LS483362.1_cds_WP_002853155.1_7	2683	3678	996	forward	2683	3678	996	2683	3678	[gene=gltB] [protein=glutamate synthase large subunit] [protein_id=WP_002853155.1] [location=8144..12634] [gbkey=CDS]	4491	100.00%	99.90%
meC0467	[locus_tag=DQM41_RS00035]	lcl|NZ_LS483362.1_cds_WP_002853155.1_7	1681	2679	999	forward	1681	2679	999	1681	2679	[gene=gltB] [protein=glutamate synthase large subunit] [protein_id=WP_002853155.1] [location=8144..12634] [gbkey=CDS]	4491	94.70%	99.60%
meC0281	[locus_tag=DQM41_RS05115]	lcl|NZ_LS483362.1_cds_WP_002837927.1_985	811	1647	837	forward	811	1647	837	811	1647	[gene=lepA][protein=elongation factor 4] [protein_id=WP_002837927.1] [location=complement(961538..963334)] [gbkey=CDS]	1797	95.20%	83.70%
meC0467	[locus_tag=DQM41_RS05115]	lcl|NZ_LS483362.1_cds_WP_002837927.1_985	1	627	627	forward	1	627	627	1	627	[gene=lepA] [protein=elongation factor 4] [protein_id=WP_002837927.1] [location=complement(961538..963334)] [gbkey=CDS]	1797	94.50%	62.70%
meC0281	[locus_tag=DQM41_RS00120]	lcl|NZ_LS483362.1_cds_WP_002865087.1_24	904	1902	999	forward	904	1902	999	904	1902	[protein=ribonucleoside-diphosphate reductase subunit alpha] [protein_id=WP_002865087.1] [location=29726..32095] [gbkey=CDS]	2370	97.90%	99.90%
meC0281	[locus_tag=DQM41_RS00120]	lcl|NZ_LS483362.1_cds_WP_002865087.1_24	1906	2370	465	forward	1906	2370	465	1906	2370	[protein=ribonucleoside-diphosphate reductase subunit alpha] [protein_id=WP_002865087.1] [location=29726..32095] [gbkey=CDS]	2370	98.70%	46.50%
meC0467	[locus_tag=DQM41_RS00120]	lcl|NZ_LS483362.1_cds_WP_002865087.1_24	1552	2370	819	forward	1552	2370	819	1552	2370	[protein=ribonucleoside-diphosphate reductase subunit alpha] [protein_id=WP_002865087.1] [location=29726..32095] [gbkey=CDS]	2370	97.80%	54.90%
meC0467	[locus_tag=DQM41_RS00120]	lcl|NZ_LS483362.1_cds_WP_002865087.1_24	1	549	549	forward	1	549	549	1	549	[protein=ribonucleoside-diphosphate reductase subunit alpha] [protein_id=WP_002865087.1] [location=29726..32095] [gbkey=CDS]	2370	94.90%	81.90%
meC0281	[locus_tag=DQM41_RS03080]	lcl|NZ_LS483362.1_cds_WP_010891867.1_594	52	741	690	reverse	52	741	690	52	741	[protein=phosphate ABC transporter ATP-binding protein] [protein_id=WP_010891867.1] [location=576216..576956] [gbkey=CDS]	741	99.60%	69.00%
meC0467	[locus_tag=DQM41_RS03080]	lcl|NZ_LS483362.1_cds_WP_010891867.1_594	1	579	579	reverse	1	579	579	1	579	[protein=phosphate ABC transporter ATP-binding protein] [protein_id=WP_010891867.1] [location=576216..576956] [gbkey=CDS]	741	99.50%	57.90%
meC0281	[locus_tag=DQM41_RS03765]	lcl|NZ_LS483362.1_cds_WP_002826316.1_726	106	1104	999	reverse	106	1104	999	106	1104	[gene=dnaK] [protein=molecular chaperone DnaK] [protein_id=WP_002826316.1] [location=709168..711039] [gbkey=CDS]	1872	98.20%	99.90%
meC0467	[locus_tag=DQM41_RS03765]	lcl|NZ_LS483362.1_cds_WP_002826316.1_726	535	1530	996	reverse	535	1530	996	535	1530	[gene=dnaK] [protein=molecular chaperone DnaK] [protein_id=WP_002826316.1] [location=709168..711039] [gbkey=CDS]	1872	94.20%	99.60%
meC0281	[locus_tag=DQM41_RS00285]	lcl|NZ_LS483362.1_cds_WP_002851977.1_51	1	456	456	reverse	1	456	456	1	456	[gene=mnmA] [protein=tRNA 2-thiouridine(34) synthase MnmA] [protein_id=WP_002851977.1] [location=complement(68528..69544)] [gbkey=CDS]	1017	99.30%	45.60%
meC0467	[locus_tag=DQM41_RS00285]	lcl|NZ_LS483362.1_cds_WP_002851977.1_51	805	1017	213	reverse	805	1017	213	805	1017	[gene=mnmA] [protein=tRNA 2-thiouridine(34) synthase MnmA] [protein_id=WP_002851977.1] [location=complement(68528..69544)] [gbkey=CDS]	1017	88.70%	21.30%
meC0467	[locus_tag=DQM41_RS00285]	lcl|NZ_LS483362.1_cds_WP_002851977.1_51	1	801	801	reverse	1	801	801	1	801	[gene=mnmA] [protein=tRNA 2-thiouridine(34) synthase MnmA] [protein_id=WP_002851977.1] [location=complement(68528..69544)] [gbkey=CDS]	1017	98.90%	80.10%
meC0281	[locus_tag=DQM41_RS00830]	lcl|NZ_LS483362.1_cds_WP_002840050.1_160	13	813	801	forward	13	813	801	13	813	[protein=iron-sulfur cluster assembly scaffold protein NifU] [protein_id=WP_002840050.1] [location=164625..165596] [gbkey=CDS]	972	99.60%	80.10%
meC0467	[locus_tag=DQM41_RS00830]	lcl|NZ_LS483362.1_cds_WP_002840050.1_160	133	972	840	forward	133	972	840	133	972	[protein=iron-sulfur cluster assembly scaffold protein NifU] [protein_id=WP_002840050.1] [location=164625..165596] [gbkey=CDS]	972	95.60%	84.00%
meC0281	[locus_tag=DQM41_RS02420]	lcl|NZ_LS483362.1_cds_WP_002864166.1_466	1	561	561	reverse	1	561	561	1	561	[gene=fucP] [protein=L-fucose:H+ symporter permease] [protein_id=WP_002864166.1] [location=453113..454369] [gbkey=CDS]	1257	89.10%	56.10%
meC0467	[locus_tag=DQM41_RS02420]	lcl|NZ_LS483362.1_cds_WP_002864166.1_466	619	1236	618	reverse	619	1236	618	619	1236	[gene=fucP] [protein=L-fucose:H+ symporter permease] [protein_id=WP_002864166.1] [location=453113..454369] [gbkey=CDS]	1257	92.20%	61.80%
meC0281	[locus_tag=DQM41_RS03750]	lcl|NZ_LS483362.1_cds_WP_010891883.1_723	841	1836	996	reverse	841	1836	996	841	1836	[gene=cfrA] [protein=TonB-dependent ferric enterobactin receptor CfrA] [protein_id=WP_010891883.1] [location=705435..707525] [gbkey=CDS]	2091	99.70%	99.60%
meC0467	[locus_tag=DQM41_RS03750]	lcl|NZ_LS483362.1_cds_WP_010891883.1_723	268	1266	999	reverse	268	1266	999	268	1266	[gene=cfrA] [protein=TonB-dependent ferric enterobactin receptor CfrA] [protein_id=WP_010891883.1] [location=705435..707525] [gbkey=CDS]	2091	100.00%	99.90%
meC0281	[locus_tag=DQM41_RS03120]	lcl|NZ_LS483362.1_cds_WP_002776343.1_602	178	1092	915	reverse	178	1092	915	178	1092	[gene=hypD] [protein=hydrogenase formation protein HypD] [protein_id=WP_002776343.1] [location=585092..586183] [gbkey=CDS]	1092	99.70%	91.50%
meC0467	[locus_tag=DQM41_RS03120]	lcl|NZ_LS483362.1_cds_WP_002776343.1_602	1	564	564	reverse	1	564	564	1	564	[gene=hypD] [protein=hydrogenase formation protein HypD] [protein_id=WP_002776343.1] [location=585092..586183] [gbkey=CDS]	1092	99.50%	56.40%
meC0281	[locus_tag=DQM41_RS06670]	lcl|NZ_LS483362.1_cds_WP_002864247.1_1293	316	663	348	forward	316	663	348	316	663	[protein=DUF2920 family protein] [protein_id=WP_002864247.1] [location=complement(1274676..1275917)] [gbkey=CDS]	1242	60.30%	34.80%
meC0467	[locus_tag=DQM41_RS06670]	lcl|NZ_LS483362.1_cds_WP_002864247.1_1293	563	1108	546	forward	563	1108	546	563	1108	[protein=DUF2920 family protein] [protein_id=WP_002864247.1] [location=complement(1274676..1275917)] [gbkey=CDS]	1242	89.60%	54.60%
meC0281	[locus_tag=DQM41_RS06490]	lcl|NZ_LS483362.1_cds_WP_002858432.1_1257	1	636	636	forward	1	636	636	1	636	[protein=DUF2920 family protein] [protein_id=WP_002858432.1] [location=complement(1235506..1236732)] [gbkey=CDS]	1227	95.50%	63.60%
meC0281	[locus_tag=DQM41_RS06490]	lcl|NZ_LS483362.1_cds_WP_002858432.1_1257	637	1227	591	forward	637	1227	591	637	1227	[protein=DUF2920 family protein] [protein_id=WP_002858432.1] [location=complement(1235506..1236732)] [gbkey=CDS]	1227	99.50%	59.10%
meC0281	[locus_tag=DQM41_RS06490]	lcl|NZ_LS483362.1_cds_WP_002858432.1_1257	733	1221	489	forward	733	1221	489	733	1221	[protein=DUF2920 family protein] [protein_id=WP_002858432.1] [location=complement(1235506..1236732)] [gbkey=CDS]	1227	86.50%	48.90%
meC0467	[locus_tag=DQM41_RS06490]	lcl|NZ_LS483362.1_cds_WP_002858432.1_1257	733	1221	489	forward	733	1221	489	733	1221	[protein=DUF2920 family protein] [protein_id=WP_002858432.1] [location=complement(1235506..1236732)] [gbkey=CDS]	1227	87.70%	48.90%
meC0281	[locus_tag=DQM41_RS08350]	lcl|NZ_LS483362.1_cds_WP_002851268.1_1617	169	1167	999	reverse	169	1167	999	169	1167	[protein=type I DNA topoisomerase] [protein_id=WP_002851268.1] [location=complement(1607343..1609445)] [gbkey=CDS]	2103	93.90%	99.90%
meC0467	[locus_tag=DQM41_RS08350]	lcl|NZ_LS483362.1_cds_WP_002851268.1_1617	1	912	912	reverse	1	912	912	1	912	[protein=type I DNA topoisomerase] [protein_id=WP_002851268.1] [location=complement(1607343..1609445)] [gbkey=CDS]	2103	97.00%	91.20%
meC0281	[locus_tag=DQM41_RS08570]	lcl|NZ_LS483362.1_cds_WP_002853122.1_1657	1	630	630	reverse	1	630	630	1	630	[protein=O-acetylhomoserine aminocarboxypropyltransferase/cysteine synthase] [protein_id=WP_002853122.1] [location=complement(1636390..1637661)] [gbkey=CDS]	1272	96.20%	63.00%
meC0467	[locus_tag=DQM41_RS08570]	lcl|NZ_LS483362.1_cds_WP_002853122.1_1657	1	357	357	reverse	1	357	357	1	357	[protein=O-acetylhomoserine aminocarboxypropyltransferase/cysteine synthase] [protein_id=WP_002853122.1] [location=complement(1636390..1637661)] [gbkey=CDS]	1272	96.60%	35.70%
meC0281	[locus_tag=DQM41_RS06770]	lcl|NZ_LS483362.1_cds_WP_002859649.1_1311	34	453	420	forward	34	453	420	34	453	[protein=hypothetical protein] [protein_id=WP_002859649.1] [location=complement(1293641..1294093)] [gbkey=CDS]	453	98.60%	42.00%
meC0467	[locus_tag=DQM41_RS06770]	lcl|NZ_LS483362.1_cds_WP_002859649.1_1311	1	453	453	forward	1	453	453	1	453	[protein=hypothetical protein] [protein_id=WP_002859649.1] [location=complement(1293641..1294093)] [gbkey=CDS]	453	100.00%	45.30%
meC0281	[locus_tag=DQM41_RS00190]	lcl|NZ_LS483362.1_cds_WP_002866314.1_33	64	543	480	reverse	64	543	480	64	543	[protein=SIMPL domain-containing protein] [protein_id=WP_002866314.1] [location=complement(51963..52664)] [gbkey=CDS]	702	93.10%	48.00%
meC0467	[locus_tag=DQM41_RS00190]	lcl|NZ_LS483362.1_cds_WP_002866314.1_33	64	702	639	reverse	64	702	639	64	702	[protein=SIMPL domain-containing protein] [protein_id=WP_002866314.1] [location=complement(51963..52664)] [gbkey=CDS]	702	90.60%	63.90%
meC0281	[locus_tag=DQM41_RS00200]	lcl|NZ_LS483362.1_cds_WP_010891829.1_35	553	1146	594	forward	553	1146	594	553	1146	[protein=DUF2130 domain-containing protein] [protein_id=WP_010891829.1] [location=53966..55315] [gbkey=CDS]	1350	96.50%	59.40%
meC0467	[locus_tag=DQM41_RS00200]	lcl|NZ_LS483362.1_cds_WP_010891829.1_35	805	1350	546	forward	805	1350	546	805	1350	[protein=DUF2130 domain-containing protein] [protein_id=WP_010891829.1] [location=53966..55315] [gbkey=CDS]	1350	97.30%	54.60%

Table S7: Common genes with putative *C. jejuni* origin of meC0280, meC0281, and meC0467.

genome	locus_tag	Sequence Name	Minimum	Maximum	Length	Direction	Min (with gaps)	Max (with gaps)	Length (with gaps)	Min (original sequence)	Max (original sequence)	Description	Sequence Length	% Pairwise Identity	Query Coverage
meC0280	[locus_tag=DQM41_RS07815]	lcl|NZ_LS483362.1_cds_WP_002851210.1_1519	169	1167	999	forward	169	1167	999	169	1167	[protein=FAD-binding oxidoreductase] [protein_id=WP_002851210.1] [location=complement(1514627..1517398)] [gbkey=CDS]	2772	91.30%	99.90%
meC0280	[locus_tag=DQM41_RS07815]	lcl|NZ_LS483362.1_cds_WP_002851210.1_1519	1168	2166	999	forward	1168	2166	999	1168	2166	[protein=FAD-binding oxidoreductase] [protein_id=WP_002851210.1] [location=complement(1514627..1517398)] [gbkey=CDS]	2772	98.50%	99.90%
meC0281	[locus_tag=DQM41_RS07815]	lcl|NZ_LS483362.1_cds_WP_002851210.1_1519	325	1323	999	forward	325	1323	999	325	1323	[protein=FAD-binding oxidoreductase] [protein_id=WP_002851210.1] [location=complement(1514627..1517398)] [gbkey=CDS]	2772	95.10%	99.90%
meC0467	[locus_tag=DQM41_RS07815]	lcl|NZ_LS483362.1_cds_WP_002851210.1_1519	1	399	399	forward	1	399	399	1	399	[protein=FAD-binding oxidoreductase] [protein_id=WP_002851210.1] [location=complement(1514627..1517398)] [gbkey=CDS]	2772	89.90%	39.90%
meC0280	[locus_tag=DQM41_RS03100]	lcl|NZ_LS483362.1_cds_WP_002826526.1_598	466	1461	996	forward	466	1461	996	466	1461	[protein=hypothetical protein] [protein_id=WP_002826526.1] [location=580316..581836] [gbkey=CDS]	1521	91.50%	99.60%
meC0281	[locus_tag=DQM41_RS03100]	lcl|NZ_LS483362.1_cds_WP_002826526.1_598	1	948	948	reverse	1	948	948	1	948	[protein=hypothetical protein] [protein_id=WP_002826526.1] [location=580316..581836] [gbkey=CDS]	1521	98.70%	94.80%
meC0467	[locus_tag=DQM41_RS03100]	lcl|NZ_LS483362.1_cds_WP_002826526.1_598	343	1338	996	reverse	343	1338	996	343	1338	[protein=hypothetical protein] [protein_id=WP_002826526.1] [location=580316..581836] [gbkey=CDS]	1521	91.60%	99.60%
meC0280	[locus_tag=DQM41_RS01430]	lcl|NZ_LS483362.1_cds_WP_002778123.1_278	447	848	402	reverse	447	848	402	447	848	[protein=pantoate--beta-alanine ligase] [protein_id=WP_002778123.1] [location=complement(271412..272260)] [gbkey=CDS]	849	99.30%	40.20%
meC0281	[locus_tag=DQM41_RS01430]	lcl|NZ_LS483362.1_cds_WP_002778123.1_278	405	848	444	forward	405	848	444	405	848	[protein=pantoate--beta-alanine ligase] [protein_id=WP_002778123.1] [location=complement(271412..272260)] [gbkey=CDS]	849	99.30%	44.40%
meC0467	[locus_tag=DQM41_RS01430]	lcl|NZ_LS483362.1_cds_WP_002778123.1_278	3	728	726	forward	3	728	726	3	728	[protein=pantoate--beta-alanine ligase] [protein_id=WP_002778123.1] [location=complement(271412..272260)] [gbkey=CDS]	849	99.20%	72.60%
meC0280	[locus_tag=DQM41_RS04280]	lcl|NZ_LS483362.1_cds_WP_002864843.1_829	1441	1509	69	forward	1441	1509	69	1441	1509	[protein=chorismate-binding protein] [protein_id=WP_002864843.1] [location=complement(807480..809264)] [gbkey=CDS]	1785	47.80%	6.90%
meC0281	[locus_tag=DQM41_RS04280]	lcl|NZ_LS483362.1_cds_WP_002864843.1_829	1441	1509	69	forward	1441	1509	69	1441	1509	[protein=chorismate-binding protein] [protein_id=WP_002864843.1] [location=complement(807480..809264)] [gbkey=CDS]	1785	47.80%	6.90%
meC0281	[locus_tag=DQM41_RS04280]	lcl|NZ_LS483362.1_cds_WP_002864843.1_829	1441	1509	69	reverse	1441	1509	69	1441	1509	[protein=chorismate-binding protein] [protein_id=WP_002864843.1] [location=complement(807480..809264)] [gbkey=CDS]	1785	47.80%	6.90%
meC0281	[locus_tag=DQM41_RS04280]	lcl|NZ_LS483362.1_cds_WP_002864843.1_829	1441	1509	69	reverse	1441	1509	69	1441	1509	[protein=chorismate-binding protein] [protein_id=WP_002864843.1] [location=complement(807480..809264)] [gbkey=CDS]	1785	47.80%	6.90%
meC0467	[locus_tag=DQM41_RS04280]	lcl|NZ_LS483362.1_cds_WP_002864843.1_829	1441	1509	69	forward	1441	1509	69	1441	1509	[protein=chorismate-binding protein] [protein_id=WP_002864843.1] [location=complement(807480..809264)] [gbkey=CDS]	1785	47.80%	6.90%
meC0280	[locus_tag=DQM41_RS03640]	lcl|NZ_LS483362.1_cds_WP_002793291.1_706	484	1326	843	forward	484	1326	843	484	1326	[protein=esterase-like activity of phytase family protein] [protein_id=WP_002793291.1] [location=682515..683840] [gbkey=CDS]	1326	98.60%	84.30%
meC0281	[locus_tag=DQM41_RS03640]	lcl|NZ_LS483362.1_cds_WP_002793291.1_706	1	948	948	reverse	1	948	948	1	948	[protein=esterase-like activity of phytase family protein] [protein_id=WP_002793291.1] [location=682515..683840] [gbkey=CDS]	1326	99.40%	94.80%
meC0467	[locus_tag=DQM41_RS03640]	lcl|NZ_LS483362.1_cds_WP_002793291.1_706	343	1326	984	reverse	343	1326	984	343	1326	[protein=esterase-like activity of phytase family protein] [protein_id=WP_002793291.1] [location=682515..683840] [gbkey=CDS]	1326	99.40%	98.40%
meC0280	[locus_tag=DQM41_RS05220]	lcl|NZ_LS483362.1_cds_WP_002864285.1_1006	1903	2628	726	reverse	1903	2628	726	1903	2628	[protein=N-6 DNA methylase] [protein_id=WP_002864285.1] [location=complement(982973..986992)] [gbkey=CDS]	4020	94.20%	72.60%
meC0280	[locus_tag=DQM41_RS05220]	lcl|NZ_LS483362.1_cds_WP_002864285.1_1006	901	1899	999	reverse	901	1899	999	901	1899	[protein=N-6 DNA methylase] [protein_id=WP_002864285.1] [location=complement(982973..986992)] [gbkey=CDS]	4020	100.00%	99.90%
meC0280	[locus_tag=DQM41_RS05220]	lcl|NZ_LS483362.1_cds_WP_002864285.1_1006	1	900	900	reverse	1	900	900	1	900	[protein=N-6 DNA methylase] [protein_id=WP_002864285.1] [location=complement(982973..986992)] [gbkey=CDS]	4020	99.00%	90.00%
meC0281	[locus_tag=DQM41_RS05220]	lcl|NZ_LS483362.1_cds_WP_002864285.1_1006	466	1464	999	forward	466	1464	999	466	1464	[protein=N-6 DNA methylase] [protein_id=WP_002864285.1] [location=complement(982973..986992)] [gbkey=CDS]	4020	99.70%	99.90%
meC0281	[locus_tag=DQM41_RS05220]	lcl|NZ_LS483362.1_cds_WP_002864285.1_1006	1465	2463	999	forward	1465	2463	999	1465	2463	[protein=N-6 DNA methylase] [protein_id=WP_002864285.1] [location=complement(982973..986992)] [gbkey=CDS]	4020	98.20%	99.90%
meC0281	[locus_tag=DQM41_RS05220]	lcl|NZ_LS483362.1_cds_WP_002864285.1_1006	3391	4020	630	forward	3391	4020	630	3391	4020	[protein=N-6 DNA methylase] [protein_id=WP_002864285.1] [location=complement(982973..986992)] [gbkey=CDS]	4020	93.60%	63.00%
meC0467	[locus_tag=DQM41_RS05220]	lcl|NZ_LS483362.1_cds_WP_002864285.1_1006	2359	3354	996	forward	2359	3354	996	2359	3354	[protein=N-6 DNA methylase] [protein_id=WP_002864285.1] [location=complement(982973..986992)] [gbkey=CDS]	4020	100.00%	99.90%
meC0467	[locus_tag=DQM41_RS05220]	lcl|NZ_LS483362.1_cds_WP_002864285.1_1006	1357	2355	999	forward	1357	2355	999	1357	2355	[protein=N-6 DNA methylase] [protein_id=WP_002864285.1] [location=complement(982973..986992)] [gbkey=CDS]	4020	98.80%	99.90%
meC0467	[locus_tag=DQM41_RS05220]	lcl|NZ_LS483362.1_cds_WP_002864285.1_1006	358	1356	999	forward	358	1356	999	358	1356	[protein=N-6 DNA methylase] [protein_id=WP_002864285.1] [location=complement(982973..986992)] [gbkey=CDS]	4020	98.50%	99.60%
meC0280	[locus_tag=DQM41_RS05910]	lcl|NZ_LS483362.1_cds_WP_002852850.1_1144	46	519	474	forward	46	519	474	46	519	[protein=cation:dicarboxylase symporter family transporter] [protein_id=WP_002852850.1] [location=1119951..1121327] [gbkey=CDS]	1377	98.70%	47.40%
meC0281	[locus_tag=DQM41_RS05910]	lcl|NZ_LS483362.1_cds_WP_002852850.1_1144	817	1377	561	reverse	817	1377	561	817	1377	[protein=cation:dicarboxylase symporter family transporter] [protein_id=WP_002852850.1] [location=1119951..1121327] [gbkey=CDS]	1377	100.00%	56.10%
meC0281	[locus_tag=DQM41_RS05910]	lcl|NZ_LS483362.1_cds_WP_002852850.1_1144	46	813	768	reverse	46	813	768	46	813	[protein=cation:dicarboxylase symporter family transporter] [protein_id=WP_002852850.1] [location=1119951..1121327] [gbkey=CDS]	1377	94.70%	76.80%
meC0467	[locus_tag=DQM41_RS05910]	lcl|NZ_LS483362.1_cds_WP_002852850.1_1144	370	1365	996	reverse	370	1365	996	370	1365	[protein=cation:dicarboxylase symporter family transporter] [protein_id=WP_002852850.1] [location=1119951..1121327] [gbkey=CDS]	1377	95.70%	99.60%
meC0280	[locus_tag=DQM41_RS06505]	lcl|NZ_LS483362.1_cds_WP_002856137.1_1260	586	1068	483	reverse	586	1068	483	586	1068	[protein=hypothetical protein] [protein_id=WP_002856137.1] [location=complement(1238565..1239635)] [gbkey=CDS]	1071	100.00%	48.30%
meC0281	[locus_tag=DQM41_RS06505]	lcl|NZ_LS483362.1_cds_WP_002856137.1_1260	280	936	657	forward	280	936	657	280	936	[protein=hypothetical protein] [protein_id=WP_002856137.1] [location=complement(1238565..1239635)] [gbkey=CDS]	1071	99.10%	65.70%
meC0467	[locus_tag=DQM41_RS06505]	lcl|NZ_LS483362.1_cds_WP_002856137.1_1260	280	849	570	forward	280	849	570	280	849	[protein=hypothetical protein] [protein_id=WP_002856137.1] [location=complement(1238565..1239635)] [gbkey=CDS]	1071	98.90%	57.00%
meC0280	[locus_tag=DQM41_RS07490]	lcl|NZ_LS483362.1_cds_WP_010891941.1_1454	1	717	717	reverse	1	717	717	1	717	[protein=formate dehydrogenase subunit alpha] [transl_except=(pos:541..543,aa:Sec)] [protein_id=WP_010891941.1] [location=complement(1446324..1449128)] [gbkey=CDS]	2805	91.00%	71.70%
meC0281	[locus_tag=DQM41_RS07490]	lcl|NZ_LS483362.1_cds_WP_010891941.1_1454	847	1422	576	forward	847	1422	576	847	1422	[protein=formate dehydrogenase subunit alpha] [transl_except=(pos:541..543,aa:Sec)] [protein_id=WP_010891941.1] [location=complement(1446324..1449128)] [gbkey=CDS]	2805	97.40%	57.60%
meC0467	[locus_tag=DQM41_RS07490]	lcl|NZ_LS483362.1_cds_WP_010891941.1_1454	1471	2157	687	forward	1471	2157	687	1471	2157	[protein=formate dehydrogenase subunit alpha] [transl_except=(pos:541..543,aa:Sec)] [protein_id=WP_010891941.1] [location=complement(1446324..1449128)] [gbkey=CDS]	2805	95.20%	68.70%
meC0280	[locus_tag=DQM41_RS02380]	lcl|NZ_LS483362.1_cds_WP_010891860.1_458	1810	2808	999	forward	1810	2808	999	1810	2808	[gene=rpoB] [protein=DNA-directed RNA polymerase subunit beta] [protein_id=WP_010891860.1] [location=438986..443122] [gbkey=CDS]	4137	99.70%	99.90%
meC0281	[locus_tag=DQM41_RS02380]	lcl|NZ_LS483362.1_cds_WP_010891860.1_458	2131	3129	999	reverse	2131	3129	999	2131	3129	[gene=rpoB] [protein=DNA-directed RNA polymerase subunit beta] [protein_id=WP_010891860.1] [location=438986..443122] [gbkey=CDS]	4137	99.70%	99.90%
meC0281	[locus_tag=DQM41_RS02380]	lcl|NZ_LS483362.1_cds_WP_010891860.1_458	1132	2127	996	reverse	1132	2127	996	1132	2127	[gene=rpoB] [protein=DNA-directed RNA polymerase subunit beta] [protein_id=WP_010891860.1] [location=438986..443122] [gbkey=CDS]	4137	98.00%	99.60%
meC0467	[locus_tag=DQM41_RS02380]	lcl|NZ_LS483362.1_cds_WP_010891860.1_458	3616	4137	522	reverse	3616	4137	522	3616	4137	[gene=rpoB] [protein=DNA-directed RNA polymerase subunit beta] [protein_id=WP_010891860.1] [location=438986..443122] [gbkey=CDS]	4137	97.70%	52.20%
meC0467	[locus_tag=DQM41_RS02380]	lcl|NZ_LS483362.1_cds_WP_010891860.1_458	1615	2613	999	reverse	1615	2613	999	1615	2613	[gene=rpoB] [protein=DNA-directed RNA polymerase subunit beta] [protein_id=WP_010891860.1] [location=438986..443122] [gbkey=CDS]	4137	99.40%	99.90%
meC0280	[locus_tag=DQM41_RS01770]	lcl|NZ_LS483362.1_cds_WP_002872052.1_346	1080	2063	984	reverse	1080	2063	984	1080	2063	[gene=cmeB] [protein=multidrug efflux RND transporter permease subunit CmeB] [protein_id=WP_002872052.1] [location=complement(332589..335711)] [gbkey=CDS]	3123	92.90%	98.40%
meC0281	[locus_tag=DQM41_RS01770]	lcl|NZ_LS483362.1_cds_WP_002872052.1_346	85	1044	960	forward	85	1044	960	85	1044	[gene=cmeB] [protein=multidrug efflux RND transporter permease subunit CmeB] [protein_id=WP_002872052.1] [location=complement(332589..335711)] [gbkey=CDS]	3123	95.20%	96.00%
meC0281	[locus_tag=DQM41_RS01770]	lcl|NZ_LS483362.1_cds_WP_002872052.1_346	1048	2046	999	forward	1048	2046	999	1048	2046	[gene=cmeB][protein=multidrug efflux RND transporter permease subunit CmeB] [protein_id=WP_002872052.1] [location=complement(332589..335711)] [gbkey=CDS]	3123	86.40%	99.90%
meC0467	[locus_tag=DQM41_RS01770]	lcl|NZ_LS483362.1_cds_WP_002872052.1_346	321	614	294	forward	321	614	294	321	614	[gene=cmeB] [protein=multidrug efflux RND transporter permease subunit CmeB] [protein_id=WP_002872052.1] [location=complement(332589..335711)] [gbkey=CDS]	3123	91.80%	29.40%
meC0280	[locus_tag=DQM41_RS02720]	lcl|NZ_LS483362.1_cds_WP_002864504.1_525	43	690	648	forward	43	690	648	43	690	[gene=fliD] [protein=flagellar filament capping protein FliD] [protein_id=WP_002864504.1] [location=510546..512474] [gbkey=CDS]	1929	93.10%	64.80%
meC0281	[locus_tag=DQM41_RS02720]	lcl|NZ_LS483362.1_cds_WP_002864504.1_525	412	1410	999	reverse	412	1410	999	412	1410	[gene=fliD] [protein=flagellar filament capping protein FliD] [protein_id=WP_002864504.1] [location=510546..512474] [gbkey=CDS]	1929	96.10%	99.90%
meC0467	[locus_tag=DQM41_RS02720]	lcl|NZ_LS483362.1_cds_WP_002864504.1_525	43	900	858	reverse	43	900	858	43	900	[gene=fliD] [protein=flagellar filament capping protein FliD] [protein_id=WP_002864504.1] [location=510546..512474] [gbkey=CDS]	1929	99.00%	85.80%
meC0280	[locus_tag=DQM41_RS05225]	lcl|NZ_LS483362.1_cds_WP_010891903.1_1007	121	1119	999	reverse	121	1119	999	121	1119	[protein=endonuclease MutS2] [protein_id=WP_010891903.1] [location=complement(987001..989211)] [gbkey=CDS]	2211	99.40%	99.90%
meC0281	[locus_tag=DQM41_RS05225]	lcl|NZ_LS483362.1_cds_WP_010891903.1_1007	685	1500	816	forward	685	1500	816	685	1500	[protein=endonuclease MutS2] [protein_id=WP_010891903.1] [location=complement(987001..989211)] [gbkey=CDS]	2211	100.00%	81.60%
meC0467	[locus_tag=DQM41_RS05225]	lcl|NZ_LS483362.1_cds_WP_010891903.1_1007	577	1500	924	forward	577	1500	924	577	1500	[protein=endonuclease MutS2] [protein_id=WP_010891903.1] [location=complement(987001..989211)] [gbkey=CDS]	2211	99.40%	92.40%
meC0280	[locus_tag=DQM41_RS05130]	lcl|NZ_LS483362.1_cds_WP_010891901.1_988	781	1779	999	forward	781	1779	999	781	1779	[gene=cmeF] [protein=multidrug efflux RND transporter permease subunit CmeF] [protein_id=WP_010891901.1] [location=965475..968492] [gbkey=CDS]	3018	91.40%	99.90%
meC0280	[locus_tag=DQM41_RS05130]	lcl|NZ_LS483362.1_cds_WP_010891901.1_988	1780	2778	999	forward	1780	2778	999	1780	2778	[gene=cmeF] [protein=multidrug efflux RND transporter permease subunit CmeF] [protein_id=WP_010891901.1] [location=965475..968492] [gbkey=CDS]	3018	96.30%	99.90%
meC0281	[locus_tag=DQM41_RS05130]	lcl|NZ_LS483362.1_cds_WP_010891901.1_988	2217	2615	399	reverse	2217	2615	399	2217	2615	[gene=cmeF] [protein=multidrug efflux RND transporter permease subunit CmeF] [protein_id=WP_010891901.1] [location=965475..968492] [gbkey=CDS]	3018	100.00%	39.90%
meC0281	[locus_tag=DQM41_RS05130]	lcl|NZ_LS483362.1_cds_WP_010891901.1_988	1216	2214	999	reverse	1216	2214	999	1216	2214	[gene=cmeF] [protein=multidrug efflux RND transporter permease subunit CmeF] [protein_id=WP_010891901.1] [location=965475..968492] [gbkey=CDS]	3018	98.50%	99.90%
meC0281	[locus_tag=DQM41_RS05130]	lcl|NZ_LS483362.1_cds_WP_010891901.1_988	217	1173	957	reverse	217	1173	957	217	1173	[gene=cmeF] [protein=multidrug efflux RND transporter permease subunit CmeF] [protein_id=WP_010891901.1] [location=965475..968492] [gbkey=CDS]	3018	98.10%	95.70%
meC0467	[locus_tag=DQM41_RS05130]	lcl|NZ_LS483362.1_cds_WP_010891901.1_988	1237	2232	996	reverse	1237	2232	996	1237	2232	[gene=cmeF] [protein=multidrug efflux RND transporter permease subunit CmeF] [protein_id=WP_010891901.1] [location=965475..968492] [gbkey=CDS]	3018	99.40%	99.60%
meC0467	[locus_tag=DQM41_RS05130]	lcl|NZ_LS483362.1_cds_WP_010891901.1_988	235	1173	939	reverse	235	1173	939	235	1173	[gene=cmeF] [protein=multidrug efflux RND transporter permease subunit CmeF] [protein_id=WP_010891901.1] [location=965475..968492] [gbkey=CDS]	3018	96.20%	93.90%
meC0280	[locus_tag=DQM41_RS05125]	lcl|NZ_LS483362.1_cds_WP_002858463.1_987	1	522	522	forward	1	522	522	1	522	[gene=cmeE] [protein=multidrug efflux RND transporter periplasmic adaptor subunit CmeE] [protein_id=WP_002858463.1] [location=964732..965472] [gbkey=CDS]	741	92.30%	52.20%
meC0281	[locus_tag=DQM41_RS05125]	lcl|NZ_LS483362.1_cds_WP_002858463.1_987	1	741	741	reverse	1	741	741	1	741	[gene=cmeE] [protein=multidrug efflux RND transporter periplasmic adaptor subunit CmeE] [protein_id=WP_002858463.1] [location=964732..965472] [gbkey=CDS]	741	97.60%	74.10%
meC0467	[locus_tag=DQM41_RS05125]	lcl|NZ_LS483362.1_cds_WP_002858463.1_987	1	741	741	reverse	1	741	741	1	741	[gene=cmeE] [protein=multidrug efflux RND transporter periplasmic adaptor subunit CmeE] [protein_id=WP_002858463.1] [location=964732..965472] [gbkey=CDS]	741	98.40%	74.10%
meC0280	[locus_tag=DQM41_RS05115]	lcl|NZ_LS483362.1_cds_WP_002837927.1_985	235	999	765	reverse	235	999	765	235	999	[gene=lepA] [protein=elongation factor 4] [protein_id=WP_002837927.1] [location=complement(961538..963334)] [gbkey=CDS]	1797	97.30%	76.50%
meC0281	[locus_tag=DQM41_RS05115]	lcl|NZ_LS483362.1_cds_WP_002837927.1_985	811	1647	837	forward	811	1647	837	811	1647	[gene=lepA][protein=elongation factor 4] [protein_id=WP_002837927.1] [location=complement(961538..963334)] [gbkey=CDS]	1797	95.20%	83.70%
meC0467	[locus_tag=DQM41_RS05115]	lcl|NZ_LS483362.1_cds_WP_002837927.1_985	1	627	627	forward	1	627	627	1	627	[gene=lepA] [protein=elongation factor 4] [protein_id=WP_002837927.1] [location=complement(961538..963334)] [gbkey=CDS]	1797	94.50%	62.70%
meC0280	[locus_tag=DQM41_RS00120]	lcl|NZ_LS483362.1_cds_WP_002865087.1_24	1	564	564	forward	1	564	564	1	564	[protein=ribonucleoside-diphosphate reductase subunit alpha] [protein_id=WP_002865087.1] [location=29726..32095] [gbkey=CDS]	2370	97.90%	56.40%
meC0281	[locus_tag=DQM41_RS00120]	lcl|NZ_LS483362.1_cds_WP_002865087.1_24	904	1902	999	forward	904	1902	999	904	1902	[protein=ribonucleoside-diphosphate reductase subunit alpha] [protein_id=WP_002865087.1] [location=29726..32095] [gbkey=CDS]	2370	97.90%	99.90%
meC0281	[locus_tag=DQM41_RS00120]	lcl|NZ_LS483362.1_cds_WP_002865087.1_24	1906	2370	465	forward	1906	2370	465	1906	2370	[protein=ribonucleoside-diphosphate reductase subunit alpha] [protein_id=WP_002865087.1] [location=29726..32095] [gbkey=CDS]	2370	98.70%	46.50%
meC0467	[locus_tag=DQM41_RS00120]	lcl|NZ_LS483362.1_cds_WP_002865087.1_24	1552	2370	819	forward	1552	2370	819	1552	2370	[protein=ribonucleoside-diphosphate reductase subunit alpha] [protein_id=WP_002865087.1] [location=29726..32095] [gbkey=CDS]	2370	97.80%	54.90%
meC0467	[locus_tag=DQM41_RS00120]	lcl|NZ_LS483362.1_cds_WP_002865087.1_24	1	549	549	forward	1	549	549	1	549	[protein=ribonucleoside-diphosphate reductase subunit alpha] [protein_id=WP_002865087.1] [location=29726..32095] [gbkey=CDS]	2370	94.90%	81.90%
meC0280	[locus_tag=DQM41_RS03080]	lcl|NZ_LS483362.1_cds_WP_010891867.1_594	1	564	564	forward	1	564	564	1	564	[protein=phosphate ABC transporter ATP-binding protein] [protein_id=WP_010891867.1] [location=576216..576956] [gbkey=CDS]	741	99.50%	56.40%
meC0281	[locus_tag=DQM41_RS03080]	lcl|NZ_LS483362.1_cds_WP_010891867.1_594	52	741	690	reverse	52	741	690	52	741	[protein=phosphate ABC transporter ATP-binding protein] [protein_id=WP_010891867.1] [location=576216..576956] [gbkey=CDS]	741	99.60%	69.00%
meC0467	[locus_tag=DQM41_RS03080]	lcl|NZ_LS483362.1_cds_WP_010891867.1_594	1	579	579	reverse	1	579	579	1	579	[protein=phosphate ABC transporter ATP-binding protein] [protein_id=WP_010891867.1] [location=576216..576956] [gbkey=CDS]	741	99.50%	57.90%
meC0280	[locus_tag=DQM41_RS02420]	lcl|NZ_LS483362.1_cds_WP_002864166.1_466	3	452	450	forward	3	452	450	3	452	[gene=fucP] [protein=L-fucose:H+ symporter permease] [protein_id=WP_002864166.1] [location=453113..454369] [gbkey=CDS]	1257	90.40%	45.00%
meC0280	[locus_tag=DQM41_RS02420]	lcl|NZ_LS483362.1_cds_WP_002864166.1_466	676	1257	582	forward	676	1257	582	676	1257	[gene=fucP] [protein=L-fucose:H+ symporter permease] [protein_id=WP_002864166.1] [location=453113..454369] [gbkey=CDS]	1257	100.00%	58.20%
meC0281	[locus_tag=DQM41_RS02420]	lcl|NZ_LS483362.1_cds_WP_002864166.1_466	1	561	561	reverse	1	561	561	1	561	[gene=fucP] [protein=L-fucose:H+ symporter permease] [protein_id=WP_002864166.1] [location=453113..454369] [gbkey=CDS]	1257	89.10%	56.10%
meC0467	[locus_tag=DQM41_RS02420]	lcl|NZ_LS483362.1_cds_WP_002864166.1_466	619	1236	618	reverse	619	1236	618	619	1236	[gene=fucP] [protein=L-fucose:H+ symporter permease] [protein_id=WP_002864166.1] [location=453113..454369] [gbkey=CDS]	1257	92.20%	61.80%
meC0280	[locus_tag=DQM41_RS03750]	lcl|NZ_LS483362.1_cds_WP_010891883.1_723	1021	2019	999	forward	1021	2019	999	1021	2019	[gene=cfrA][protein=TonB-dependent ferric enterobactin receptor CfrA] [protein_id=WP_010891883.1] [location=705435..707525] [gbkey=CDS]	2091	94.90%	99.90%
meC0281	[locus_tag=DQM41_RS03750]	lcl|NZ_LS483362.1_cds_WP_010891883.1_723	841	1836	996	reverse	841	1836	996	841	1836	[gene=cfrA] [protein=TonB-dependent ferric enterobactin receptor CfrA] [protein_id=WP_010891883.1] [location=705435..707525] [gbkey=CDS]	2091	99.70%	99.60%
meC0467	[locus_tag=DQM41_RS03750]	lcl|NZ_LS483362.1_cds_WP_010891883.1_723	268	1266	999	reverse	268	1266	999	268	1266	[gene=cfrA] [protein=TonB-dependent ferric enterobactin receptor CfrA] [protein_id=WP_010891883.1] [location=705435..707525] [gbkey=CDS]	2091	100.00%	99.90%
meC0280	[locus_tag=DQM41_RS06490]	lcl|NZ_LS483362.1_cds_WP_002858432.1_1257	733	1221	489	reverse	733	1221	489	733	1221	[protein=DUF2920 family protein] [protein_id=WP_002858432.1] [location=complement(1235506..1236732)] [gbkey=CDS]	1227	89.00%	48.90%
meC0281	[locus_tag=DQM41_RS06490]	lcl|NZ_LS483362.1_cds_WP_002858432.1_1257	1	636	636	forward	1	636	636	1	636	[protein=DUF2920 family protein] [protein_id=WP_002858432.1] [location=complement(1235506..1236732)] [gbkey=CDS]	1227	95.50%	63.60%
meC0281	[locus_tag=DQM41_RS06490]	lcl|NZ_LS483362.1_cds_WP_002858432.1_1257	637	1227	591	forward	637	1227	591	637	1227	[protein=DUF2920 family protein] [protein_id=WP_002858432.1] [location=complement(1235506..1236732)] [gbkey=CDS]	1227	99.50%	59.10%
meC0281	[locus_tag=DQM41_RS06490]	lcl|NZ_LS483362.1_cds_WP_002858432.1_1257	733	1221	489	forward	733	1221	489	733	1221	[protein=DUF2920 family protein] [protein_id=WP_002858432.1] [location=complement(1235506..1236732)] [gbkey=CDS]	1227	86.50%	48.90%
meC0467	[locus_tag=DQM41_RS06490]	lcl|NZ_LS483362.1_cds_WP_002858432.1_1257	733	1221	489	forward	733	1221	489	733	1221	[protein=DUF2920 family protein] [protein_id=WP_002858432.1] [location=complement(1235506..1236732)] [gbkey=CDS]	1227	87.70%	48.90%
meC0280	[locus_tag=DQM41_RS08350]	lcl|NZ_LS483362.1_cds_WP_002851268.1_1617	598	1596	999	reverse	598	1596	999	598	1596	[protein=type I DNA topoisomerase] [protein_id=WP_002851268.1] [location=complement(1607343..1609445)] [gbkey=CDS]	2103	96.10%	99.90%
meC0281	[locus_tag=DQM41_RS08350]	lcl|NZ_LS483362.1_cds_WP_002851268.1_1617	169	1167	999	reverse	169	1167	999	169	1167	[protein=type I DNA topoisomerase] [protein_id=WP_002851268.1] [location=complement(1607343..1609445)] [gbkey=CDS]	2103	93.90%	99.90%
meC0467	[locus_tag=DQM41_RS08350]	lcl|NZ_LS483362.1_cds_WP_002851268.1_1617	1	912	912	reverse	1	912	912	1	912	[protein=type I DNA topoisomerase] [protein_id=WP_002851268.1] [location=complement(1607343..1609445)] [gbkey=CDS]	2103	97.00%	91.20%
meC0280	[locus_tag=DQM41_RS00190]	lcl|NZ_LS483362.1_cds_WP_002866314.1_33	64	690	627	reverse	64	690	627	64	690	[protein=SIMPL domain-containing protein] [protein_id=WP_002866314.1] [location=complement(51963..52664)] [gbkey=CDS]	702	90.40%	62.70%
meC0281	[locus_tag=DQM41_RS00190]	lcl|NZ_LS483362.1_cds_WP_002866314.1_33	64	543	480	reverse	64	543	480	64	543	[protein=SIMPL domain-containing protein] [protein_id=WP_002866314.1] [location=complement(51963..52664)] [gbkey=CDS]	702	93.10%	48.00%
meC0467	[locus_tag=DQM41_RS00190]	lcl|NZ_LS483362.1_cds_WP_002866314.1_33	64	702	639	reverse	64	702	639	64	702	[protein=SIMPL domain-containing protein] [protein_id=WP_002866314.1] [location=complement(51963..52664)] [gbkey=CDS]	702	90.60%	63.90%

Table S8: The 23 common genes with putative *C. jejuni* origin based on the meC0280 data set.

locus_tag	Sequence Name	Minimum	Maximum	Length	Direction	Min (with gaps)	Max (with gaps)	Length (with gaps)	Min (original sequence)	Max (original sequence)	Description	Sequence Length	% Pairwise Identity	Query Coverage
DQM41_RS01430	lcl|NZ_LS483362.1_cds_WP_002778123.1_278	447	848	402	reverse	447	848	402	447	848	[protein=pantoate--beta-alanine ligase] [protein_id=WP_002778123.1] [location=complement(271412..272260)] [gbkey=CDS]	849	99.30%	40.20%
DQM41_RS03100	lcl|NZ_LS483362.1_cds_WP_002826526.1_598	466	1461	996	forward	466	1461	996	466	1461	[protein=hypothetical protein] [protein_id=WP_002826526.1] [location=580316..581836] [gbkey=CDS]	1521	91.50%	99.60%
DQM41_RS08350	lcl|NZ_LS483362.1_cds_WP_002851268.1_1617	598	1596	999	reverse	598	1596	999	598	1596	[protein=type I DNA topoisomerase] [protein_id=WP_002851268.1] [location=complement(1607343..1609445)] [gbkey=CDS]	2103	96.10%	99.90%
DQM41_RS07490	lcl|NZ_LS483362.1_cds_WP_010891941.1_1454	1	717	717	reverse	1	717	717	1	717	[protein=formate dehydrogenase subunit alpha] [transl_except=(pos:541..543,aa:Sec)] [protein_id=WP_010891941.1] [location=complement(1446324..1449128)] [gbkey=CDS]	2805	91.00%	71.70%
DQM41_RS07815	lcl|NZ_LS483362.1_cds_WP_002851210.1_1519	169	1167	999	forward	169	1167	999	169	1167	[protein=FAD-binding oxidoreductase] [protein_id=WP_002851210.1] [location=complement(1514627..1517398)] [gbkey=CDS]	2772	91.30%	99.90%
DQM41_RS07815	lcl|NZ_LS483362.1_cds_WP_002851210.1_1519	1168	2166	999	forward	1168	2166	999	1168	2166	[protein=FAD-binding oxidoreductase] [protein_id=WP_002851210.1] [location=complement(1514627..1517398)] [gbkey=CDS]	2772	98.50%	99.90%
DQM41_RS05220	lcl|NZ_LS483362.1_cds_WP_002864285.1_1006	1903	2628	726	reverse	1903	2628	726	1903	2628	[protein=N-6 DNA methylase] [protein_id=WP_002864285.1] [location=complement(982973..986992)] [gbkey=CDS]	4020	94.20%	72.60%
DQM41_RS05220	lcl|NZ_LS483362.1_cds_WP_002864285.1_1006	901	1899	999	reverse	901	1899	999	901	1899	[protein=N-6 DNA methylase] [protein_id=WP_002864285.1] [location=complement(982973..986992)] [gbkey=CDS]	4020	100.00%	99.90%
DQM41_RS05220	lcl|NZ_LS483362.1_cds_WP_002864285.1_1006	1	900	900	reverse	1	900	900	1	900	[protein=N-6 DNA methylase] [protein_id=WP_002864285.1] [location=complement(982973..986992)] [gbkey=CDS]	4020	99.00%	90.00%
DQM41_RS03750	lcl|NZ_LS483362.1_cds_WP_010891883.1_723	1021	2019	999	forward	1021	2019	999	1021	2019	[gene=cfrA][protein=TonB-dependent ferric enterobactin receptor CfrA] [protein_id=WP_010891883.1] [location=705435..707525] [gbkey=CDS]	2091	94.90%	99.90%
DQM41_RS05125	lcl|NZ_LS483362.1_cds_WP_002858463.1_987	1	522	522	forward	1	522	522	1	522	[gene=cmeE] [protein=multidrug efflux RND transporter periplasmic adaptor subunit CmeE] [protein_id=WP_002858463.1] [location=964732..965472] [gbkey=CDS]	741	92.30%	52.20%
DQM41_RS00120	lcl|NZ_LS483362.1_cds_WP_002865087.1_24	1	564	564	forward	1	564	564	1	564	[protein=ribonucleoside-diphosphate reductase subunit alpha] [protein_id=WP_002865087.1] [location=29726..32095] [gbkey=CDS]	2370	97.90%	56.40%
DQM41_RS02720	lcl|NZ_LS483362.1_cds_WP_002864504.1_525	43	690	648	forward	43	690	648	43	690	[gene=fliD] [protein=flagellar filament capping protein FliD] [protein_id=WP_002864504.1] [location=510546..512474] [gbkey=CDS]	1929	93.10%	64.80%
DQM41_RS00190	lcl|NZ_LS483362.1_cds_WP_002866314.1_33	64	690	627	reverse	64	690	627	64	690	[protein=SIMPL domain-containing protein] [protein_id=WP_002866314.1] [location=complement(51963..52664)] [gbkey=CDS]	702	90.40%	62.70%
DQM41_RS05225	lcl|NZ_LS483362.1_cds_WP_010891903.1_1007	121	1119	999	reverse	121	1119	999	121	1119	[protein=endonuclease MutS2] [protein_id=WP_010891903.1] [location=complement(987001..989211)] [gbkey=CDS]	2211	99.40%	99.90%
DQM41_RS06490	lcl|NZ_LS483362.1_cds_WP_002858432.1_1257	733	1221	489	reverse	733	1221	489	733	1221	[protein=DUF2920 family protein] [protein_id=WP_002858432.1] [location=complement(1235506..1236732)] [gbkey=CDS]	1227	89.00%	48.90%
DQM41_RS01770	lcl|NZ_LS483362.1_cds_WP_002872052.1_346	1080	2063	984	reverse	1080	2063	984	1080	2063	[gene=cmeB] [protein=multidrug efflux RND transporter permease subunit CmeB] [protein_id=WP_002872052.1] [location=complement(332589..335711)] [gbkey=CDS]	3123	92.90%	98.40%
DQM41_RS03080	lcl|NZ_LS483362.1_cds_WP_010891867.1_594	1	564	564	forward	1	564	564	1	564	[protein=phosphate ABC transporter ATP-binding protein] [protein_id=WP_010891867.1] [location=576216..576956] [gbkey=CDS]	741	99.50%	56.40%
DQM41_RS05130	lcl|NZ_LS483362.1_cds_WP_010891901.1_988	781	1779	999	forward	781	1779	999	781	1779	[gene=cmeF] [protein=multidrug efflux RND transporter permease subunit CmeF] [protein_id=WP_010891901.1] [location=965475..968492] [gbkey=CDS]	3018	91.40%	99.90%
DQM41_RS05130	lcl|NZ_LS483362.1_cds_WP_010891901.1_988	1780	2778	999	forward	1780	2778	999	1780	2778	[gene=cmeF] [protein=multidrug efflux RND transporter permease subunit CmeF] [protein_id=WP_010891901.1] [location=965475..968492] [gbkey=CDS]	3018	96.30%	99.90%
DQM41_RS02380	lcl|NZ_LS483362.1_cds_WP_010891860.1_458	1810	2808	999	forward	1810	2808	999	1810	2808	[gene=rpoB] [protein=DNA-directed RNA polymerase subunit beta] [protein_id=WP_010891860.1] [location=438986..443122] [gbkey=CDS]	4137	99.70%	99.90%
DQM41_RS03640	lcl|NZ_LS483362.1_cds_WP_002793291.1_706	484	1326	843	forward	484	1326	843	484	1326	[protein=esterase-like activity of phytase family protein] [protein_id=WP_002793291.1] [location=682515..683840] [gbkey=CDS]	1326	98.60%	84.30%
DQM41_RS05115	lcl|NZ_LS483362.1_cds_WP_002837927.1_985	235	999	765	reverse	235	999	765	235	999	[gene=lepA] [protein=elongation factor 4] [protein_id=WP_002837927.1] [location=complement(961538..963334)] [gbkey=CDS]	1797	97.30%	76.50%
DQM41_RS02420	lcl|NZ_LS483362.1_cds_WP_002864166.1_466	3	452	450	forward	3	452	450	3	452	[gene=fucP] [protein=L-fucose:H+ symporter permease] [protein_id=WP_002864166.1] [location=453113..454369] [gbkey=CDS]	1257	90.40%	45.00%
DQM41_RS02420	lcl|NZ_LS483362.1_cds_WP_002864166.1_466	676	1257	582	forward	676	1257	582	676	1257	[gene=fucP] [protein=L-fucose:H+ symporter permease] [protein_id=WP_002864166.1] [location=453113..454369] [gbkey=CDS]	1257	100.00%	58.20%
DQM41_RS06505	lcl|NZ_LS483362.1_cds_WP_002856137.1_1260	586	1068	483	reverse	586	1068	483	586	1068	[protein=hypothetical protein] [protein_id=WP_002856137.1] [location=complement(1238565..1239635)] [gbkey=CDS]	1071	100.00%	48.30%
DQM41_RS04280	lcl|NZ_LS483362.1_cds_WP_002864843.1_829	1441	1509	69	forward	1441	1509	69	1441	1509	[protein=chorismate-binding protein] [protein_id=WP_002864843.1] [location=complement(807480..809264)] [gbkey=CDS]	1785	47.80%	6.90%
DQM41_RS05910	lcl|NZ_LS483362.1_cds_WP_002852850.1_1144	46	519	474	forward	46	519	474	46	519	[protein=cation:dicarboxylase symporter family transporter] [protein_id=WP_002852850.1] [location=1119951..1121327] [gbkey=CDS]	1377	98.70%	47.40%

Table S9: The 23 common genes with putative C. jejuni origin based on the meC0281 data set.

locus_tag	Sequence Name	Minimum	Maximum	Length	Direction	Min (with gaps)	Max (with gaps)	Length (with gaps)	Min (original sequence)	Max (original sequence)	Description	Sequence Length	% Pairwise Identity	Query Coverage
DQM41_RS01430	lcl|NZ_LS483362.1_cds_WP_002778123.1_278	405	848	444	forward	405	848	444	405	848	[protein=pantoate--beta-alanine ligase] [protein_id=WP_002778123.1] [location=complement(271412..272260)] [gbkey=CDS]	849	99.30 %	44.40 %
DQM41_RS03100	lcl|NZ_LS483362.1_cds_WP_002826526.1_598	1	948	948	reverse	1	948	948	1	948	[protein=hypothetical protein] [protein_id=WP_002826526.1] [location=580316..581836] [gbkey=CDS]	1521	98.70 %	94.80 %
DQM41_RS08350	lcl|NZ_LS483362.1_cds_WP_002851268.1_1617	169	1167	999	reverse	169	1167	999	169	1167	[protein=type I DNA topoisomerase] [protein_id=WP_002851268.1] [location=complement(1607343..1609445)] [gbkey=CDS]	2103	93.90 %	99.90 %
DQM41_RS07490	lcl|NZ_LS483362.1_cds_WP_010891941.1_1454	847	1422	576	forward	847	1422	576	847	1422	[protein=formate dehydrogenase subunit alpha] [transl_except=(pos:541..543,aa:Sec)] [protein_id=WP_010891941.1] [location=complement(1446324..1449128)] [gbkey=CDS]	2805	97.40 %	57.60 %
DQM41_RS07815	lcl|NZ_LS483362.1_cds_WP_002851210.1_1519	325	1323	999	forward	325	1323	999	325	1323	[protein=FAD-binding oxidoreductase] [protein_id=WP_002851210.1] [location=complement(1514627..1517398)] [gbkey=CDS]	2772	95.10 %	99.90 %
DQM41_RS05220	lcl|NZ_LS483362.1_cds_WP_002864285.1_1006	466	1464	999	forward	466	1464	999	466	1464	[protein=N-6 DNA methylase] [protein_id=WP_002864285.1] [location=complement(982973..986992)] [gbkey=CDS]	4020	99.70 %	99.90 %
DQM41_RS05220	lcl|NZ_LS483362.1_cds_WP_002864285.1_1006	1465	2463	999	forward	1465	2463	999	1465	2463	[protein=N-6 DNA methylase] [protein_id=WP_002864285.1] [location=complement(982973..986992)] [gbkey=CDS]	4020	98.20 %	99.90 %
DQM41_RS05220	lcl|NZ_LS483362.1_cds_WP_002864285.1_1006	3391	4020	630	forward	3391	4020	630	3391	4020	[protein=N-6 DNA methylase] [protein_id=WP_002864285.1] [location=complement(982973..986992)] [gbkey=CDS]	4020	93.60 %	63.00 %
DQM41_RS03750	lcl|NZ_LS483362.1_cds_WP_010891883.1_723	841	1836	996	reverse	841	1836	996	841	1836	[gene=cfrA] [protein=TonB-dependent ferric enterobactin receptor CfrA] [protein_id=WP_010891883.1] [location=705435..707525] [gbkey=CDS]	2091	99.70 %	99.60 %
DQM41_RS05125	lcl|NZ_LS483362.1_cds_WP_002858463.1_987	1	741	741	reverse	1	741	741	1	741	[gene=cmeE] [protein=multidrug efflux RND transporter periplasmic adaptor subunit CmeE] [protein_id=WP_002858463.1] [location=964732..965472] [gbkey=CDS]	741	97.60 %	74.10 %
DQM41_RS00120	lcl|NZ_LS483362.1_cds_WP_002865087.1_24	904	1902	999	forward	904	1902	999	904	1902	[protein=ribonucleoside-diphosphate reductase subunit alpha] [protein_id=WP_002865087.1] [location=29726..32095] [gbkey=CDS]	2370	97.90 %	99.90 %
DQM41_RS00120	lcl|NZ_LS483362.1_cds_WP_002865087.1_24	1906	2370	465	forward	1906	2370	465	1906	2370	[protein=ribonucleoside-diphosphate reductase subunit alpha] [protein_id=WP_002865087.1] [location=29726..32095] [gbkey=CDS]	2370	98.70 %	46.50 %
DQM41_RS02720	lcl|NZ_LS483362.1_cds_WP_002864504.1_525	412	1410	999	reverse	412	1410	999	412	1410	[gene=fliD] [protein=flagellar filament capping protein FliD] [protein_id=WP_002864504.1] [location=510546..512474] [gbkey=CDS]	1929	96.10 %	99.90 %
DQM41_RS00190	lcl|NZ_LS483362.1_cds_WP_002866314.1_33	64	543	480	reverse	64	543	480	64	543	[protein=SIMPL domain-containing protein] [protein_id=WP_002866314.1] [location=complement(51963..52664)] [gbkey=CDS]	702	93.10 %	48.00 %
DQM41_RS05225	lcl|NZ_LS483362.1_cds_WP_010891903.1_1007	685	1500	816	forward	685	1500	816	685	1500	[protein=endonuclease MutS2] [protein_id=WP_010891903.1] [location=complement(987001..989211)] [gbkey=CDS]	2211	100.00 %	81.60 %
DQM41_RS06490	lcl|NZ_LS483362.1_cds_WP_002858432.1_1257	1	636	636	forward	1	636	636	1	636	[protein=DUF2920 family protein] [protein_id=WP_002858432.1] [location=complement(1235506..1236732)] [gbkey=CDS]	1227	95.50 %	63.60 %
DQM41_RS06490	lcl|NZ_LS483362.1_cds_WP_002858432.1_1257	637	1227	591	forward	637	1227	591	637	1227	[protein=DUF2920 family protein] [protein_id=WP_002858432.1] [location=complement(1235506..1236732)] [gbkey=CDS]	1227	99.50 %	59.10 %
DQM41_RS06490	lcl|NZ_LS483362.1_cds_WP_002858432.1_1257	733	1221	489	forward	733	1221	489	733	1221	[protein=DUF2920 family protein] [protein_id=WP_002858432.1] [location=complement(1235506..1236732)] [gbkey=CDS]	1227	86.50 %	48.90 %
DQM41_RS01770	lcl|NZ_LS483362.1_cds_WP_002872052.1_346	85	1044	960	forward	85	1044	960	85	1044	[gene=cmeB] [protein=multidrug efflux RND transporter permease subunit CmeB] [protein_id=WP_002872052.1] [location=complement(332589..335711)] [gbkey=CDS]	3123	95.20 %	96.00 %
DQM41_RS01770	lcl|NZ_LS483362.1_cds_WP_002872052.1_346	1048	2046	999	forward	1048	2046	999	1048	2046	[gene=cmeB][protein=multidrug efflux RND transporter permease subunit CmeB] [protein_id=WP_002872052.1] [location=complement(332589..335711)] [gbkey=CDS]	3123	86.40 %	99.90 %
DQM41_RS03080	lcl|NZ_LS483362.1_cds_WP_010891867.1_594	52	741	690	reverse	52	741	690	52	741	[protein=phosphate ABC transporter ATP-binding protein] [protein_id=WP_010891867.1] [location=576216..576956] [gbkey=CDS]	741	99.60 %	69.00 %
DQM41_RS05130	lcl|NZ_LS483362.1_cds_WP_010891901.1_988	2217	2615	399	reverse	2217	2615	399	2217	2615	[gene=cmeF] [protein=multidrug efflux RND transporter permease subunit CmeF] [protein_id=WP_010891901.1] [location=965475..968492] [gbkey=CDS]	3018	100.00 %	39.90 %
DQM41_RS05130	lcl|NZ_LS483362.1_cds_WP_010891901.1_988	1216	2214	999	reverse	1216	2214	999	1216	2214	[gene=cmeF] [protein=multidrug efflux RND transporter permease subunit CmeF] [protein_id=WP_010891901.1] [location=965475..968492] [gbkey=CDS]	3018	98.50 %	99.90 %
DQM41_RS05130	lcl|NZ_LS483362.1_cds_WP_010891901.1_988	217	1173	957	reverse	217	1173	957	217	1173	[gene=cmeF] [protein=multidrug efflux RND transporter permease subunit CmeF] [protein_id=WP_010891901.1] [location=965475..968492] [gbkey=CDS]	3018	98.10 %	95.70 %
DQM41_RS02380	lcl|NZ_LS483362.1_cds_WP_010891860.1_458	2131	3129	999	reverse	2131	3129	999	2131	3129	[gene=rpoB] [protein=DNA-directed RNA polymerase subunit beta] [protein_id=WP_010891860.1] [location=438986..443122] [gbkey=CDS]	4137	99.70 %	99.90 %
DQM41_RS02380	lcl|NZ_LS483362.1_cds_WP_010891860.1_458	1132	2127	996	reverse	1132	2127	996	1132	2127	[gene=rpoB] [protein=DNA-directed RNA polymerase subunit beta] [protein_id=WP_010891860.1] [location=438986..443122] [gbkey=CDS]	4137	98.00 %	99.60 %
DQM41_RS03640	lcl|NZ_LS483362.1_cds_WP_002793291.1_706	1	948	948	reverse	1	948	948	1	948	[protein=esterase-like activity of phytase family protein] [protein_id=WP_002793291.1] [location=682515..683840] [gbkey=CDS]	1326	99.40 %	94.80 %
DQM41_RS05115	lcl|NZ_LS483362.1_cds_WP_002837927.1_985	811	1647	837	forward	811	1647	837	811	1647	[gene=lepA][protein=elongation factor 4] [protein_id=WP_002837927.1] [location=complement(961538..963334)] [gbkey=CDS]	1797	95.20 %	83.70 %
DQM41_RS02420	lcl|NZ_LS483362.1_cds_WP_002864166.1_466	1	561	561	reverse	1	561	561	1	561	[gene=fucP] [protein=L-fucose:H+ symporter permease] [protein_id=WP_002864166.1] [location=453113..454369] [gbkey=CDS]	1257	89.10 %	56.10 %
DQM41_RS06505	lcl|NZ_LS483362.1_cds_WP_002856137.1_1260	280	936	657	forward	280	936	657	280	936	[protein=hypothetical protein] [protein_id=WP_002856137.1] [location=complement(1238565..1239635)] [gbkey=CDS]	1071	99.10 %	65.70 %
DQM41_RS04280	lcl|NZ_LS483362.1_cds_WP_002864843.1_829	1441	1509	69	forward	1441	1509	69	1441	1509	[protein=chorismate-binding protein] [protein_id=WP_002864843.1] [location=complement(807480..809264)] [gbkey=CDS]	1785	47.80 %	6.90 %
DQM41_RS04280	lcl|NZ_LS483362.1_cds_WP_002864843.1_829	1441	1509	69	reverse	1441	1509	69	1441	1509	[protein=chorismate-binding protein] [protein_id=WP_002864843.1] [location=complement(807480..809264)] [gbkey=CDS]	1785	47.80 %	6.90 %
DQM41_RS04280	lcl|NZ_LS483362.1_cds_WP_002864843.1_829	1441	1509	69	reverse	1441	1509	69	1441	1509	[protein=chorismate-binding protein] [protein_id=WP_002864843.1] [location=complement(807480..809264)] [gbkey=CDS]	1785	47.80 %	6.90 %
DQM41_RS05910	lcl|NZ_LS483362.1_cds_WP_002852850.1_1144	817	1377	561	reverse	817	1377	561	817	1377	[protein=cation:dicarboxylase symporter family transporter] [protein_id=WP_002852850.1] [location=1119951..1121327] [gbkey=CDS]	1377	100.00 %	56.10 %
DQM41_RS05910	lcl|NZ_LS483362.1_cds_WP_002852850.1_1144	46	813	768	reverse	46	813	768	46	813	[protein=cation:dicarboxylase symporter family transporter] [protein_id=WP_002852850.1] [location=1119951..1121327] [gbkey=CDS]	1377	94.70 %	76.80 %

Table S10: The 23 common genes with putative *C. jejuni* origin based on the meC0467 data set.

<table>
<tr><th>locus_tag</th><th>Sequence Name</th><th>Minimum</th><th>Maximum</th><th>Length</th><th>Direction</th><th>Min (with gaps)</th><th>Max (with gaps)</th><th>Length (with gaps)</th><th>Min (original sequence)</th><th>Max (original sequence)</th><th>Description</th><th>Sequence Length</th><th>% Pairwise Identity</th><th>Query Coverage</th></tr>
<tr><td>DQM41_RS01430</td><td>lcl|NZ_LS483362.1_cds_WP_002778123.1_278</td><td>3</td><td>728</td><td>726</td><td>forward</td><td>3</td><td>728</td><td>726</td><td>3</td><td>728</td><td>[protein=pantoate--beta-alanine ligase] [protein_id=WP_002778123.1] [location=complement(271412..272260)] [gbkey=CDS]</td><td>849</td><td>99.20 %</td><td>72.60 %</td></tr>
<tr><td>DQM41_RS03100</td><td>lcl|NZ_LS483362.1_cds_WP_002826526.1_598</td><td>343</td><td>1338</td><td>996</td><td>reverse</td><td>343</td><td>1338</td><td>996</td><td>343</td><td>1338</td><td>[protein=hypothetical protein] [protein_id=WP_002826526.1] [location=580316..581836] [gbkey=CDS]</td><td>1521</td><td>91.60 %</td><td>99.60 %</td></tr>
<tr><td>DQM41_RS08350</td><td>lcl|NZ_LS483362.1_cds_WP_002851268.1_1617</td><td>1</td><td>912</td><td>912</td><td>reverse</td><td>1</td><td>912</td><td>912</td><td>1</td><td>912</td><td>[protein=type I DNA topoisomerase] [protein_id=WP_002851268.1] [location=complement(1607343..1609445)] [gbkey=CDS]</td><td>2103</td><td>97.00 %</td><td>91.20 %</td></tr>
<tr><td>DQM41_RS07490</td><td>lcl|NZ_LS483362.1_cds_WP_010891941.1_1454</td><td>1471</td><td>2157</td><td>687</td><td>forward</td><td>1471</td><td>2157</td><td>687</td><td>1471</td><td>2157</td><td>[protein=formate dehydrogenase subunit alpha] [transl_except=(pos:541..543,aa:Sec)] [protein_id=WP_010891941.1] [location=complement(1446324..1449128)] [gbkey=CDS]</td><td>2805</td><td>95.20 %</td><td>68.70 %</td></tr>
<tr><td>DQM41_RS07815</td><td>lcl|NZ_LS483362.1_cds_WP_002851210.1_1519</td><td>1</td><td>399</td><td>399</td><td>forward</td><td>1</td><td>399</td><td>399</td><td>1</td><td>399</td><td>[protein=FAD-binding oxidoreductase] [protein_id=WP_002851210.1] [location=complement(1514627..1517398)] [gbkey=CDS]</td><td>2772</td><td>89.90 %</td><td>39.90 %</td></tr>
<tr><td>DQM41_RS05220</td><td>lcl|NZ_LS483362.1_cds_WP_002864285.1_1006</td><td>2359</td><td>3354</td><td>996</td><td>forward</td><td>2359</td><td>3354</td><td>996</td><td>2359</td><td>3354</td><td>[protein=N-6 DNA methylase] [protein_id=WP_002864285.1] [location=complement(982973..986992)] [gbkey=CDS]</td><td>4020</td><td>100.00 %</td><td>99.90 %</td></tr>
<tr><td>DQM41_RS05220</td><td>lcl|NZ_LS483362.1_cds_WP_002864285.1_1006</td><td>1357</td><td>2355</td><td>999</td><td>forward</td><td>1357</td><td>2355</td><td>999</td><td>1357</td><td>2355</td><td>[protein=N-6 DNA methylase] [protein_id=WP_002864285.1] [location=complement(982973..986992)] [gbkey=CDS]</td><td>4020</td><td>98.80 %</td><td>99.90 %</td></tr>
<tr><td>DQM41_RS05220</td><td>lcl|NZ_LS483362.1_cds_WP_002864285.1_1006</td><td>358</td><td>1356</td><td>999</td><td>forward</td><td>358</td><td>1356</td><td>999</td><td>358</td><td>1356</td><td>[protein=N-6 DNA methylase] [protein_id=WP_002864285.1] [location=complement(982973..986992)] [gbkey=CDS]</td><td>4020</td><td>98.50 %</td><td>99.60 %</td></tr>
<tr><td>DQM41_RS03750</td><td>lcl|NZ_LS483362.1_cds_WP_010891883.1_723</td><td>268</td><td>1266</td><td>999</td><td>reverse</td><td>268</td><td>1266</td><td>999</td><td>268</td><td>1266</td><td>[gene=cfrA] [protein=TonB-dependent ferric enterobactin receptor CfrA] [protein_id=WP_010891883.1] [location=705435..707525] [gbkey=CDS]</td><td>2091</td><td>100.00 %</td><td>99.90 %</td></tr>
<tr><td>DQM41_RS05125</td><td>lcl|NZ_LS483362.1_cds_WP_002858463.1_987</td><td>1</td><td>741</td><td>741</td><td>reverse</td><td>1</td><td>741</td><td>741</td><td>1</td><td>741</td><td>[gene=cmeE] [protein=multidrug efflux RND transporter periplasmic adaptor subunit CmeE] [protein_id=WP_002858463.1] [location=964732..965472] [gbkey=CDS]</td><td>741</td><td>98.40 %</td><td>74.10 %</td></tr>
<tr><td>DQM41_RS00120</td><td>lcl|NZ_LS483362.1_cds_WP_002865087.1_24</td><td>1552</td><td>2370</td><td>819</td><td>forward</td><td>1552</td><td>2370</td><td>819</td><td>1552</td><td>2370</td><td>[protein=ribonucleoside-diphosphate reductase subunit alpha] [protein_id=WP_002865087.1] [location=29726..32095] [gbkey=CDS]</td><td>2370</td><td>97.80 %</td><td>54.90 %</td></tr>
<tr><td>DQM41_RS00120</td><td>lcl|NZ_LS483362.1_cds_WP_002865087.1_24</td><td>1</td><td>549</td><td>549</td><td>forward</td><td>1</td><td>549</td><td>549</td><td>1</td><td>549</td><td>[protein=ribonucleoside-diphosphate reductase subunit alpha] [protein_id=WP_002865087.1] [location=29726..32095] [gbkey=CDS]</td><td>2370</td><td>94.90 %</td><td>81.90 %</td></tr>
<tr><td>DQM41_RS02720</td><td>lcl|NZ_LS483362.1_cds_WP_002864504.1_525</td><td>43</td><td>900</td><td>858</td><td>reverse</td><td>43</td><td>900</td><td>858</td><td>43</td><td>900</td><td>[gene=fliD] [protein=flagellar filament capping protein FliD] [protein_id=WP_002864504.1] [location=510546..512474] [gbkey=CDS]</td><td>1929</td><td>99.00 %</td><td>85.80 %</td></tr>
<tr><td>DQM41_RS00190</td><td>lcl|NZ_LS483362.1_cds_WP_002866314.1_33</td><td>64</td><td>702</td><td>639</td><td>reverse</td><td>64</td><td>702</td><td>639</td><td>64</td><td>702</td><td>[protein=SIMPL domain-containing protein] [protein_id=WP_002866314.1] [location=complement(51963..52664)] [gbkey=CDS]</td><td>702</td><td>90.60 %</td><td>63.90 %</td></tr>
<tr><td>DQM41_RS05225</td><td>lcl|NZ_LS483362.1_cds_WP_010891903.1_1007</td><td>577</td><td>1500</td><td>924</td><td>forward</td><td>577</td><td>1500</td><td>924</td><td>577</td><td>1500</td><td>[protein=endonuclease MutS2] [protein_id=WP_010891903.1] [location=complement(987001..989211)] [gbkey=CDS]</td><td>2211</td><td>99.40 %</td><td>92.40 %</td></tr>
<tr><td>DQM41_RS06490</td><td>lcl|NZ_LS483362.1_cds_WP_002858432.1_1257</td><td>733</td><td>1221</td><td>489</td><td>forward</td><td>733</td><td>1221</td><td>489</td><td>733</td><td>1221</td><td>[protein=DUF2920 family protein] [protein_id=WP_002858432.1] [location=complement(1235506..1236732)] [gbkey=CDS]</td><td>1227</td><td>87.70 %</td><td>48.90 %</td></tr>
<tr><td>DQM41_RS01770</td><td>lcl|NZ_LS483362.1_cds_WP_002872052.1_346</td><td>321</td><td>614</td><td>294</td><td>forward</td><td>321</td><td>614</td><td>294</td><td>321</td><td>614</td><td>[gene=cmeB] [protein=multidrug efflux RND transporter permease subunit CmeB] [protein_id=WP_002872052.1] [location=complement(332589..335711)] [gbkey=CDS]</td><td>3123</td><td>91.80 %</td><td>29.40 %</td></tr>
<tr><td>DQM41_RS03080</td><td>lcl|NZ_LS483362.1_cds_WP_010891867.1_594</td><td>1</td><td>579</td><td>579</td><td>reverse</td><td>1</td><td>579</td><td>579</td><td>1</td><td>579</td><td>[protein=phosphate ABC transporter ATP-binding protein] [protein_id=WP_010891867.1] [location=576216..576956] [gbkey=CDS]</td><td>741</td><td>99.50 %</td><td>57.90 %</td></tr>
<tr><td>DQM41_RS05130</td><td>lcl|NZ_LS483362.1_cds_WP_010891901.1_988</td><td>1237</td><td>2232</td><td>996</td><td>reverse</td><td>1237</td><td>2232</td><td>996</td><td>1237</td><td>2232</td><td>[gene=cmeF] [protein=multidrug efflux RND transporter permease subunit CmeF] [protein_id=WP_010891901.1] [location=965475..968492] [gbkey=CDS]</td><td>3018</td><td>99.40 %</td><td>99.60 %</td></tr>
<tr><td>DQM41_RS05130</td><td>lcl|NZ_LS483362.1_cds_WP_010891901.1_988</td><td>235</td><td>1173</td><td>939</td><td>reverse</td><td>235</td><td>1173</td><td>939</td><td>235</td><td>1173</td><td>[gene=cmeF] [protein=multidrug efflux RND transporter permease subunit CmeF] [protein_id=WP_010891901.1] [location=965475..968492] [gbkey=CDS]</td><td>3018</td><td>96.20 %</td><td>93.90 %</td></tr>
<tr><td>DQM41_RS02380</td><td>lcl|NZ_LS483362.1_cds_WP_010891860.1_458</td><td>3616</td><td>4137</td><td>522</td><td>reverse</td><td>3616</td><td>4137</td><td>522</td><td>3616</td><td>4137</td><td>[gene=rpoB] [protein=DNA-directed RNA polymerase subunit beta] [protein_id=WP_010891860.1] [location=438986..443122] [gbkey=CDS]</td><td>4137</td><td>97.70 %</td><td>52.20 %</td></tr>
<tr><td>DQM41_RS02380</td><td>lcl|NZ_LS483362.1_cds_WP_010891860.1_458</td><td>1615</td><td>2613</td><td>999</td><td>reverse</td><td>1615</td><td>2613</td><td>999</td><td>1615</td><td>2613</td><td>[gene=rpoB] [protein=DNA-directed RNA polymerase subunit beta] [protein_id=WP_010891860.1] [location=438986..443122] [gbkey=CDS]</td><td>4137</td><td>99.40 %</td><td>99.90 %</td></tr>
<tr><td>DQM41_RS03640</td><td>lcl|NZ_LS483362.1_cds_WP_002793291.1_706</td><td>343</td><td>1326</td><td>984</td><td>reverse</td><td>343</td><td>1326</td><td>984</td><td>343</td><td>1326</td><td>[protein=esterase-like activity of phytase family protein] [protein_id=WP_002793291.1] [location=682515..683840] [gbkey=CDS]</td><td>1326</td><td>99.40 %</td><td>98.40 %</td></tr>
<tr><td>DQM41_RS05115</td><td>lcl|NZ_LS483362.1_cds_WP_002837927.1_985</td><td>1</td><td>627</td><td>627</td><td>forward</td><td>1</td><td>627</td><td>627</td><td>1</td><td>627</td><td>[gene=lepA] [protein=elongation factor 4] [protein_id=WP_002837927.1] [location=complement(961538..963334)] [gbkey=CDS]</td><td>1797</td><td>94.50 %</td><td>62.70 %</td></tr>
<tr><td>DQM41_RS02420</td><td>lcl|NZ_LS483362.1_cds_WP_002864166.1_466</td><td>619</td><td>1236</td><td>618</td><td>reverse</td><td>619</td><td>1236</td><td>618</td><td>619</td><td>1236</td><td>[gene=fucP] [protein=L-fucose:H+ symporter permease] [protein_id=WP_002864166.1] [location=453113..454369] [gbkey=CDS]</td><td>1257</td><td>92.20 %</td><td>61.80 %</td></tr>
<tr><td>DQM41_RS06505</td><td>lcl|NZ_LS483362.1_cds_WP_002856137.1_1260</td><td>280</td><td>849</td><td>570</td><td>forward</td><td>280</td><td>849</td><td>570</td><td>280</td><td>849</td><td>[protein=hypothetical protein] [protein_id=WP_002856137.1] [location=complement(1238565..1239635)] [gbkey=CDS]</td><td>1071</td><td>98.90 %</td><td>57.00 %</td></tr>
<tr><td>DQM41_RS04280</td><td>lcl|NZ_LS483362.1_cds_WP_002864843.1_829</td><td>1441</td><td>1509</td><td>69</td><td>forward</td><td>1441</td><td>1509</td><td>69</td><td>1441</td><td>1509</td><td>[protein=chorismate-binding protein] [protein_id=WP_002864843.1] [location=complement(807480..809264)] [gbkey=CDS]</td><td>1785</td><td>47.80 %</td><td>6.90 %</td></tr>
<tr><td>DQM41_RS05910</td><td>lcl|NZ_LS483362.1_cds_WP_002852850.1_1144</td><td>370</td><td>1365</td><td>996</td><td>reverse</td><td>370</td><td>1365</td><td>996</td><td>370</td><td>1365</td><td>[protein=cation:dicarboxylase symporter family transporter] [protein_id=WP_002852850.1] [location=1119951..1121327] [gbkey=CDS]</td><td>1377</td><td>95.70 %</td><td>99.60 %</td></tr>
</table>

Supplementary Data 2

Table S11. Methylation Motifs of *C. coli* meC0281

No.	Motif	Modified Position	Modification Type	% Motifs detected	# of Motifs detected	# of Motifs in Genome	Mean Modification QV[1]	Mean Motif Coverage	Partner Motif
A	GANTC	2	^{m6}A	100	4326	4326	291.43	216.99	GANTC
B1	CAYNNNNNCTC	2	^{m6}A	100	884	884	270.23	216.88	GAGNNNNNRTG
B2	GAGNNNNNRTG	2	^{m6}A	99.66	881	884	256.05	216.75	CAYNNNNNCTC
C	RAATTY	3	^{m6}A	99.77	30324	30394	285.27	205.13	RAATTY
D	GGGTDA	6	^{m6}A	99.65	1708	1714	281.33	211.80	

[1] QV = quality value

Table S12. Putative *C. coli* meC0281 restriction modification systems

ORF (REBASE)	Strand	Position in Genome	Description	Type/subunit	Predicted rec. seq.
298	-	222971-224458	M.Cco104626ORFFP Type I restriction-modification system, DNA-methyltransferase subunit M (EC 2.1.1.72)	I/M	
300	-	225650-226459	S1.Cco104626ORFFP Type I restriction-modification system, specificity subunit S (EC 3.1.21.3)	I/S	
301	-	226452-226985	S2.Cco104626ORFFP Type I restriction-modification system, specificity subunit S (EC 3.1.21.3)	I/S	
306	-	229942-232260	Cco104626ORFFP Type I restriction-modification system, restriction subunit R (EC 3.1.21.3)	I/R	
55/56	+	45829-49640	Cco104626ORFHP putative type IIS restriction/modification enzyme	II/RM	
775	-	579476-580291	M.Cco104626ORFAP DNA adenine methylase	II/M	GANTC
1065	+	771536-775630	Cco104626I Type II restriction-modification system, DNA-methyltransferase subunit M (EC 2.1.1.72) / Type II restriction-modification system, restriction subunit R (EC 3.1.21.3)	II/RM	GAGNNNNNRTG
1071	+	778082-781168	Cco104626IV/Cco104626ORFEP adenine specific DNA methyltransferase	II/RM	GGGTDA
2187	-	1576178-1577281	M.Cco104626III DNA modification methylase (Adenine-specific methyltransferase) (EC 2.1.1.72)	II/M	RAATTY
2294	-	1651978-1653351	Cco104626McrCP McrBC restriction endonuclease system, McrB subunit, putative	IV/R	
2295	-	1653314-1655383	Cco104626McrBP McrBC restriction endonuclease system, McrB subunit, putative	IV/R	

Note: The enzymes and motifs in this strain are very close to three of those in *Campylobacter coli* meC0280 and have been assigned accordingly.

Table S13. Methylation Motifs of *C. coli* meC0467

No.	Motif	Modified Position	Modification Type	% Motifs detected	# of Motifs detected	# of Motifs in Genome	Mean Modification QV[1]	Mean Motif Coverage	Partner Motif
A	CAGAG	4	^{m6}A	100	723	723	278.48	208.65	
B	CACNNNNNGT	2	^{m6}A	100	1087	1087	275.18	209.79	
C	DACNNNNNGTG	2	^{m6}A	100	871	871	268.34	202.71	
D	GNGGTA	6	^{m6}A	99.94	1564	1565	191.64	210.31	
E	RAATTY	3	^{m6}A	99.75	27661	27730	267.53	189.52	RAATTY
F	GGGTDA	6	^{m6}A	97.79	1550	1585	284.65	208.09	

[1] QV = quality value

Table S14. Putative *C. coli* meC0467 restriction modification systems

ORF (REBASE)	Strand	Position in Genome	Description	Type/subunit	Predicted rec. seq.
305	-	224917-226404	M.Cco104627ORFDP Type I restriction-modification system, DNA-methyltransferase subunit M (EC 2.1.1.72)	I/M	
307	-	227596-228093	S1.Cco104627ORFDP Type I restriction-modification system, specificity subunit S (EC 3.1.21.3)	I/S	
309	-	228173-228820	S2.Cco104627ORFDP Type I restriction-modification system, specificity subunit S (EC 3.1.21.3)	I/S	
314	-	231860-234178	Cco104627ORFDP Type I restriction-modification system, restriction subunit R (EC 3.1.21.3)	I/R	
51/52	+	46222-50018	Cco104627ORFFP putative type IIS restriction/modification enzyme	II/RM	CAGAG or GNGGTA
977	+	727644-731651	Cco104627ORFBP Type II restriction-modification system, DNA-methyltransferase subunit M (EC 2.1.1.72) / Type II restriction-modification system, restriction subunit R (EC 3.1.21.3)	II/RM	CACNNNNNGT
983	+	734103-737189	Cco104627II / Cco104627ORFCP adenine specific DNA methyltransferase	II/RM	GGGTDA
2026	-	1493860-1494963	M.Cco104627I DNA modification methylase (Adenine-specific methyltransferase) (EC 2.1.1.72)	II/M	RAATTY
2133		1571005-1572378	Cco104627McrCP McrBC restriction endonuclease system, McrB subunit, putative	IV/R	
2134		1572341-1574416	Cco104627McrBP McrBC restriction endonuclease system, McrB subunit, putative	IV/R	

Supplementary Data 3: Phenotypic Assays

Methods

Growth curves

Growth curves were performed in 20 mL of Mueller Hinton (MH) broth (ThermoFisher Scientific, Waltham, Massachusetts, USA) in 100 mL Erlenmeyer flasks at 37°C and 150 rotations per minute (rpm) under microaerophilic conditions. Bacteria were taken from pre-cultures grown for 24 h at 37°C under the same conditions. The ODs were adjusted by inoculation to an OD_{600nm} of 0.05 at the start of the growth experiments. The growth experiments were performed in three biological and two technical replicates [1].

Motility assay

Motility assays were performed as described by Tareen *et al*. (2010) with minor modifications [2]. The bacteria were grown in MH broth (ThermoFisher Scientific, Waltham, Massachusetts, USA) for 16 h at 37°C under microaerophilic conditions. The cultures, adjusted to an OD_{600nm} of 0.025, were then stabbed into 0.4% MH agar plates (bioMérieux, Nürtingen, Baden-Württemberg, Germany) with a 1 µL inoculation loop. Following an incubation of the plates at 37°C under microaerophilic conditions for 36 h, the diameters of the motility zones were measured with a ruler.

Eukaryotic cell invasion and adhesion

Invasion and adhesion assays were performed as described by Tareen *et al*. (2010) with minor modifications [2]. $2x10^5$ human colon carcinoma (CaCo-2) cells in Dulbecco's Modified Eagle's Medium (DMEM; 1% FCS, 1x NEA, without antibiotics) were seeded in each well of a 24-well plate and incubated for 16-18 h before the start of the experimental procedure. Bacteria were

grown on Columbia agar plates (bioMérieux, Nürtingen, Baden-Württemberg, Germany) for 16 h at 42°C under microaerophilic conditions prior to the start of the experiment. The bacteria on the Columbia agar plates were re-suspended in DMEM (without supplements) and adjusted to an OD_{600nm} of 0.0007 (corresponding to ~$2x10^6$ CFU/mL for *C. jejuni* 81-176). Bacterial suspensions of each 1 mL were added to the cell monolayers, resulting in a multiplicity of infection (MOI) of 10. The 24-well plates were centrifuged at 600 x *g* for 5 min, before incubating the infected plates at 37°C and 5% CO_2 for either 2 h for invasion assaying or 30 min for adhesion assaying. Following incubation, the CaCo-2 cell monolayers were washed three times with DMEM (without supplements). For adhesion assaying, the cell monolayer was hereafter lysed with 100 µL of 0.1% Triton X-100 in DMEM for 10 min at room temperature. The number of viable bacteria released from the cell monolayer was then determined by plating serial dilutions of the bacterial suspension on Columbia agar plates. These plates were counted after 48 h of incubation. For invasion assaying, the cells were further incubated for 2 h at 37°C and 5% CO_2 in 100 µg/mL gentamicin in DMEM (without supplements) to remove extracellular bacteria (susceptibility to gentamicin was tested before using the disc diffusion method according to the standards of the European Committee on Antimicrobial Susceptibility Testing [3]). After gentamicin treatment, the cell monolayer was washed again three times with DMEM (without supplements) and lysed with 100 µL of 0.1% Triton X-100 in DMEM to release intracellular bacteria. The number of viable bacteria in each well was again determined. Percentage invasion/adhesion was calculated by dividing the number of invaded/adhered bacteria by the number of viable bacteria that were added to the wells.

Autoagglutination assay

Autoagglutination assays were performed as described by Misawa and Blaser (2000) [4]. Bacteria grown for 16 h on Columbia agar plates (bioMérieux, Nürtingen, Baden-Württemberg, Germany) at 42°C under microaerophilic conditions were resuspended in phosphate buffered saline (PBS; pH 7.4) and adjusted to an OD_{600nm} of 1. The bacterial suspensions were then

added in 2 mL volumes into glass tubes and incubated for 24 h at 37°C under microaerophilic conditions without shaking. After incubation, 1 mL of each supernatant was carefully removed and the OD_{600nm} was measured. Relative autoagglutination was calculated by dividing the initial OD of the bacterial suspension by the final OD of the bacterial supernatant.

Biofilm assay

The biofilm assays were performed as described by Reeser *et al*. (2007) with some modifications [5]. Bacteria grown for 16 h on Columbia agar plates (bioMérieux, Nürtingen, Baden-Württemberg, Germany) at 42°C under microaerophilic conditions were resuspended in MH medium, adjusted to an OD_{600nm} of 0.05 and added in 100 µL volumes to each well of a 96-well plate. After 48 h of incubation at 37°C under microaerophilic conditions, the bacterial cultures were removed and the plate was dried for 30 min at 60°C. The wells were then stained with 0.1% crystal violet for 15 min at room temperature, after which the wells were washed twice with water and the plate was again dried for 15 min at 60°C. The bound crystal violet was decolorized with a solution of 80% ethanol and 20% acetone for 15 min and added to a fresh 96-well plate. The plate was then read at an absorbance of 570 nm with a microplate reader. Only MH medium served as the negative control.

Water survival assay

The water survival assays were performed as described by Cools *et al*. (2003) with modifications [6]. Bacteria grown for 16 h on Columbia agar plates (bioMérieux, Nürtingen, Baden-Württemberg, Germany) at 42°C under microaerophilic conditions were resuspended in sterile filtered (0.2 µm pore size) and autoclaved (120°C for 15 min) 15 mL rain water and washed once by centrifuging the suspension at 5000 x *g* for 10 min. The bacterial pellet was resuspended and the bacterial suspensions were adjusted to an OD_{600nm} of 0.05 (corresponding to approximately $2x10^8$ CFU/mL) in 5 mL of the rain water and placed into glass

tubes. The bacterial suspensions were incubated in a refrigerator at 4°C under aerobic conditions. The density of bacteria in the samples were determined before incubation (day 0) and on days 2 and 6.

Statistics

LibreOffice Calc was used to calculate the average values and standard deviations. The t-test was used to calculate the p-values and a significance level of 0.05 was set. Generation times were calculated using the GraphPad Prism 6 software (graphpad.com).

Results

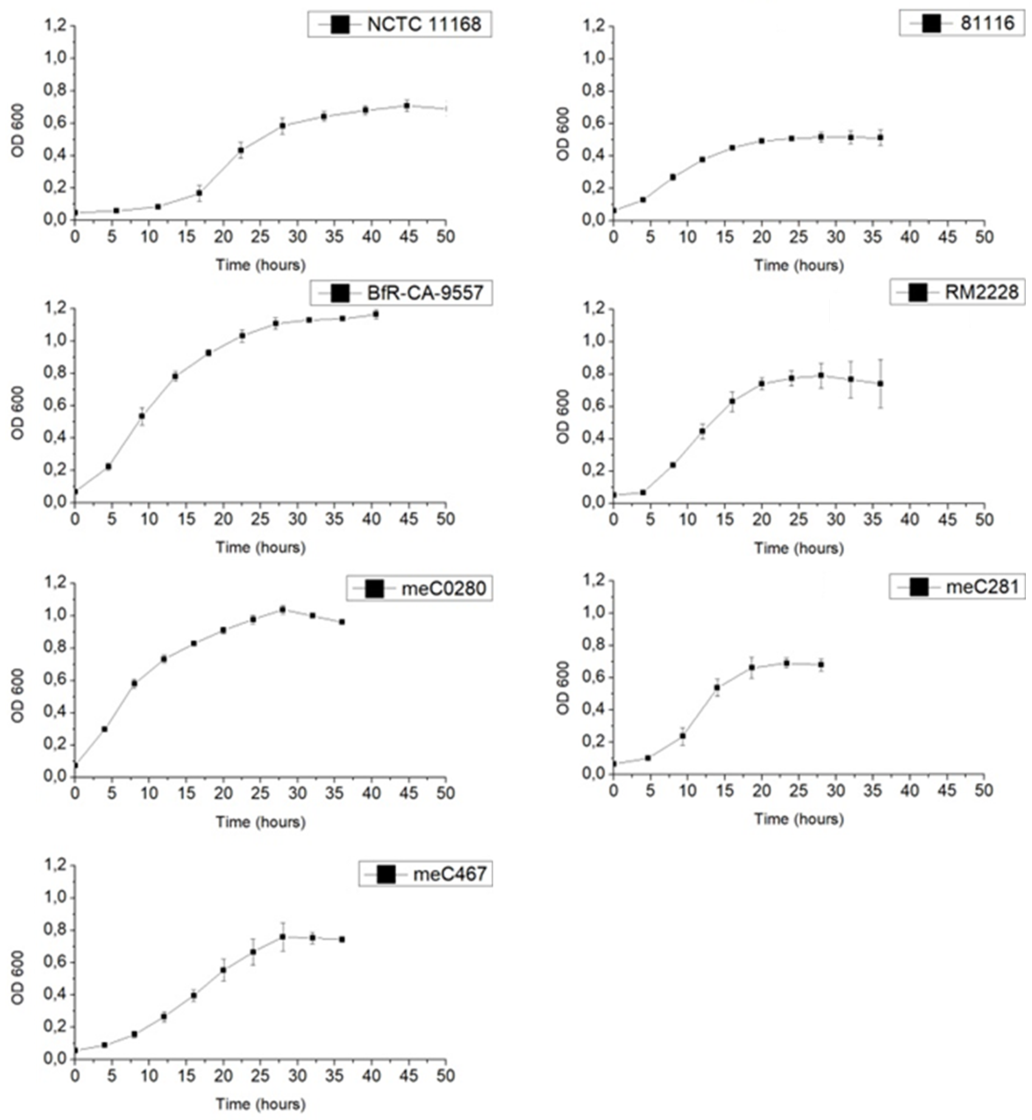

Figure S1. Growth Behaviour of tested *C. jejuni, C. coli* and *C. jejuni*/*C. coli* hybrid strains. Growth experiments were performed in MH medium at 37°C, under microaerophilic conditions and 150 rpm shaking. The data points represent the means and standard deviations of two biological

Strain	Average peak OD_{600nm}	Average time to peak OD_{600nm} (hours)	Average generation time (min)
NCTC 11168	0.58 ± 0.02	28 ± 2.00	287 ± 19
81116	0.50 ± 0.01	20 ± 3.60	228 ± 15
BfR-CA-9557	1.11 ± 0.02	27 ± 0.13	215 ± 0
RM2228	0.77 ± 0.05	24.5 ± 3.50	133 ± 6
meC0280	1.04 ± 0.02	28 ± 0.00	121 ± 1
meC0281	0.66 ± 0.01	19 ± 1.65	230 ± 30
meC0467	0.76 ± 0.04	28 ± 4.00	296 ± 35

Table S15. Peak OD_{600nm} values, times to peak OD_{600nm} (in hours) and generation times (in min) of *C. jejuni, C. coli* and *C. jejuni*/*C. coli* hybrid strains. Values represent the means ± standard deviations of three biological replicate experiments. Within the *C. jejuni* and *C. coli* reference strains as well as within the *C. coli*/*C. jejuni* hybrid strains there is a difference of up to approx. 8 h in the 'Average Time to peak'.

In the comparison of growth dynamics, meC0280 in particular stands out, which grows about twice as fast as NCTC 11168, 81116, BfR-CA-9557, meC0281 and meC0467. Only *C. coli* RM2228 shows similar growth dynamics as meC0280 with an average generation time of 133 min.

p-values for the comparison of the hybrid strains with the reference strains (average generation time):

	**NCTC 11168**	**81116**	**BfR-CA-9557**	**RM2228**	**meC0280**	**meC0281**	**meC0467**
**meC0280**	0.381	0.022	<0.001	0.024	X	0.180	0.016
**meC0281**	0.381	0.359	0.486	0.084	0.180	X	0.045
**meC0467**	0.024	0.046	0.021	0.013	0.016	0.045	X

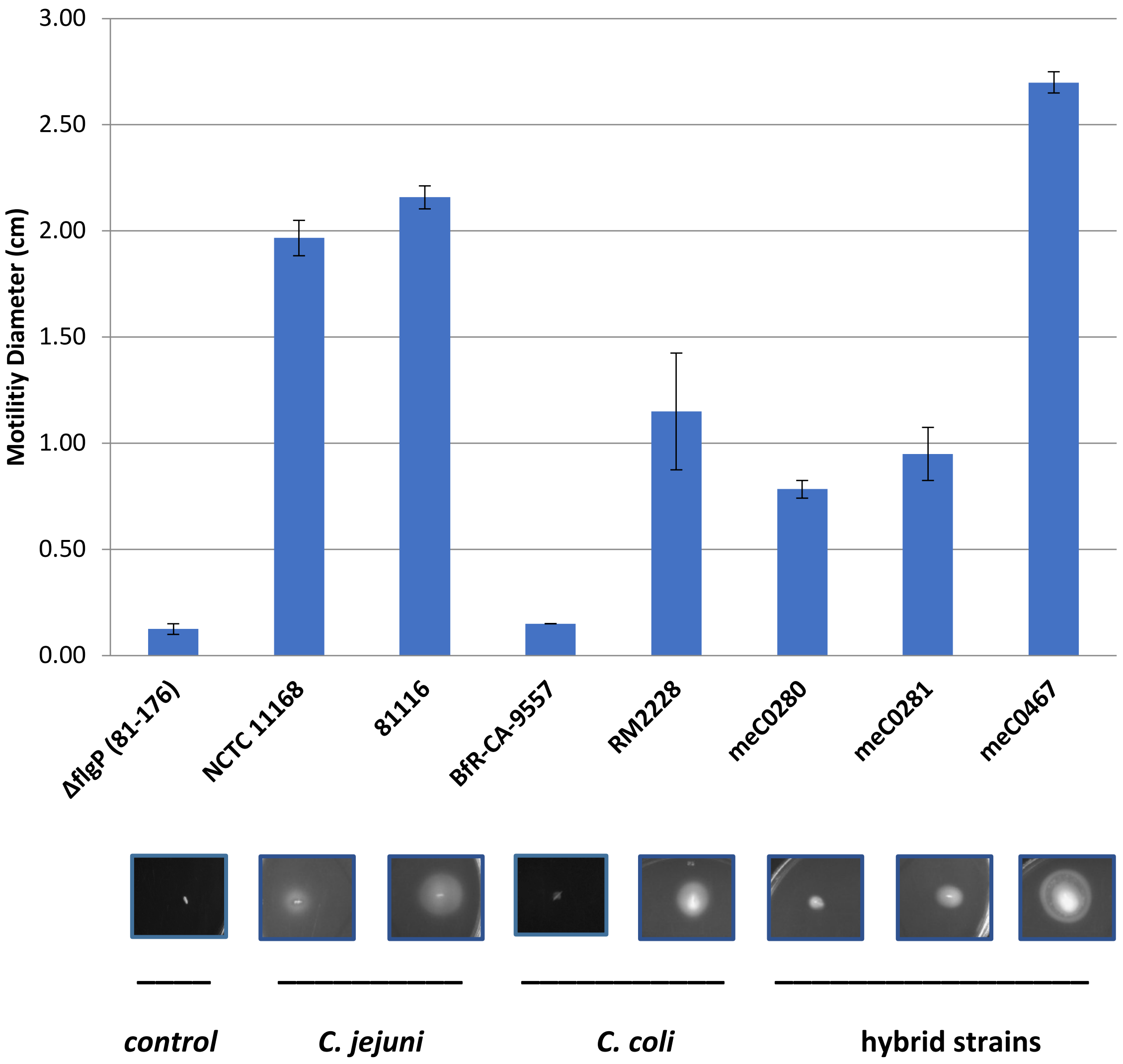

Figure S2. Motility behavior of the tested *C. jejuni, C. coli* and *C. jejuni*/*C. coli* hybrid strains. Motility assays were performed by stabbing 0.4% MH agar plates with bacteria grown for 16 h (to late log phase) in MH medium. Bars represent the means ± standard deviations of three biological replicate experiments. Each experiment was performed in two technical replicates. Below the X-axis are representative pictures of the motility zones for each strain on the MH agar plates. An aflagellar knockout mutant *C. jejuni* 81-176 Δ*flgP* functioned as a control. These pictures were taken using the Gel Imaging System (Gel Doc XR+, Bio-Rad Laboratories GmbH, Feldkirchen, Germany).
Statistical analysis revealed that the meC0280 strain is significantly ($p<0.05$) less motile compared to NCTC 11168, 81116, and meC0467. On the other hand, meC0467 is the most motile strain in our analysis, which is also significantly more motile than BfR-CA-9557 and meC0280. In conclusion, there is a high variability in motility within the hybrid strains as well as within the reference strains.

p-values for the comparison of the hybrid strains with the reference strains:

	NCTC 11168	81116	BfR-CA-9557	RM2228	meC0280	meC0281	meC0467
**meC0280**	0.048	0.012	0.083	0.625	X	0.625	0.005
**meC0281**	0.093	0.090	0.193	0.783	0.625	X	0.059
**meC0467**	0.086	0.067	0.025	0.208	0.005	0.059	X

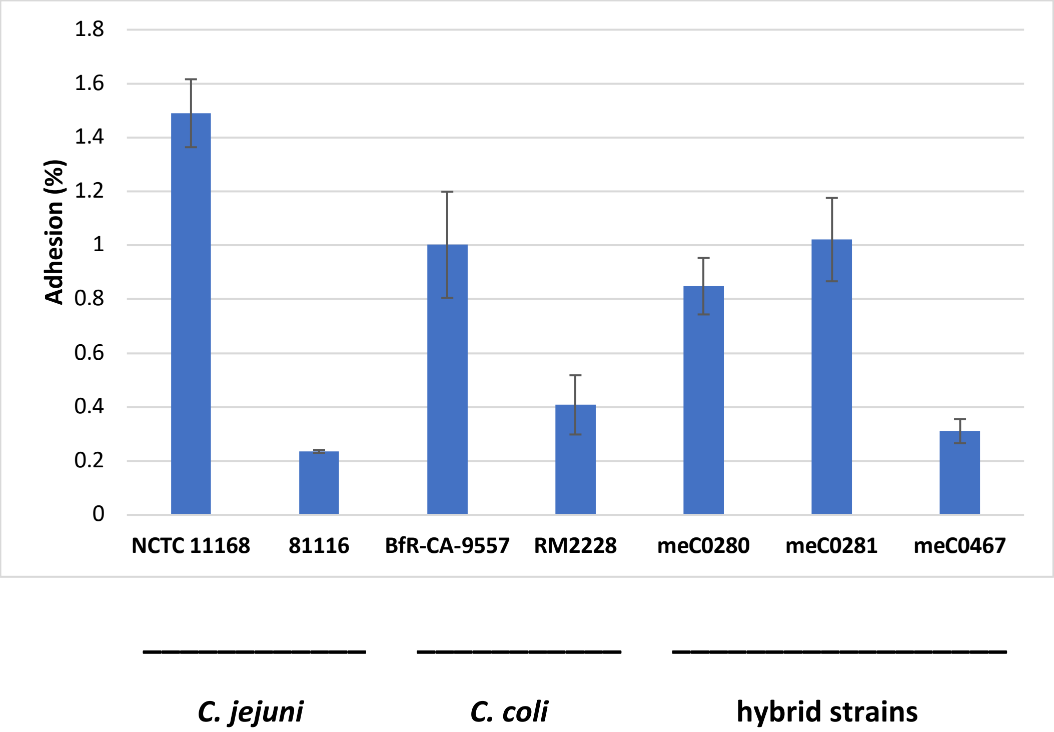

Figure S3. *In vitro* adhesion behavior of the tested *C. jejuni, C. coli* and *C. jejuni*/*C. coli* hybrid strains. Adhesion experiments were performed on CaCo-2 cells at 37°C and with a multiplicity of infection (MOI) of 10. Adhesion values were calculated as a percentage of the CFU of inoculated bacteria. Bars represent the means ± standard deviations of three biological replicate experiments. Each experiment was performed in two technical replicates.

The differences in adhesion to Caco-2 cells between the individual isolates tested were in the low significant range ($p<0.05$) or not significant.

p-values for the comparison of the hybrid strains with the reference strains:

	**NCTC 11168**	**81116**	**BfR-CA-9557**	**RM2228**	**meC0280**	**meC0281**	**meC0467**
**meC0280**	0.031	0.014	0.433	0.055	X	0.321	0.022
**meC0281**	0.080	0.019	0.923	0.045	0.321	X	0.025
**meC0467**	0.006	0.144	0.040	0.364	0.022	0.025	X

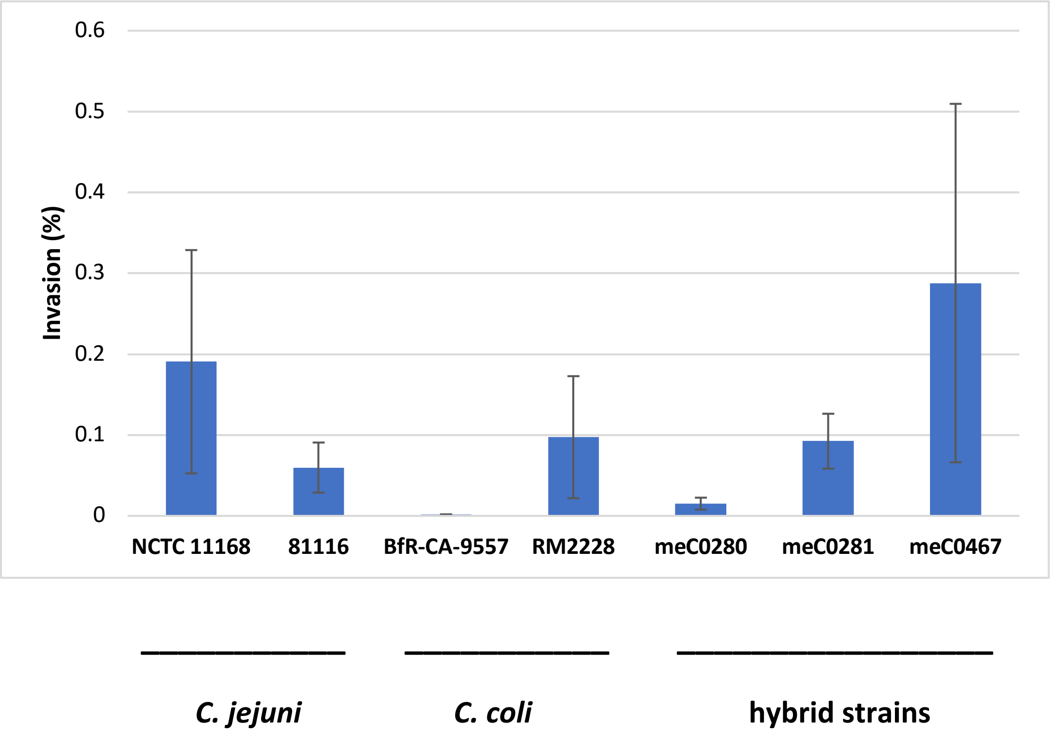

Figure S4. *In vitro* invasion behavior of the tested *C. jejuni, C. coli* and *C. jejuni*/*C. coli* hybrid strains. Invasion experiments were performed on CaCo-2 cells at 37°C and with a multiplicity of infection (MOI) of 10. Invasion values were calculated as a percentage of the CFU of infecting bacteria that were added to the experiment. Bars represent the means ± standard deviations of three biological replicate experiments. Each experiment was performed in two technical replicates.
There were no significant ($p>0.05$) differences in Caco-2 cell invasion when comparing the hybrid strains with the reference strains. Hyperinvasive strains (invasiveness 25x strain 81116) were not observed in this test setting.

p-values for the comparison of the hybrid strains with the reference strains:

	NCTC 11168	81116	BfR-CA-9557	RM2228	meC0280	meC0281	meC0467
**meC0280**	0.215	0.186	0.121	0.265	X	0.088	0.224
**meC0281**	0.432	0.421	0.063	0.940	0.088	X	0.343
**meC0467**	0.651	0.286	0.209	0.369	0.224	0.343	X

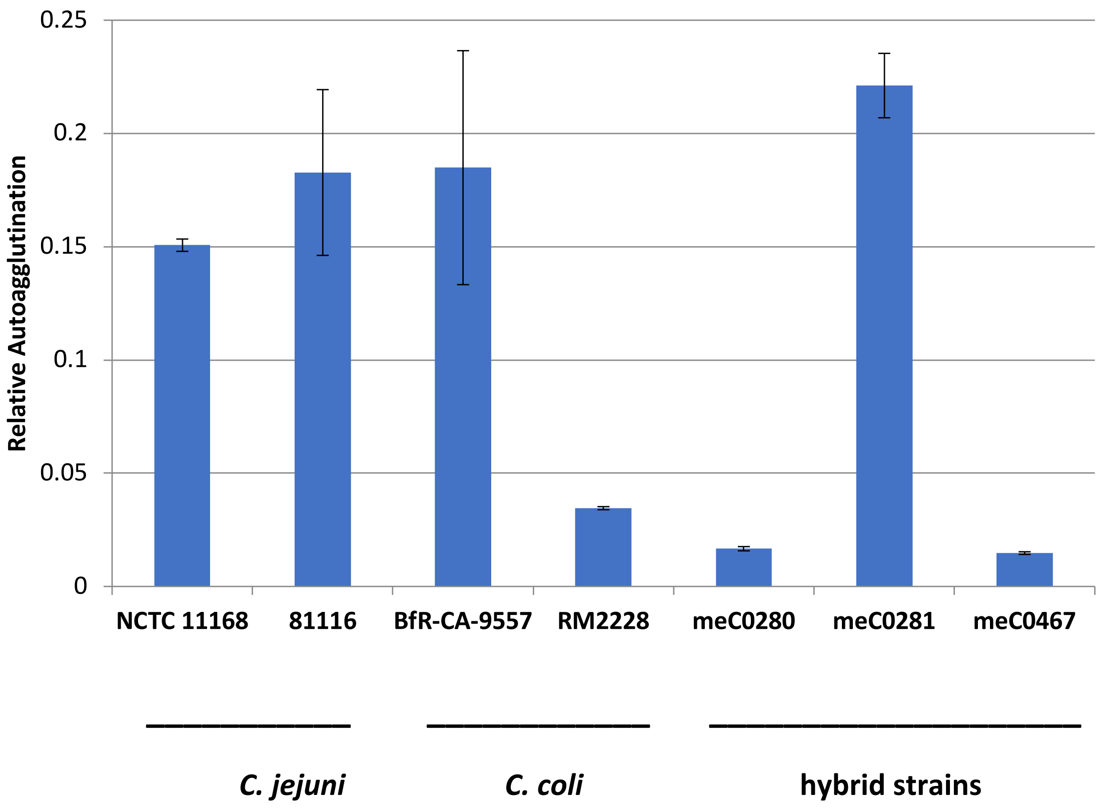

Figure S5. Autoagglutination behavior of the tested *C. jejuni, C. coli* and *C. jejuni*/*C. coli* hybrid strains. Autoagglutination experiments were performed in PBS (pH 7.4) at 37°C with bacteria grown for 16 h on COS plates at 42°C prior to the experiment. Bacteria were left to autoagglutinate for 24 h prior to measurement. Bars represent the means ± standard deviations of three biological replicate experiments. Each experiment was performed in two technical replicates.

The meC0281 strain presented a significantly ($p<0.05$) higher amount of autoagglutination than the strains meC0280, meC0467 and RM2228. However, there were no significant differences when compared to the three reference strains NCTC 11168, 81116, and BFR-CA-9557. There were similar differences in autoagglutination between the two *C. coli* reference strains as within the group of the hybrid strains.

p-values for the comparison of the hybrid strains with the reference strains:

	NCTC 11168	81116	BfR-CA-9557	RM2228	meC0280	meC0281	meC0467
**meC0280**	0.006	0.138	0.190	0.006	X	0.043	0.244
**meC0281**	0.116	0.476	0.609	0.048	0.043	X	0.043
**meC0467**	0.009	0.136	0.188	0.002	0.244	0.043	X

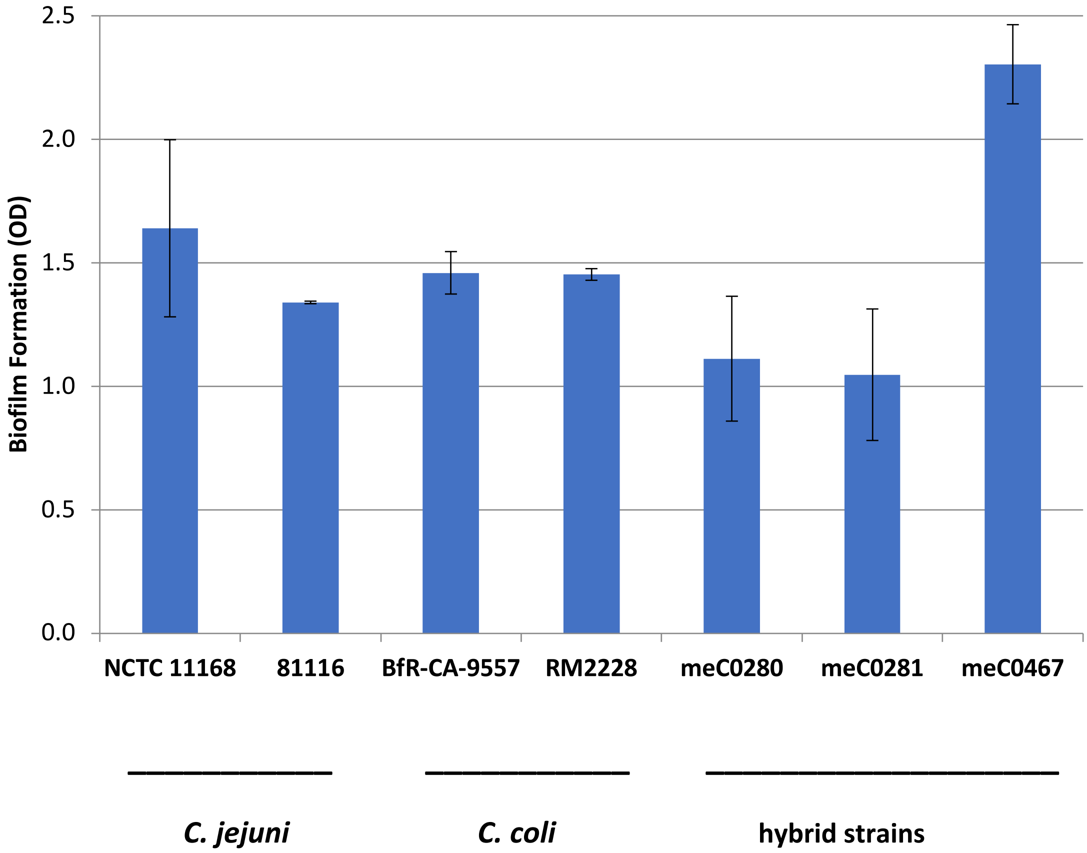

Figure S6. Biofilm formation by the tested *C. jejuni, C. coli* and *C. jejuni/C. coli* hybrid strains. Biofilm assays were performed in PBS (pH 7.4) at 37°C with bacteria grown for 16 h on COS agar plates at 42°C prior to the experiment. Bacteria were incubated for 48 h prior to measurement. Bars represent the means ± standard deviations of three biological replicate experiments. Each experiment was performed in two technical replicates.

Strain meC0467 showed significantly (p=0.001 or 0.002) higher biofilm formation in our experiment compared to all other isolates tested except NCTC 11168. Furthermore, the differences in biofilm formation among the other isolates were not significantly different.

p-values for the comparison of the hybrid strains with the reference strains:

	**NCTC 11168**	**81116**	**BfR-CA-9557**	**RM2228**	**meC0280**	**meC0281**	**meC0467**
**meC0280**	0.160	0.216	0.093	0.100	X	0.770	<0.001
**meC0281**	0.128	0.153	0.069	0.076	0.770	X	<0.001
**meC0467**	0.106	0.002	0.001	0.002	<0.001	<0.001	X

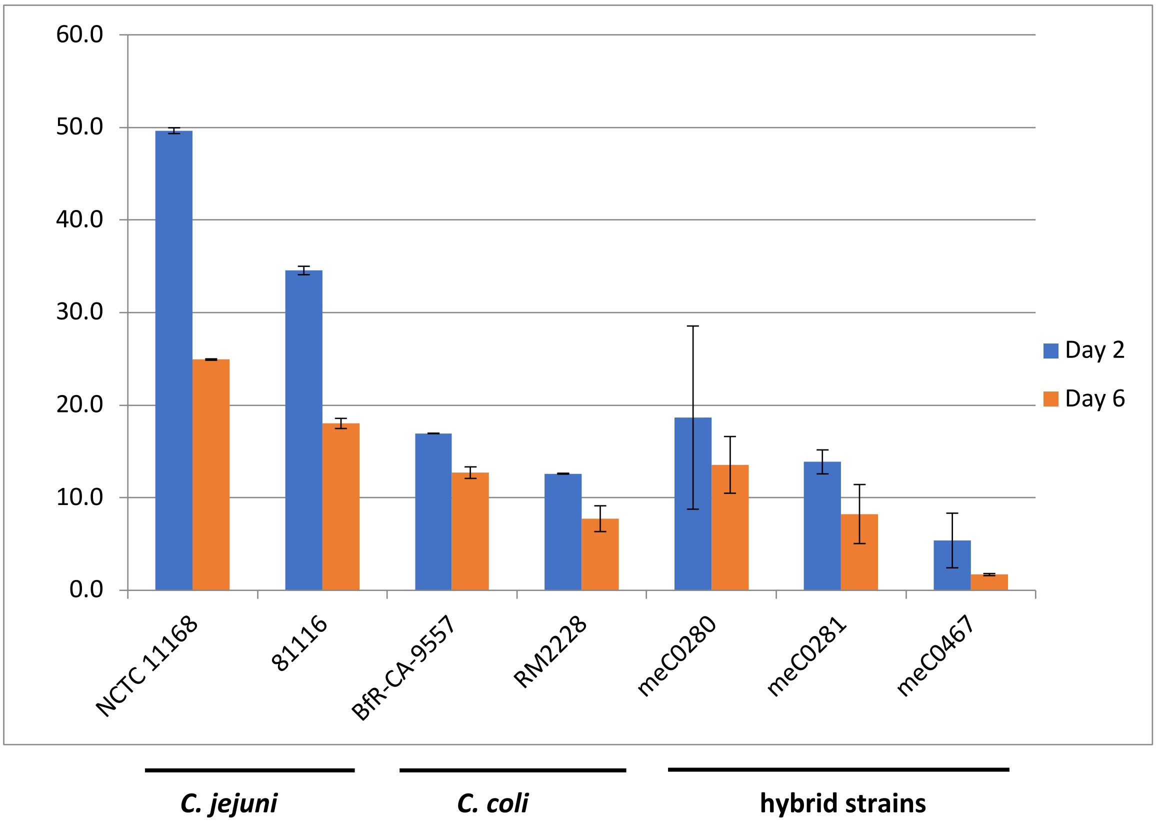

Figure S7. Water survival ability of tested *C. jejuni, C. coli* and *C. jejuni*/*C. coli* hybrid strains. Water survival experiments were performed in rain water in glass tubes stored at 4°C. The percentage of water survival after 2 and 6 days refers to the bacterial CFU/mL in the glass tubes after 2 and 6 days of incubation as a percentage of the CFU/ml in the tubes at day 0.

After two days of incubation, water survival abilities of the hybrid strains meC0281 and meC0467 were significantly lower than the water survival abilities of *C. jejuni* NCTC 11168 ($p < 0.05$). Additionally, meC0281 showed a significant ($p<0.05$) lower water survival compared to *C. jejuni* 81116. When the experiment was extended to 6 days, there was significantly lowered water survival when comparing meC0467 and both NCTC 11168 and 81116.

p-values (day 2) for the comparison of the hybrid strains with the reference strains:

	NCTC 11168	81116	BfR-CA-9557	RM2228	meC0280	meC0281	meC0467
**meC0280**	0.196	0.354	0.891	0.650	X	0.713	0.395
**meC0281**	0.017	0.023	0.254	0.506	0.713	X	0.172
**meC0467**	0.040	0.059	0.159	0.246	0.395	0.172	X

p-values (day 6) for the comparison of the hybrid strains with the reference strains:

	NCTC 11168	81116	BfR-CA-9557	RM2228	meC0280	meC0281	meC0467
**meC0280**	0,167	0,376	0,830	0,277	X	0,353	0,161
**meC0281**	0,120	0,192	0,388	0,904	0,353	X	0,288
**meC0467**	<0,001	0,017	0,031	0,142	0,161	0,288	X

References

1. **Lübke A-L, Minatelli S, Riedel T, Lugert R, Schober I, *et al.*** The transducer-like protein Tlp12 of *Campylobacter jejuni* is involved in glutamate and pyruvate chemotaxis. *BMC Microbiol*;18:111.

2. **Tareen AM, Dasti JI, Zautner AE, Groß U, Lugert R**. *Campylobacter jejuni* proteins Cj0952c and Cj0951c affect chemotactic behaviour towards formic acid and are important for invasion of host cells. *Microbiology* 2010;156:3123–35.

3. **Ge B, Wang F, Sjolund-Karlsson M, McDermott PF**. Antimicrobial resistance in *Campylobacter*: susceptibility testing methods and resistance trends. *J Microbiol Methods* 2013;95:57–67.

4. **Misawa N, Blaser MJ**. Detection and characterization of autoagglutination activity by *Campylobacter jejuni*. *Infect Immun* 2000;68:6168–6175.

5. **Reeser RJ, Medler RT, Billington SJ, Jost BH, Joens LA**. Characterization of *Campylobacter jejuni* biofilms under defined growth conditions. *Appl Environ Microbiol* 2007;73:1908–1913.

6. **Cools I, Uyttendaele M, Caro C, D'Haese E, Nelis HJ, *et al.*** Survival of *Campylobacter jejuni* strains of different origin in drinking water. *J Appl Microbiol* 2003;94:886–892.
